# Antimicrobial Peptide‐Peptoid Macrocycles from the Polymyxin B2 Chemical Space

**DOI:** 10.1002/anie.202501299

**Published:** 2025-04-03

**Authors:** Etienne Bonvin, Markus Orsi, Thierry Paschoud, Ashvin Gopalasingam, Jérémie Reusser, Thilo Köhler, Christian van Delden, Jean‐Louis Reymond

**Affiliations:** ^1^ Department of Chemistry Biochemistry and Pharmaceutical Sciences University of Bern Freiestrasse 3 Bern CH‐3012 Switzerland; ^2^ Department of Microbiology and Molecular Medicine University of Geneva Geneva CH‐1211 Switzerland

**Keywords:** Antimicrobials, Chemical space, Macrocycles, Natural products, Peptoids

## Abstract

Macrocycles have emerged as important new modalities in drug discovery. In the context of addressing the global threat of antimicrobial resistance, here we used a genetic algorithm as a computational tool to evolve peptide‐peptoid macrocycles to resemble polymyxin B2 (**PMB2**), a macrocyclic lipopeptide natural product used as last resort antibiotic. Synthesis and testing of 41 **PMB2** analogs revealed several peptide‐peptoid macrocycles showing strong, although salt sensitive, activity against *Escherichia coli* and multidrug‐resistant strains of *Pseudomonas aeruginosa*, high serum stability, and lower toxicity to kidney cells compared to **PMB2**. These macrocycles resembled **PMB2** in terms of outer membrane permeabilization, inner membrane depolarization, lipopolysaccharide binding, and loss of activity when linearized, but, unlike **PMB2**, induced aggregation of intracellular contents, an effect was reported for other antimicrobial peptoids. These experiments exemplify a combined computational and experimental approach which might be generally useful to explore the chemical space of macrocyclic peptide natural products.

## Graphical abstract

We optimized mixed peptide‐peptoid sequences for molecular similarity to the macrocyclic lipopeptide antibiotic polymyxin B2 (PMB2) using a genetic algorithm. Synthesis and testing of top‐scoring compounds allowed us to identify new macrocycles with similar antimicrobial activity and reduced toxicity compared to PMB2. Our approach might be generally useful to explore the chemical space of macrocyclic natural products.

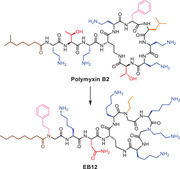

Peptide macrocycles, often inspired from natural products^[^ ^1^, ^2^ ^]^ and including peptoid (*N*‐alkylated glycines)^[^ ^3^, ^4^ ^]^ units to increase protease stability and enable oral bioavailability and cellular uptake,^[^ ^5^, ^6^, ^7^, ^8^, ^9^, ^10^ ^]^ have emerged as important new modalities in drug discovery.^[^ ^11^, ^12^, ^13^, ^14^, ^15^, ^16^ ^]^ Herein, we report a computational approach to discover analogs of macrocyclic peptide natural products based on a genetic algorithm evolving sequences for structural similarity to a target.^[^ ^17^, ^18^ ^]^ Starting from the macrocyclic lipopeptide natural product polymyxin B2 (**PMB2**), a last resort membrane targeting antimicrobial peptide^[^ ^19^, ^20^ ^]^ used to treat infections by multidrug‐resistant (MDR) bacteria but exhibiting kidney toxicity,^[^ ^21^, ^22^, ^23^ ^]^ we identify peptide‐peptoid macrocycles with comparable antimicrobial activity and reduced toxicity compared to **PMB2**.

To generate macrocyclic analogs of **PMB2**, we used our peptide design genetic algorithm (PDGA),^[^ ^24^, ^25^ ^]^ which evolves random amino acid sequences for shortest distance to a target molecule.^[^ ^26^, ^27^, ^28^ ^]^ We calculated the distance in terms of shape and pharmacophore similarity using the molecular fingerprint MXFP, identified in a preliminary computational study as suitable to drive PDGA toward peptide macrocycles.^[^ ^28^ ^]^ Aiming for analogs different from those identified by classical mutagenesis studies on **PMB2**,^[^ ^29^, ^30^, ^31^ ^]^ we removed all amino acids present in **PMB2** from the list of PDGA building blocks, except for the diaminobutyric acid used for cyclization, which we preserved to provide a common synthetic route. Instead, we introduced 33 peptoid units featuring aromatic, aliphatic, cationic, or polar side chains related to those in **PMB2** and 12 fatty acids which PDGA might add at the *N*‐terminus (Figures 1a and S1).

**Figure 1:**
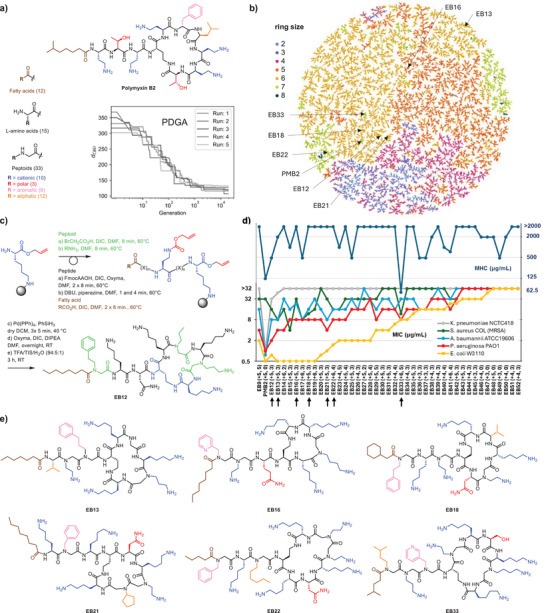
Selection of antimicrobial peptide‐peptoid macrocycles resembling polymyxin B2 (PMB2). a) Structure of PMB2 and abbreviated list of building blocks used by PDGA (side chain color code: fatty acid in brown, cationic in blue, polar in red, aromatic in pink, and aliphatic in orange), and overall minimum Manhattan distance achieved up to each generation across five parallel PDGA runs. b) TMAP visualization of the 63 066 macrocycles with shortest distance to PMB2, color‐coded by the number of residues in the macrocycle. An interactive version of the TMAP including further color‐codes is accessible at: https://tm.gdb.tools/map4/PDGA_PMB2/. c) Solid‐phase synthesis of peptide‐peptoid macrocycles on 2‐chlorotrityl chloride (2‐CTC) resin at the example of EB12. Color code: peptoids in green, amino acids in black, fatty acid in brown, protecting groups in red. See supporting information for procedures, yields, and HPLC and MS data on all macrocycles. d) Activity profiling of peptide‐peptoid hybrids. The net charge and the number of peptoid building blocks of each compound is given in brackets. The minimal inhibitory concentration (MIC, lower lines) values were measured in 12.5% Mueller‐Hinton broth at pH 7.4, after 16–20 h of incubation at 37°C. The minimal hemolytic concentration (MHC, upper dark blue line) value against human red blood cells was measured in 10 mM phosphate buffer, 150 mM NaCl, pH 7.4, 25°C for 4 h. All values are in µg/mL and represent two different duplicate MIC or MHC determinations. e) Structural formula of selected macrocycles. Residues are color‐coded as in Figure 1a.

We performed five PDGA runs of 12 h, during which each run performed over 10 000 generations, producing in total over 1.5 million unique sequences. For a closer analysis, we selected in each run the 20 000 sequences with the shortest MXFP distance to **PMB2** and retained only cyclic sequences, which resulted in 63 066 macrocycles. Almost all these macrocycles had evolved to display **PMB2** features, namely a fatty acid at the *N*‐terminal linear extension (98%), a single aromatic residue (100%), and a comparable number of cationic side chains (4.2 ± 0.7, **PMB2**: 5) which were mostly (90.3%) primary amines. In contrast to **PMB2** however, the aromatic residue was most often located in the acyclic stretch (80.3%), and the molecules only contained one or no polar residue (0.5 ± 0.6, **PMB2**: 2), incorporated peptoid residues (2.3 ± 1.1, **PMB2**: 0), and had slightly smaller sizes overall (9.6 ± 0.7 residues, **PMB2**: 11) and in the macrocycle (5.3 ± 1.2 residues, **PMB2**: 7, Figures 1b and S2). These differences reflected the imperfect encoding of molecular shape by the molecular fingerprint MXFP used to drive PDGA.

For biological evaluation, we selected 41 macrocycles featuring a common Lys‐Dab ring closure chemistry (40.1% of the PDGA‐generated macrocycles) accessible by high‐temperature mixed peptide‐peptoid solid‐phase synthesis starting from a common lysine allyl ester attached to a 2‐chlorotrityl chloride (2‐CTC) resin via its side chain (Figure 1c).^[^ ^32^ ^]^ Although **PMB2** contains a single D‐residue, we used only L‐amino acids for our synthesis for simplicity, also considering that the MXFP similarity measure used to drive the PDGA did not take chirality into account. To reflect the overall composition of the PDGA‐generated library, we chose sequences featuring mostly a single aromatic residue in the acyclic stretch, primary amines as cationic side chains, and none or a single polar residue. Thirty‐six sequences contained two or more peptoid units and five sequences contained only amino acids (Tables S1 and S2).

Several macrocycles with two or three peptoid units and five or six cationic side chains exhibited good activities against the Gram‐negative bacteria *Escherichia coli, Pseudomonas aeruginosa*, and to a less extent *Acinetobacter baumannii* and the Gram‐positive methicillin‐resistant *Staphylococcus aureus* (MRSA) when tested in diluted medium (12.5% MH), which are conditions favoring the uptake of antibacterial compounds such as non‐lytic proline‐rich antimicrobial peptides^[^ ^33^, ^34^, ^35^, ^36^, ^37^ ^]^ and peptide‐peptoid hybrids,^[^ ^32^ ^]^ and better reproduce the physiological conditions.^[^ ^38^ ^]^ While weaker than **PMB2**, these macrocycles were clearly more active than **EB9**, a previously reported linear peptide‐peptoid used here as a second positive control.^[^ ^32^ ^]^ Furthermore, almost all macrocycles showed much lower hemolysis of red blood cells than **PMB2**, except for **EB33** which was slightly more hemolytic than **PMB2** (Figure 1d, upper dark blue line, Table S3).

Six of the most active compounds (**EB12**, **EB13**, **EB16**, **EB18**, **EB21**, **EB22,** Figure 1e) were further evaluated against additional bacteria including MDR *P. aeruginosa* strains under slightly alkaline conditions (pH 8.5), which enhance the activity of **PMB2**,^[^ ^39^ ^]^ and showed activities in many cases comparable to **PMB2** and **EB9** except for *Klebsiella pneumoniae* (Table 1). Furthermore, these macrocycles did not show any significant degradation in human serum, as expected from the protective effect of peptoid units and their macrocyclic structure (Figure 2a), and were significantly less toxic against HK‐2 cells (human kidney cells) and HEK293 cells than the parent compound **PMB2** or the hemolytic macrocycle **EB33** (Figure 2b). This lower toxicity was probably related to their low membrane disruptive activity, as assessed for the three most active and least toxic macrocycles **EB12**, **EB13,** and **EB21** by fluorescein leakage assays from vesicles composed of both egg yolk phosphatidyl glycerol (EYPG) mimicking the anionic bacterial membrane and egg yolk phosphatidyl choline (EYPC) mimicking eukaryotic membranes (Figure 2c).

**Table 1:** Activities against additional bacteria.

	MIC compounds (µg mL^−1^)^a)^							
Bacterial strains/isolates	EB9	PMB2	EB12	EB13	EB16	EB18	EB21	EB22
*Pseudomonas aeruginosa* PA14	2	0.25	2	2	4	8	4	4
*Pseudomonas aeruginosa* ZEM‐1A^b)^	2–4	1	2	2	2	2–4	4	2
*Pseudomonas aeruginosa* ZEM9A^b)^	2	1–2	4	8	16	16	16	8
*Enterobacter cloacae*	16	1–2	16	16	2–4	4	16	4
*Klebsiella pneumoniae* (OXA‐48)	32	1	32	>32	>32	>32	>32	32
*Stenotrophomonas maltophilia*	8	2	2–4	2–4	16	8	4	4
*Staphylococcus epidermidis*	2	2	1	1	1	1	1	1

^a)^ Minimum inhibitory concentration (MIC), in µg/mL, was determined on bacteria in diluted Mueller‐Hinton (12.5% MH) broth, at pH 8.5, after incubation for 16–20 h at 37 °C. Values represent two different duplicate MIC determinations.

^b)^ Multidrug‐resistant clinical isolates (MDR).

**Figure 2:**
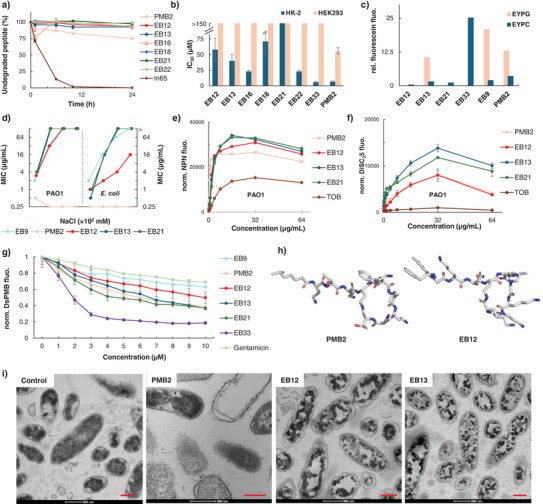
Evaluation of antimicrobial peptide‐peptoid macrocycles. a) Stability of the macrocycles (200 µM) against proteolysis in human serum (12.5%, in 0.1 M Tris buffer, pH 7.4) at 37°C. The values of undegraded peptide were determined by liquid chromatography (LC) analysis using 4‐hydroxybenzoic acid as internal standard. ln65 is a membrane disruptive antimicrobial undecapeptide used as positive control.^[^ ^44^ ^]^ b) Toxicity on HK‐2 and HEK293 cells, IC_50_ values in µM measured by Alamar blue assay after 24 h. See Table S5 and Figures S3 and S4. c) Fluorescein leakage assay from vesicles consisting of egg yolk phosphatidyl glycerol (EYPG) or egg yolk phosphatidyl choline (EYPC). The compounds (10 µg mL^−1^) were added to a suspension of carboxyfluorescein‐loaded vesicles, EYPG or EYPC, in buffer (10 mM Tris, 107 mM NaCl, pH 7.4). The percentage of fluorescein leakage is observed after 220 s. See Figure S5. d) Antibacterial activities of the compounds, against *P. aeruginosa* PAO1 and *E. coli*, measured as a function of NaCl concentration in 12.5% Mueller‐Hinton broth at pH 8.5. See Tables S6 and S7. e) Outer membrane permeability assay with 10 µM NPN, on *P. aeruginosa* PAO1. See Figure S6 for data on *E. coli*. f) Inner membrane depolarization assay with 2 µM DiSC_3_(5), on *P. aeruginosa* PAO1. See Figure S7 for data on *E. coli*. g) Dansyl‐PMB (DsPMB) displacement assay, performed at 37°C at 2.5 µM LPS. The fluorescence emission was recorded at 485 nm and normalized with the respective mean emission value without the presence of the DsPMB in the sample. See also Figure S8/S9. h) Molecular models of PMB2 and the macrocycle EB12. The conformations were generated using the software CORINA.^[^ ^45^ ^]^ i) TEM micrographs of *P. aeruginosa* PAO1 (OD_600_ = 0.5) taken after treatment with the corresponding compound (10 × MIC) in 12.5% MH at pH 8.5 and 2 h of incubation at 37°C. Scale bars are 500 nm. See Figure S10 for all micrographs. Data for toxicity, NPN, DiSC_3_(5), and DsPMB represent mean ± SD, *n* = 3. See Supporting Information for details.

In contrast to **PMB2**, the antibacterial activity of **EB12**, **EB13,** and **EB21** against both *P. aeruginosa* and *E. coli* was inhibited by high salt concentrations, similar to the reference antimicrobial linear peptide‐peptoid **EB9** (Figure 2d). Although the effect occurred across analogs with different numbers of positive charges (+3: **EB36**, **EB37**, +4: **EB23**, **EB26**, **EB32**, **EB34**, +5: **EB16**, **EB20**, +6: **EB14**, **EB22**), salt inhibition suggested that binding to bacteria was primarily mediated by electrostatic interactions (Tables S6 and S7). Nevertheless, all three macrocycles permeabilized the outer bacterial membrane to a similar extent as **PMB2** as assessed by the *N*‐phenyl‐1‐naphthylamine (NPN) fluorescence assay^[^ ^40^ ^]^ with *P. aeruginosa* PAO1 cells (Figure 2e). Likewise, all three compounds induced a depolarization of the inner membrane like **PMB2** as measured by the 3,3′‐Dipropylthiadicarbocyanine (DISC_3_(5)) assay (Figure 2f).^[^ ^41^ ^]^ Furthermore, a fluorescence displacement assay with dansyl‐PMB indicated that these three macrocycles bound to lipopolysaccharide,^[^ ^42^, ^43^ ^]^ the main target of **PMB2**, to a similar extent as **PMB2** itself, compared to **EB9** or gentamycin used as negative controls, while the hemolytic macrocycle **EB33** gave an even stronger signal in this assay (Figure 2g).

The data above might suggest a similar mechanism of action of our macrocycles and **PMB2** in line with their overall similar molecular shape when comparing 3D‐models of **PMB2** and **EB12** generated using CORINA (Figure 2h).^[^ ^45^ ^]^ The role of the macrocyclic structure in activity was evidenced by the fact that the linearized analogs **EB12lin** and **EB13lin** were between two‐fold and eight‐fold less active than their macrocyclic parent **EB12** and **EB13**, similar to the reduction in activity observed when linearizing **PMB2** to **PMB2lin** (Table S4).^[^ ^46^ ^]^ Nevertheless, transmission electron microscopy (TEM) images of *P. aeruginosa* cells exposed to the two most active compounds **EB12** and **EB13** showed patterns very different from the membrane disruptive effects seen with **PMB2**. These TEM images indicate aggregation of intracellular contents, a mechanism which is consistent with their low membrane disruptive activity measured by vesicle leakage assay and resembles that reported for other peptoids (Figure 2i).^[^ ^47^, ^48^ ^]^

In summary, the experiments mentioned above illustrate the potential of a computationally guided search to explore the chemical space of macrocyclic peptide natural products. We used a genetic algorithm to evolve macrocycles to resemble **PMB2** as measured by molecular fingerprint similarity, however differing substantially in their sequence since all **PMB2** residues were excluded from the building blocks available to the algorithm except the cyclizing Dab, and peptoid units were introduced. By testing a small number of the generated molecules accessible by a common synthetic route, we identified macrocycles with structures similar to **PMB2**, such as **EB12** and **EB13**, which displayed strong activities against *E. coli* and MDR strains of *P. aeruginosa*, showed lower toxicity to kidney cells than **PMB2**, and acted by a different mechanism. While providing an important proof‐of‐concept for the approach, the activities observed were sensitive to high salt, precluding further development. More potent macrocycles might be accessible by using different scoring functions to drive the genetic algorithm and an expanded set of building blocks and building block coupling chemistries.

## Supporting Information

The authors have cited additional references within the Supporting Information.^[^ ^49^, ^50^, ^51^, ^52^, ^53^ ^]^

## Conflict of Interests

The authors declare no conflict of interest.

## Supporting information



Supporting Information S1

Supporting Information S2

## Data Availability

The data that support the findings of this study are available in the supplementary material of this article.
