# Supplementary material for: Antimicrobial Peptide‐Peptoid Macrocycles from the Polymyxin B2 Chemical Space
Source: Angew Chem Int Ed Engl. 2025 Apr 3;64(23):e202501299. doi: 10.1002/anie.202501299 (PMC12124432; doi:10.1002/anie.202501299)
Supplement: Supplementary file 1 — Supporting Information S1 [file ANIE-64-e202501299-s002.pdf]

Supporting Information for: Antimicrobial Peptide-Peptoid Macrocycles from the Polymyxin B2 Chemical Space

Etienne Bonvin,[1] Markus Orsi,[1] Thierry Paschoud,[1] Ashvin Gopalasingam,[1] Jérémie Reusser,[1] Thilo Köhler,[2,3] Christian van Delden[2,3] and Jean-Louis Reymond*

[1]Department of Chemistry, Biochemistry and Pharmaceutical Sciences, University of Bern, Freiestrasse 3, CH-3012 Bern, Switzerland.
[2]Department of Microbiology and Molecular Medicine, University of Geneva, CH-1211 Geneva, Switzerland.
[3]Service of Infectious Diseases, University Hospital of Geneva, CH-1205 Geneva, Switzerland.

1. Peptide Design Genetic Algorithm (PDGA)

Our previously developed Peptide Design Genetic Algorithm (PDGA)[1,2] was implemented to design optimized peptide sequences by simulating an evolutionary process. The algorithm starts with the initialization of a population of 200 linear peptide sequences, which were randomly generated using a building block list that included all natural amino acids (excluding those found in polymyxin), 33 peptoid building blocks, and 12 possible N-terminal fatty acids. Peptides were encoded in a format specifying the topology ("chain" or "c_to_n_cyclization"), the sequence of building blocks (automatically numbered), and the modifications at both the N-terminus (none or a fatty acid) and C-terminus (none or amide).

Fitness evaluation was performed by encoding each peptide into a 217-dimensional MXFP fingerprint[3,4] and calculating the Manhattan distance between the generated peptides and the query, represented by the MXFP of polymyxin. The top 100 peptides with the smallest distances were selected. These selected peptides underwent genetic operations, beginning with single-point crossover to generate offspring, followed by random mutations, including residue insertion, substitution, deletion, modification of peptide topology, N-terminal modifications with fatty acids, and C-terminal modification to amide.

The algorithm terminated after 12 hours of runtime or if the query sequence was found, the latter not being achievable in this case. To account for the stochastic nature of the genetic algorithm, five independent runs were conducted in parallel for 12 hours each. A virtual screening process was applied to the 20,000 sequences closest to the query from each run (a combined total of 100,000 sequences), with acyclic structures filtered out. The remaining sequences were manually curated based on their distance to the query, number of peptoid building blocks, and macrocycle ring size to select candidates for synthesis.

2. List of building blocks

Aliphatic side chains

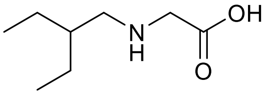

H1

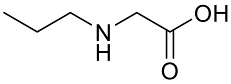

H2

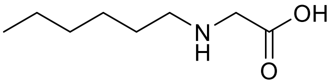

H3

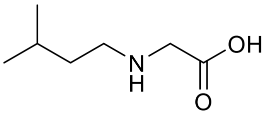

H4

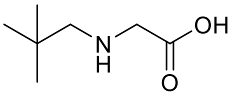

H5

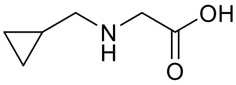

H6

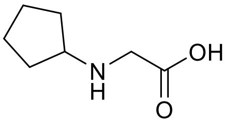

H7

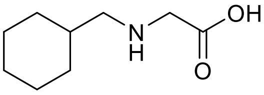

H8

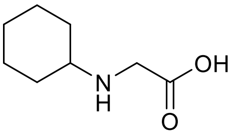

H9

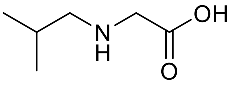

H10

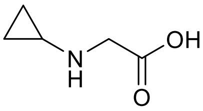

H11

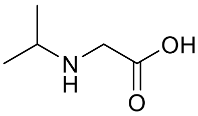

H12

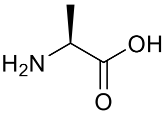

Ala

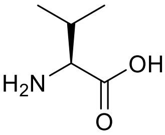

Val

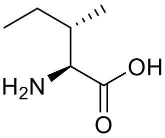

Ile

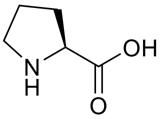

Pro

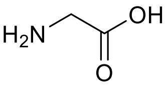

Gly

Polar side chains

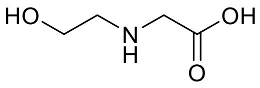

P1

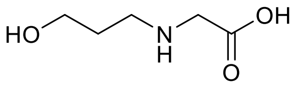

P2

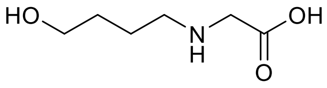

P3

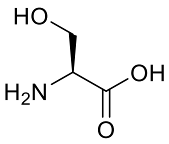

Ser

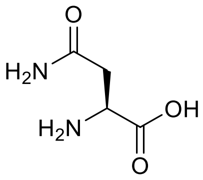

Asn

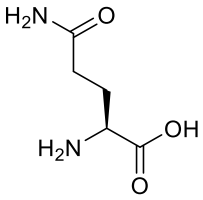

Gln

Cationic side chains

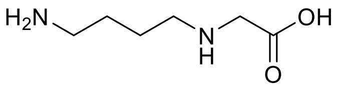

C1

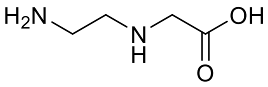

C2

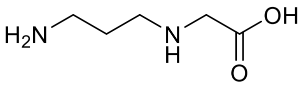

C3

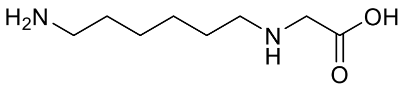

C4

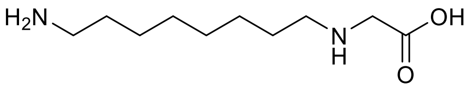

C5

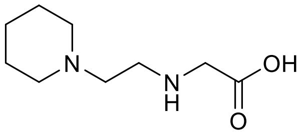

C6

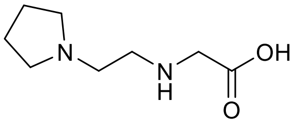

C7

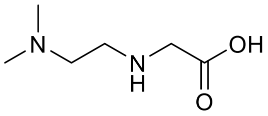

C8

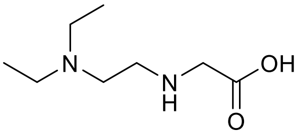

C9

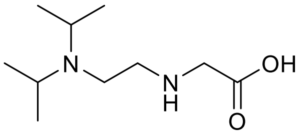

C10

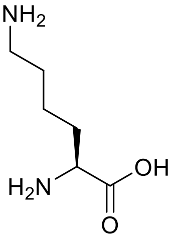

Lys

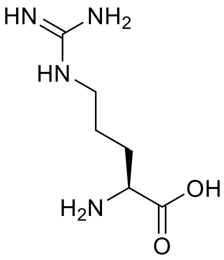

Arg

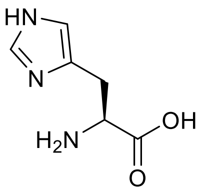

His

Aromatic side chains

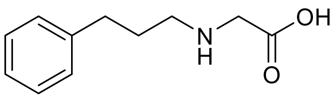

A1

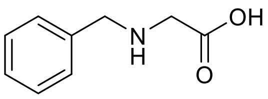

A2

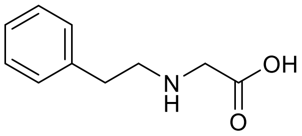

A3

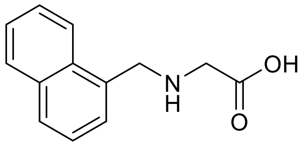

A4

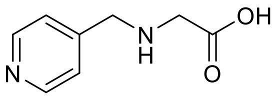

A5

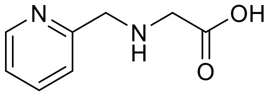

A6

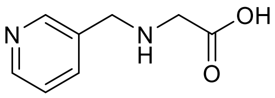

A7

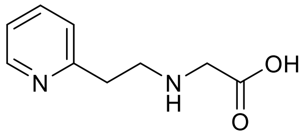

A8

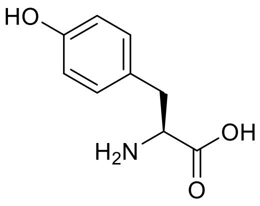

Tyr

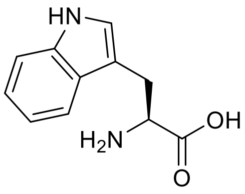

Trp

Anionic side chains

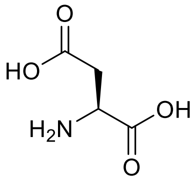

Glu

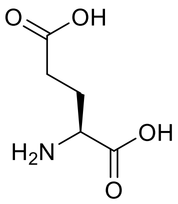

Asp

Fatty acids

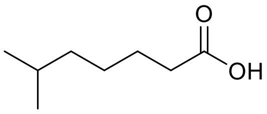

FA0

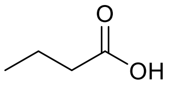

FA1

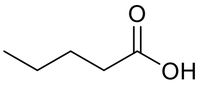

FA2

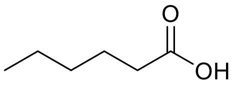

FA3

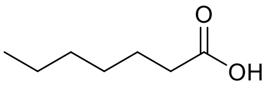

FA4

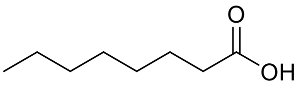

FA5

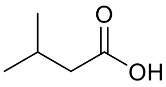

FA6

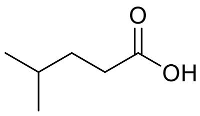

FA7

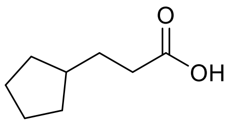

FA8

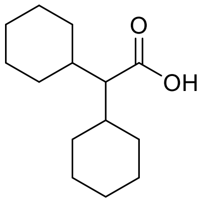

FA9

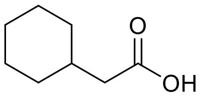

FA10

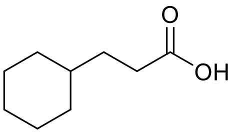

FA11

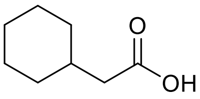

FA12

Figure S1. Structures of the fatty acid and peptoid building blocks used by the PDGA.

3. PDGA Results

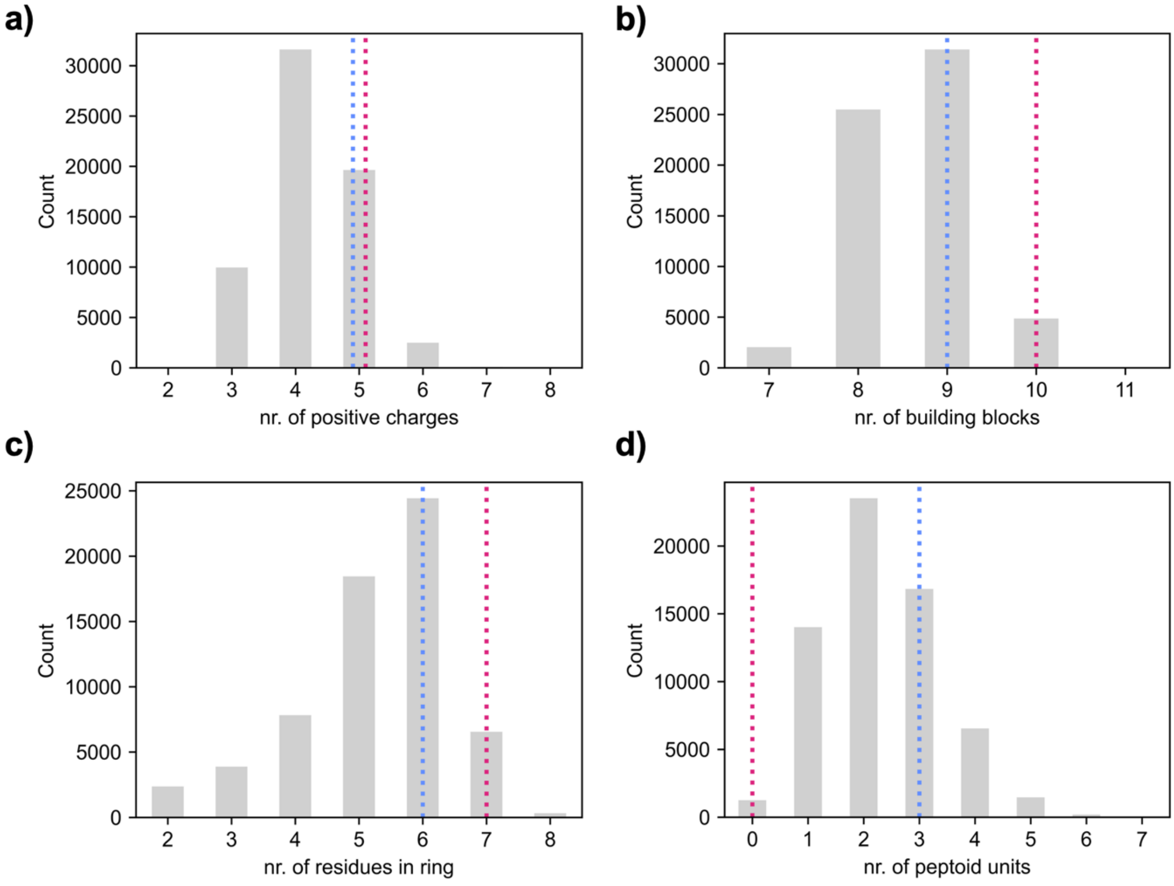

Figure S2. The distribution of a) the number of positive charges, b) the total number of building blocks, c) the number of residues in the macrocyclic ring, and d) the number of peptoid units in the sequence is shown for the highest-scoring macrocycles from the combined PDGA runs (63,815 structures). The blue dotted line represents the corresponding value for EB12, while the red dotted line indicates the value for polymyxin B2.

4. Preparation of the Fmoc-L-Lys-OAll

Fmoc-L-Lys(Boc)-OAll

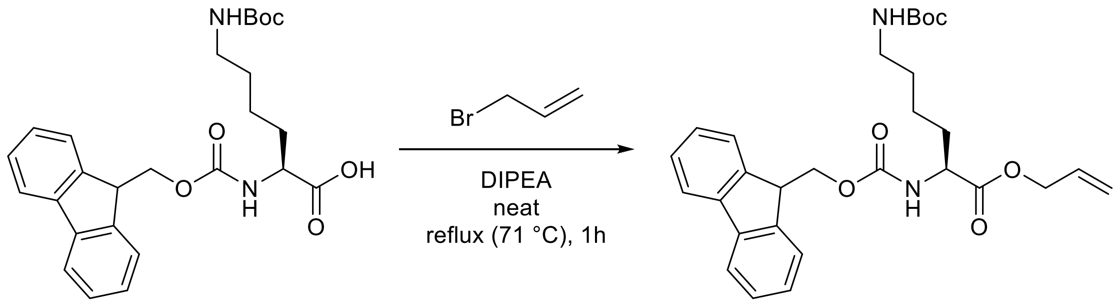

Fmoc-L-Lys(Boc)-OH (15.3 g, 32 mmol, 1 equiv.) was added to a mixture of allyl bromide (80 mL, 480 mmol, 15 equiv.) and DIPEA (7 mL, 64 mmol, 2 equiv.) and the solution was stirred at reflux, for 1 h. The solution was cooled down to RT and diluted with EtOAc. The solution was then washed with 0.1 % HCl, then brine, and the organic layer was separated and concentrated under reduced pressure. The crude was used without purification in the next step.

^{1}H-NMR (300 MHz, DMSO): δ = 7.89 (d, J = 7.5 Hz, 2H), 7.78 (d, J = 7.8 Hz, 1H), 7.71 (d, J = 7.4 Hz, 2H), 7.47 – 7.27 (m, 6H), 6.77 (t, J = 5.8 Hz, 1H), 5.89 (ddt, J = 17.3, 10.5, 5.3 Hz, 1H), 5.37 – 5.14 (m, 2H), 4.57 (m, 2H), 4.37 – 4.17 (m, 4H), 2.88 (t, J = 6.5 Hz, 2H), 1.79 – 1.52 (m, 2H), 1.36 (s, 13H).

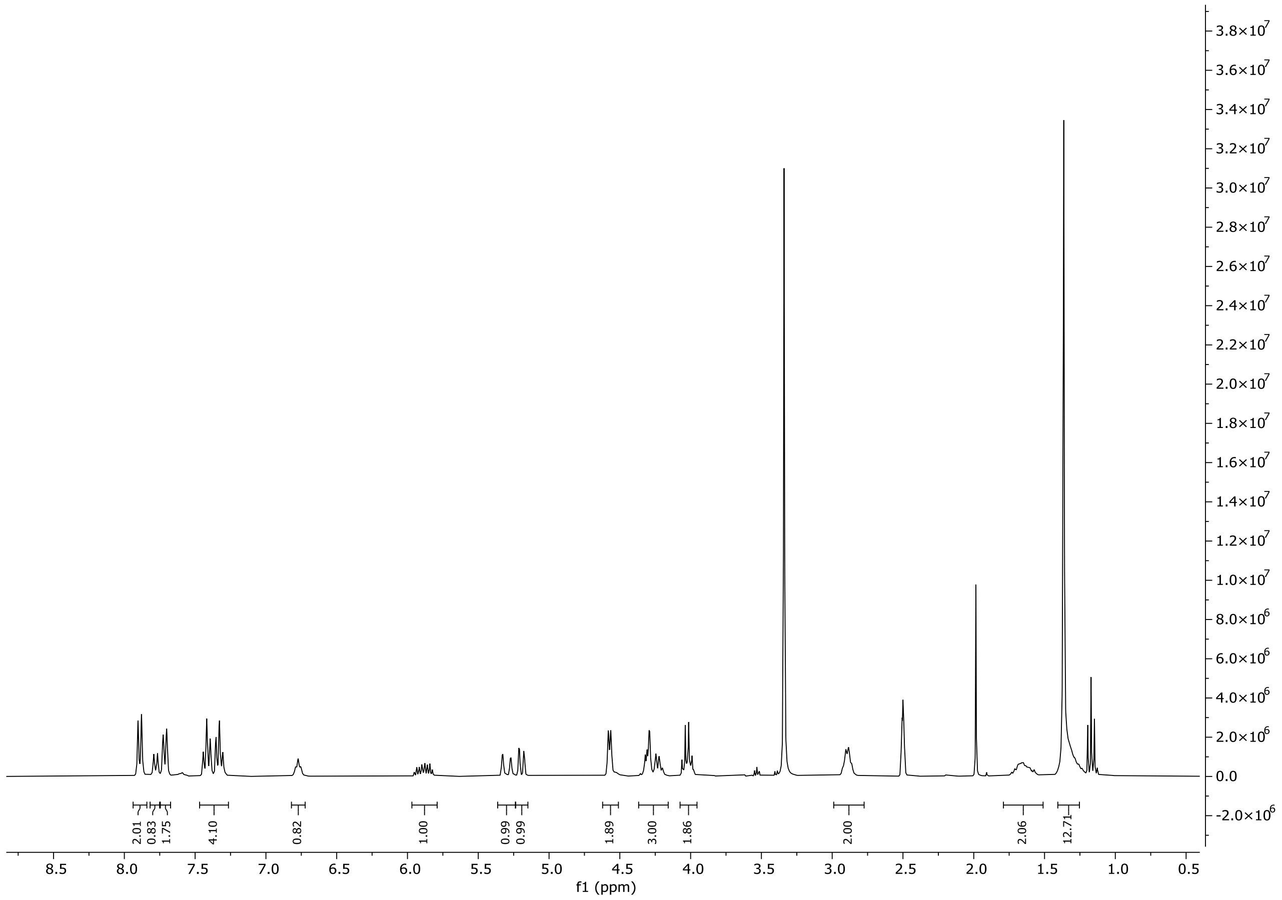

3.8×10^7
3.6×10^7
3.4×10^7
3.2×10^7
3.0×10^7
2.8×10^7
2.6×10^7
2.4×10^7
2.2×10^7
2.0×10^7
1.8×10^7
1.6×10^7
1.4×10^7
1.2×10^7
1.0×10^7
8.0×10^6
6.0×10^6
4.0×10^6
2.0×10^6
0.0
-2.0×10^6
2.01
0.83
1.75
4.10
0.82
1.00
0.99
0.99
1.89
3.00
1.86
2.00
2.06
12.71
8.5
8.0
7.5
7.0
6.5
6.0
5.5
5.0
4.5
4.0
3.5
3.0
2.5
2.0
1.5
1.0
0.5
f1 (ppm)

Fmoc-L-Lys-OAll

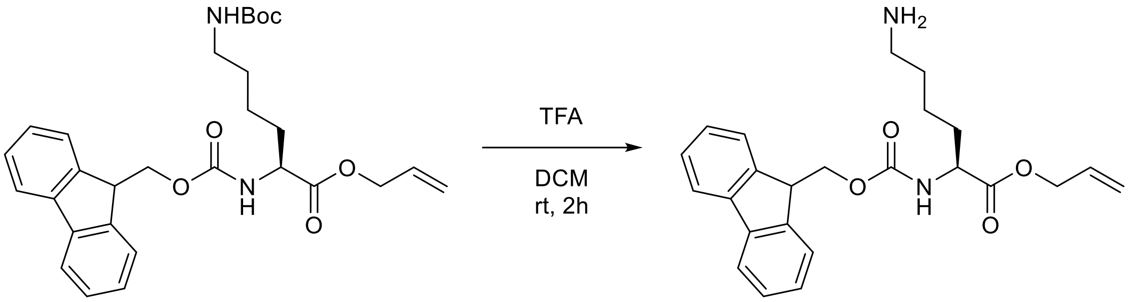

Fmoc-L-Lys(Boc)-OAll (16.3 g, 32 mmol, 1 equiv., crude) was dissolved in DCM (15 mL) and TFA (15 mL). The mixture was stirred at RT for 2h. The solvent was then evaporated under reduced pressure. The crude was then purified by flash column chromatography (DCM:MeOH 9:1). The final product was obtained as a yellowish powder in quantitative yield over two steps.

^{1}H-NMR (300 MHz, DMSO): δ = 7.90 (d, *J* = 7.5 Hz, 4H), 7.81 (d, *J* = 7.8 Hz, 1H), 7.71 (dd, *J* = 7.5, 2.8 Hz, 2H), 7.48 – 7.27 (m, 4H), 5.99 – 5.80 (m, 1H), 5.37 – 5.14 (m, 2H), 4.58 (dt, *J* = 5.4, 1.5 Hz, 2H), 4.41 – 4.17 (m, 3H), 2.81 – 2.66 (m, 2H), 1.77 – 1.61 (m, 2H), 1.61 – 1.46 (m, 1H), 1.44 – 1.22 (m, 2H). **^{13}C-NMR** (75 MHz, DMSO): δ = 172.48, 156.66, 144.27, 144.22, 141.22, 132.87, 128.13, 127.55, 125.68, 120.62, 118.20, 66.10, 65.28, 54.24, 47.11, 30.50, 26.89, 22.91. (1 signal is covered by the solvent signal, ca. 40 ppm).

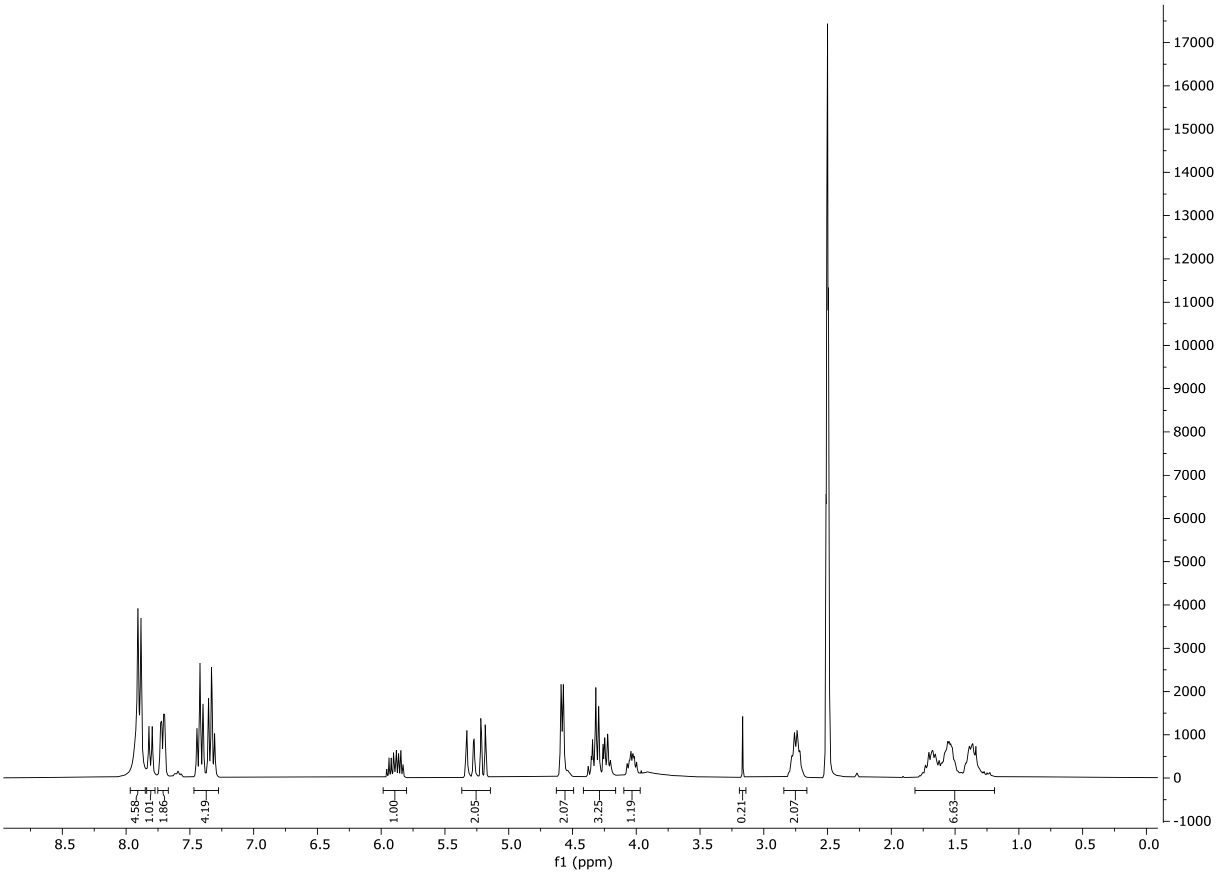
4.58
1.01
1.86
4.19
1.00
2.05
2.07
3.25
1.19
0.21
2.07
6.63
f1 (ppm)

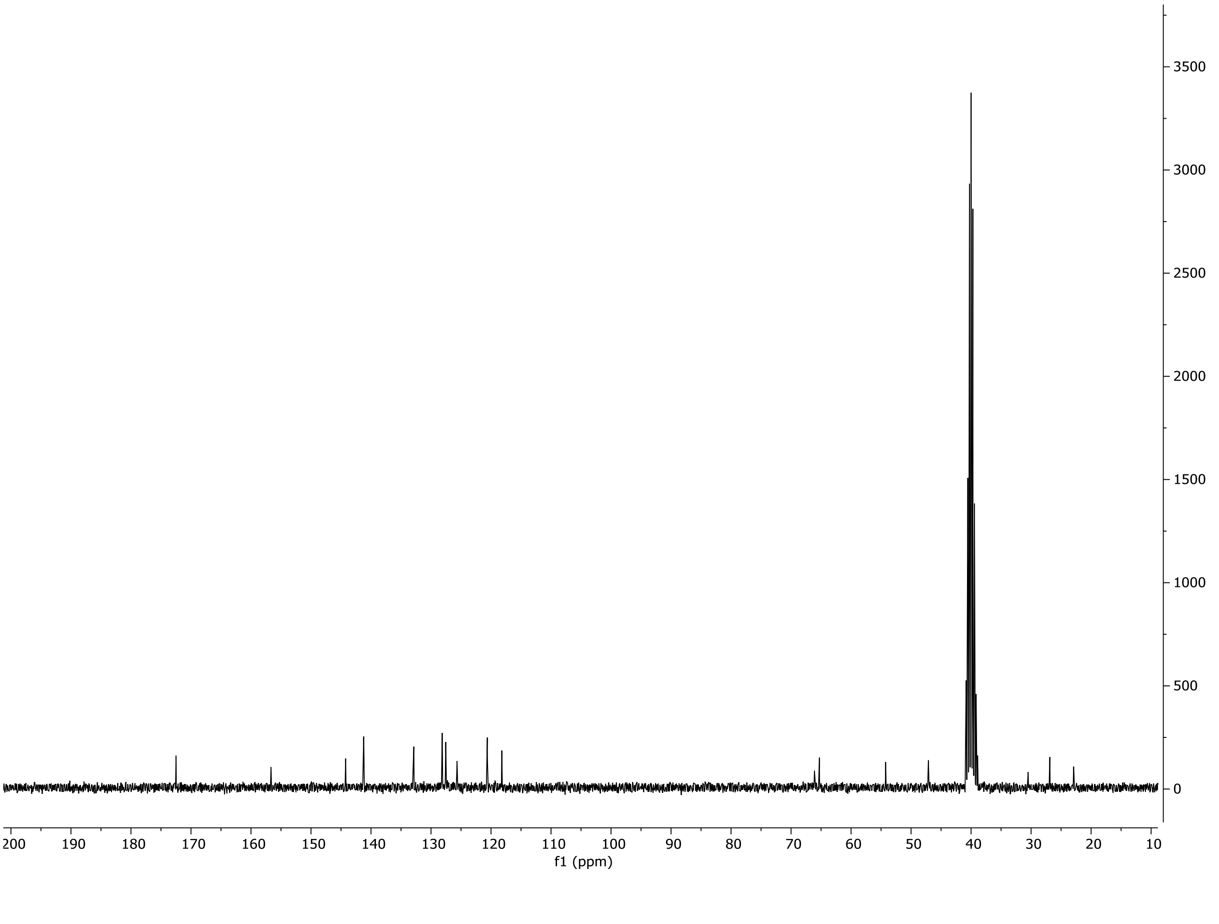
f1 (ppm)

5. Peptide Synthesis

Material and Methods. DMF (N,N-dimethylformamide) was purchased from Thommen-Furler AG, Ethyl 2-cyano-2-(hydroxyimino)acetate (OxymaPure®) was purchased from SENN AG, *N,N'*-diisopropyl carbodiimide (DIC) was purchased from Iris Biotech GMBH, piperazine, 1,8-diazabicyclo[5.4.0]undec-7-ene (DBU), 1-butanol, *N,N*-diisopropylamine (DIPEA), triisopropylsilane (TIS), bromoacetic acid, polymyxin B, tobramycin, 1,4-diaminobutane and isobutylamine were purchased from Sigma Aldrich, phenylsilane was purchased from TCI (Tokyo Chemical Company) and trifluoroacetic acid (TFA) purchased by Fluorochem Ltd. Chemicals were used as supplied and solvents were of analytical grade. The amino acids were purchased from Shanghai Space Peptides Pharmaceuticals Co., Ltd. 2-chlorotrytil resin (2-CTC, 100-200 mesh, 1% DVB, substitution: 1.06 mmol/g) was purchased from Aapptec. The primary amines used for the peptoids were purchased from various suppliers: Sigma Aldrich, TCI, Acros and Fluorochem.

Resin attachment. In a round-bottom flask, 2-chlorotrityl chloride resin (2 g, 1.06 mmol/g, 2.12 mmol) was swelled in dry DCM/DMF (1:1, 20 mL) for 20 min under inert atmosphere, at rt. After that, the amino acid Fmoc-L-Lys-OAll (3.464 g, 4 equiv., 8.48 mmol, *see preparation above*) dissolved in dry DMF (20 mL) with DIPEA (1.0 mL, ca. 10 mmol) was added into the flask and the mixture was stirred overnight at rt. The day after, the resin was transferred in plastic syringes with filter, washed 3x DMF, 3x DCM, 2x MeOH, 2x DMF and 2x DCM. The loading of the resin was then quantified following the procedure described in the literature.[5]

Coupling of Fmoc-amino acids. 5 equiv. of Fmoc-protected amino acid with a concentration of 0.2 M together with 5 equiv. of OxymaPure® and 6 equiv. of DIC, both with a concentration of 0.2 M, were used as coupling reagents in 4.5 mL of DMF. The reaction was stirred for 8 min at 60 °C, washed 2 times with 6 mL DMF and a second coupling step was performed

under the same conditions. The resin was then washed 3 times with 6 mL DMF. After each standard amino acids coupling, the Fmoc protecting group was removed with 2 times 6 mL of a deprotection cocktail containing piperazine (5 %), DBU (2 %) and butanol (10 %) in DMF, respectively for 1 and 4 min at 60 °C. The resin was then washed 4 times with DMF (6 mL).

Coupling of peptoid residues. 13 equiv. of bromoacetic acid, with a concentration of 0.5 M together with 6 equiv. of DIC, with a concentration of 0.2 M were stirred at 60 °C for 8 min in 5 mL DMF. After washing the resin 2 times with 6 mL DMF, 5 equiv. of the corresponding primary amine, with a concentration of 0.2 M was stirred for 8 min at 60 °C. The resin was then washed 3 times with 6 mL DMF. The final cleavage was carried out by treating the resin with 7 mL of a TFA/TIS/H_2O (94:5:1, v/v/v) solution for 3 h at room temperature. The peptide solution was then precipitated with 25 mL of *tert*-butyl methyl ester (TBME), centrifuged for 10 min at 4’000 rpm (twice), evaporated and dried with argon. The dried crude was dissolved in a water/acetonitrile mixture, filtered (pore size 0.45 µm) and purified by preparative RP-HPLC with gradients of 15 min. The pure fraction was analysed by LC-MS with a 5 min gradient. Pure products were obtained as white foamy solids after lyophilisation. The yields were calculated for the TFA salts.

Coupling of the fatty acids. 5 equiv. of the desired acid with a concentration of 0.2 M together with 6 equiv. of DIC (0.2 M) were used as coupling reagents in a total volume of 6 mL of DMF. The reaction was stirred for 8 min at 60 °C, washed 2 times with 6 mL DMF and a second coupling step was performed under the same conditions. The resin was then washed 3 times with 6 mL DMF.

Allyl and/or Alloc deprotection. Directly in the reactor vessel, a catalytic amount of tetrakis(triphenylphosphine)palladium(0) (0.25 equiv.) was diluted in dry DCM and added to the resin. Immediately, phenylsilane (12.5 equiv.) was added. The reaction was performed

directly in the open reactor used for the synthesis, with nitrogen bubbling, at 40 °C for 5 min. Due to evaporation of the DCM, the reactions were constantly refilled with DCM. The deprotection step was repeated three times in total.

Cyclization procedure (on-resin). The peptide was cyclized on the resin by adding Oxyma (2 mL of a 2.5 g/10 mL solution in DMF, ca. 10 equiv.) and DIC (3 mL of a 5 mL/12.5 mL solution in DMF, ca. 15 equiv.) and DIPEA (0.18 mL, ca. 3 equiv.). The plastic syringes were closed with caps and shake overnight at room temperature. The resin beads were washed 3 times with DMF (6 mL each time) and 2 times with MeOH (6 mL).

Global deprotection and resin cleavage. The cleavage was carried out by treating the resin with 7 mL of a TFA/TIS/H2O (94:5:1, v/v/v) solution for 3 h at room temperature. The dendrimer solution was then precipitated with 25 mL of tert-butyl methyl ester (TBME), centrifuged for 10 min at 4’000 rpm (twice), evaporated and dried with argon. The dried crude was dissolved in a water/acetonitrile mixture, filtered (pore size 0.45 µm) and purified by preparative RP-HPLC. Pure products were obtained as white solids after lyophilisation. The yields were calculated for the TFA salts.

LC and MS. Analytical RP-HPLC was performed with an *Ultimate 3000 Rapid Separation* LC–MS System (*DAD-3000RS* diode array detector) using an *Acclaim RSLC 120 C18* column (2.2 µm, 120 Å, 3×50 mm, flow 1.2 mL/min) from *Dionex*. Data recording and processing was done with Dionex Chromeleon Management System Version 6.80 (analytical RP-HPLC). All RP-HPLC were using HPLC-grade acetonitrile and *Milli-Q* deionized water. The elution solutions were *A*) *MilliQ* deionized water containing 0.05 % TFA; *D*) *MilliQ* deionized water/acetonitrile (10 : 90, *v/v*) containing 0.05 % TFA. Preparative RP-HPLC was performed with a *Waters* automatic Prep LC Controller system containing the four following modules: *Waters 515* HPLC pump, *Waters 2489* UV/vis detector, *SQ Detector*

2 (single quadrupole mass detector and *Waters 2767* Sample Manager, Injector and Collector. A *Dr. Maisch GmbH Reprospher* column (*C18-DE*, 100×30 mm, particle size 5 µm, pore size 100 Å, flow rate 40 mL/min) was used. Compounds were collected according to their mass using SQ Detector 2. Data recording and processing was performed with MassLynx software version 4.2. The elution solutions were: *A*) *MilliQ* deionized water containing 0.05 % TFA; *D*) Acetonitrile containing 0.05 % TFA. Method used was a gradient from 2 % to 50 % of solvent *D* in 14 min with a total runtime of 20 min. MS Spectra, recorded on a *Thermo Scientific LTQ OrbitrapXL*, were provided by the MS analytical service of the Department of Chemistry, Biochemistry and Pharmaceutical Sciences at the University of Bern (group PD Dr. *Stefan Schürch*).

NMR Spectra (^{1}H, ^{13}C). NMR Spectra were recorded at 20 °C. Chemical shifts (δ) are reported in ppm relative to the signal of tetramethylsilane (TMS) and residual solvent signals in ^{1}H- and ^{13}C-NMR spectra were used as internal reference. ^{1}H- and ^{13}C-NMR spectra were measured on a *Bruker Avance III 300* spectrometer (at 300 MHz and 75 MHz, resp.).

6. Compounds Sequences and Analysis

Table S1. Sequences synthesized and yield

Cpd	Sequences [a]	SPPS Yield [b] mg (%)	MS Analysis [c] calc./obs. (g/mol)
**PMB2**	FA0-Dab-Thr-Dab-Dab(*)-Dab-dPhe-Leu-Dab-Dab-Thr-*	40 (9%)	1188.7343/1188.7338
**EB12**	FA5-A3-Lys-Asn-Dab(*)-Lys-H2-Lys-C3-Lys-*	77 (22%)	1226.8227/1226.8207
**EB13**	FA4-Val-C2-A1-Dab(*)-Lys-Gly-Lys-C3-Lys-*	63 (19%)	1141.7699/1141.7690
**EB14**	FA5-C2-Tyr-Lys-Dab(*)-Lys-Arg-C2-Lys-*	29 (10%)	1129.7448/1129.7439
**EB15**	FA11-A2-Lys-Asn-Dab(*)-C2-Arg-C9-Lys-*	59 (22%)	1167.7604/1167.7646
**EB16**	FA5-A6-C2-Gln-Dab(*)-Lys-C1-Lys-Lys-*	24 (11%)	1114.7339/1114.7364
**EB17**	FA11-A2-Lys-Asn-Dab(*)-Lys-C3-Lys-C8-Lys-*	10 (4%)	1253.8336/1253.8312
**EB18**	FA10-A3-Lys-C2-Dab(*)-Asn-C2-Lys-Val-Lys-*	64 (22%)	1182.7601/1182.7606
**EB19**	FA2-A3-C2-Asn-Arg-Dab(*)-Lys-C1-Lys-Lys-*	32 (10%)	1227.7928/1227.7967
**EB20**	FA12-A3-Lys-C2-Dab(*)-Asn-C2-Lys-Val-Lys-*	94 (28%)	1168.7444/1168.7455
**EB21**	FA5-Lys-A2-Lys-Dab(*)-Asn-Lys-C2-H7-Lys-*	39 (11%)	1224.8070/1224.8059
**EB22**	FA2-A2-Lys-H3-Dab(*)-Lys-Asn-Lys-C2-C2-Lys-*	85 (22%)	1298.8551/1298.8549
**EB23**	FA4-A2-C3-C2-Dab(*)-Arg-Gln-C2-H9-Lys-*	10 (3%)	1224.7819/1224.7826
**EB24**	FA5-C3-Val-A6-Dab(*)-C1-Lys-Lys-P3-Lys-*	38 (16%)	1228.8383/1228.8360
**EB25**	FA10-A2-C1-C2-Dab(*)-Lys-Asn-Lys-Gly-Lys-*	11 (5%)	1154.7288/1154.7265
**EB26**	FA7-A3-Gly-Arg-Dab(*)-H2-Asn-Lys-C3-Lys-*	14 (7%)	1155.7240/1175.7219
**EB27**	FA2-A2-Arg-Asn-Dab(*)-Ser-C2-Lys-Arg-Lys-*	90 (39%)	1200.7204/1200.7193
**EB28**	FA11-A7-Lys-Asn-Dab(*)-H2-Lys-C3-C2-Lys-*	23 (10%)	1197.7710/1197.7696
**EB29**	FA2-A2-Lys-Ser-Dab(*)-C2-H10-Lys-H6-C2-Lys-*	14 (4%)	1226.7863/1226.7845
**EB30**	FA2-A3-C1-Asn-Lys-Dab(*)-Asn-Lys-C2-Lys-*	50 (22%)	1185.7346/1185.7326
**EB31**	FA1-A3-Ser-C2-Dab(*)-Ser-Lys-H6-Lys-Lys-Lys-*	14 (6%)	1228.7656/1228.7634
**EB32**	FA8-A2-Lys-Lys-Asn-Dab(*)-C3-Pro-P1-Lys-*	61 (23%)	1181.7285/1181.7324
**EB33**	FA7-H4-C3-A6-Dab(*)-C2-Lys-Ser-Lys-C1-Lys-*	19 (6%)	1286.8551/1286.8593
**EB34**	FA5-A7-C3-Gln-Dab(*)-C1-Gln-Lys-Lys-*	46 (15%)	1128.7131/1128.7133
**EB35**	FA2-A2-C1-Asn-Lys-Dab(*)-Ser-Lys-C1-Lys-*	100 (31%)	1172.7394/1172.7389
**EB36**	FA11-A2-H12-Asn-Dab(*)-Ser-Lys-C1-Asn-Lys-*	72 (24%)	1183.7077/1183.7083
**EB37**	FA11-A3-Pro-Lys-Dab(*)-Asn-Gly-H12-Lys-Ser-Lys-*	62 (20%)	1237.7547/1237.7549
**EB38**	FA4-A8-Lys-Asn-Dab(*)-Asn-C2-Lys-Pro-Lys-*	78 (24%)	1183.7190/1183.7191

**EB39**	FA8-A8-Lys-Asn-Dab(*)-Asn-H2-Lys-C2-Lys-*	78 (29 %)	1197.7346/1197.7384
**EB40**	FA2-A2-Lys-Asn-Dab(*)-C2-Ser-Ser-H7-C1-Lys-*	28 (9%)	1228.7292/1228.7279
**EB41**	FA3-A6-Lys-Asn-Dab(*)-C2-C2-C1-Lys-Lys-*	187 (52%)	1172.7506/1172.7511
**EB42**	FA6-C2-Ala-Tyr-Arg-Dab(*)-Ser-C2-C2-Lys-*	55 (20%)	1089.6407/1089.6415
**EB43**	FA2-Lys-A5-Lys-Dab(*)-C2-Asn-Arg-H12-Lys-*	46 (16%)	1185.7458/1185.7498
**EB44**	FA3-C3-Gly-A2-Dab(*)-Lys-Lys-P1-Asn-Lys-*	32 (15%)	1116.6815/1116.6929
**EB45**	FA3-A3-Lys-Asn-Dab(*)-C3-Asn-Ala-H10-Lys-*	42 (17%)	1141.6972/1141.7009
**EB46**	FA10-Lys-Tyr-Lys-Dab(*)-Gln-Asn-Lys-Lys-*	30 (10%)	1141.6972/1141.6969
**EB47**	FA4-Tyr-Lys-Gln-Dab(*)-Val-Ile-Lys-Ser-Lys-*	24 (8%)	1186.7438/1186.7438
**EB48**	FA4-Lys-Tyr-Lys-Dab(*)-Lys-Gln-Lys-Lys-*	24 (7%)	1143.7492/1143.7489
**EB49**	FA5-Tyr-Gln-Lys-Dab(*)-Gln-Gln-Lys-Lys-*	35 (13%)	1157.6921/1157.6920
**EB50**	FA7-Lys-Lys-Gln-Lys-Dab(*)-Asn-Tyr-Lys-*	22 (7%)	1115.6815/1115.6818
**EB51**	FA3-A8-Val-Lys-Asn-Dab(*)-C2-P1-Lys-Lys-*	17 (8%)	1158.7237/1158.7227
**EB52**	FA12-A8-Lys-Asn-Dab(*)-Asn-H2-Lys-C2-Lys-*	12 (6%)	1183.7190/1183.7167

[a] Three-letter codes for amino acids. All amino are in L- configuration, with the exception of D-Phe in PMB. The peptoid and fatty acid building blocks are denoted with a specific code according to their side chain residues: FA = fatty acid, H = aliphatic, P = polar, C = cationic and A = aromatic. A detailed list of the building blocks can be found in the *Supporting Information* (**Figure S1**). The "*" represent the position of the ring closure. [b] Yield given for RP-HPLC purified product. [c] Electrospray ionization mass spectrometry (positive mode), the calculated monoisotropic masses, and the observed masses for [M] are reported.

Table S2. Characteristics of the peptide-peptoid macrocycles

Cpd	Sequences[a]	MIC[b] on *E. coli* (µg/mL)	Total positive charges	Size of the macrocycle[c]	Number of peptoids[d]	Number of peptides[e]	Heavy atom count[f]	Hydrophobic count[g]
**PMB2[h]**	FA0-Dab-Thr-Dab-Dab(*)-Dab-dPhe-Leu-Dab-Dab-Thr-*	0.5	5	7	0	10	84	55
**EB12**	FA5-A3-Lys-Asn-Dab(*)-Lys-H2-Lys-C3-Lys-*	0.5 - 1	5	6	3	6	87	60
**EB13**	FA4-Val-C2-A1-Dab(*)-Lys-Gly-Lys-C3-Lys-*	0.5	5	6	3	5	81	56
**EB14**	FA5-C2-Tyr-Lys-Dab(*)-Lys-Arg-C2-Lys-*	0.5	6	5	2	6	80	53
**EB15**	FA11-A2-Lys-Asn-Dab(*)-C2-Arg-C9-Lys-*	0.5	5	5	3	5	83	56
**EB16**	FA5-A6-C2-Gln-Dab(*)-Lys-C1-Lys-Lys-*	0.5	5	5	3	5	79	54
**EB17**	FA11-A2-Lys-Asn-Dab(*)-Lys-C3-Lys-C8-Lys-*	0.5	6	6	3	6	89	61
**EB18**	FA10-A3-Lys-C2-Dab(*)-Asn-C2-Lys-Val-Lys-*	0.5	5	6	3	6	84	57
**EB19**	FA2-A3-C2-Asn-Arg-Dab(*)-Lys-C1-Lys-Lys-*	0.5	6	5	3	6	87	57
**EB20**	FA12-A3-Lys-C2-Dab(*)-Asn-C2-Lys-Val-Lys-*	0.5 - 1	5	6	3	6	83	56
**EB21**	FA5-Lys-A2-Lys-Dab(*)-Asn-Lys-C2-H7-Lys-*	1	5	6	3	6	87	60
**EB22**	FA2-A2-Lys-H3-Dab(*)-Lys-Asn-Lys-C2-C2-Lys-*	1 - 2	6	7	4	6	92	62
**EB23**	FA4-A2-C3-C2-Dab(*)-Arg-Gln-C2-H9-Lys-*	2	4	6	5	4	87	58
**EB24**	FA5-C3-Val-A6-Dab(*)-C1-Lys-Lys-P3-Lys-*	2	5	6	4	5	87	61
**EB25**	FA10-A2-C1-C2-Dab(*)-Lys-Asn-Lys-Gly-Lys-*	2	5	6	3	6	82	55
**EB26**	FA7-A3-Gly-Arg-Dab(*)-H2-Asn-Lys-C3-Lys-*	2	4	6	3	6	82	54
**EB27**	FA2-A2-Arg-Asn-Dab(*)-Ser-C2-Lys-Arg-Lys-*	2	5	6	2	7	85	53
**EB28**	FA11-A7-Lys-Asn-Dab(*)-H2-Lys-C3-C2-Lys-*	2	5	6	4	5	85	58
**EB29**	FA2-A2-Lys-Ser-Dab(*)-C2-H10-Lys-H6-C2-Lys-*	4	5	7	5	5	87	59
**EB30**	FA2-A3-C1-Asn-Lys-Dab(*)-Asn-Lys-C2-Lys-*	4	5	5	3	6	84	55
**EB31**	FA1-A3-Ser-C2-Dab(*)-Ser-Lys-H6-Lys-Lys-Lys-*	4	5	7	3	7	87	58
**EB32**	FA8-A2-Lys-Lys-Asn-Dab(*)-C3-Pro-P1-Lys-*	4 - 8	4	5	4	5	84	57
**EB33**	FA7-H4-C3-A6-Dab(*)-C2-Lys-Ser-Lys-C1-Lys-*	8	6	6	5	5	91	62
**EB34**	FA5-A7-C3-Gln-Dab(*)-C1-Gln-Lys-Lys-*	8	4	5	3	5	80	54
**EB35**	FA2-A2-C1-Asn-Lys-Dab(*)-Ser-Lys-C1-Lys-*	8 - 16	5	5	3	6	83	55
**EB36**	FA11-A2-H12-Asn-Dab(*)-Ser-Lys-C1-Asn-Lys-*	16	3	6	3	6	84	56
**EB37**	FA11-A3-Pro-Lys-Dab(*)-Asn-Gly-H12-Lys-Ser-Lys-*	16	3	7	3	7	88	60
**EB38**	FA4-A8-Lys-Asn-Dab(*)-Asn-C2-Lys-Pro-Lys-*	16	4	6	3	6	84	56
**EB39**	FA8-A8-Lys-Asn-Dab(*)-Asn-H2-Lys-C2-Lys-*	16	4	6	3	6	85	57
**EB40**	FA2-A2-Lys-Asn-Dab(*)-C2-Ser-Ser-H7-C1-Lys-*	16	4	7	4	6	87	57
**EB41**	FA3-A6-Lys-Asn-Dab(*)-C2-C2-C1-Lys-Lys-*	16 - 32	6	6	4	5	83	55
**EB42**	FA6-C2-Ala-Tyr-Arg-Dab(*)-Ser-C2-C2-Lys-*	16 - 32	5	5	3	6	77	48

**EB43**	FA2-Lys-A5-Lys-Dab(*)-C2-Asn-Arg-H12-Lys-*	32	5	6	3	6	84	55
**EB44**	FA3-C3-Gly-A2-Dab(*)-Lys-Lys-P1-Asn-Lys-*	32	4	6	3	6	79	52
**EB45**	FA3-A3-Lys-Asn-Dab(*)-C3-Asn-Ala-H10-Lys-*	32	3	6	3	6	81	54
**EB46**	FA10-Lys-Tyr-Lys-Dab(*)-Gln-Asn-Lys-Lys-*	32	4	5	0	8	81	54
**EB47**	FA4-Tyr-Lys-Gln-Dab(*)-Val-Ile-Lys-Ser-Lys-*	32 - >32	3	6	0	9	84	57
**EB48**	FA4-Lys-Tyr-Lys-Dab(*)-Lys-Gln-Lys-Lys-*	>32	5	5	0	8	81	55
**EB49**	FA5-Tyr-Gln-Lys-Dab(*)-Gln-Gln-Lys-Lys-*	>32	3	5	0	8	82	54
**EB50**	FA7-Lys-Lys-Gln-Lys-Dab(*)-Asn-Tyr-Lys-*	>32	4	4	0	8	79	52
**EB51**	FA3-A8-Val-Lys-Asn-Dab(*)-C2-P1-Lys-Lys-*	>32	4	5	3	6	82	55
**EB52**	FA12-A8-Lys-Asn-Dab(*)-Asn-H2-Lys-C2-Lys-*	>32	4	6	3	6	84	56

[a] Three-letter codes for amino acids. All amino are in L- configuration, with the exception of D-Phe in PMB. The peptoid and fatty acid building blocks are denoted with a specific code according to their side chain residues: FA = fatty acid, H = aliphatic, P = polar, C = cationic and A = aromatic. A detailed list of the building blocks can be found in the *Supporting Information* (**Figure S1**). The "*" represent the position of the ring closure. [b] Minimum inhibitory concentration (MIC), in µg/mL, determined on *E. coli* W3110 in diluted Mueller-Hinton (MH 12.5%) broth at pH 7.4, after incubation for 16-20 hours at 37 °C. Values represent two different duplicate MIC determinations. [c] Total number of building block (amino acids or peptoid units) involved in the macrocycle. [d] Number of peptoid units within the molecule. Prolines were considered as peptoid units, as they lack the -NH bond donating group on the α-nitrogen atom (tertiary amide). [e] Total number of amino acids (except Pro) within the molecule. [f] Number of atoms, excluding hydrogens. [g] Number of aliphatic carbons, aromatic atoms heavy atoms and sulfur atoms. [h] Polymyxin B2.

7. Compounds Activities

Minimal Inhibitory Concentration (MIC)

Antimicrobial activity was assayed for all the peptide-peptoids against *P. aeruginosa* PAO1, *K. pneumoniae* NCTC418, *E. coli* W3110, *A. baumannii* ATCC 19606, methicillin-resistant *S. aureus* COL (MRSA) and for selected peptides on *P. aeruginosa* PA14, as well as the clinical isolates ZEM-1A and ZEM9A, *K. pneumoniae* OXA-48, *Enterobacter cloacae*, *Stenotrophomonas maltophilia* and *Staphylococus epidermidis*. To determine the Minimal Inhibitory Concentration (MIC), Broth Microdilution method was used.[6] A colony of bacteria was picked and grown in LB medium overnight at 37 °C. The next day, the culture was then regrown in LB medium to log phase (OD_{600} = 0.6 to 0.8), which lasted approximately 4 hours, and diluted to an OD_{600} of 0.022 in the desired medium (full MH or 12.5% MH).

The compounds were prepared as stock solutions of 2 mg/mL in sterilized MilliQ deionized water and then diluted in the desired media (full MH or 12.5% MH) to reach the first concentration tested (32 µg/mL). The compounds were added to the first well of a 96-well sterile, round bottom microtiter plates in polystyrene (TPP®, untreated) and diluted serially by ½ in the desired media (full MH or 12.5% MH), to a final volume of 150 µL/well. To each well was finally added 4 µL of the diluted bacterial suspension (see above), corresponding to approximately 5×10^5 CFU. For each test, two columns of the plate were kept for sterility control (medium only), growth control (medium with bacterial inoculum, no compound). The positive control, Polymyxin B (starting with a concentration of 16 µg/mL) in MH medium with bacterial inoculums, was introduced in the two first lines of the plate. The plates were incubated at 37 °C for ca. 18 hours under static conditions. 15 µL of 3-(4,5-dimethylthiazol-2-yl)-2,5-diphenyltetrazolium bromide (MTT)[7] (1 mg/mL in sterilized MilliQ deionized water) were added to each well and the plates were incubated for 20-30 minutes at room temperature. The

minimal inhibitory concentration (MIC) was defined as the lowest concentration of the compound that inhibits the visible growth of the tested bacteria (yellow) with the unaided eye.

Minimal Hemolytic Concentration (MHC)

To determine the minimal hemolytic concentration (MHC), stock solutions of 8 mg/mL of the peptide in sterilized MilliQ water deionized were prepared and 50 µL were diluted serially by ½ in 50 µL PBS (pH 7.4) in 96-well plate (Costar or Nunc, polystyrene, untreated). Human red blood cells (hRBC) were obtained by centrifugation of 1.5 mL of whole blood, from the blood bank of Bern, at 3000 rpm for 15 minutes at 4 °C. Plasma was discarded and the pellet was re-suspended in a 15 mL Falcon tube in 5 mL of PBS. The washing was repeated three times, and the remaining pellet was re-suspended in 10 mL of PBS at a final hRBC concentration of 5%. The hRBC suspension (50 µL) was added to each well and the plate was incubated at room temperature for 4 hours. Minimal hemolytic concentration (MHC) end points were determined by visual determination of the wells after the incubation period. Controls on each plate included a blank medium control (50 µL PBS + 50 µL of hRBC suspension) and a hemolytic activity control t-octylphenoxypolyethoxyethanol (Triton-X100) (1 % in MilliQ-deionized water) 50 µL + 50 µL hRBC suspension).

Table S3. Antimicrobial activities of peptide-peptoid macrocycles

**Cpd**			**MIC[a] (µg/mL)**			**MHC[b] (µg/mL)**
	E. coli W3110	***P. aeruginosa*** PAO1	***A. baumannii*** ATCC19606	***K. pneumoniae*** NCTC418	***S. aureus*** COL (MRSA)	
**EB9[c]**	2 / 2	4 / 2	16 / 8	>32 / 16	32 / 4 - 8	>2000
**PMB2[d]**	0.5 / 1	1 / 1	1 / 1	32 / 2	32 / 2	125
**EB12**	0.5 - 1 / 0.5	2 / 2 - 4	8 / 4	32 / 32	16 / 1 - 2	500
**EB13**	0.5 / 0.25 - 0.5	4 / 4	8 / 4	32 - >32 / 32	8 / 2	2000
**EB14**	0.5 / 1	4 / 4	32 / 32	>32 / >32	16 / 1 - 2	>2000
**EB15**	0.5 / 1	8 / 8	16 / 16	>32 / 32	4 / 1	1000
**EB16**	0.5 / 0.5	8 / 4	16 - 32 / 16	>32 / >32	32 / 2	>2000
**EB17**	0.5 / 1	8 / 4	8 / 4	>32 / 32	8 / 1	500
**EB18**	0.5 / 1	8 / 8	16 / 8	>32 / >32	8 - 16 / 2	>2000

**EB19**	0.5 / 0.5 - 1	8 / 4	32 / 16	>32 / >32	16 / 1 - 2	>2000
**EB20**	0.5 - 1 / 0.5	8 / 8	16 / 8	>32 / >32	>32 / 8	>2000
**EB21**	1 / 0.5	4 / 4	8 / 4	>32 / >32	32 / 4	>2000
**EB22**	1 - 2 / 1	8 / 4	16 / 8 - 16	>32 / >32	16 / 4	>2000
**EB23**	2 / 1 - 2	8 / 8	16 / 8	>32 / >32	16 / 4 - 8	1000
**EB24**	2 / 0.5	8 / 4	16 / 8	>32 / 32	32 / 4	>2000
**EB25**	2 / 1	16 / 8	32 / 8	>32 / >32	32 / 4	500
**EB26**	2 / 2	16 / 8 - 16	16 / 8	>32 / >32	32 / 4	>2000
**EB27**	2 / 2	16 / 16	>32 / 32	>32 / >32	32 / 2 - 4	>2000
**EB28**	2 / 2 - 4	32 / 16	32 / 32	>32 / >32	32 / 4 - 8	>2000
**EB29**	4 / 2	8 / 8	16 / 16	>32 / >32	32 / 8	>2000
**EB30**	4 / 2	16 / 16	32 / 32	>32 / >32	>32 / 8	>2000
**EB31**	4 / 1	16 / 16	32 / 16	>32 / >32	>32 / 8	>2000
**EB32**	4 - 8 / 2 - 4	16 / 16	16 / 16	>32 / >32	>32 / 8	>2000
**EB33**	8 / 4	8 / 8	32 / 16 - 32	>32 / >32	8 - 16 / 2	31.25 - 62.5
**EB34**	8 / 4	16 / 16	32 / 16	>32 / >32	>32 / 32	>2000
**EB35**	8 - 16 / 2 - 4	16 / 16	32 / 16	>32 / >32	>32 / 8	>2000
**EB36**	16 / 8 - 16	16 / 16	16 / 8 - 16	>32 / >32	>32 / 16	500
**EB37**	16 / 16	16 / 16	16 / 16	>32 / >32	>32 / 16	500
**EB38**	16 / 4 - 8	32 / 16	32 / 16	>32 / >32	>32 / 16	2000
**EB39**	16 / 16	32 / 32	>32 / >32	>32 / >32	>32 / 16	>2000
**EB40**	16 / 4	32 / 16	32 / 32	>32 / >32	>32 / 16 - 32	1000
**EB41**	16 - 32 / 4 - 8	16 / 8	32 / 16	>32 / >32	32 / 8	500 - 1000
**EB42**	16 - 32 / 4 - 8	32 / 32	>32 / >32	>32 / >32	32 / 4 - 8	>2000
**EB43**	32 / 4 - 8	>32 / >32	>32 / >32	>32 / >32	>32 / 16 - 32	>2000
**EB44**	32 / 32	>32 / >32	>32 / >32	>32 / >32	>32 / 32	>2000
**EB45**	32 / 32	>32 / >32	32 / 32	>32 / >32	>32 / 32	>2000
**EB46**	32 / 16	>32 / >32	>32 / >32	>32 / >32	>32 / 32 - >32	>1000
**EB47**	32 - >32 / 16	>32 / 32 - >32	>32 / 16 - 32	>32 / >32	>32 / 16 - 32	>1000
**EB48**	>32 / 16 - 32	>32 / 32	>32 / >32	>32 / >32	>32 / 16 - 32	>1000
**EB49**	>32 / >32	>32 / 32 - >32	>32 / >32	>32 / >32	>32 / >32	500
**EB50**	>32 / 32 - >32	>32 / >32	>32 / >32	>32 / >32	>32 / >32	>1000
**EB51**	>32 / >32	>32 / >32	>32 / >32	>32 / >32	>32 / >32	>2000
**EB52**	>32 / 16	>32 / >32	>32 / >32	>32 / >32	>32 / 32	>2000

[a] Minimum inhibitory concentration (MIC), in µg/mL, was determined on bacteria in diluted Mueller-Hinton (MH 12.5%) broth at pH 7.4 / 8.5, after incubation for 16-20 hours at 37 °C. Values represent two different duplicate MIC determinations. [b] Minimum Hemolytic Concentration (MHC) measured on human red blood cells (hRBC) in 10 mM phosphate buffer, 150 mM NaCl, pH 7.4, 25 °C, 4 h. [c] **EB9** is a linear peptide-peptoid hybrids with the sequence K*k*L*l*K*l*L*k*L*l*L, where the italic letters represent the peptoid version of the corresponding single letter amino acid code. This compound has already been reported.[8] [d] Polymyxin B2.

Table S4. Antimicrobial activities of peptide-peptoid macrocycles and their corresponding linear versions

**Cpd**	**MIC[a] (µg/mL)**					**MHC[b] (µg/mL)**
	E. coli W3110	***P. aeruginosa*** PAO1	***S. aureus*** COL (MRSA)	***A. baumannii*** ATCC19606	***K. pneumoniae*** NCTC418	
**PMB2[c]**	0.5 – 1	1	8	1 – 2	0.5 – 1	125
**PMB2lin**	1	4	32	32	8	>1000
**EB12**	0.5 – 1	2	16	8	32	500
**EB12lin**	2	16	16	16	>32	500
**EB13**	0.5	4	8	8	>32	>1000
**EB13lin**	2	16	16	32	>32	250

[a] Minimum inhibitory concentration (MIC), in µg/mL, was determined on bacteria in diluted Mueller-Hinton (MH 12.5%) broth at pH 7.4, after incubation for 16-20 hours at 37 °C. Values represent two different duplicate MIC determinations. [b] Minimum Hemolytic Concentration (MHC) measured on human red blood cells (hRBC) in 10 mM phosphate buffer, 150 mM NaCl, pH 7.4, 25 °C, 4 h. [c] Polymyxin B2.

8. Serum Stability Assay

Human serum was diluted in 0.1 M filtered TRIS buffer pH 7.4 (25%, 1:3, v/v). Selected hybrids were diluted in 0.1 M filtered TRIS buffer pH 7.4 to a concentration of 400 µM and 0.1 mg/mL of 4-hydroxybenzoic acid was added as internal standard. Aliquots of peptide solution (50 µL) were added to aliquots of serum (50 uL) in sterile Eppendorf tubes, to reach a peptide concentration of 200 µM during the assay. Samples were incubated at 37 °C under gentle stirring (350 rpm). Different samples (triplicates) were quenched at different time points (0 / 1 / 6 / 10 / 24 h) by precipitating serum proteins through the addition of 100 µL of a solution $ZnSO_4 \cdot 7\ H_2O$ (0.05 mM) in water/ACN (1:1) and cooling in ice bath for 10 minutes. Protein precipitates were pelleted under centrifugation (5 min at 14'000 rpm) and the supernatants were analysed by LC-MS. Experiment controls included a precipitation control for each peptide to test their resistance to the protein precipitation conditions and serum blanks to check reproducibility over different serum batches. Peaks corresponding to the internal standard and the undegraded peptides were integrated, with the ratio peptide/standard at t = 0 h as 100 % undegraded peptide.

9. Cell Viability Assay

Cell Culture Conditions. HEK293 cells were cultured and maintained in Dulbecco's Modified Eagle's Medium (DMEM) high glucose (Sigma Aldrich) medium, supplemented with 10% fetal bovine serum (FBS) (Sigma Aldrich) and 1% penicillin/streptomycin. HK-2 cells were cultured and maintained in keratinocyte serum free medium (K-SFM) (Sigma Aldrich), supplemented with bovine pituitary extract (BPE) (0.05 mg/mL) and human recombinant epidermal growth factor (EGF) (5 ng/mL). The cells were handled and sub-cultured according to the manufacturer instructions. Cells were incubated in a humidified incubator at 37°C in the presence of 5% CO_2.

Cell Viability Assay by Alamar Blue. HEK293 or HK-2 cells were seeded into 96 well plates, in amount 2×10^3, 4×10^3 cells/well respectively, the day before the experiment. HEK293 were seeded into well plates, pre-treated with solution of poly-L-Lysine. The medium was removed, and the compounds were added into the wells, with increasing concentrations. The cells were incubated for 72 h in 200 μL/well at 37°C in corresponding medium in the presence of 5% CO_2. After incubation time, the medium was removed and replaced by 100 μL/well of medium containing 10% Alamar Blue. The cells were incubated for 3-5 h at 37°C with 5% CO_2 in a humidified atmosphere. The fluorescence was then measured on a Molecular Devices Spectra Max I3x plate reader at λ_{ex} 560 nm and λ_{em} 590 nm. The value was normalized according to the untreated cells.

Table S5. Cytotoxicity evaluation for a selection of compounds, against HEK293 and HK-2 cells.

				**IC_{50} (μM)**				
**Cpd**	**EB12**	**EB13**	**EB16**	**EB18**	**EB21**	**EB22**	**EB33**	**PMB2**
**HEK293**	> 200	> 200	> 200	> 200	> 200	> 200	135 ± 20	56 ± 5
**HK-2**	58 ± 18	40 ± 10	23 ± 2	71 ±22	> 200	23 ± 2	6 ± 1	7 ± 1

All data represented as the IC_{50} value measured by Alamar blue assay after 24 h treatments with concentrations from 0 to 200 μM. The data are represented as the mean value ± SD, *n* = 3.

HEK293 cells

EB12

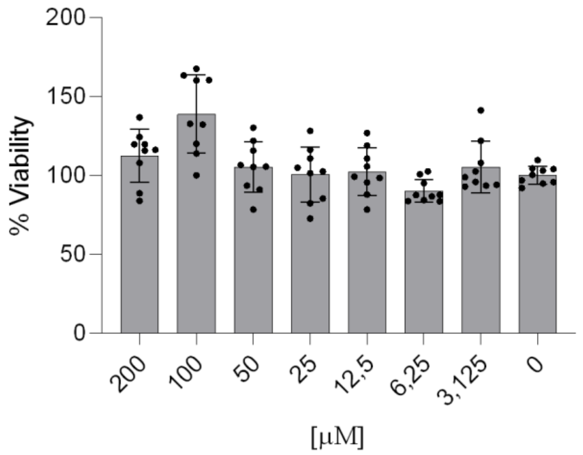

EB13

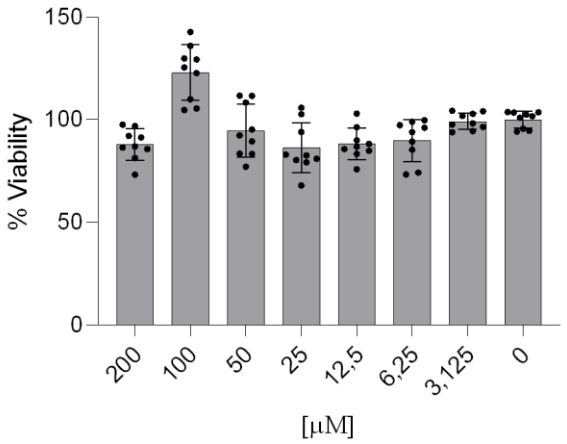

EB16

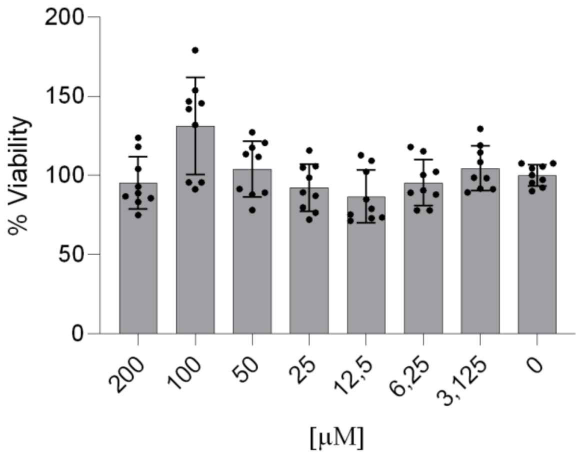

EB18

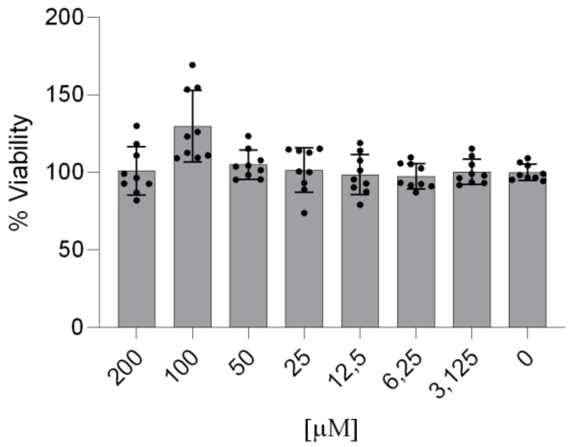

EB21

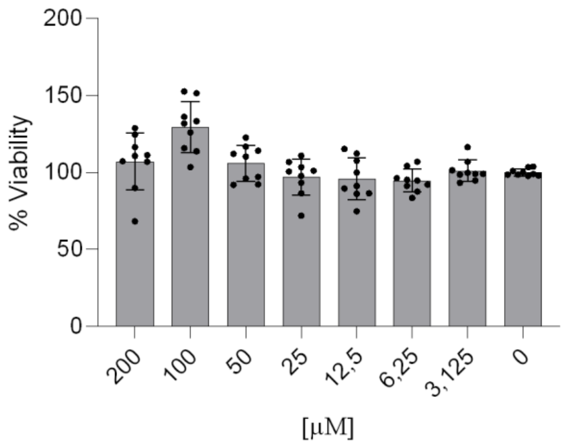

EB22

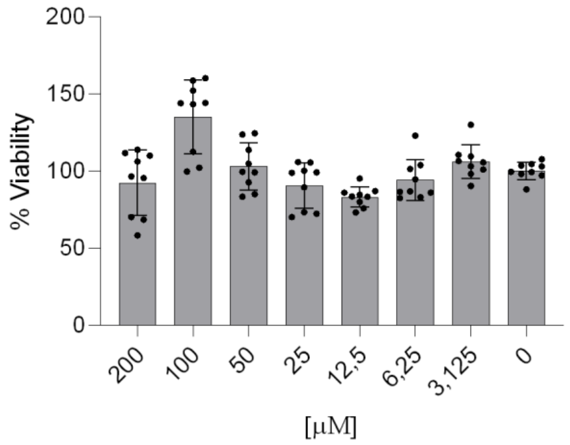

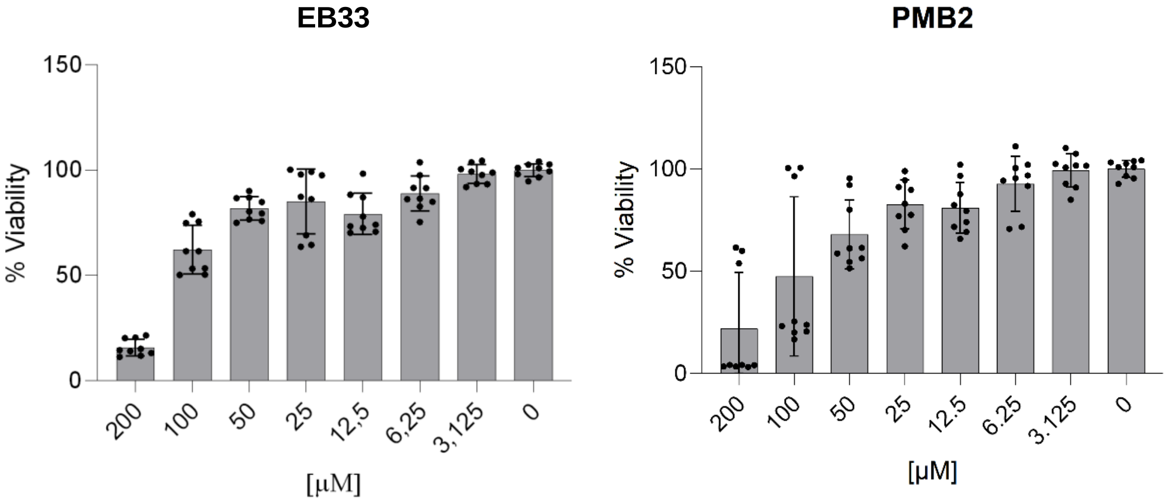

Figure S3. Cytotoxicity of selected compounds against HEK293 cells. The cells were treated with the compound at the desired concentration for 24 h and the viability was determined with an Alamar blue assay. Data of three experiments performed in triplicates were pooled and represented as barplots.

HK-2 cells

EB12

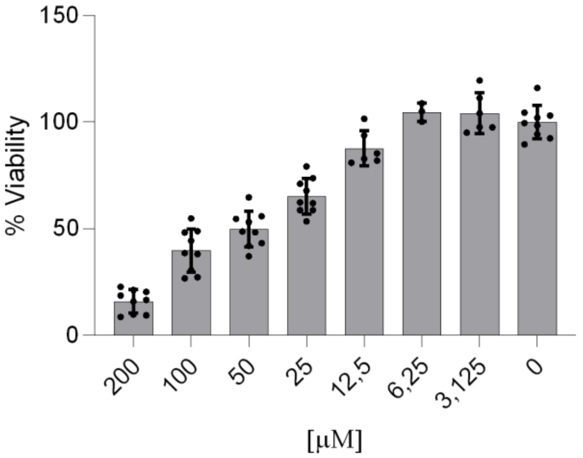

EB13

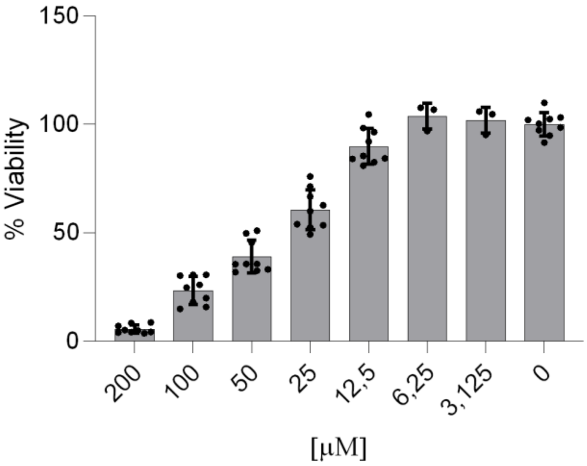

EB16

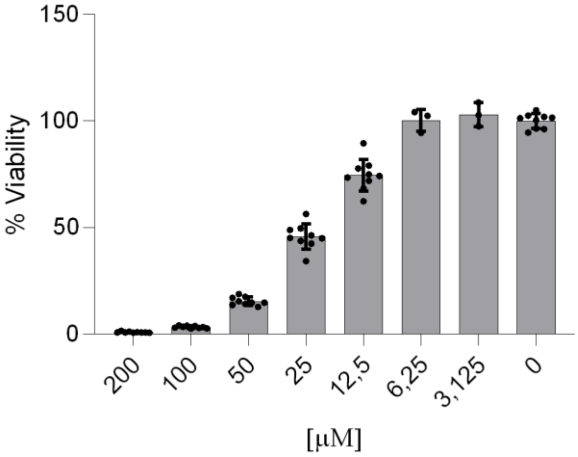

EB18

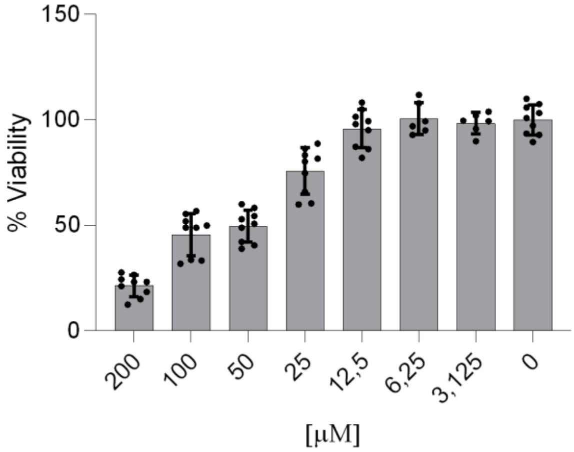

EB21

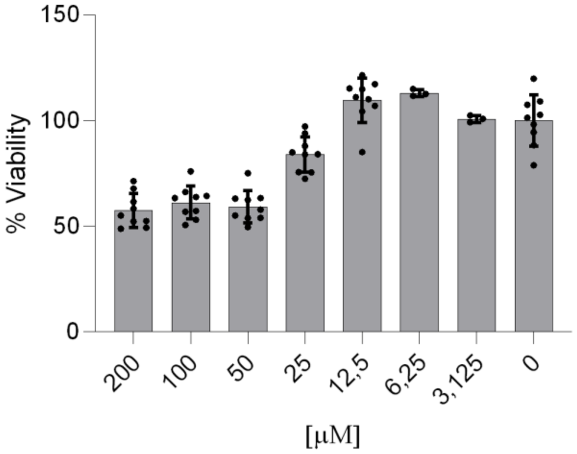

EB22

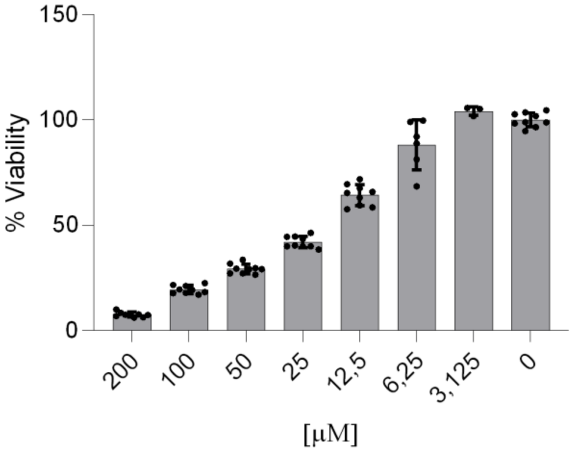

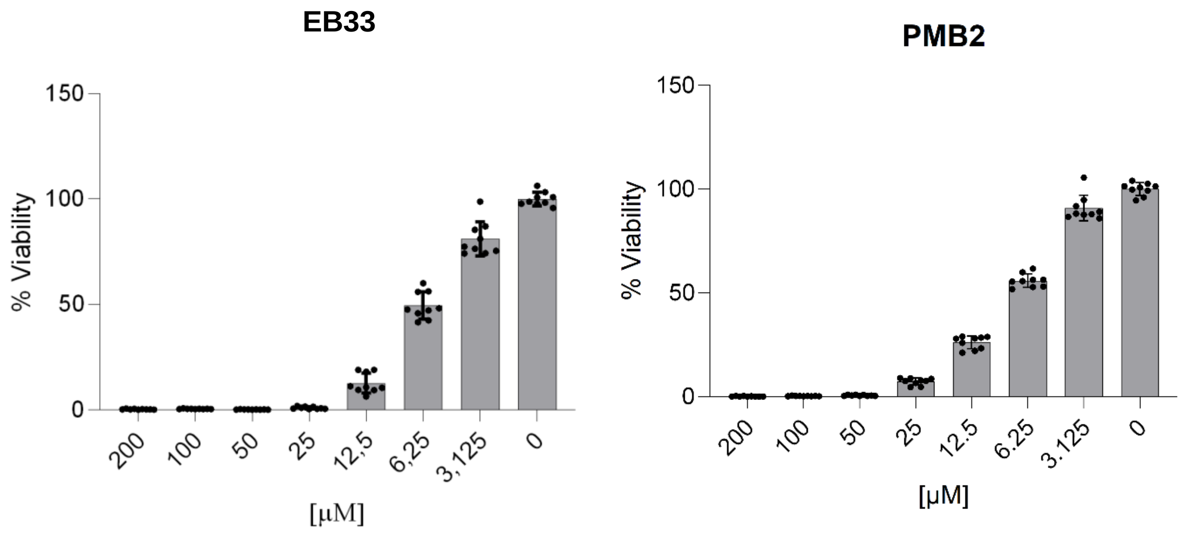

Figure S4. Cytotoxicity of selected compounds against HK-2 cells. The cells were treated with the compound at the desired concentration for 24 h and the viability was determined with an Alamar blue assay. Data of three experiments performed in triplicates were pooled and represented as barplots.

10. Lipid Vesicle Leakage Assay

5(6)-carboxyfluorescein (CF) was purchased from Sigma Aldrich. Egg Yolk Phosphatidylcholine (EYPC), Egg Yolk Phosphatidylglycerol (EYPG) and a Mini-Extruder were purchased from Avanti Polar Lipids.

Vesicles preparation. Egg yolk PC or egg yolk PG thin lipid layers were prepared by evaporating a solution of 100 mg EYPC or EYPG in 4 mL MeOH/$CHCl_3$ (1:1) on a rotary evaporator at room temperature and then dried in vacuo overnight. The resulting film was then hydrated with 2 mL CF buffer (50 mM CF, 10 mM TRIS, 10 mM NaCl, pH 7.4) for 30 min, subjected to freeze-thaw cycles (7×) and extrusion (15×) through a polycarbonate membrane (pore size 100 nm). Extra vesicular components were removed by gel filtration (Sephadex G-50) with 10 mM TRIS, 107 mM NaCl, pH 7.4 buffer. Final conditions: ca. 2.5 mM EYPC or EYPG; inside: 50 mM CF, 10 mM TRIS, 10 mM NaCl, pH 7.4 buffer; outside: 10 mM TRIS, 107 mM NaCl, pH 7.4.

Vesicle leakage assay. EYPC or EYPG vesicle stock solutions (37.5 μL) were diluted to 3000 μL with a buffer (10 mM TRIS, 107 mM NaCl, pH 7.4) in a thermostated fluorescence cuvette (25 °C) and gently stirred (final lipid concentration ca. 10 μg/mL for both EYPG and EYPC). The desired volume of peptide (1 or 20 mg/mL stock in MilliQ water) was added at t = 45 s to obtain final concentrations of 10 and 50 or 200 μg/mL, and CF efflux was monitored at λ_{em} 517 nm (λ_{ex} 492 nm) as a function of time. Finally, 30 μL of 1.2% Triton X-100 was added to the cuvette (0.012% final concentration) at t = 240 s to reach the maximum intensity. Fluorescence intensities were then normalized to the maximal emission intensity using $I(t) = (I_t - I_0)/(I_\infty - I_0)$ where $I_0 = I_t$ at peptide addition and $I_\infty = I_t$ at saturation of lysis.

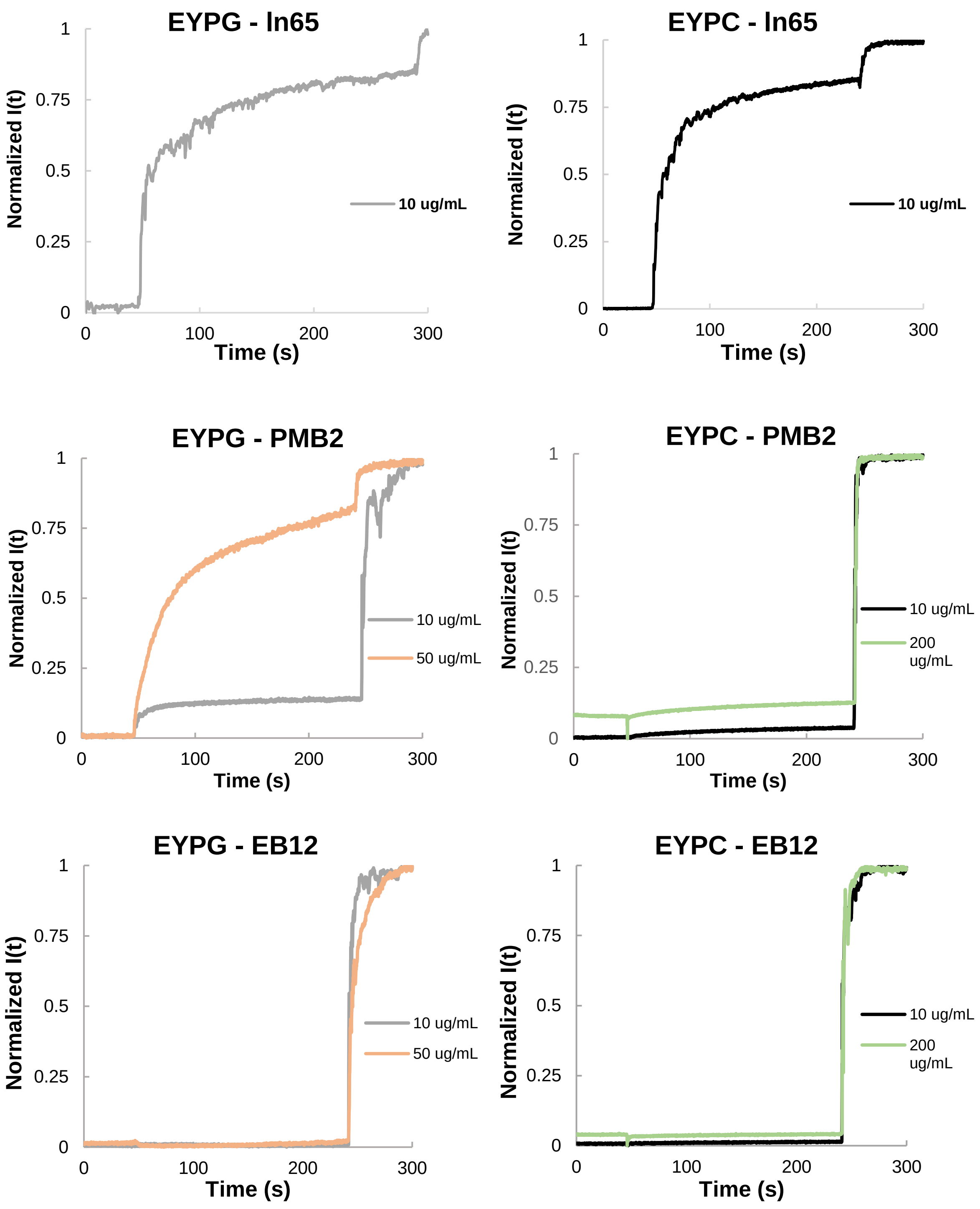
EYPG - In65
Normalized I(t)
Time (s)
10 ug/mL
EYPC - In65
Normalized I(t)
Time (s)
10 ug/mL
EYPG - PMB2
Normalized I(t)
Time (s)
10 ug/mL
50 ug/mL
EYPC - PMB2
Normalized I(t)
Time (s)
10 ug/mL
200 ug/mL
EYPG - EB12
Normalized I(t)
Time (s)
10 ug/mL
50 ug/mL
EYPC - EB12
Normalized I(t)
Time (s)
10 ug/mL
200 ug/mL

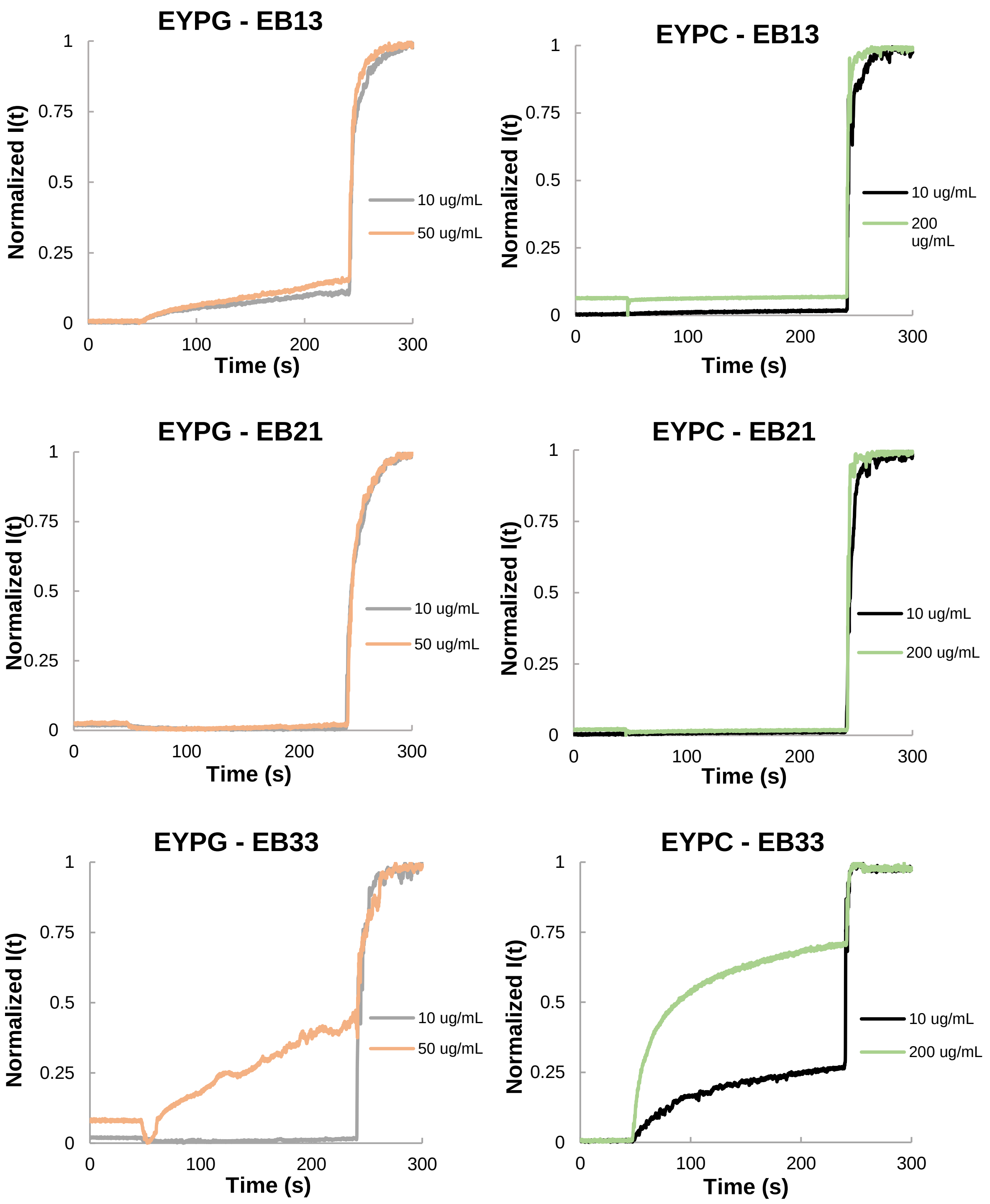

Figure S5. Vesicle leakage experiments using, measuring the release of 5(6)-carboxyfluorescein from the unilamellar giant vesicles, induced by peptides. EYPG and EYPC vesicles were suspended in buffer (10 mM TRIS, 107 mM NaCl, pH 7.4) and the indicated concentration of the compound was added after 45 sec. After 240 seconds, 30 µL Triton X-100 1.2% was added for full release of fluorescein. **ln65** was measured as positive control.[9]

11. Minimal inhibitory in presence of salt

The same procedure as for the antibacterial activity measurements by minimal inhibitory concentration assay (MIC) was applied. The media, 12.5% Mueller-Hinton broth was this time supplemented with different salt concentrations (0, 100, 200 300 mM NaCl). The MIC activities were then determined against *Pseudomonas aeruginosa* PAO1 and *Escherichia coli* W3110.

Table S6. Minimal inhibitory concentration (MIC) on *P. aeruginosa* PAO1 as function of salt concentration

**Cpd**	**MIC on *P. aeruginosa***				
**NaCl**	**Charge**	**0 mM**	**100 mM**	**200 mM**	**300 mM**
**EB9**	+5	2	32	> 64	> 64
**PMB2**	+5	0.5	0.25	0.25	0.25
**EB12**	+5	2 – 4	32	> 64	> 64
**EB13**	+5	4	> 64	> 64	> 64
**EB14**	+6	4	> 64	> 64	> 64
**EB16**	+5	4	> 64	> 64	> 64
**EB20**	+5	16	> 64	> 64	> 64
**EB21**	+5	4	> 64	> 64	> 64
**EB22**	+6	4	> 64	> 64	> 64
**EB23**	+4	16	> 64	> 64	> 64
**EB26**	+4	16	> 64	> 64	> 64
**EB32**	+4	32	> 64	> 64	> 64
**EB34**	+4	32	> 64	> 64	> 64
**EB36**	+3	32	> 64	> 64	> 64
**EB37**	+3	32	> 64	> 64	> 64

All data, in µg/mL, measured against *P. aeruginosa* PAO1, with or without addition of salt (NaCl). The values were measured in dilute medium (12.5% MH) and at slightly basic pH (pH = 8.5), after incubation for 16-20 h at 37 °C. Values represent two different duplicate MIC determinations.

Table S7. Minimal inhibitory concentration (MIC) on *E. coli* W3110 as function of salt concentration

**Cpd**		**MIC on *E. coli***			
**NaCl**	**Charge**	**0 mM**	**100 mM**	**200 mM**	**300 mM**
**EB9**	+5	2	16	64	> 64
**PMB2**	+5	0.25	0.25	0.25	0.25
**EB12**	+5	1	2	4	16
**EB13**	+5	0.5	16	> 64	> 64
**EB14**	+6	1	16	64	> 64
**EB16**	+5	0.5	32	> 64	> 64
**EB20**	+5	1	64	> 64	> 64
**EB21**	+5	1	16	> 64	> 64
**EB22**	+6	1	16	> 64	> 64
**EB23**	+4	2	> 64	> 64	> 64
**EB26**	+4	4	16	> 64	> 64
**EB32**	+4	4	> 64	> 64	> 64
**EB34**	+4	8	> 64	> 64	> 64
**EB36**	+3	16 – 32	> 64	> 64	> 64
**EB37**	+3	32	> 64	> 64	> 64

All data, in µg/mL, measured against *E. coli* W3110, with or without addition of salt (NaCl). The values were measured in dilute medium (12.5% MH) and at slightly basic pH (pH = 8.5), after incubation for 16-20 h at 37 °C. Values represent two different duplicate MIC determinations.

12. NPN Outer-Membrane Permeabilization Assay

A single colony of *P. aeruginosa* PAO1 and *E. coli* W3110 was grown overnight with shaking (150 rpm) in LB-broth (5 mL) at 37 °C. 100 µL of the overnight culture was regrown in 10 mL LB broth with shaking (200 rpm) to the exponential phase OD_{600} = 1 (10^9 CFU/mL). Bacteria were washed three times with HEPES buffer (5 mM HEPES, 5 mM glucose, pH 7.4), but not diluted yet to their final concentration. 200 µL peptide samples were added to the first well of 96-well plates (black wells, flat bottom, BRAND® GmbH Wertheim, Germany), 100 µL of HEPES/Glucose buffer with 10 µM *N*-Phenyl-1-naphthylamine (NPN, purchased from Acros Organics) was added in all the wells and the compounds were diluted serially by ½ (add a control with a final volume of 200 µL with 10 µM NPN in buffer only). Ca. 10 min before the measurement and the final dilution of the bacteria, the plates and the 10 µM NPN in HEPES/Glucose buffer (for bacteria dilution) were incubated at 37 °C (prior to the dilution of the bacteria with buffer containing NPN 10 µM). Later, the bacteria were diluted to OD_{600} = 0.5 with the NPN in HEPES/Glucose buffer. 100 µL of the bacterial suspension were added to each well. In this case, the final OD of bacteria was 0.25, the final concentration of the desired compound 64 µg/mL and NPN 10 µM. The control wells are buffer containing NPN (10 µM, 200 µL) and bacterial suspension containing NPN in HEPES buffer. The plate was measured with a Tecan instrument Infinite M1000 within 5 min. The plate was enabled to shake for 5 sec before measurement. The excitation wavelength used was 340 ± 5 nm and emission wavelength 415 ± 5 nm. The assay was repeated at least three times.

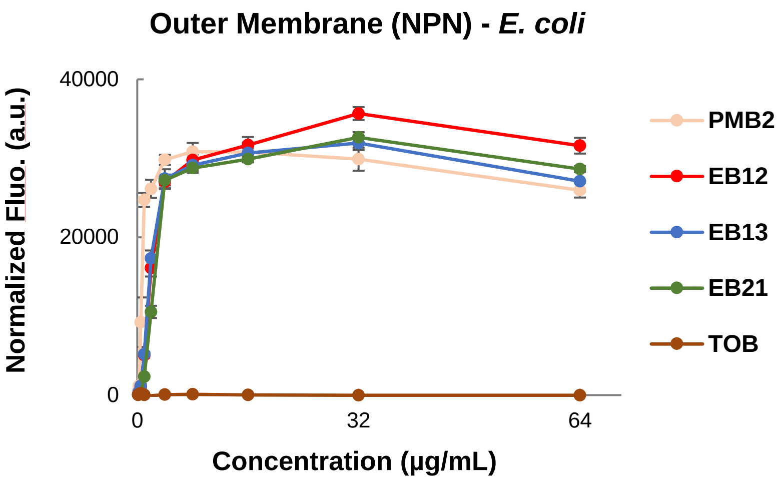

Figure S6. Outer membrane permeability assay with 10 µM NPN, on *E. coli* W3110.

13. DiSC3(5) Inner-Membrane Depolarization Assay

A single colony of *P. aeruginosa* PAO1 and *E. coli* W3110 was grown overnight with shaking (150 rpm) in LB broth (5 mL) at 37 °C. 100 µL of the overnight culture was regrown in 10 mL LB broth with shaking (200 rpm) to the exponential phase OD_{600} = 1 (10^9 CFU/mL). Bacteria were washed once with HEPES buffer (5 mM HEPES, 5 mM glucose, pH 7.4) and diluted to OD_{600} = 0.5. Stock solution of 10 mM of 3,3′-Dipropylthiadicarbocyanine iodide (DiSC3(5), purchased from Sigma) was prepared in DMSO. Stock solutions of 2 mg/mL of the compounds were prepared in sterilized milli-Q water and diluted to the beginning concentration of 128 µg/mL in 200 µL HEPES buffer. The diluted samples were added to the first well of 96-well plates (black wells, flat bottom, BRAND® GmbH Wertheim, Germany) and diluted serially by 1/2. 100 µL of the bacterial suspension in HEPES buffer (with 4 µM fluorescent probe DiSC3(5)) were added to each well. In this case, the final OD of bacteria was 0.25, the final concentration of peptide in the first column is 64 µg/mL and DiSC3(5) is 2 µM. The control wells are buffer containing DiSC3(5) and bacterial suspension containing DiSC3(5) in HEPES buffer. The plate was measured with a Tecan instrument Infinite M1000 within 5 min. The plate was enabled to shake for 5 sec before measurement. The excitation

wavelength used was 610 nm ± 5 nm and the emission wavelength 660 nm ± 5 nm. The assay was measured in triplicate and repeated at least two times.[10,11]

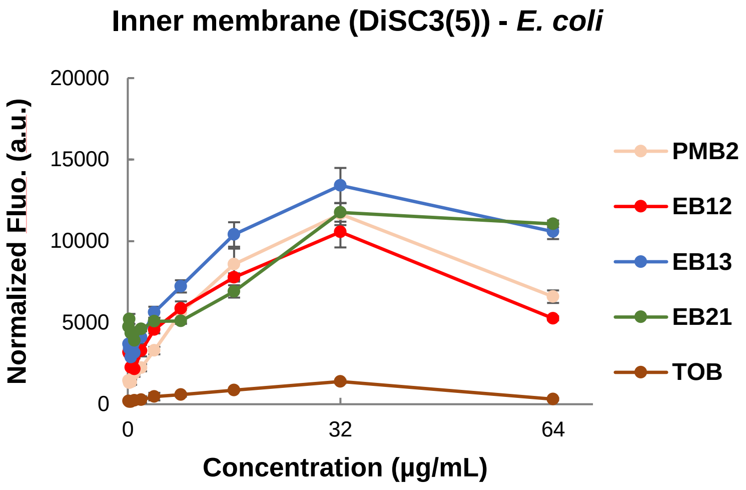

Figure S7. Inner membrane depolarization assay with 2 µM DiSC3(5), on *E. coli* W3110.

14. Dansyl-Polymyxin (DsPMB) Displacement Assay

Saturation determination. Dansyl-polymyxin (DsPMB) in water (1.5 mg/mL) and lyophilized lipopolysaccharides from *P. aeruginosa* were obtained from Merck. LPS were prepared as a 1 mg/mL stock solution in mQ water and stored at 4 °C. The fluorescence saturation of DsPMB with LPS was first determined, by titration (0.25 µM increments, up to 5 µM) of the DsPMB into a 3 µg/mL of LPS (10 mM Tris, 10 mM NaCl buffer, pH 7.4), at 37 °C. The fluorescence was recorded with a Jasco spectrofluorometer FP-8300, with the excitation at λ_{ex} = 340 nm (5 nm slit width) and recorded from 420 to 650 nm. The fluorescence was recorded two minutes after each addition. Saturation was graphically determined, at a concentration of DsPMB ≈ 2.5 µM (λ_{em} = 485 nm). Each measurement was determined in triplicates.

Displacement assay. The displacement effect of the various peptides was determined by measuring the reduction of fluorescence intensity, at 37 °C. Initially, DsPMB (2.5 µM) was mixed with LPS (3 µg/mL) and let it react for 15 minutes before recording the fluorescence

intensity (at λ_{em} = 485 nm and λ_{ex} = 340 nm). Then, the peptide was titrated (1 μM increments, up to 10 μM) into the DsPMB/LPS mixture, and the fluorescence was recorded two minutes after the compound addition. The decrease in fluorescence was interpreted as the displacement of DsPMB from the LPS due to competitive binding by the peptide.[12,13]

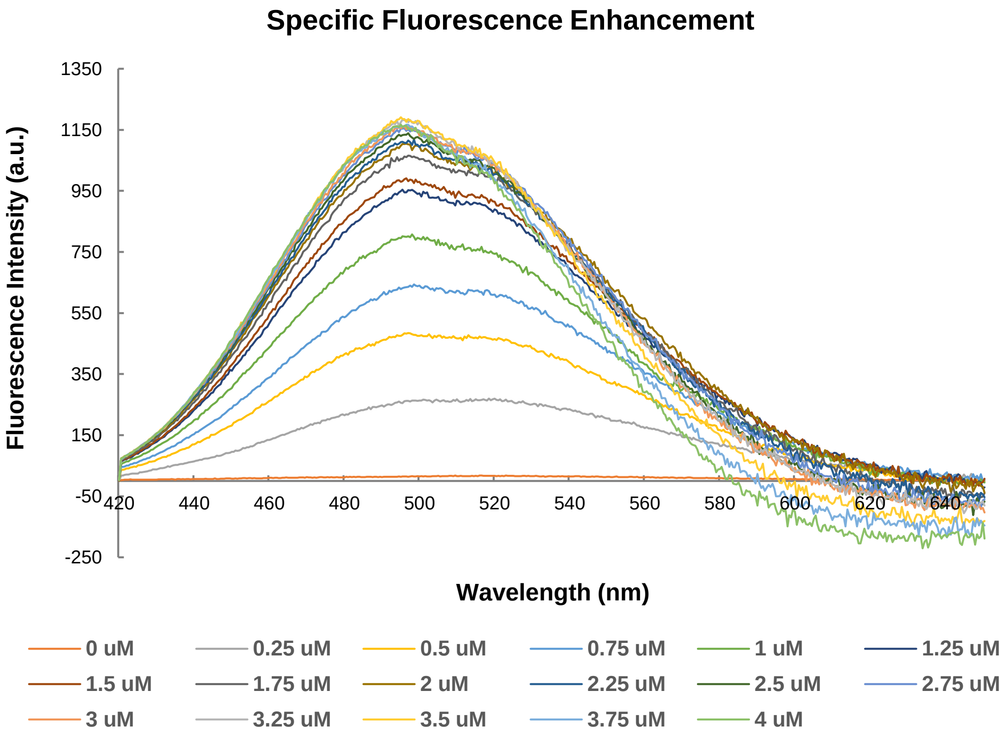

Figure S8. Fluorescence emission of dansyl-polymyxin (DsPMB) in presence of lipopolysaccharide (LPS) extracted from P. aeruginosa (3 μg/mL) in TRIS/NaCl buffer (10 mM, pH 7.4). The fluorophore was excited at 340 nm (5 nm slit width) and recorded from 420 to 650 nm. The data represent mean value from three measurement. Specific fluorescence represents the emission of DsPMB with the non-specific fluorescence (no LPS) deduced.

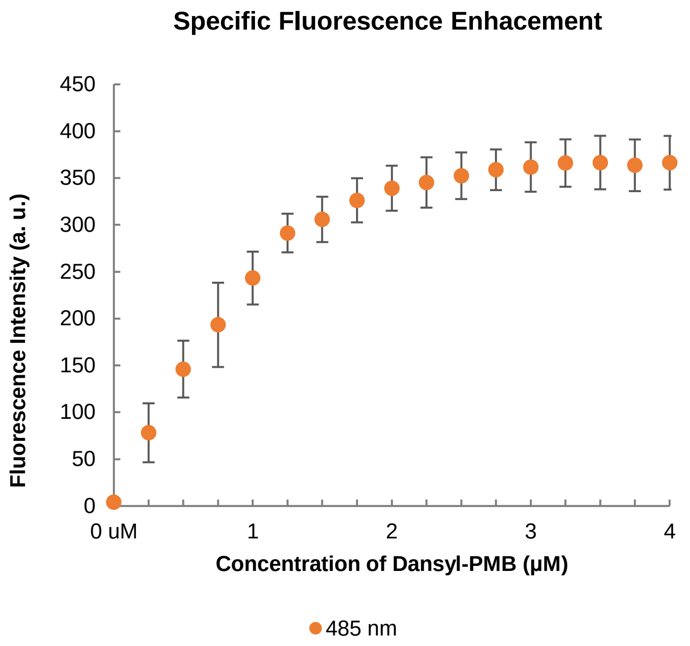

Figure S9. Extracted data from the specific fluorescence enhancement data (**Figure S8**) from DsPMB at 485 nm. The plateau is reached at approximately 2.5 µM. Each data point represent mean ± SD, n =3.

15. Transmission Electron Microscopy (TEM)

Transmission electron microscopy was evaluated at a high cell density (10^8 CFU) and a concentration (10x MIC) that killed the bacteria, but not all of them. An overnight culture of *P. aeruginosa* PAO1 was regrown until exponential phase (1 mL, OD_{600} = 0.5) in 12.5% MH medium pH 7.4, treated with the compounds, in diluted MH medium (at pH 7.4) and incubated for 2 hours at 37 °C. Just before the samples were further prepared for the microscopy, surviving bacteria were quantified by plating 10-fold dilutions of samples in sterilized normal saline (NaCl 0.9 %) on LB agar plates. LB agar plates were incubated at 37 °C for 10 hours and the number of individual colonies was counted at each time-point, to monitor partial bacteria killing. The assay was performed in triplicates. Each bacteria sample was centrifuged at 12’000 rpm for 3 min and the supernatant was discarded. The samples were fixed overnight with 2.5% glutaraldehyde (Agar Scientific, Stansted, Essex, UK) in 0.15 M HEPES (Fluka, Buchs, Switzerland) with an osmolarity of 630 mOsm and adjusted to a pH of 7.44. The next day, samples were washed with 0.15 M HEPES three times for 5 min at 4 °C, postfixed with 1% OsO_4

(SPI Supplies, West Chester, USA) in 0.1 M Na-cacodylate-buffer (Merck, Darmstadt, Germany) at 4 °C for 1 h. Thereafter, bacteria cells were washed in 0.1 M Na-cacodylate-buffer twice for 5 min at 4 °C and dehydrated in 70, 80, and 96% ethanol (Alcosuisse, Switzerland) for 15 min each at room temperature. Subsequently, they were immersed in 100% ethanol (Merck, Darmstadt, Germany) three times for 10 min, in acetone (Merck, Darmstadt, Germany) two times for 10 min, and finally in acetone-Epon (1:1) overnight at room temperature. The next day, bacteria cells were embedded in Epon (Fluka, Buchs, Switzerland) and hardened at 60 °C for 5 days. Sections were produced with an ultramicrotome UC6 (Leica Microsystems, Vienna, Austria), first semithin sections (1µm) for light microscopy which were stained with a solution of 0.5% toluidine blue O (Merck, Darmstadt, Germany) and then ultrathin sections (70-80 nm) for electron microscopy. The sections, mounted on single slot copper grids, were stained with 1% uranyl acetate at 40 °C for 30 min and 3% lead citrate at RT for 20 min or UranyLess (Electron Microscopy Sciences, Hatfield, UK) at 40 °C for 10 min and 3% lead citrate at 25 °C for 10 min with an ultrostainer (Leica Microsystems, Vienna, Austria). Sections were then examined with a Tecnai Spirit transmission electron microscope equipped with two digital cameras (FEI Eagle CCD Camera). The growth, incubation and fixation were performed in the biosafety level 2 lab.

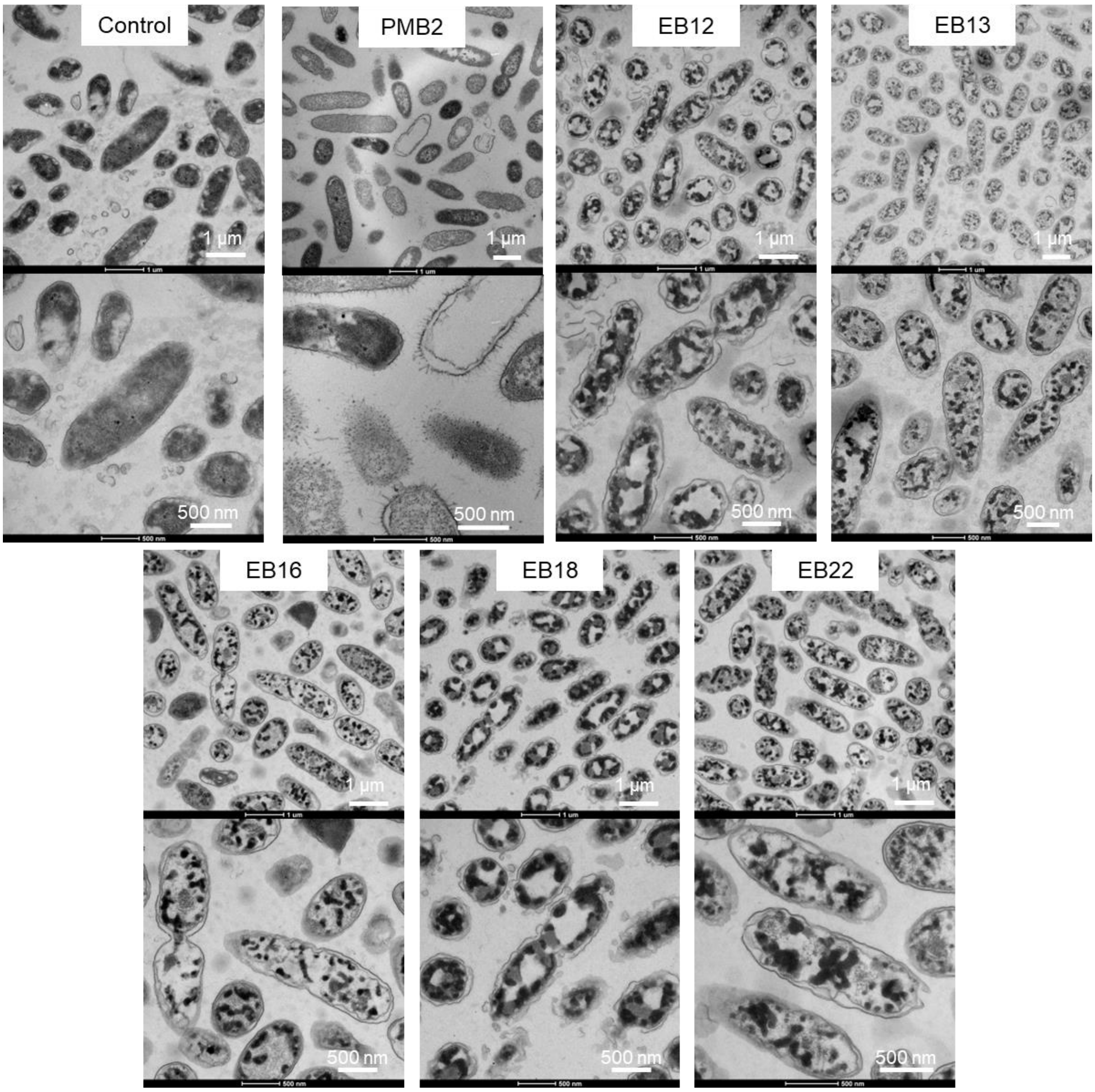

Figure S10. Transmission electron microscopy (TEM) micrograms of *P. aeruginosa* PAO1, treated with 10x the respective MIC value (12.5% MH, pH 7.4), and incubated for 2 h at 37 °C.

16. HPLC and MS data

PMB2 (**Polymyxin B2**) was obtained as a foamy white solid after preparative RP-HPLC (40 mg, 9 %). **Analytical RP-HPLC**: t_R = 2.95 min (A/D 100:0 to 0:100 in 7.0 min, λ = 214 nm). **MS** (ESI$^+$): $C_{55}H_{96}N_{16}O_{13}$ calc./obs. 1188.7343/1188.7338 Da [M].

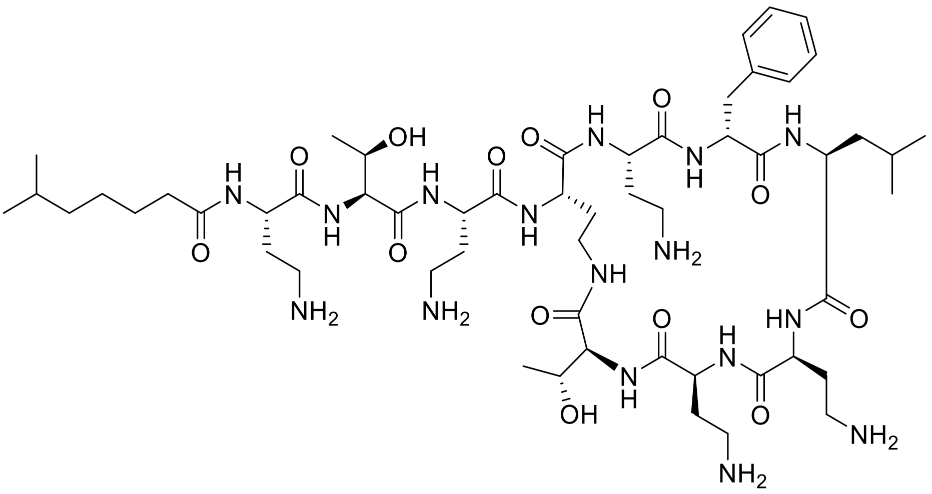

Chemical Formula: $C_{55}H_{96}N_{16}O_{13}$
Exact Mass: 1188.7343
Molecular Weight: 1189.4720

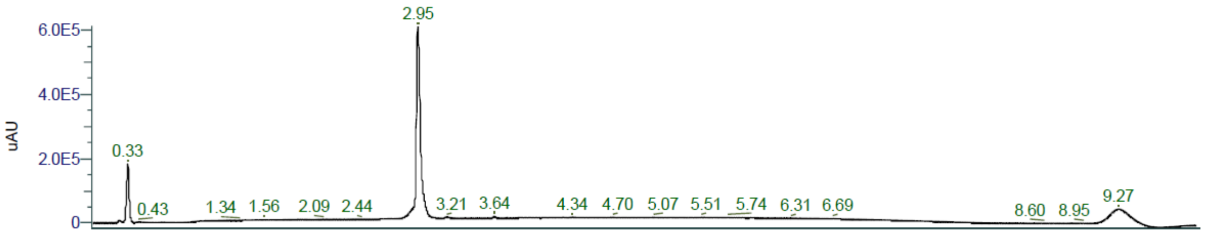

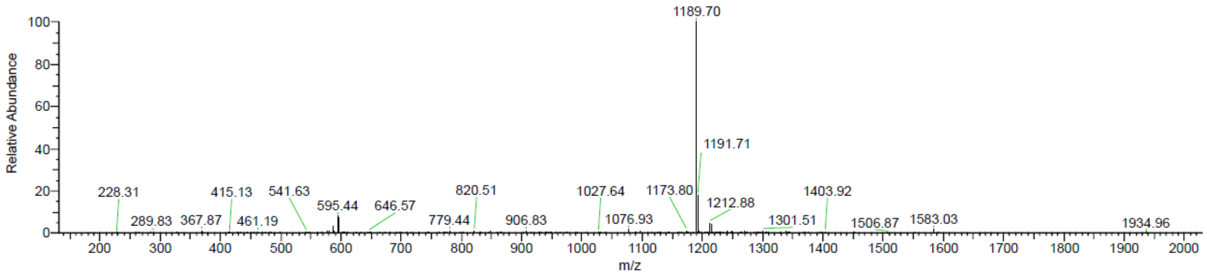

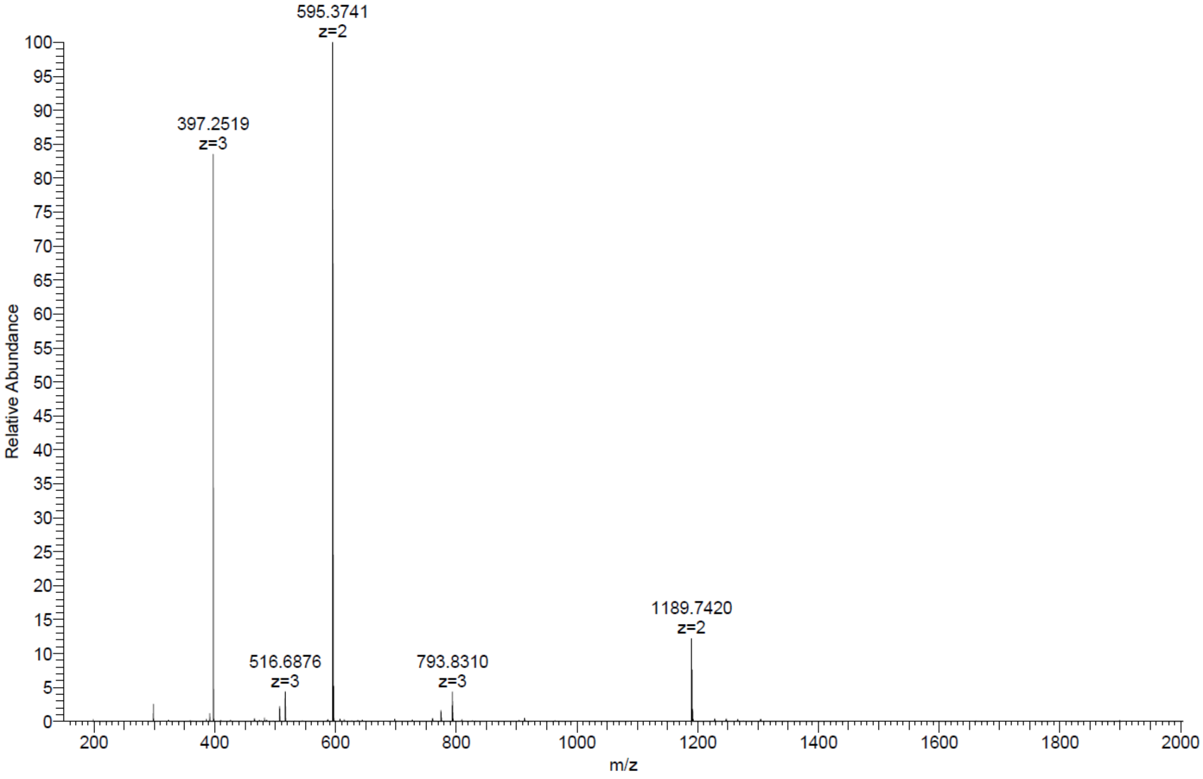

595.3741
z=2
397.2519
z=3
516.6876
z=3
793.8310
z=3
1189.7420
z=2
Relative Abundance
m/z

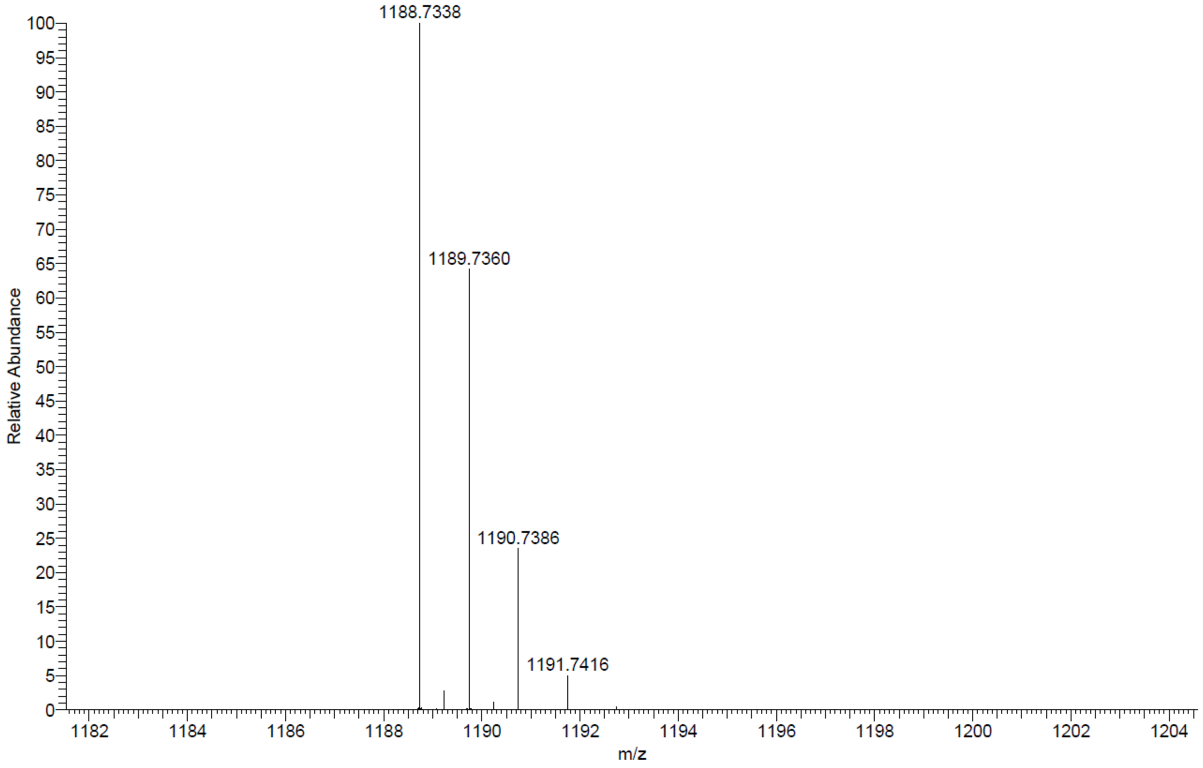

1188.7338
1189.7360
1190.7386
1191.7416
Relative Abundance
m/z

EB12 was obtained as a foamy white solid after preparative RP-HPLC (77 mg, 22 %). **Analytical RP-HPLC**: t_R = 3.05 min (A/D 100:0 to 0:100 in 7.0 min, λ = 214 nm). **MS** (ESI$^+$): $C_{60}H_{106}N_{16}O_{11}$ calc./obs. 1226.8227/1226.8207 Da [M].

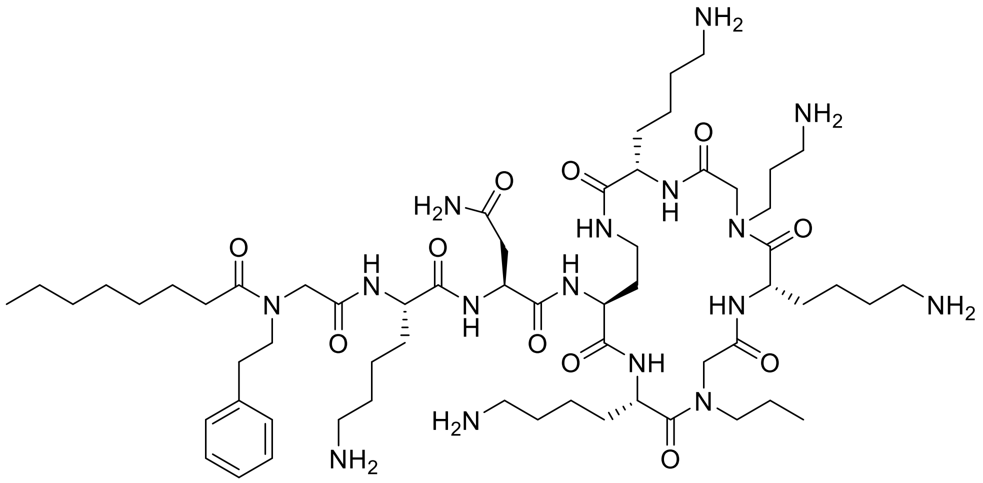

Chemical Formula: $C_{60}H_{106}N_{16}O_{11}$
Exact Mass: 1226.8227
Molecular Weight: 1227.6090

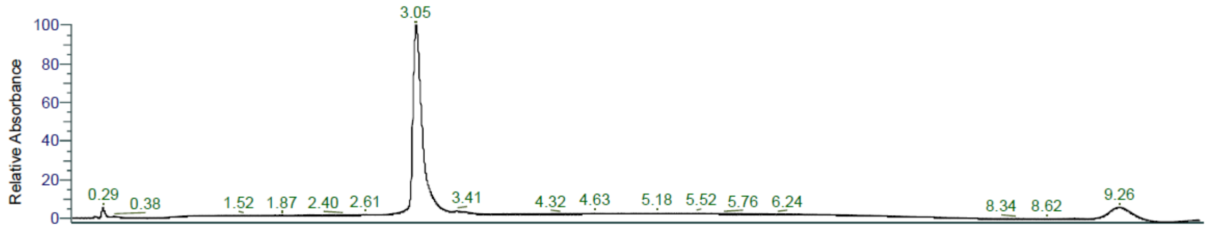

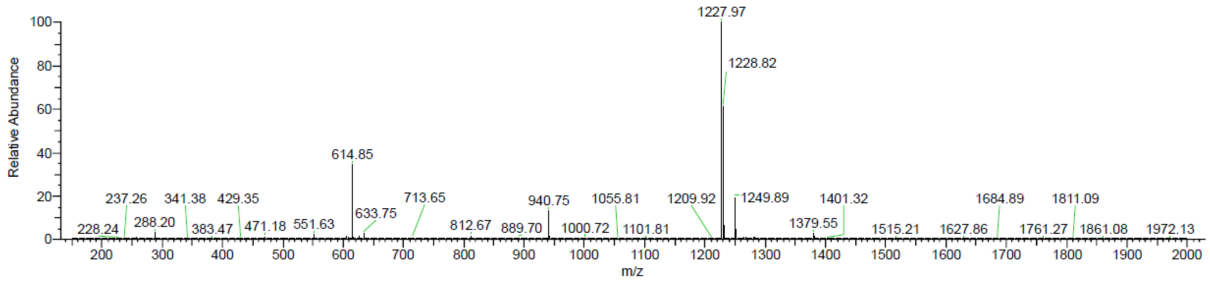

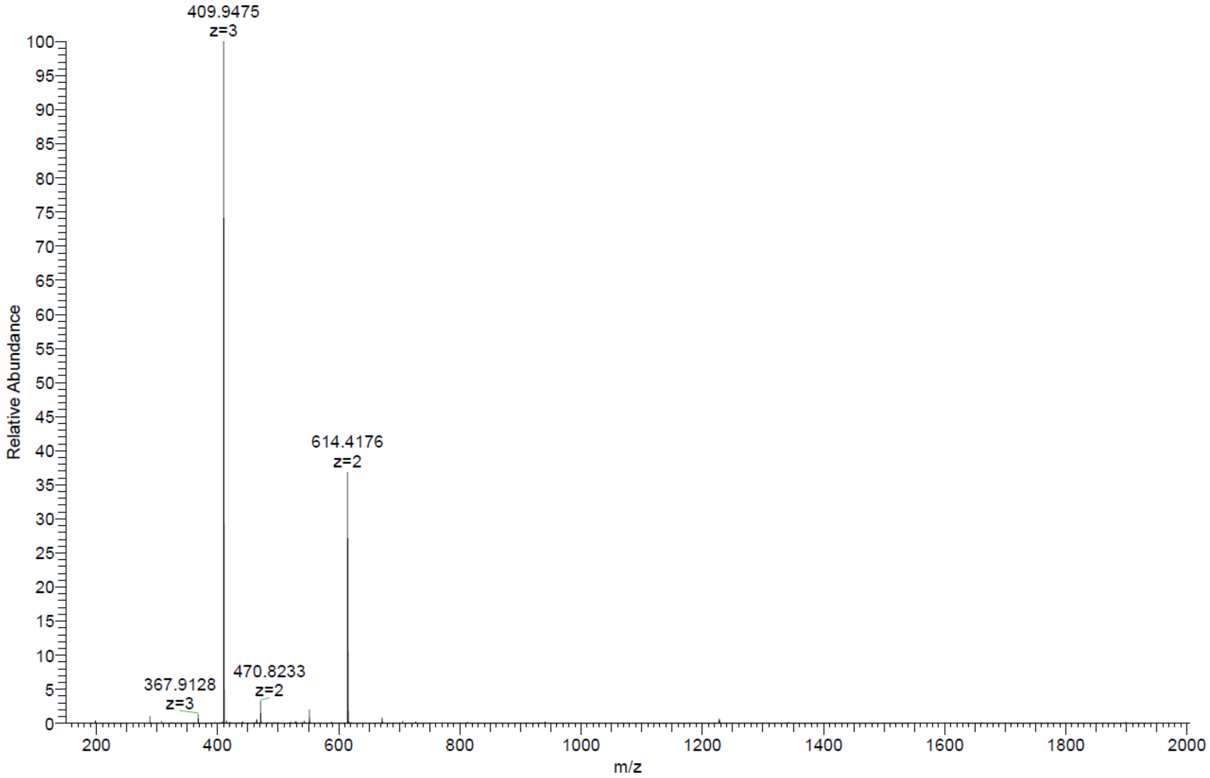
409.9475
z=3
614.4176
z=2
367.9128
z=3
470.8233
z=2
Relative Abundance
m/z

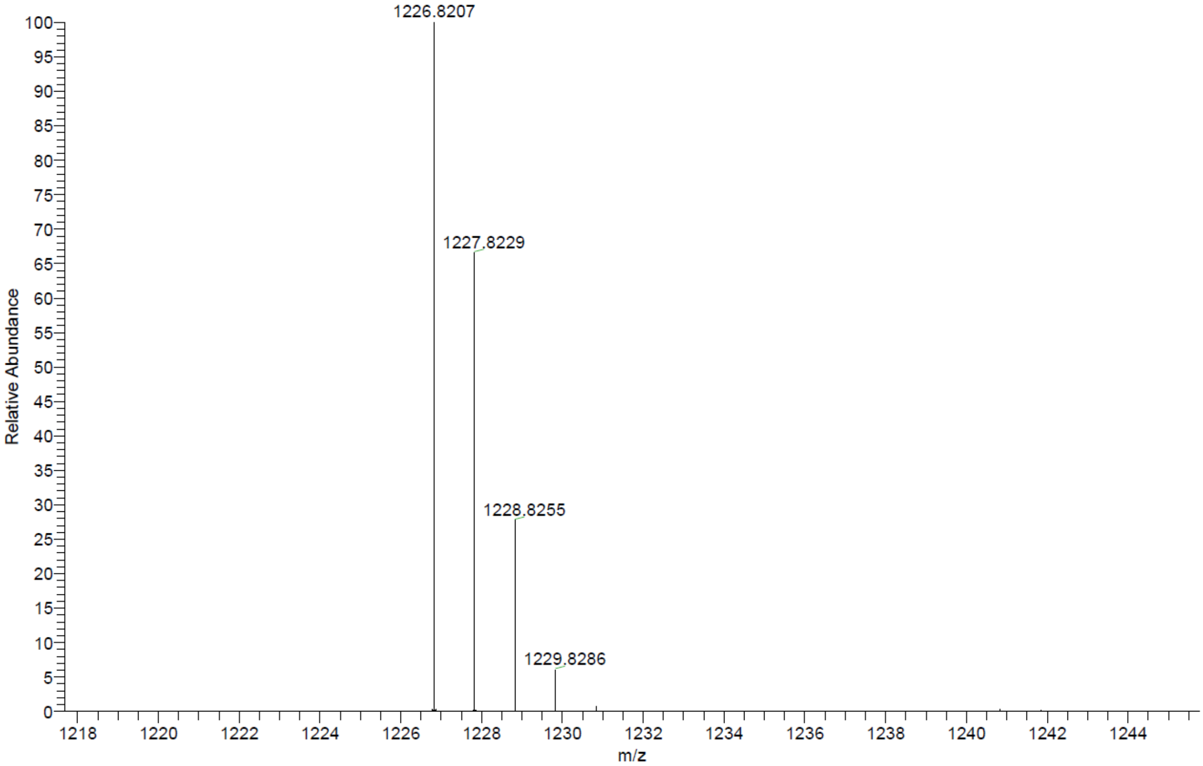
1226.8207
1227.8229
1228.8255
1229.8286
Relative Abundance
m/z

NMR EB12:

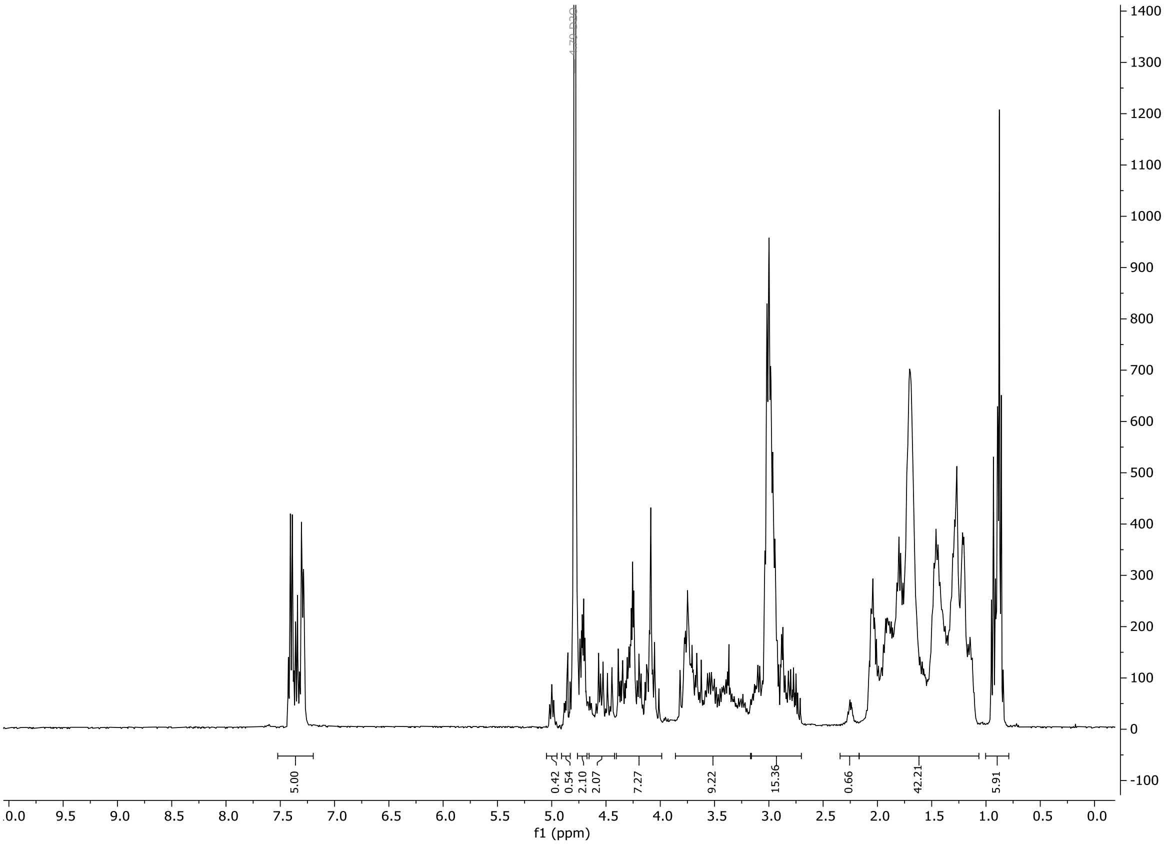

4.79 D2O
1400
1300
1200
1100
1000
900
800
700
600
500
400
300
200
100
0
-100
5.00
0.42
0.54
2.10
2.07
7.27
9.22
15.36
0.66
42.21
5.91
.0.0 9.5 9.0 8.5 8.0 7.5 7.0 6.5 6.0 5.5 5.0 4.5 4.0 3.5 3.0 2.5 2.0 1.5 1.0 0.5 0.0
f1 (ppm)

EB13 was obtained as a foamy white solid after preparative RP-HPLC (63 mg, 19 %). **Analytical RP-HPLC**: t_R = 3.03 min (A/D 100:0 to 0:100 in 7.0 min, λ = 214 nm). **MS** (ESI^+): $C_{56}H_{99}N_{15}O_{10}$ calc./obs. 1141.7699/1141.7690 Da [M].

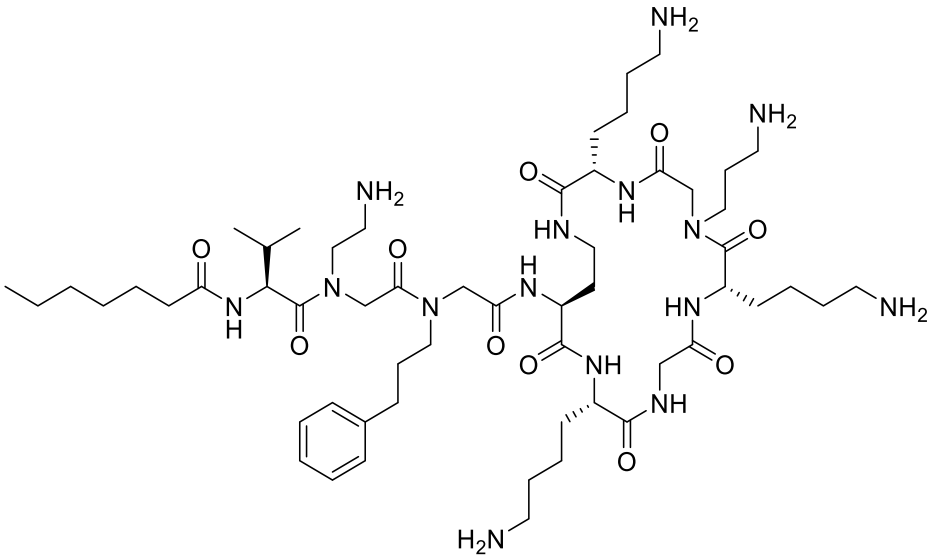

Chemical Formula: $C_{56}H_{99}N_{15}O_{10}$
Exact Mass: 1141.7699
Molecular Weight: 1142.5030

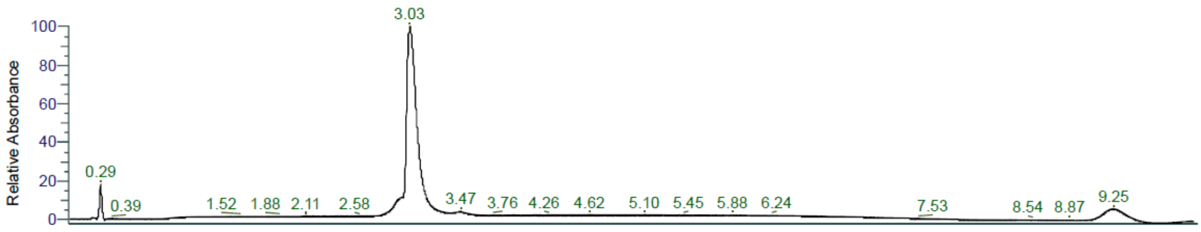

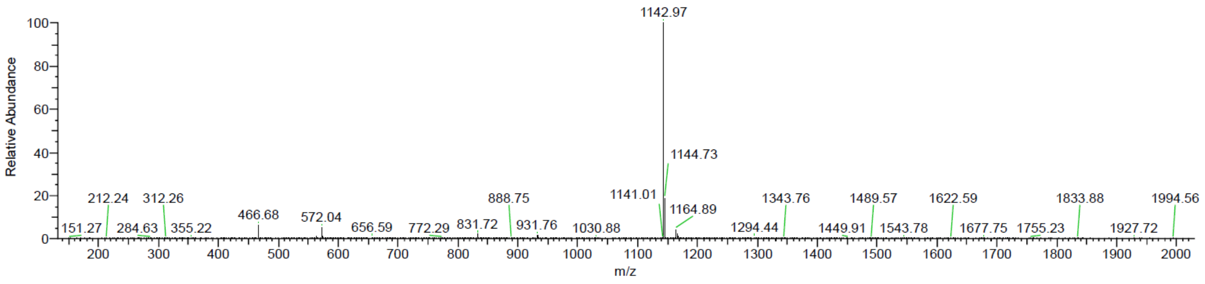

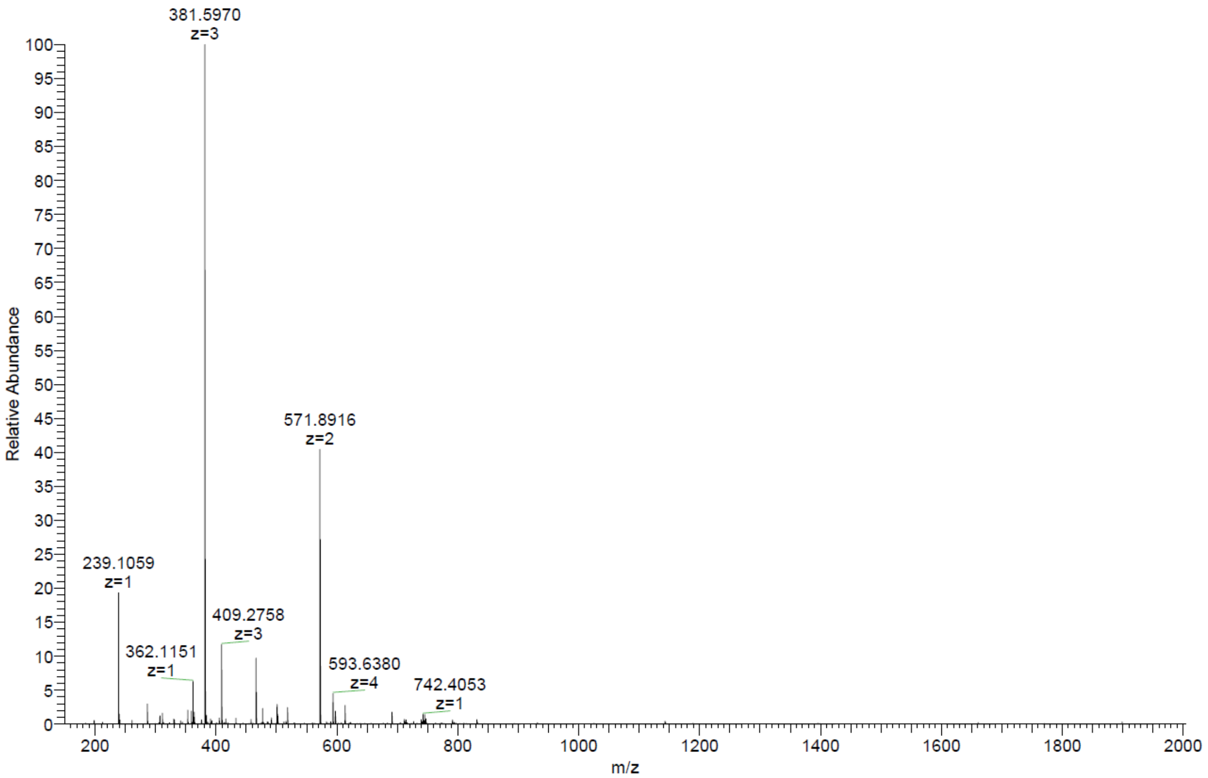
381.5970
z=3
571.8916
z=2
239.1059
z=1
409.2758
z=3
362.1151
z=1
593.6380
z=4
742.4053
z=1
Relative Abundance
m/z

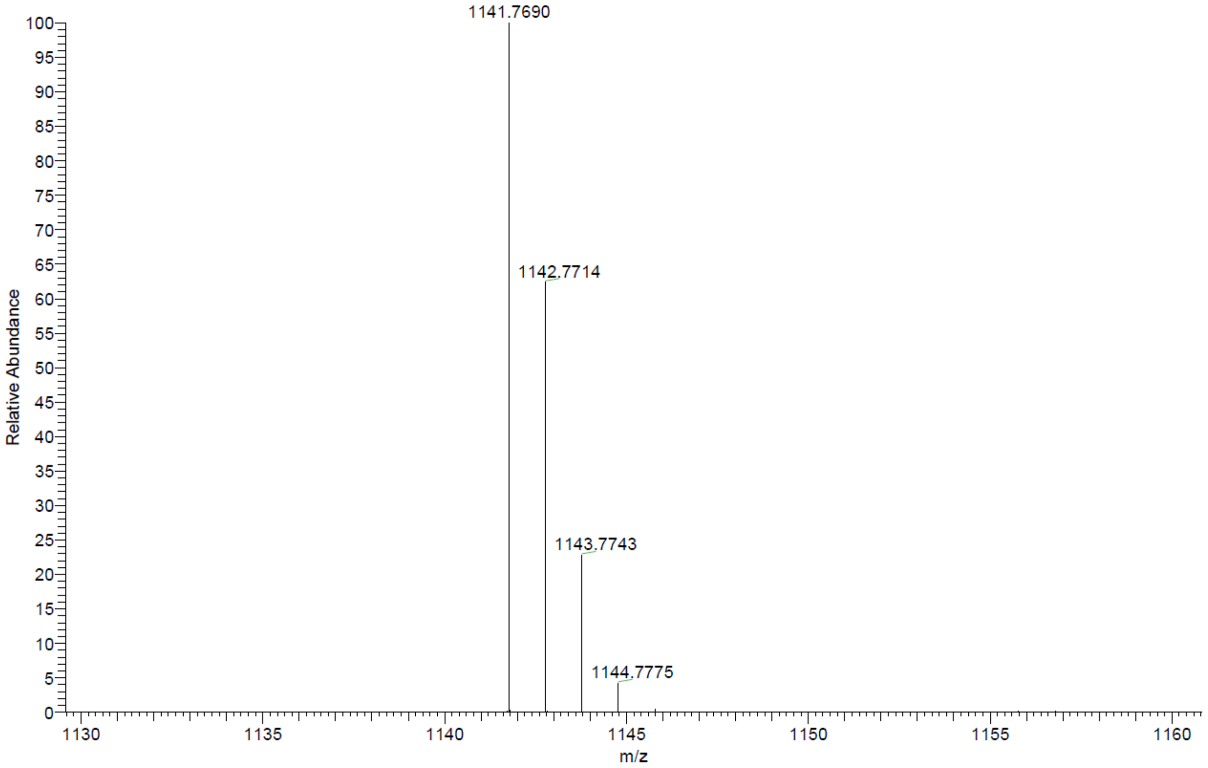
1141.7690
1142.7714
1143.7743
1144.7775
Relative Abundance
m/z

EB14 was obtained as a foamy white solid after preparative RP-HPLC (29 mg, 10 %). **Analytical RP-HPLC**: t_R = 2.38 min (A/D 100:0 to 0:100 in 7.0 min, λ = 214 nm). **MS** (ESI$^+$): $C_{53}H_{95}N_{17}O_{10}$ calc./obs. 1129.7448/1129.7439 Da [M].

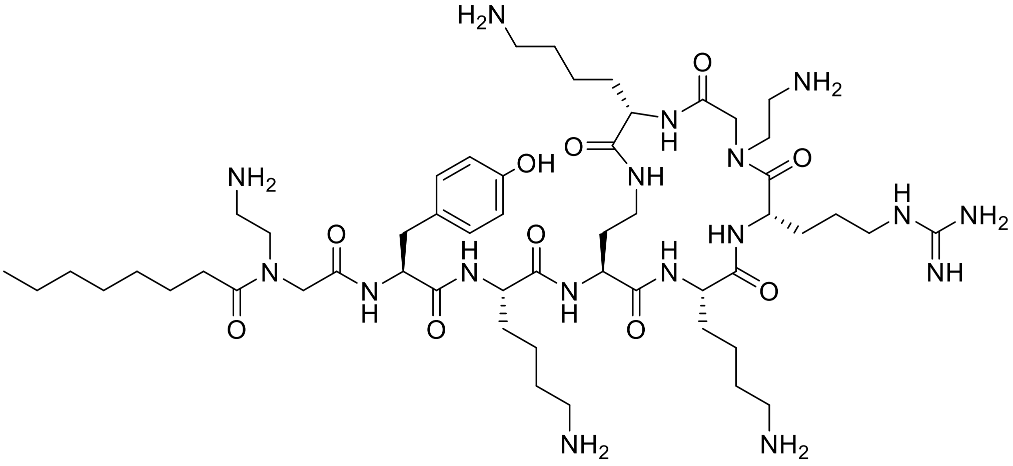

Chemical Formula: $C_{53}H_{95}N_{17}O_{10}$
Exact Mass: 1129.7448
Molecular Weight: 1130.4520

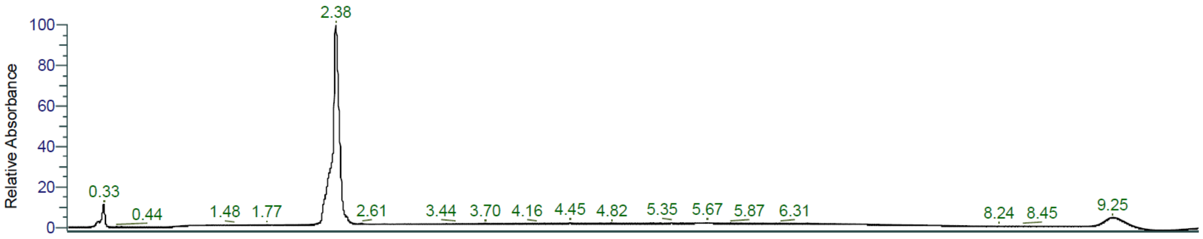

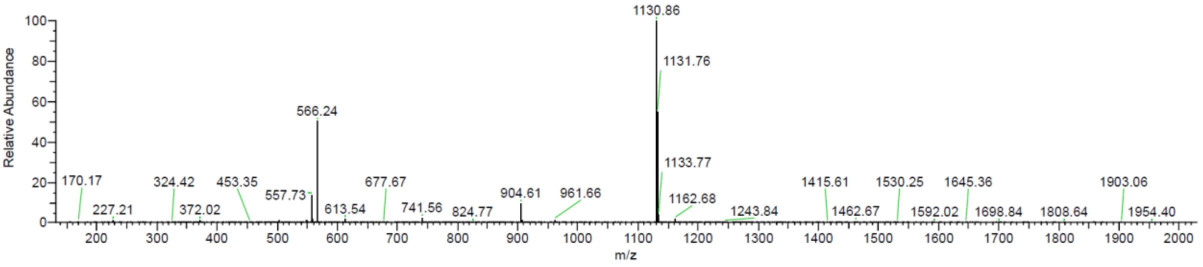

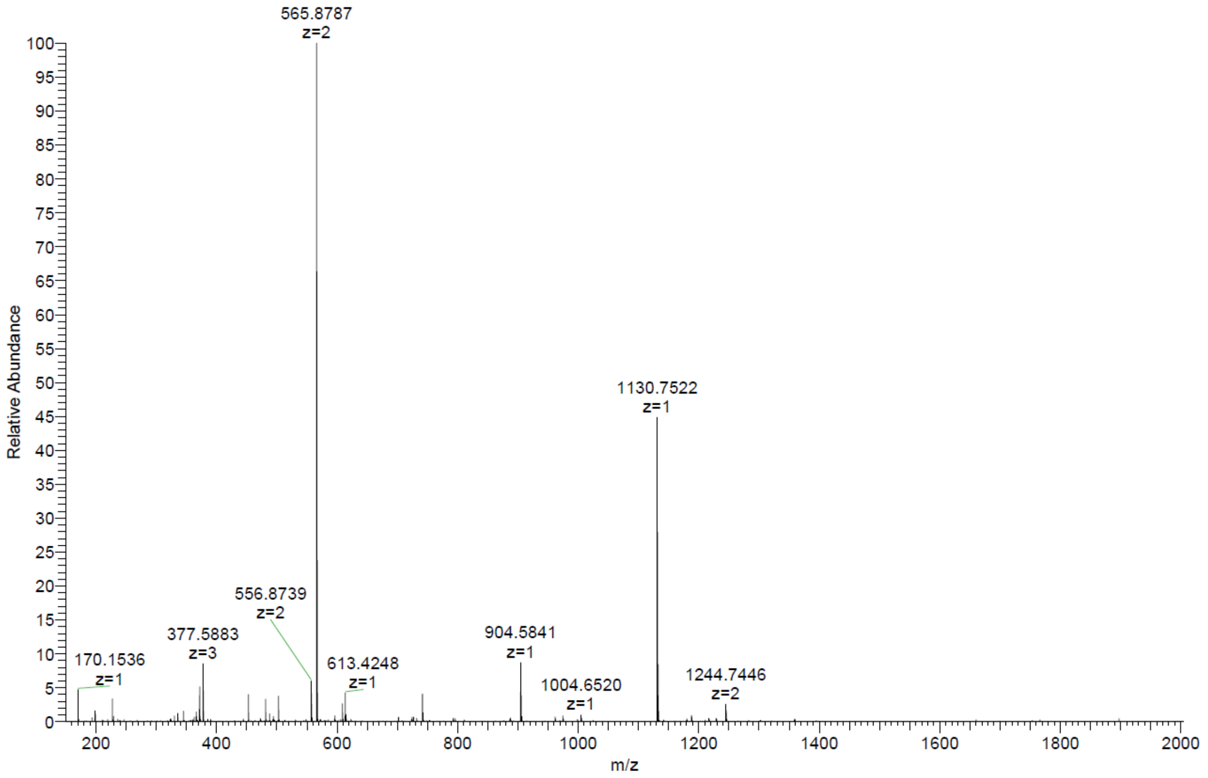
565.8787
z=2
1130.7522
z=1
556.8739
z=2
377.5883
z=3
904.5841
z=1
170.1536
z=1
613.4248
z=1
1004.6520
z=1
1244.7446
z=2
Relative Abundance
m/z

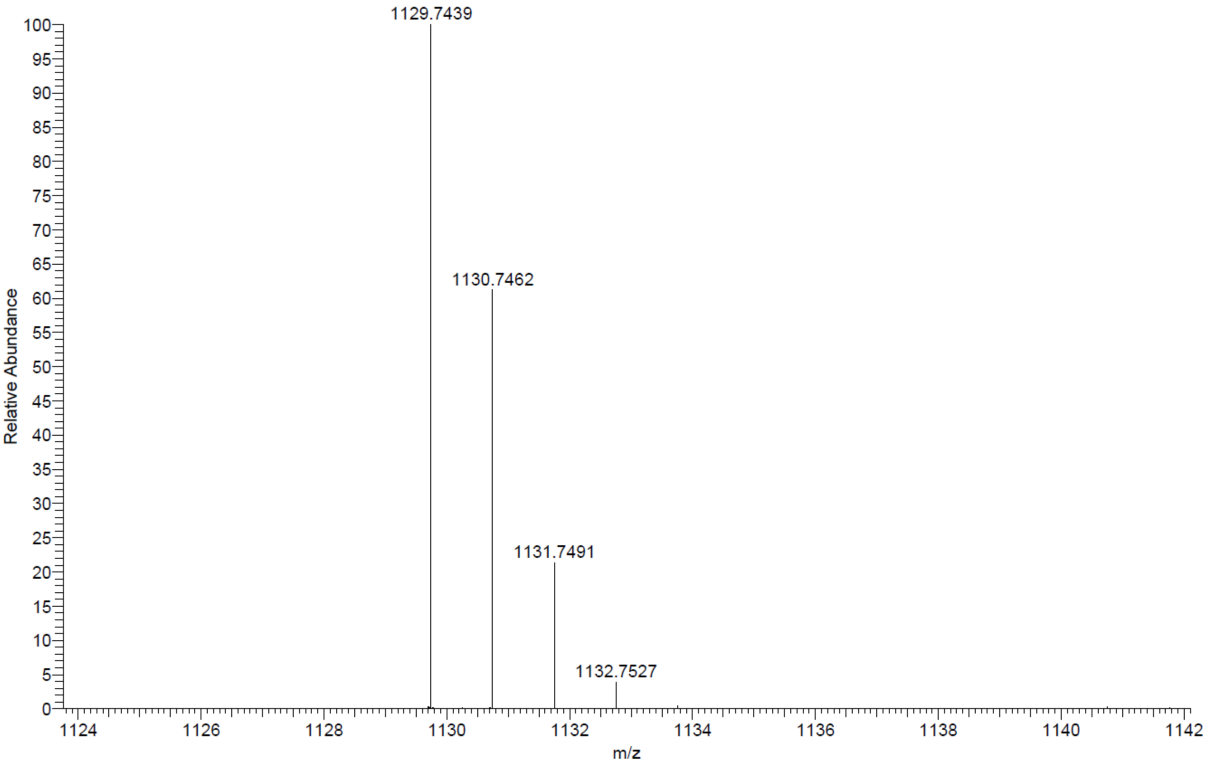
1129.7439
1130.7462
1131.7491
1132.7527
Relative Abundance
m/z

EB15 was obtained as a foamy white solid after preparative RP-HPLC (59 mg, 22 %). **Analytical RP-HPLC**: t_R = 3.02 min (A/D 100:0 to 0:100 in 7.0 min, λ = 214 nm). **MS** (ESI$^+$): $C_{56}H_{97}N_{17}O_{10}$ calc./obs. 1167.7604/1167.7646 Da [M].

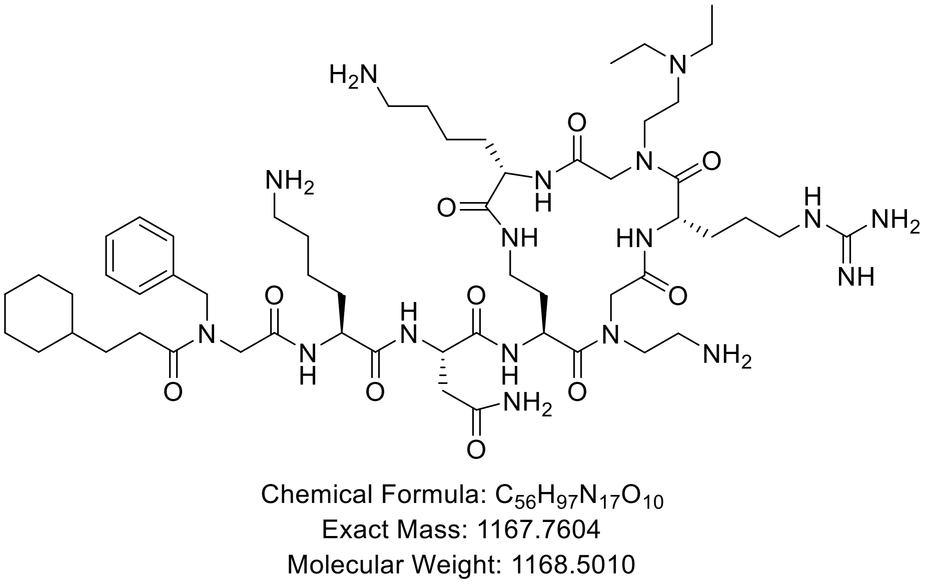

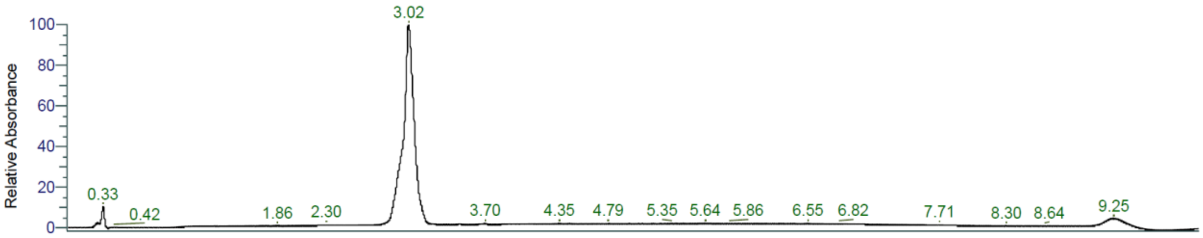

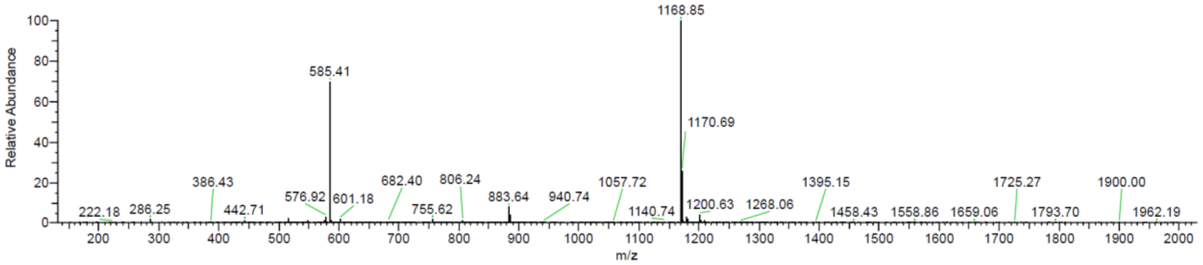

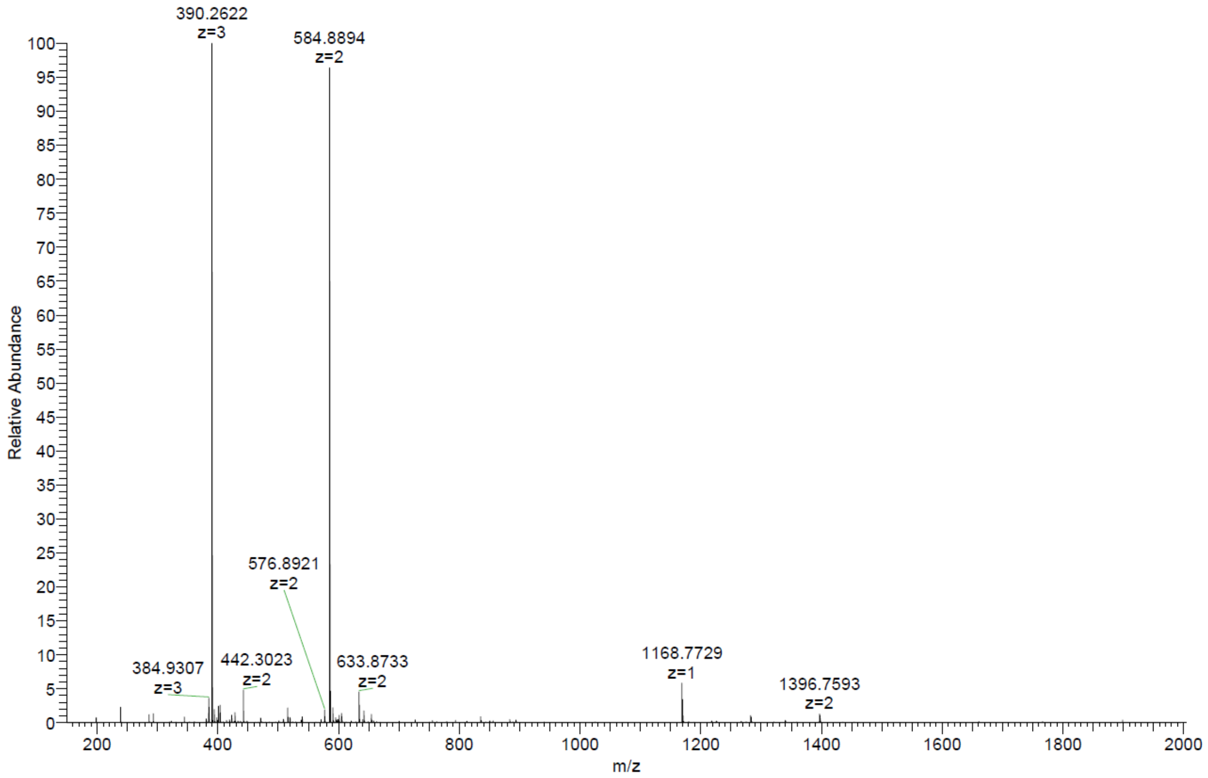
390.2622
z=3
584.8894
z=2
576.8921
z=2
384.9307
z=3
442.3023
z=2
633.8733
z=2
1168.7729
z=1
1396.7593
z=2
Relative Abundance
m/z

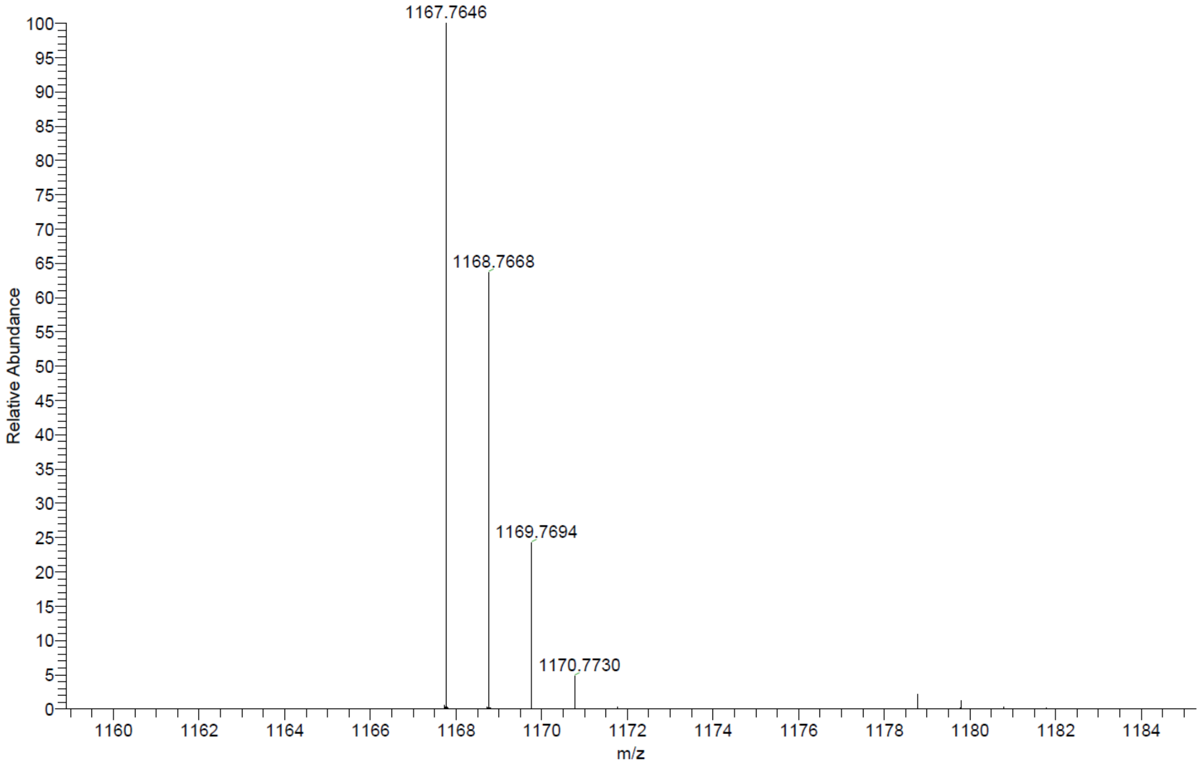
1167.7646
1168.7668
1169.7694
1170.7730
Relative Abundance
m/z

EB16 was obtained as a foamy white solid after preparative RP-HPLC (24 mg, 11 %). **Analytical RP-HPLC**: t_R = 2.34 min (A/D 100:0 to 0:100 in 7.0 min, λ = 214 nm). **MS** (ESI$^+$): $C_{53}H_{94}N_{16}O_{10}$ calc./obs. 1114.7339/1114.7364 Da [M].

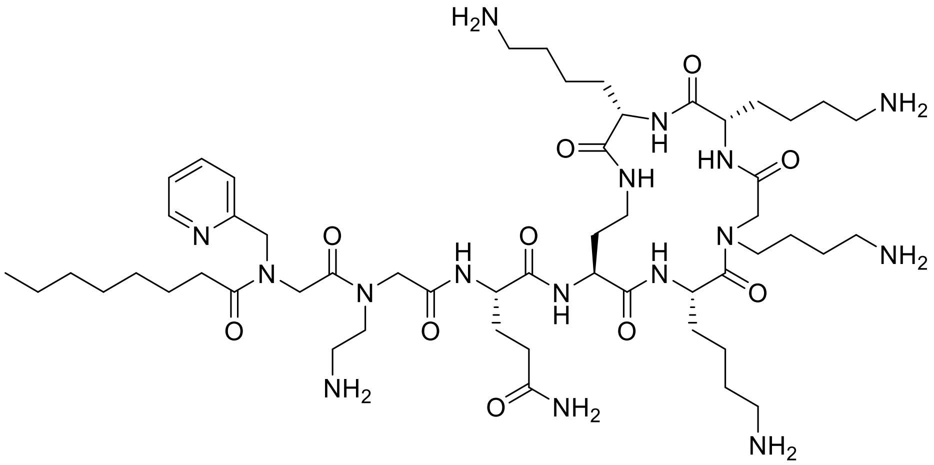

Chemical Formula: $C_{53}H_{94}N_{16}O_{10}$
Exact Mass: 1114.7339
Molecular Weight: 1115.4370

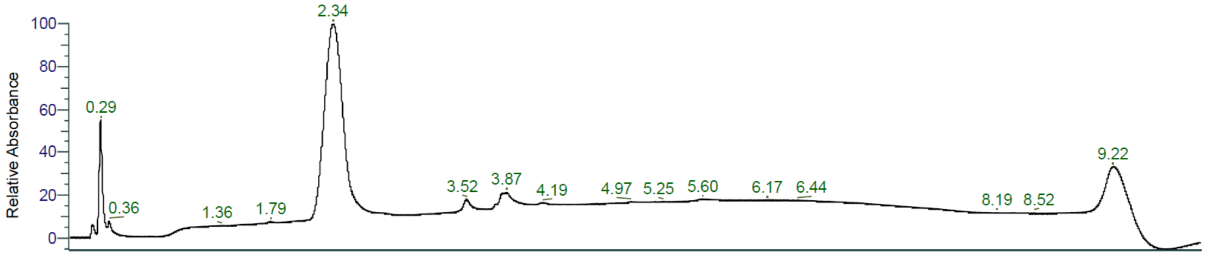

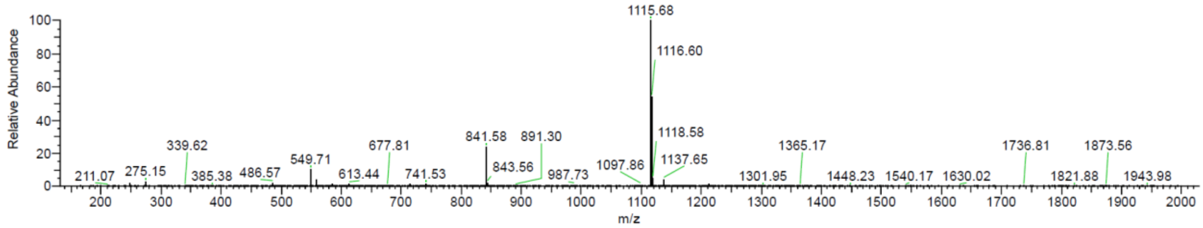

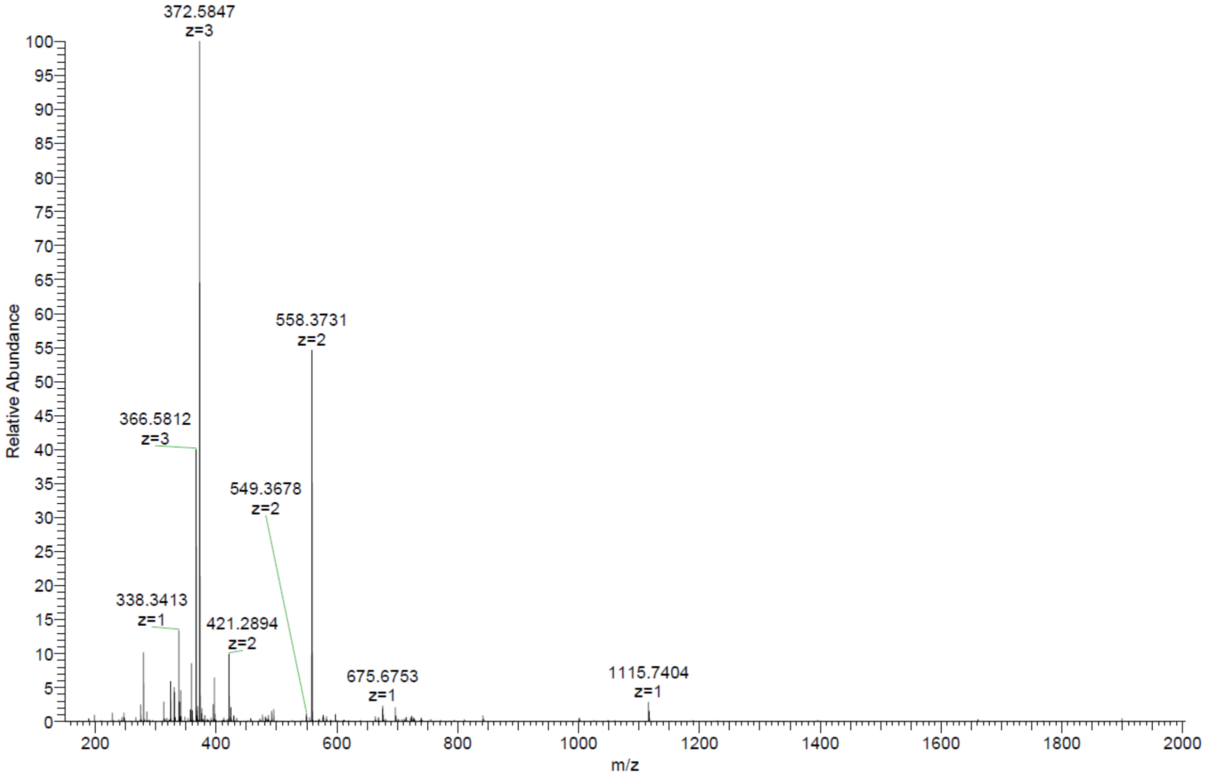
372.5847
z=3
558.3731
z=2
366.5812
z=3
549.3678
z=2
338.3413
z=1
421.2894
z=2
675.6753
z=1
1115.7404
z=1
Relative Abundance
m/z

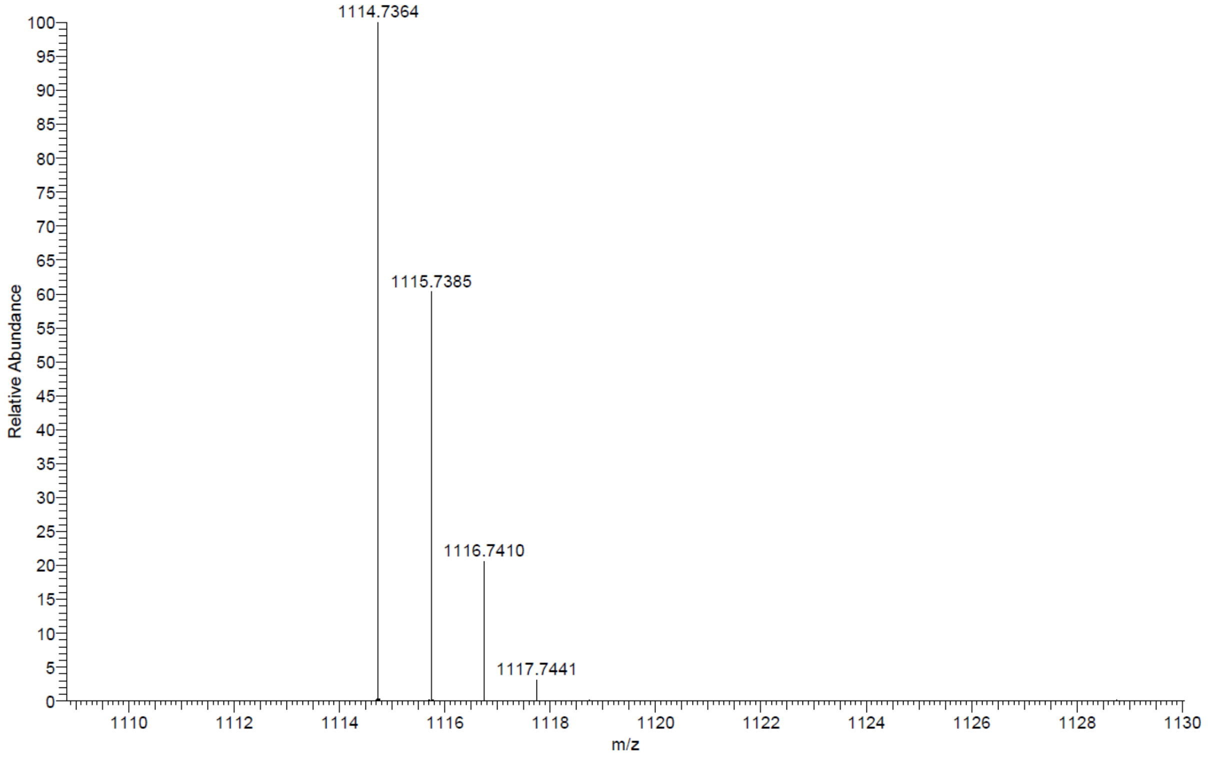
1114.7364
1115.7385
1116.7410
1117.7441
Relative Abundance
m/z

EB17 was obtained as a foamy white solid after preparative RP-HPLC (10 mg, 4 %). **Analytical RP-HPLC**: t_R = 2.83 min (A/D 100:0 to 0:100 in 7.0 min, λ = 214 nm). **MS** (ESI$^+$): $C_{61}H_{107}N_{17}O_{11}$ calc./obs. 1253.8336/1253.8312 Da [M].

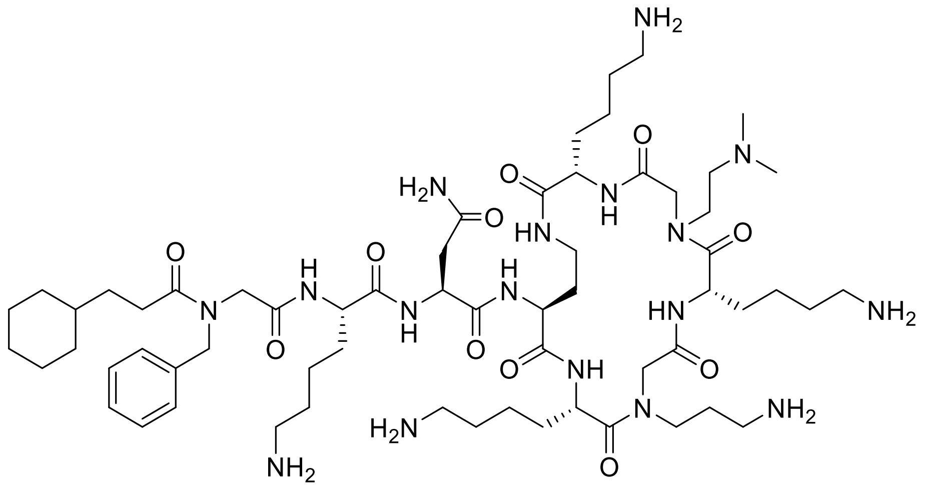

Chemical Formula: $C_{61}H_{107}N_{17}O_{11}$
Exact Mass: 1253.8336
Molecular Weight: 1254.6350

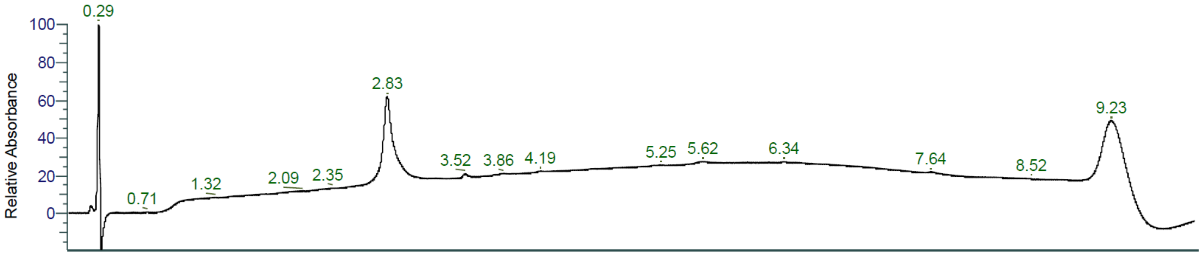

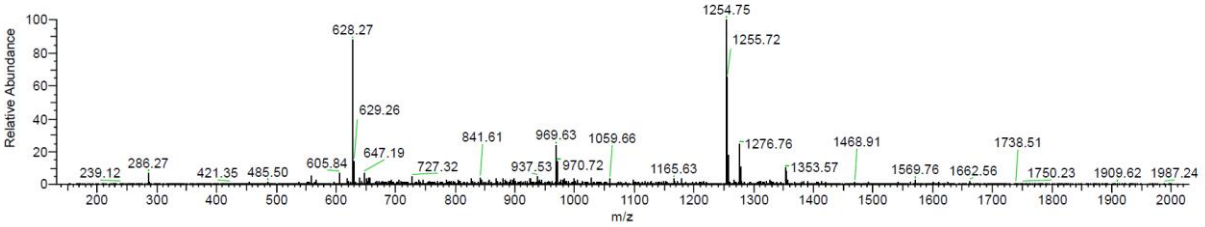

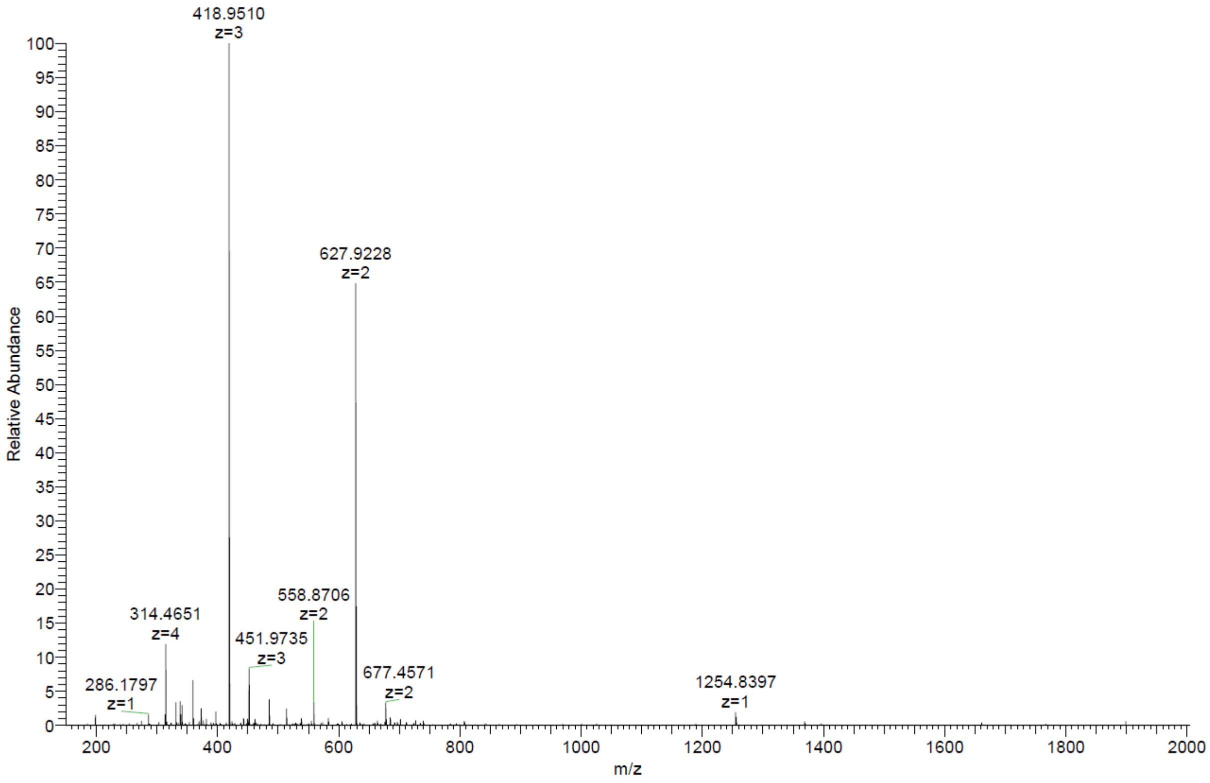
418.9510
z=3
627.9228
z=2
558.8706
z=2
314.4651
z=4
451.9735
z=3
286.1797
z=1
677.4571
z=2
1254.8397
z=1
Relative Abundance
m/z

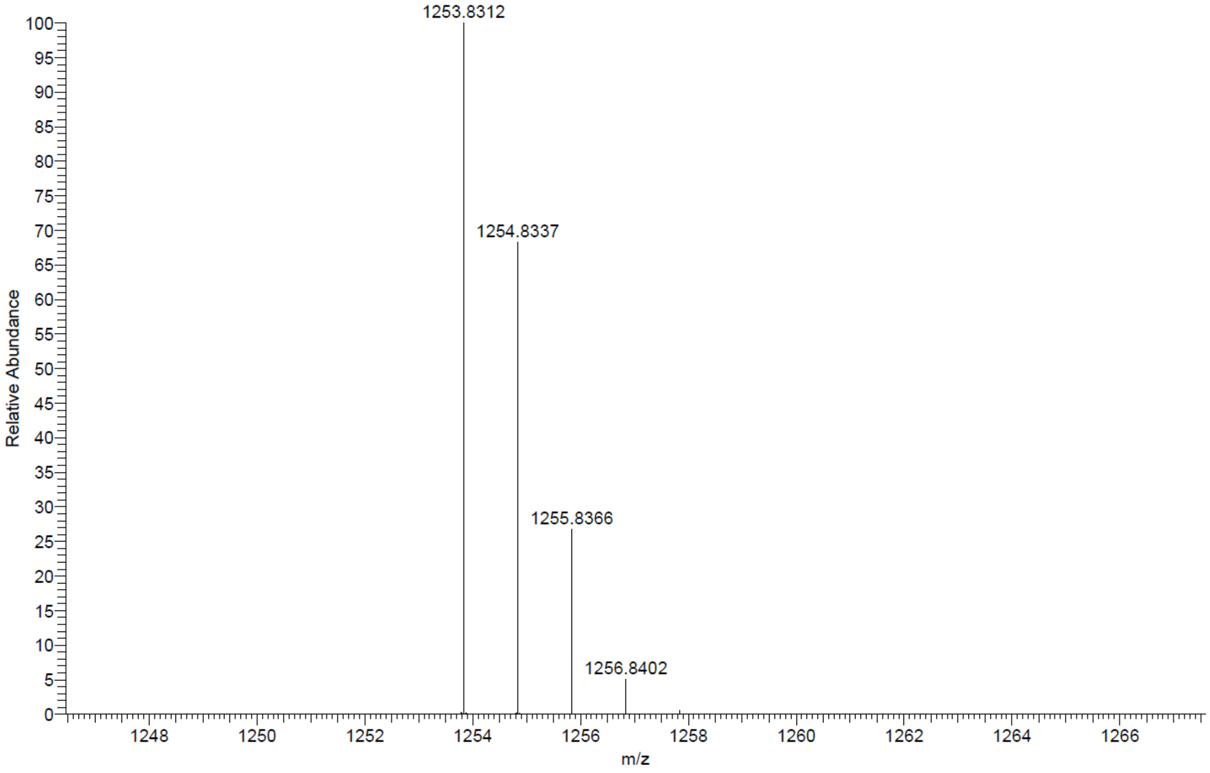
1253.8312
1254.8337
1255.8366
1256.8402
Relative Abundance
m/z

EB18 was obtained as a foamy white solid after preparative RP-HPLC (64 mg, 22 %). **Analytical RP-HPLC**: t_R = 2.87 min (A/D 100:0 to 0:100 in 7.0 min, λ = 214 nm). **MS** (ESI$^+$): $C_{57}H_{98}N_{16}O_{11}$ calc./obs. 1182.7601/1182.7606 Da [M].

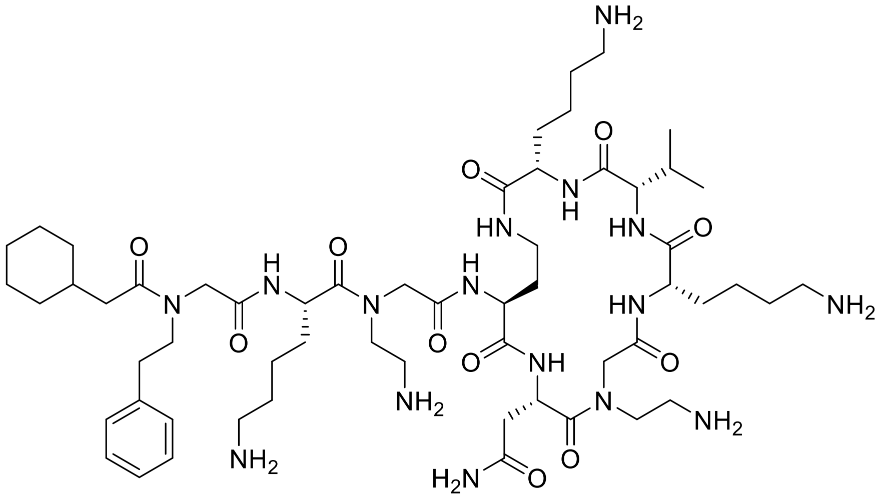

Chemical Formula: $C_{57}H_{98}N_{16}O_{11}$
Exact Mass: 1182.7601
Molecular Weight: 1183.5120

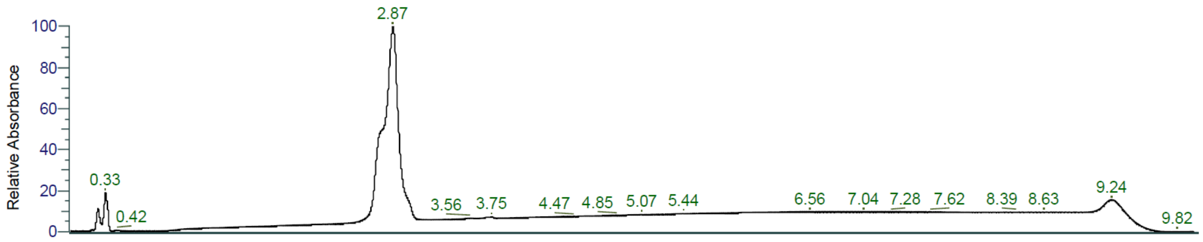

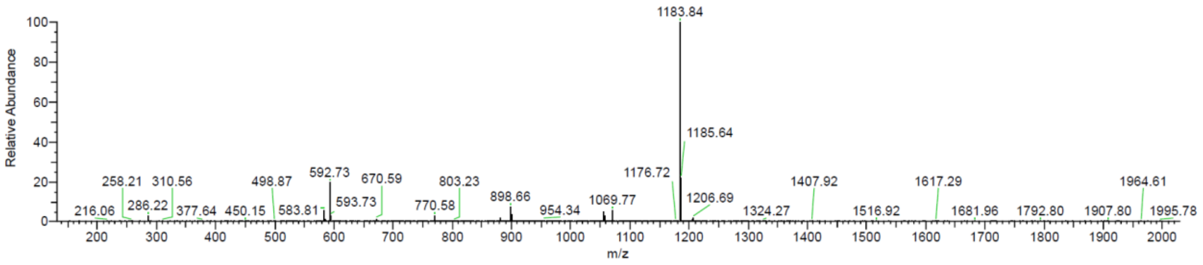

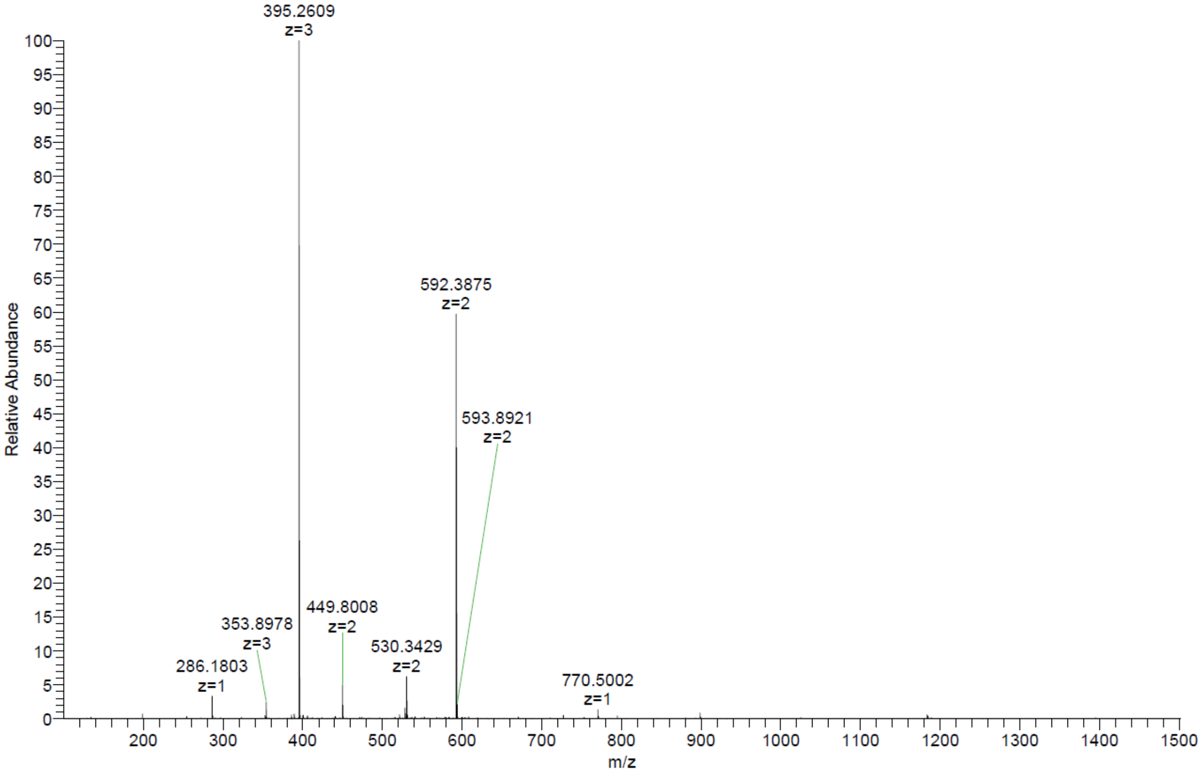
395.2609
z=3
592.3875
z=2
593.8921
z=2
353.8978
z=3
449.8008
z=2
530.3429
z=2
286.1803
z=1
770.5002
z=1
Relative Abundance
m/z

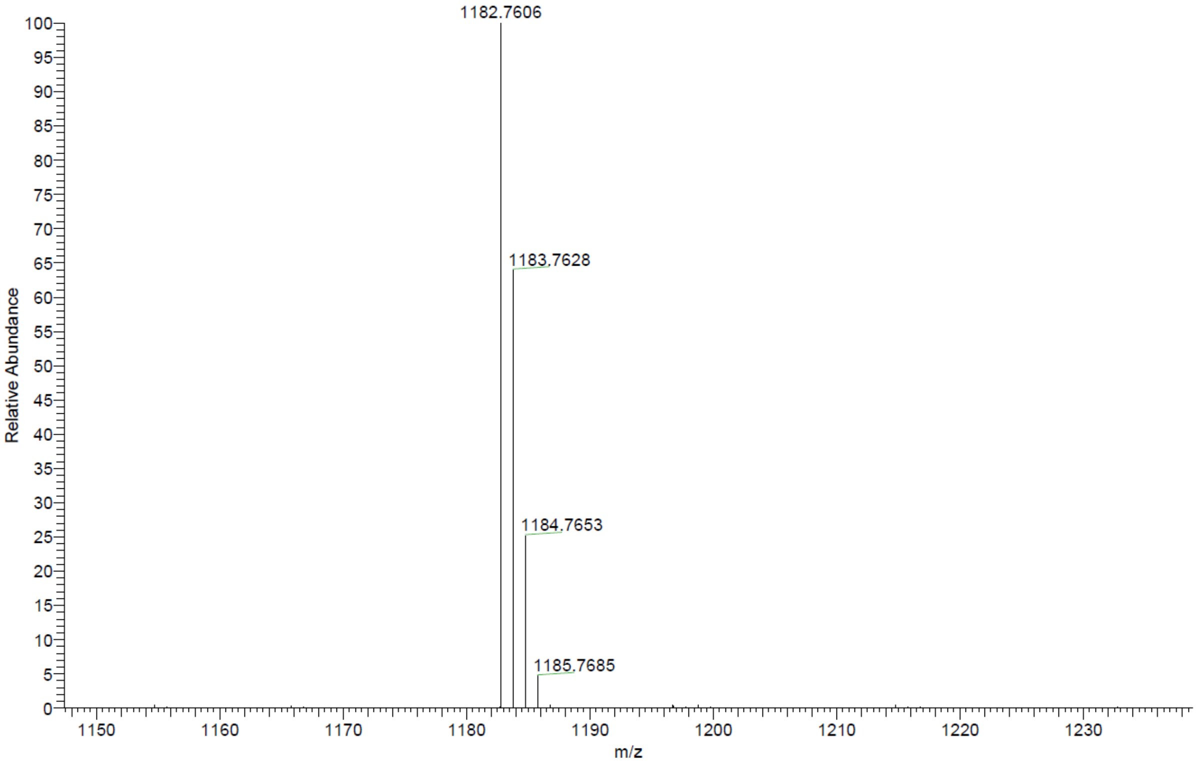
1182.7606
1183.7628
1184.7653
1185.7685
Relative Abundance
m/z

EB19 was obtained as a foamy white solid after preparative RP-HPLC (32 mg, 10 %). **Analytical RP-HPLC**: t_R = 2.50 min (A/D 100:0 to 0:100 in 7.0 min, λ = 214 nm). **MS** (ESI$^+$): $C_{57}H_{101}N_{19}O_{11}$ calc./obs. 1227.7928/1227.7967 Da [M].

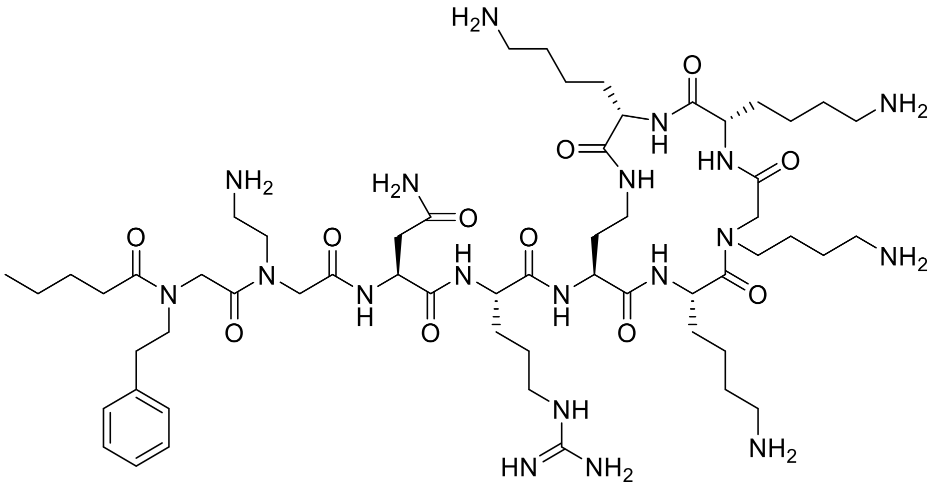

Chemical Formula: $C_{57}H_{101}N_{19}O_{11}$
Exact Mass: 1227.7928
Molecular Weight: 1228.5570

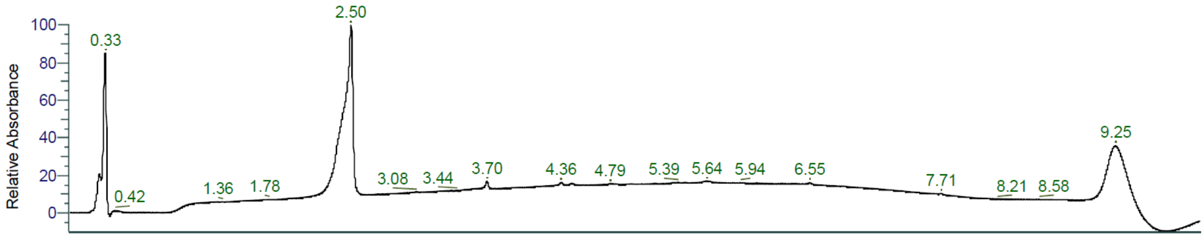

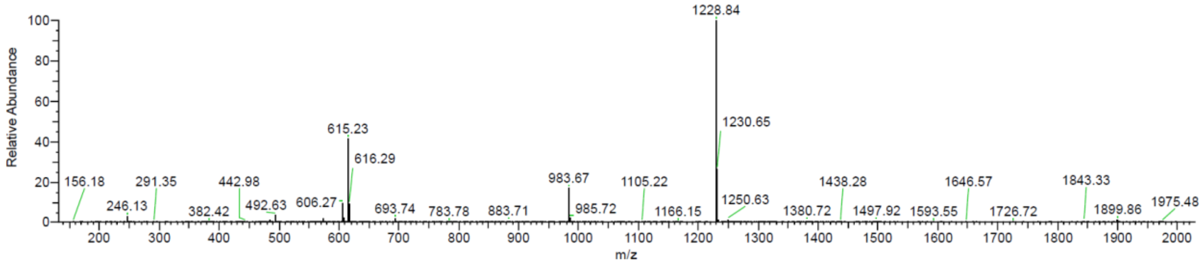

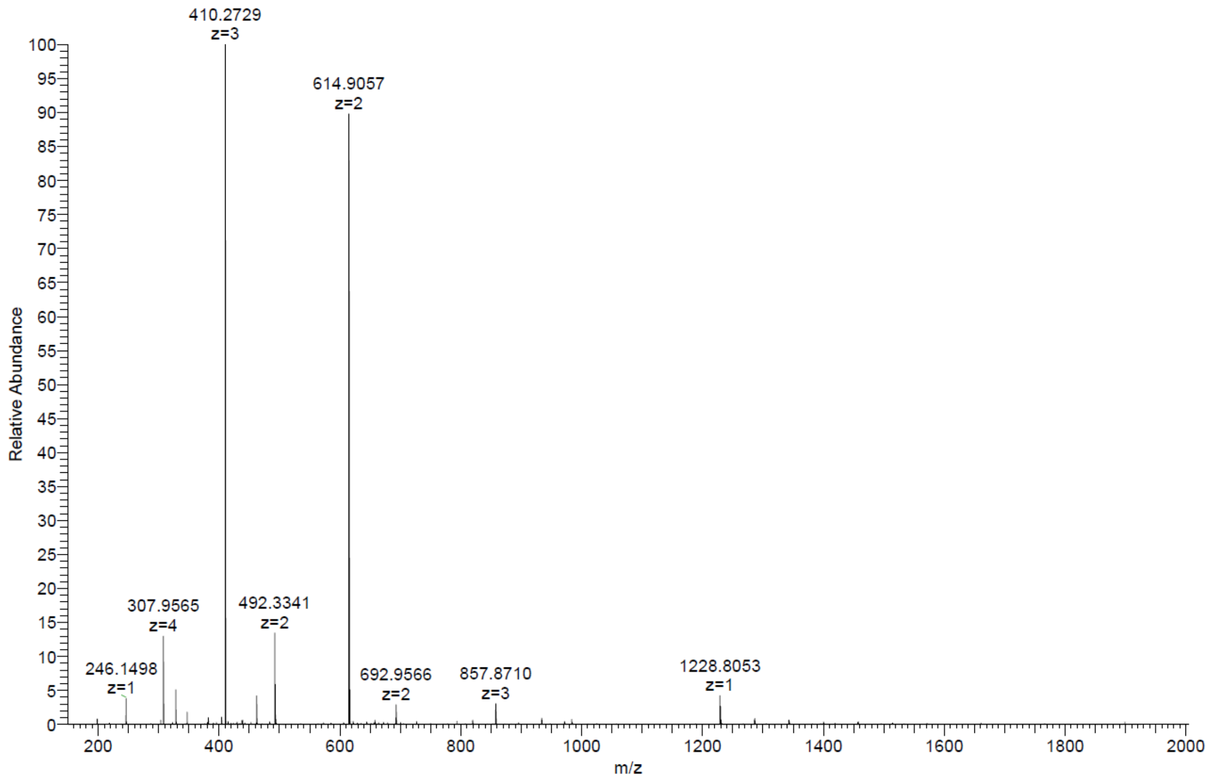
410.2729
z=3
614.9057
z=2
307.9565
z=4
492.3341
z=2
246.1498
z=1
692.9566
z=2
857.8710
z=3
1228.8053
z=1
Relative Abundance
m/z

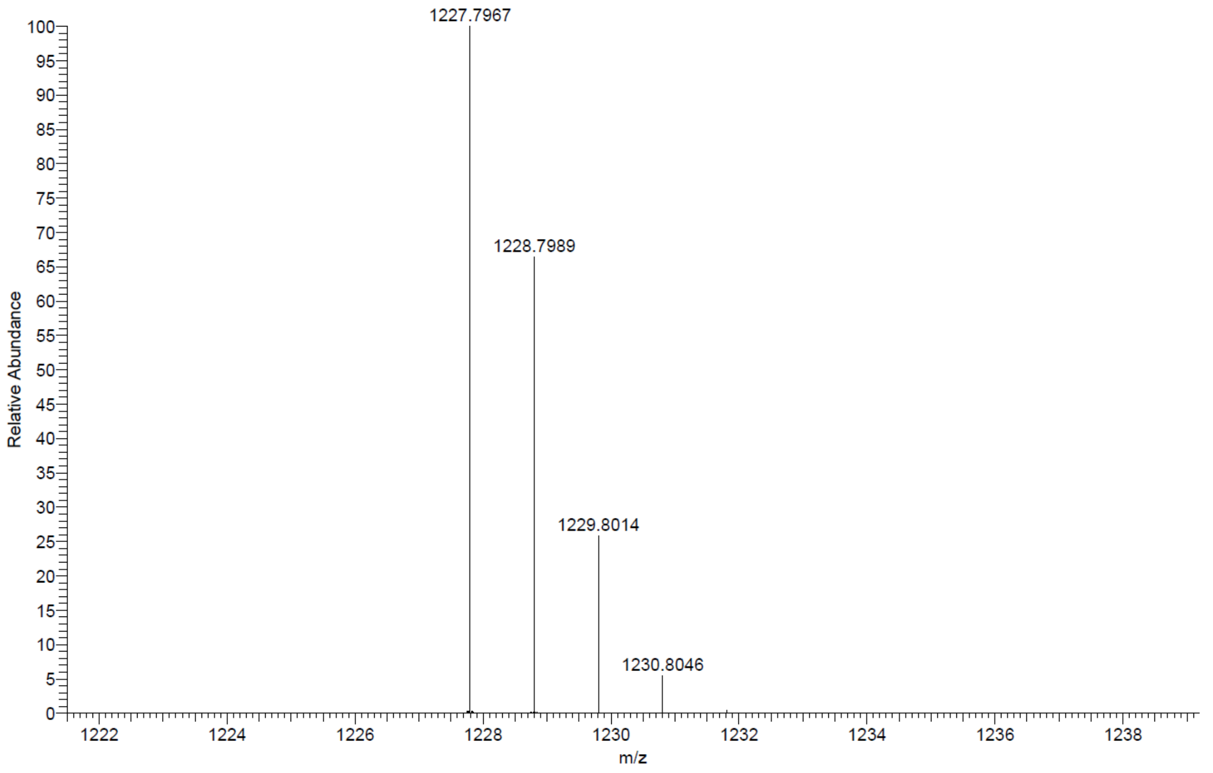
1227.7967
1228.7989
1229.8014
1230.8046
Relative Abundance
m/z

EB20 was obtained as a foamy white solid after preparative RP-HPLC (94 mg, 28 %).

Analytical RP-HPLC: t_R = 2.68 min (A/D 100:0 to 0:100 in 7.0 min, λ = 214 nm). **MS** (ESI$^+$): $C_{56}H_{96}N_{16}O_{11}$ calc./obs. 1168.7444/1168.7455 Da [M].

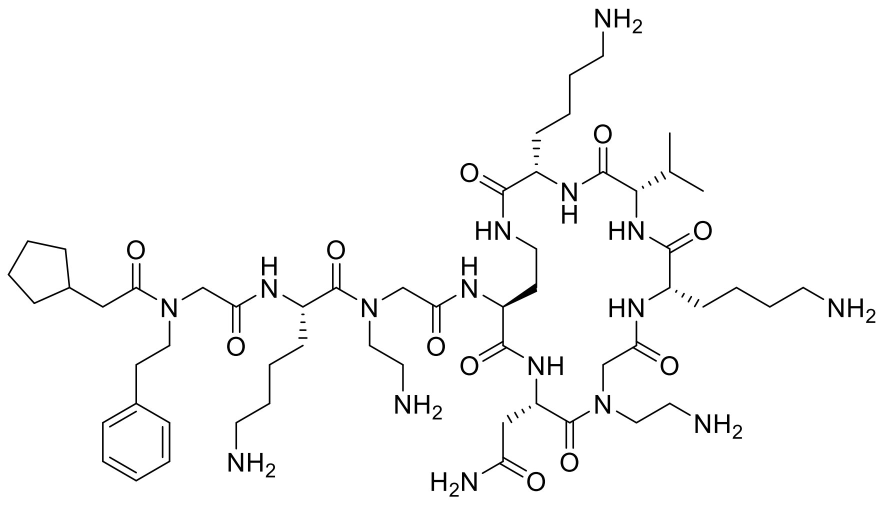

Chemical Formula: $C_{56}H_{96}N_{16}O_{11}$
Exact Mass: 1168.7444
Molecular Weight: 1169.4850

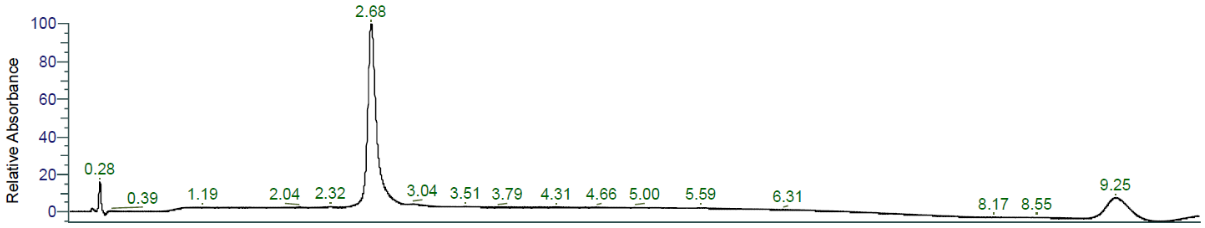

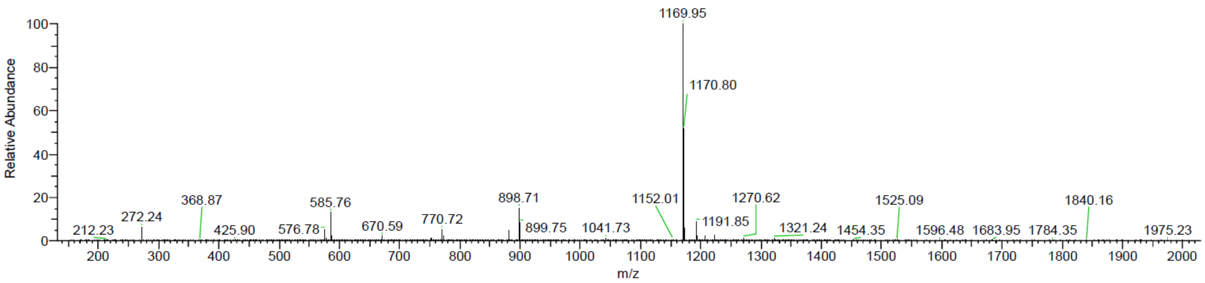

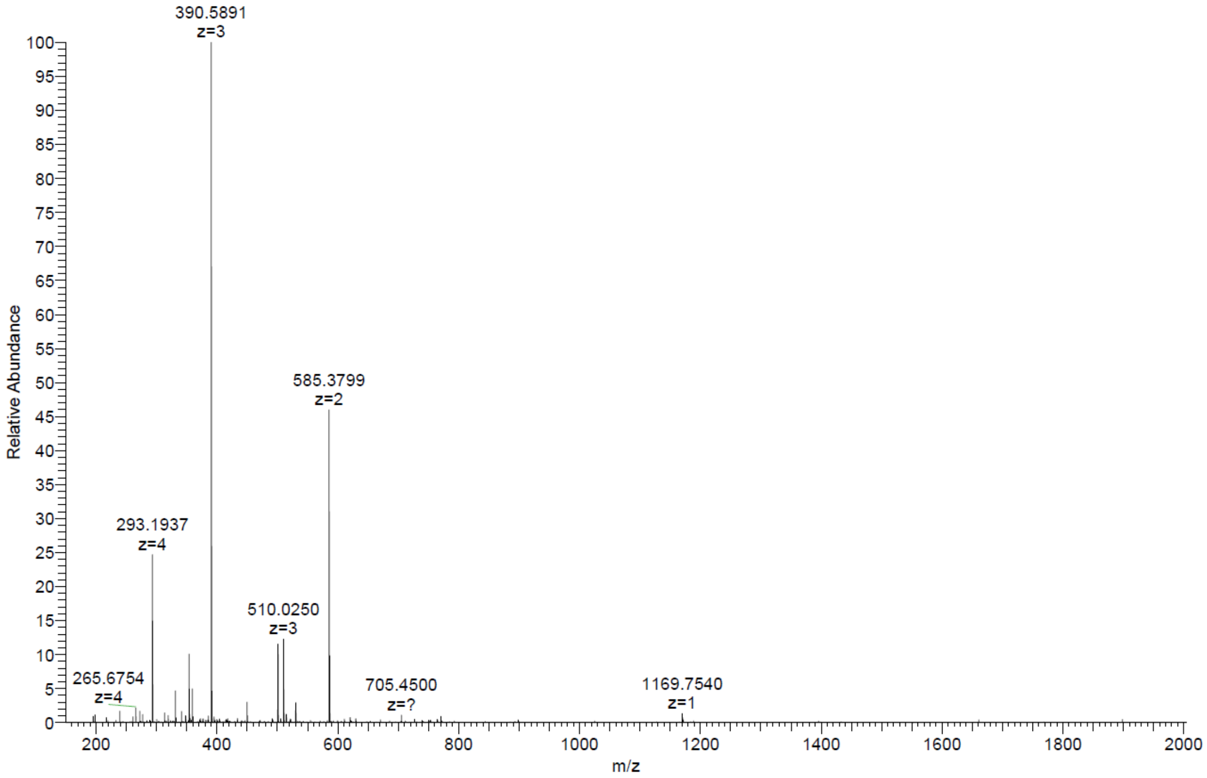
390.5891
z=3
585.3799
z=2
293.1937
z=4
510.0250
z=3
265.6754
z=4
705.4500
z=?
1169.7540
z=1
Relative Abundance
m/z

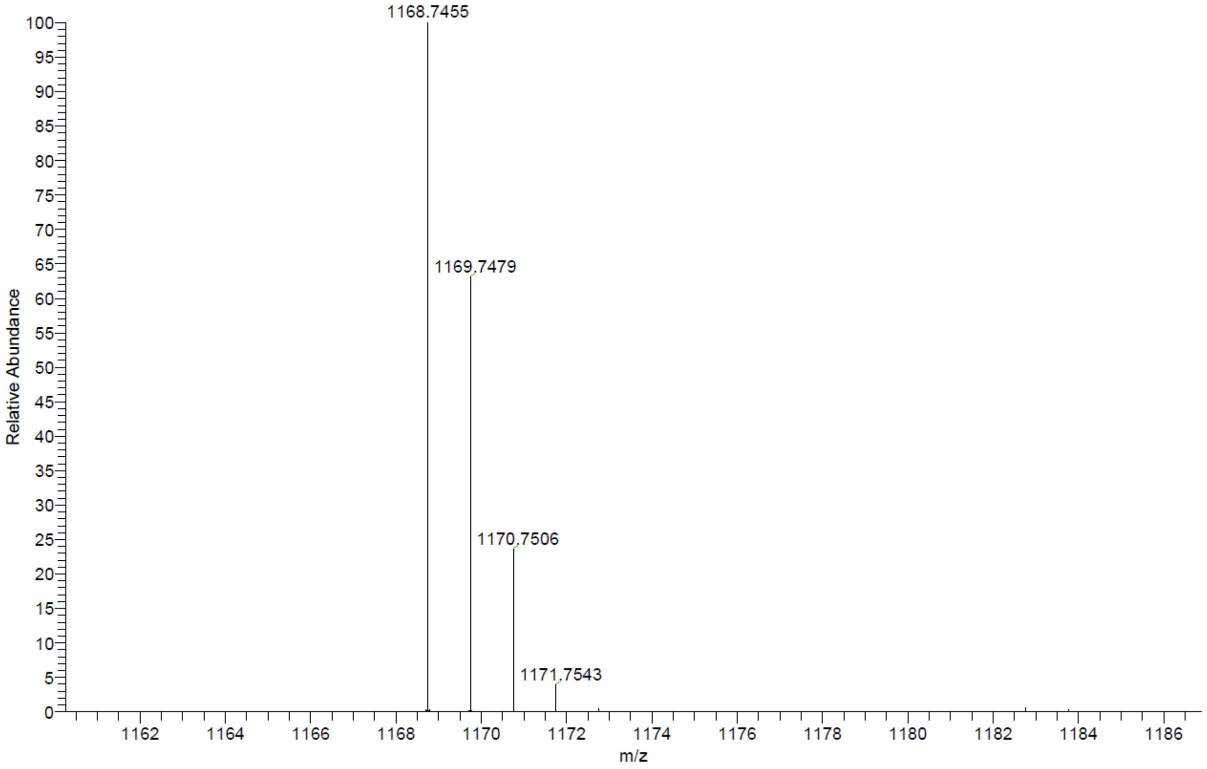
1168.7455
1169.7479
1170.7506
1171.7543
Relative Abundance
m/z

EB21 was obtained as a foamy white solid after preparative RP-HPLC (39 mg, 11 %). **Analytical RP-HPLC**: t_R = 2.72 min (A/D 100:0 to 0:100 in 7.0 min, λ = 214 nm). **MS** (ESI$^+$): $C_{60}H_{104}N_{16}O_{11}$ calc./obs. 1224.8070/1224.8059 Da [M].

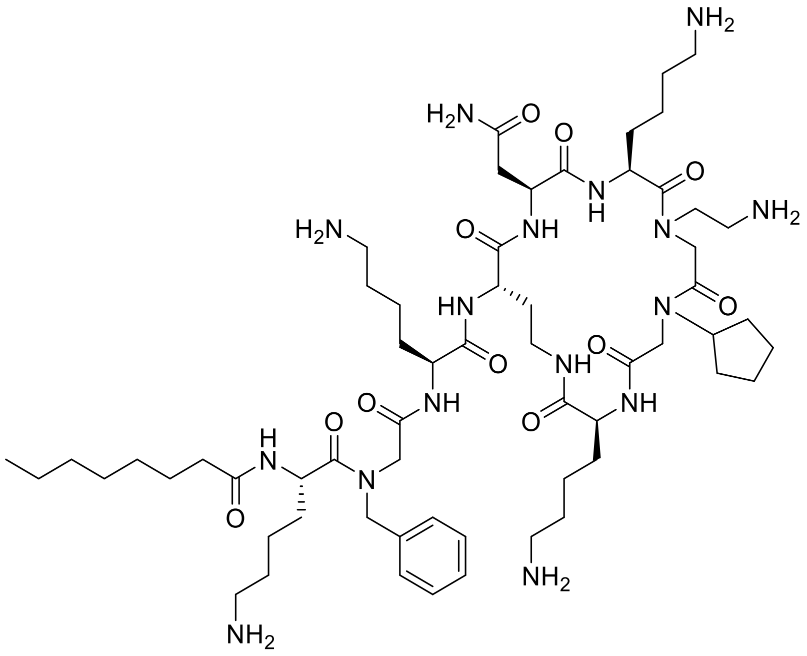

Chemical Formula: $C_{60}H_{104}N_{16}O_{11}$
Exact Mass: 1224.8070
Molecular Weight: 1225.5930

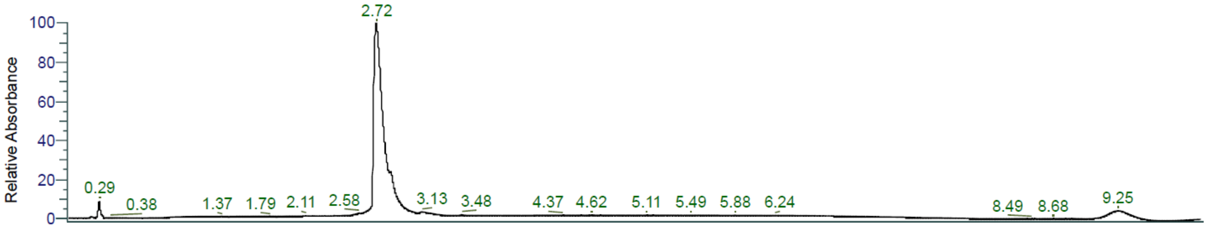

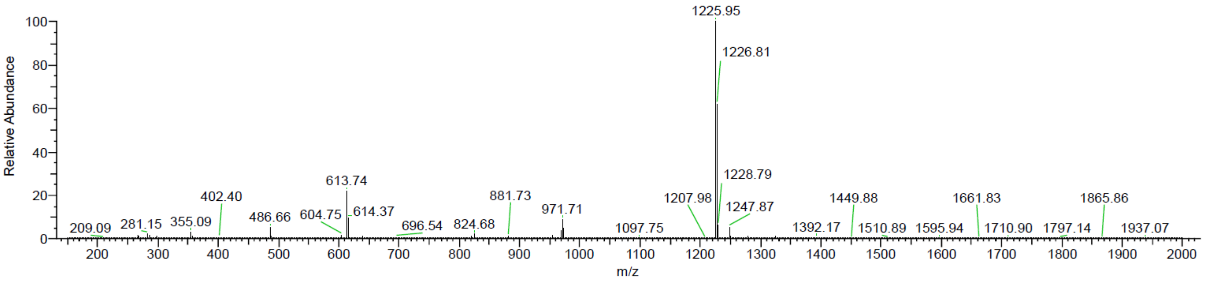

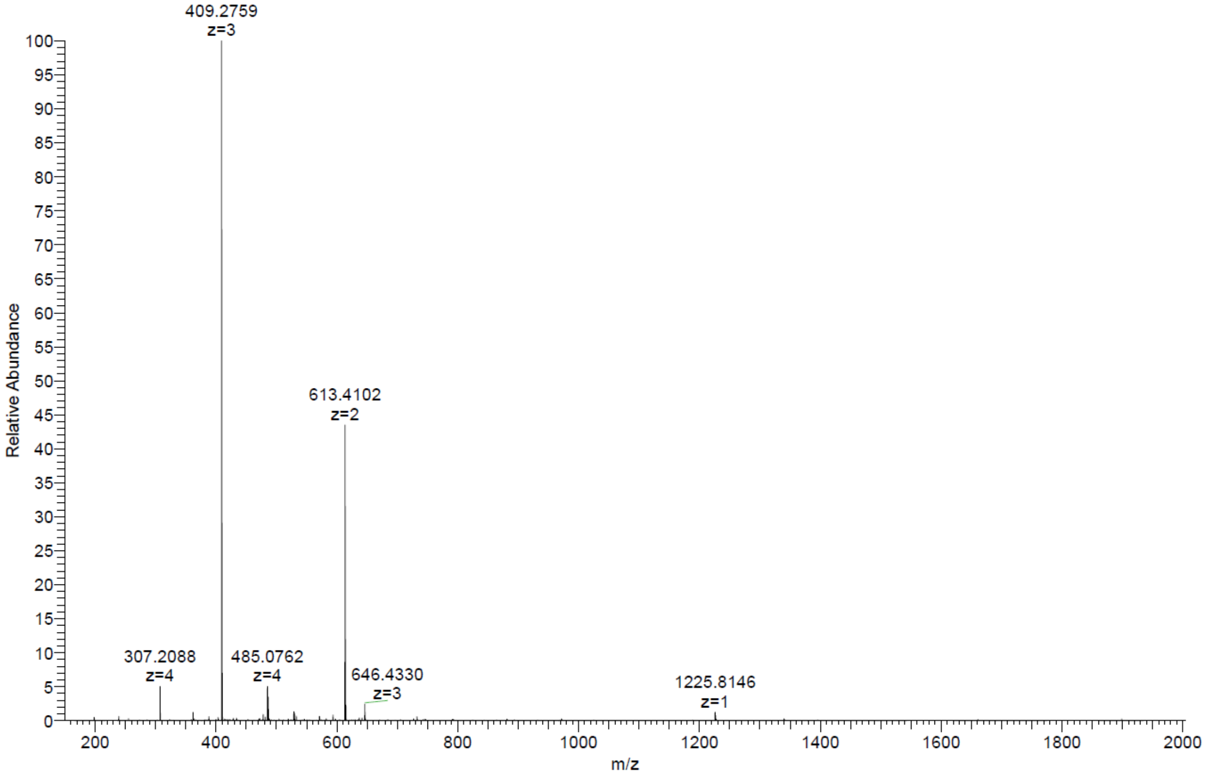
409.2759
z=3
613.4102
z=2
307.2088
z=4
485.0762
z=4
646.4330
z=3
1225.8146
z=1
Relative Abundance
m/z

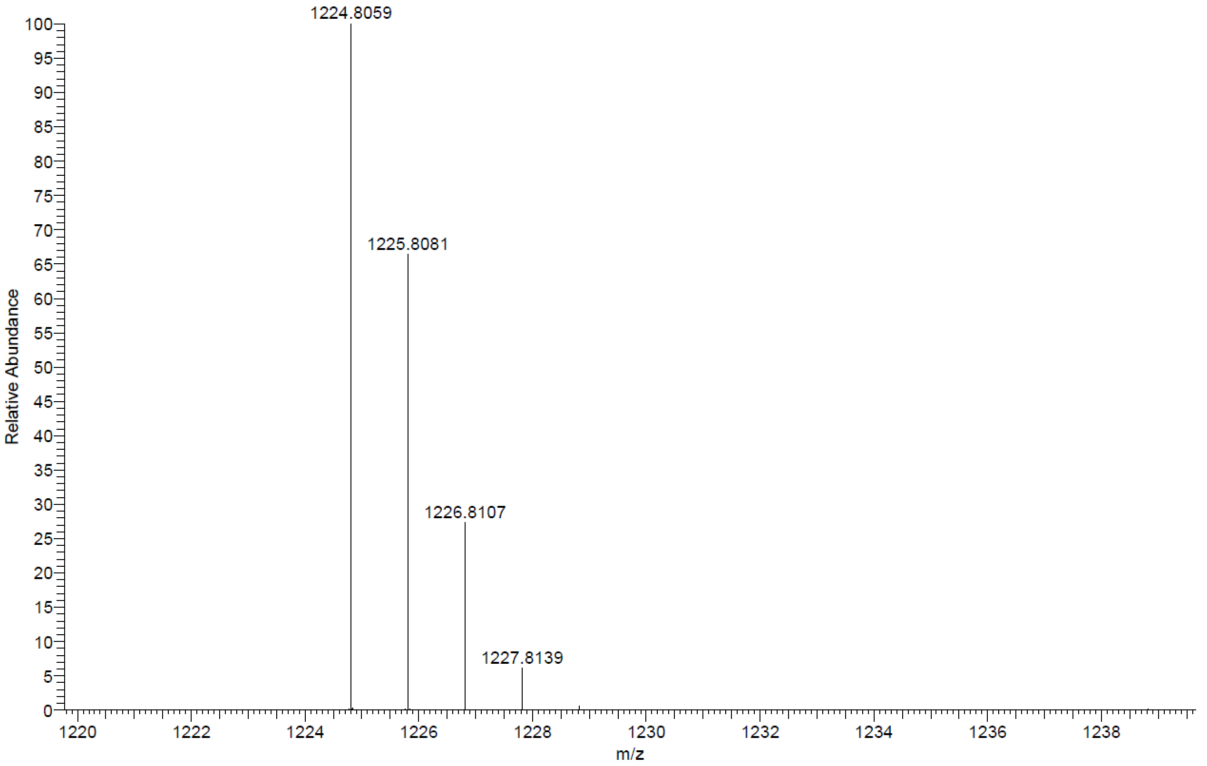
1224.8059
1225.8081
1226.8107
1227.8139
Relative Abundance
m/z

EB22 was obtained as a foamy white solid after preparative RP-HPLC (85 mg, 22 %). **Analytical RP-HPLC**: t_R = 2.81 min (A/D 100:0 to 0:100 in 7.0 min, λ = 214 nm). **MS** (ESI$^+$): $C_{62}H_{110}N_{18}O_{12}$ calc./obs. 1298.8551/1298.8549 Da [M].

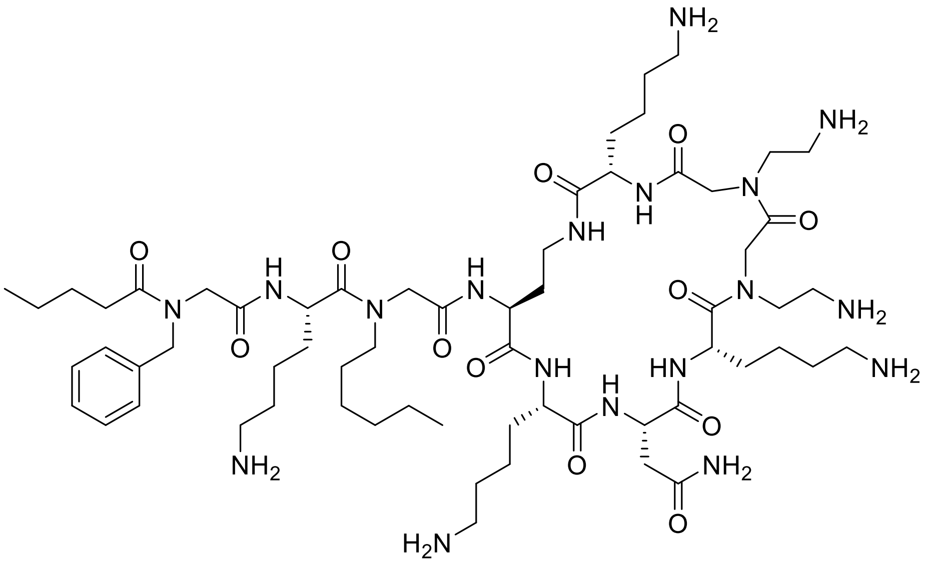

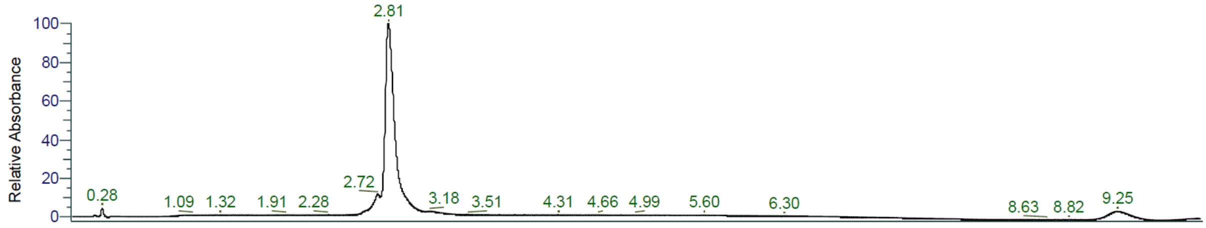

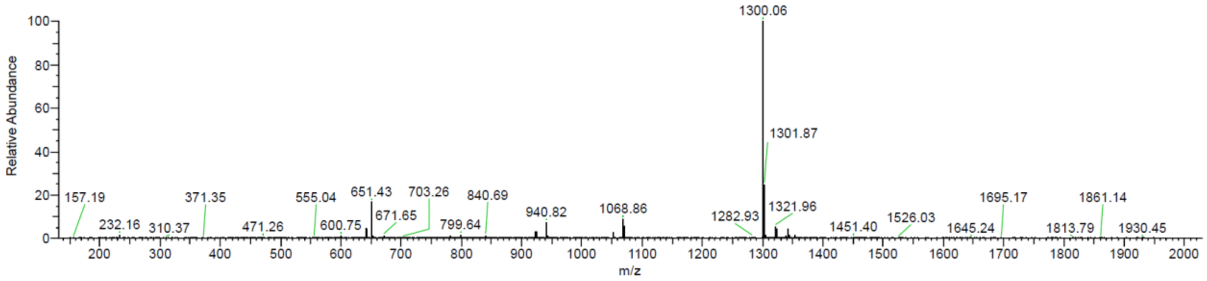

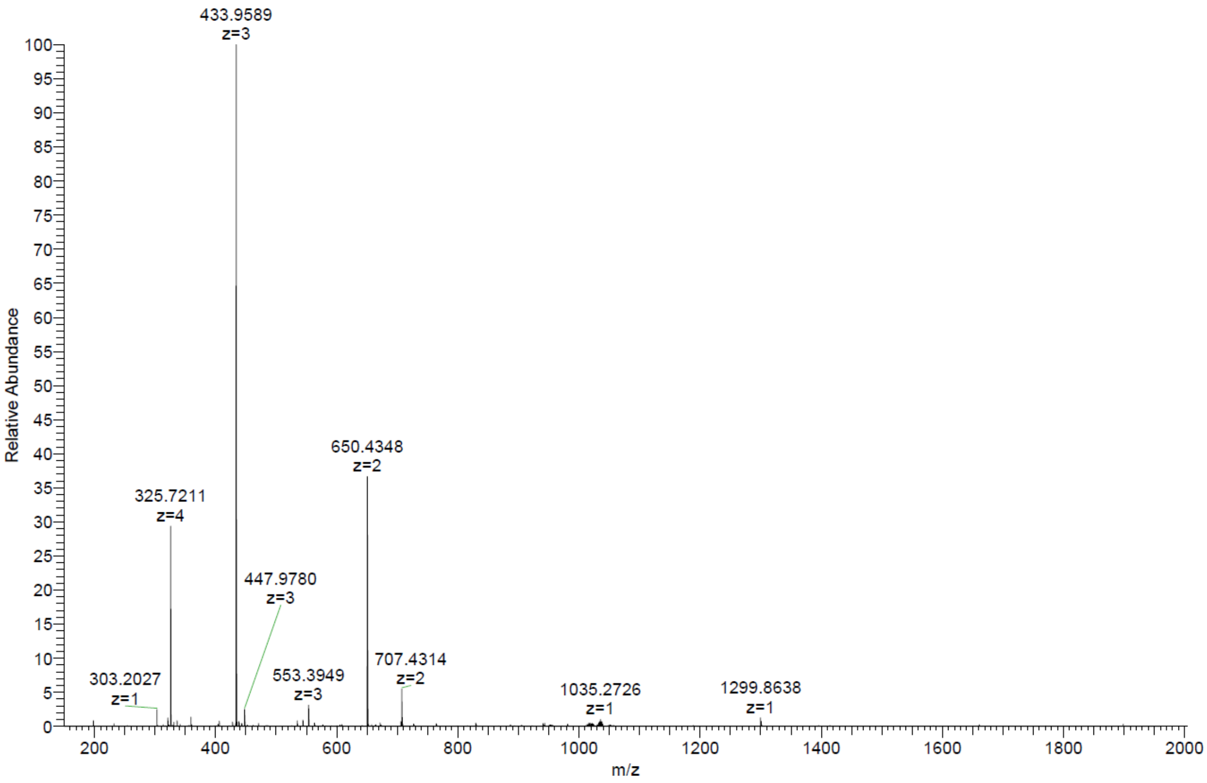
433.9589
z=3
650.4348
z=2
325.7211
z=4
447.9780
z=3
707.4314
z=2
553.3949
z=3
303.2027
z=1
1035.2726
z=1
1299.8638
z=1
Relative Abundance
m/z

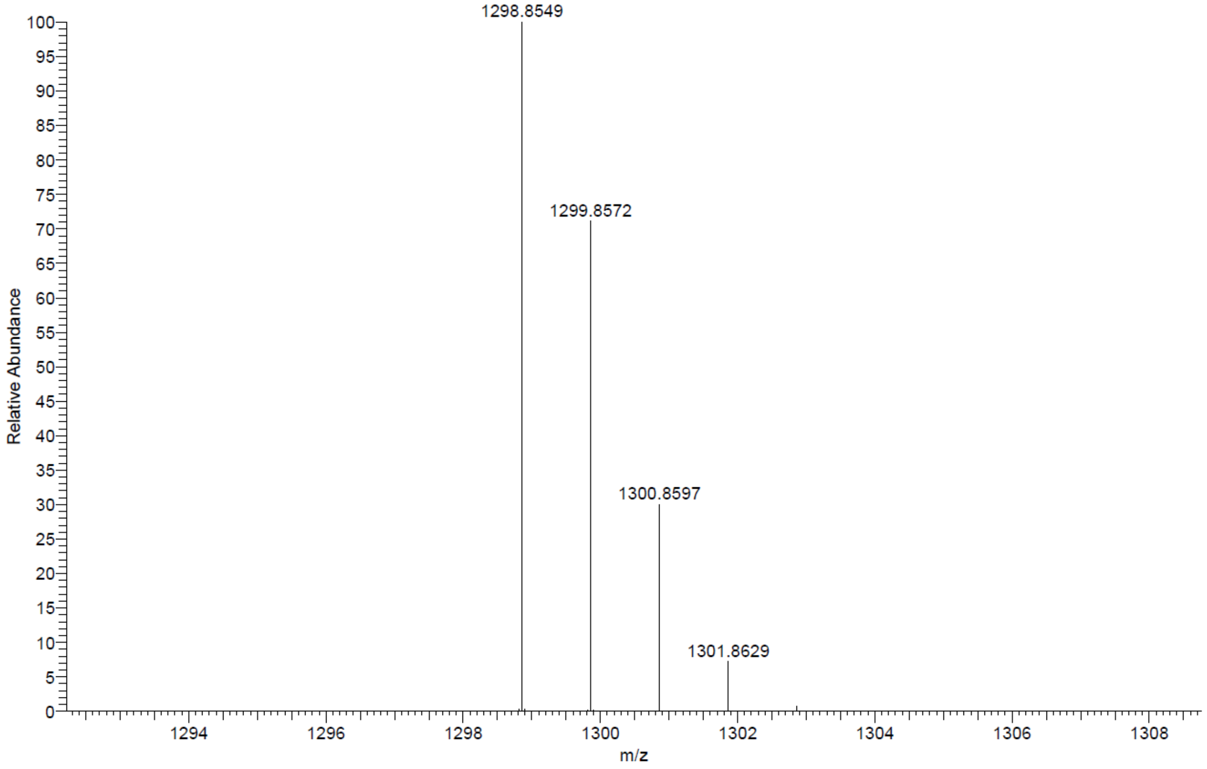
1298.8549
1299.8572
1300.8597
1301.8629
Relative Abundance
m/z

EB23 was obtained as a foamy white solid after preparative RP-HPLC (10 mg, 3 %). **Analytical RP-HPLC**: t_R = 2.86 min (A/D 100:0 to 0:100 in 7.0 min, λ = 214 nm). **MS** (ESI$^+$): $C_{58}H_{100}N_{18}O_{11}$ calc./obs. 1224.7819/1224.7826 Da [M].

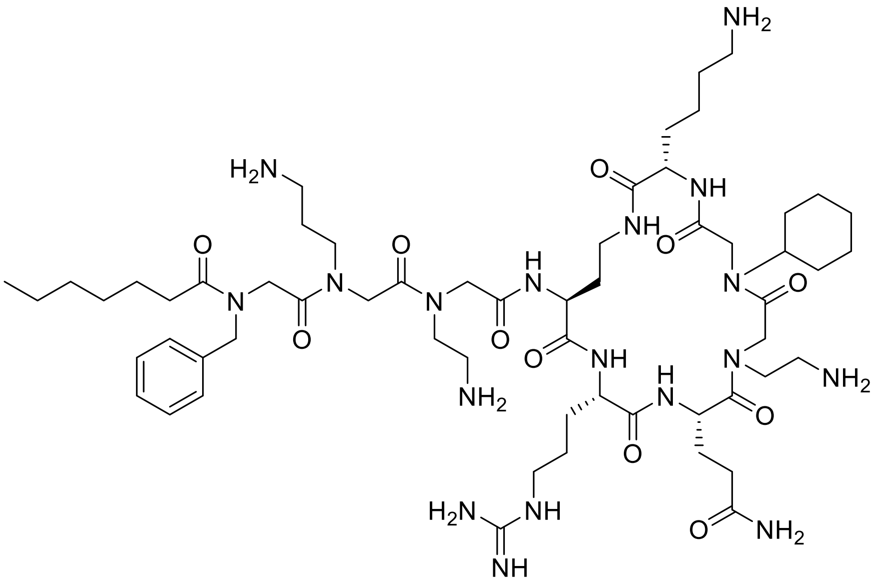

Chemical Formula: $C_{58}H_{100}N_{18}O_{11}$
Exact Mass: 1224.7819
Molecular Weight: 1225.5530

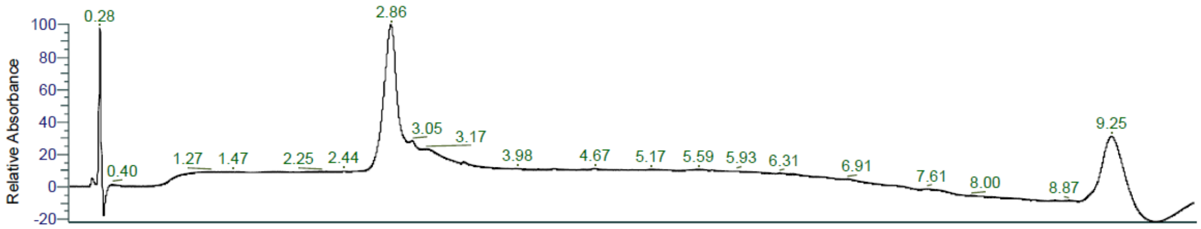

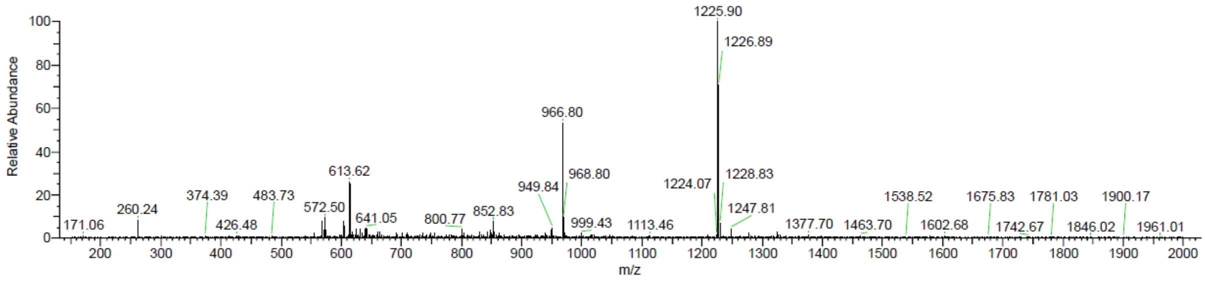

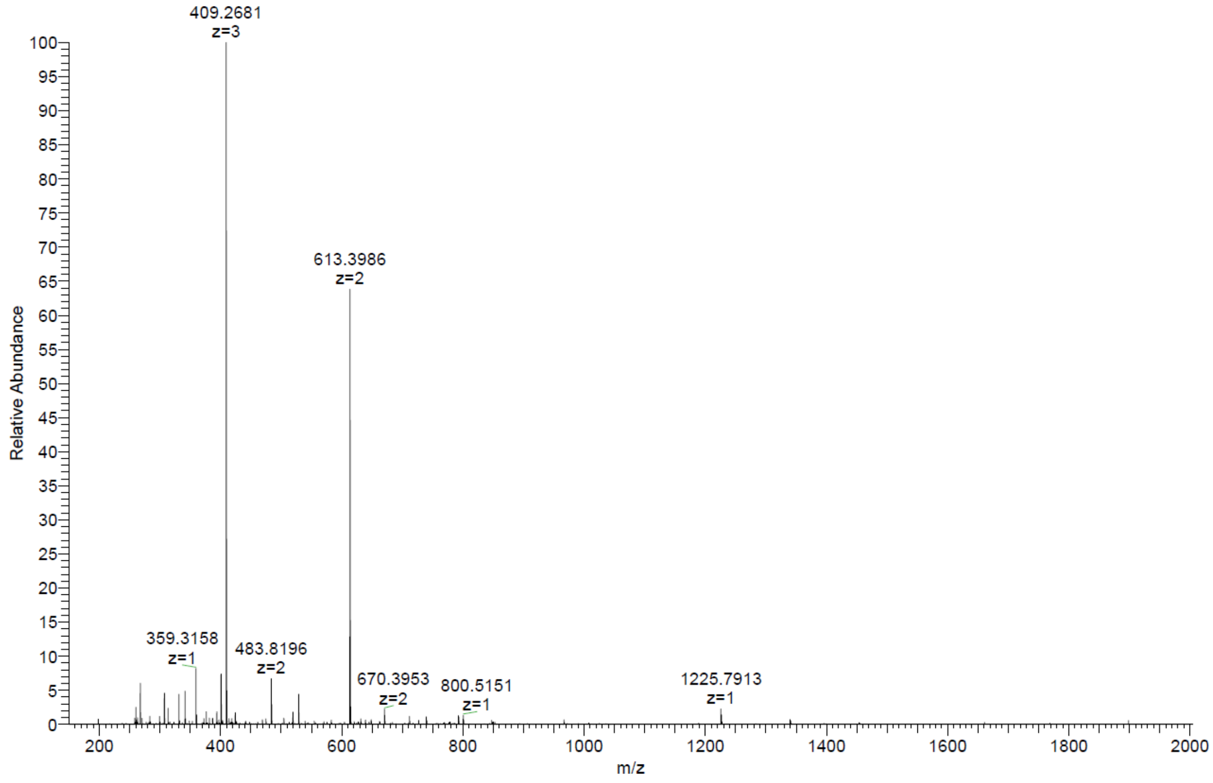
409.2681
z=3
613.3986
z=2
359.3158
z=1
483.8196
z=2
670.3953
z=2
800.5151
z=1
1225.7913
z=1
Relative Abundance
m/z

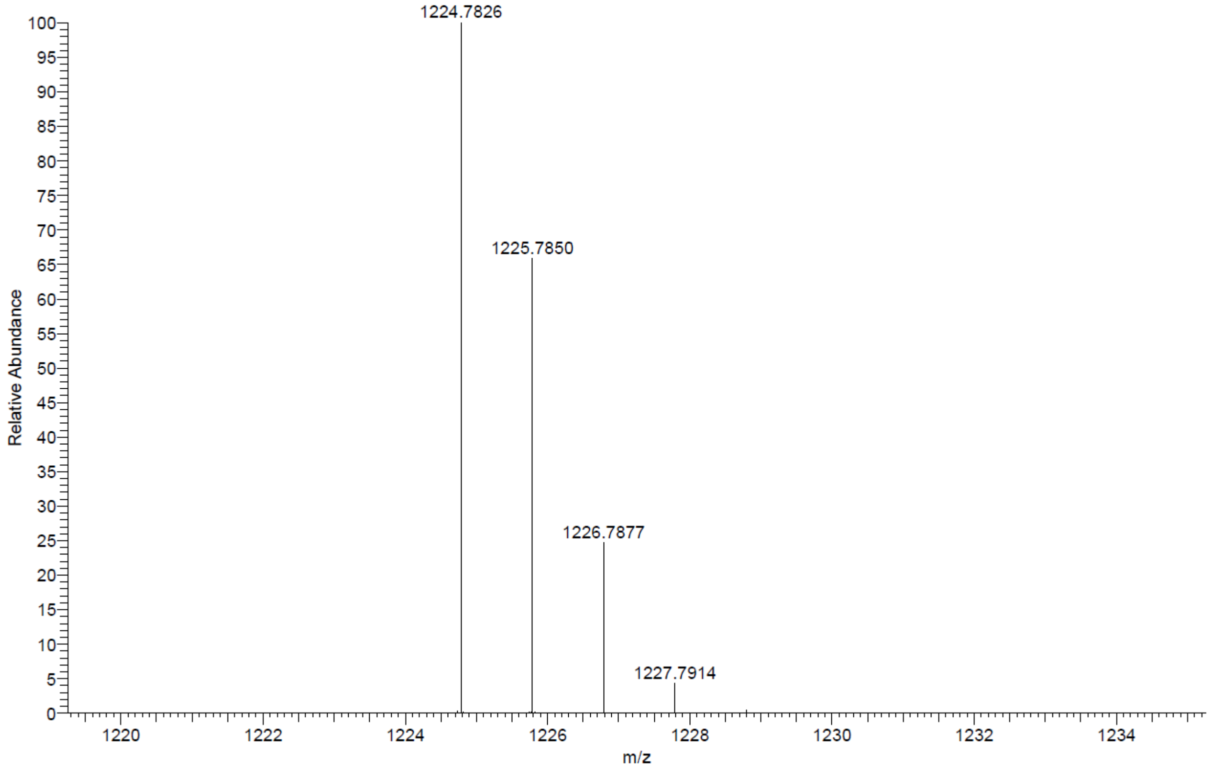
1224.7826
1225.7850
1226.7877
1227.7914
Relative Abundance
m/z

EB24 was obtained as a foamy white solid after preparative RP-HPLC (38 mg, 16 %). **Analytical RP-HPLC**: t_R = 2.50 min (A/D 100:0 to 0:100 in 7.0 min, λ = 214 nm). **MS** (ESI$^+$): $C_{60}H_{108}N_{16}O_{11}$ calc./obs. 1228.8383/1228.8360 Da [M].

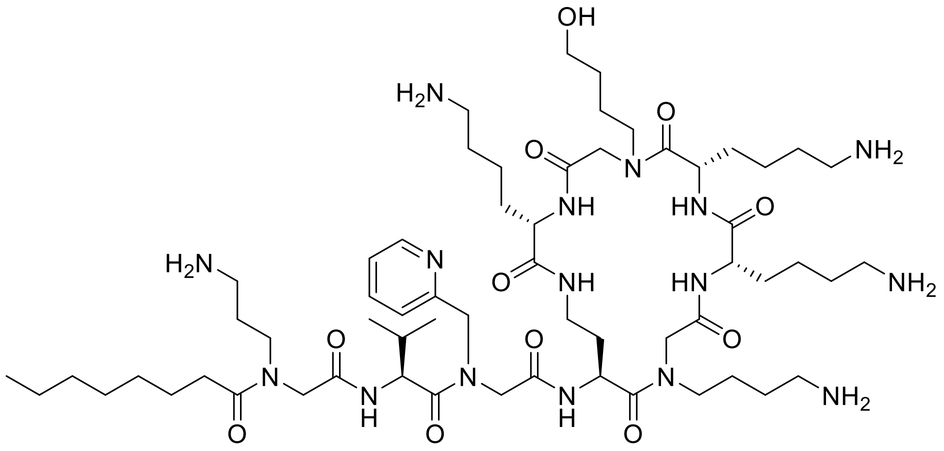

Chemical Formula: $C_{60}H_{108}N_{16}O_{11}$
Exact Mass: 1228.8383
Molecular Weight: 1229.6250

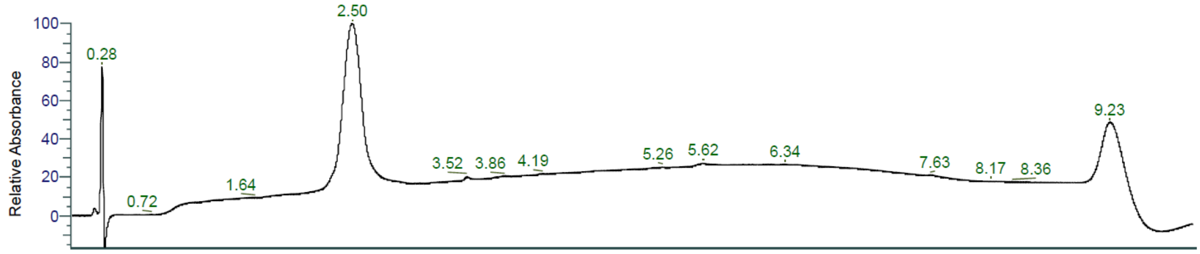

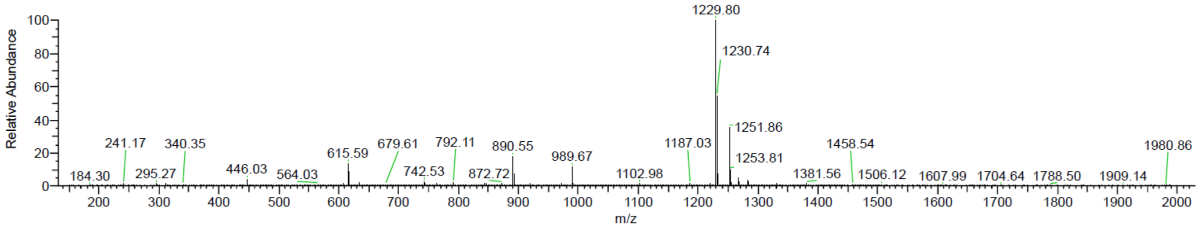

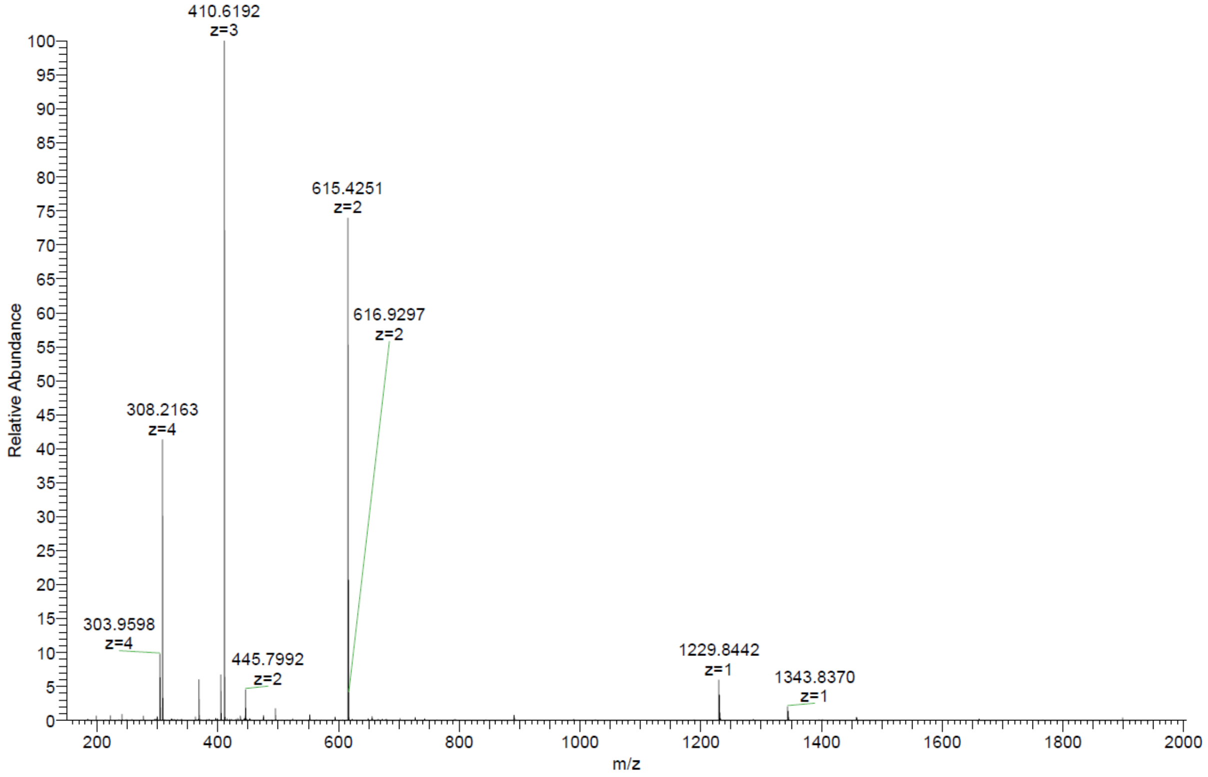
410.6192
z=3
615.4251
z=2
616.9297
z=2
308.2163
z=4
303.9598
z=4
445.7992
z=2
1229.8442
z=1
1343.8370
z=1
Relative Abundance
m/z

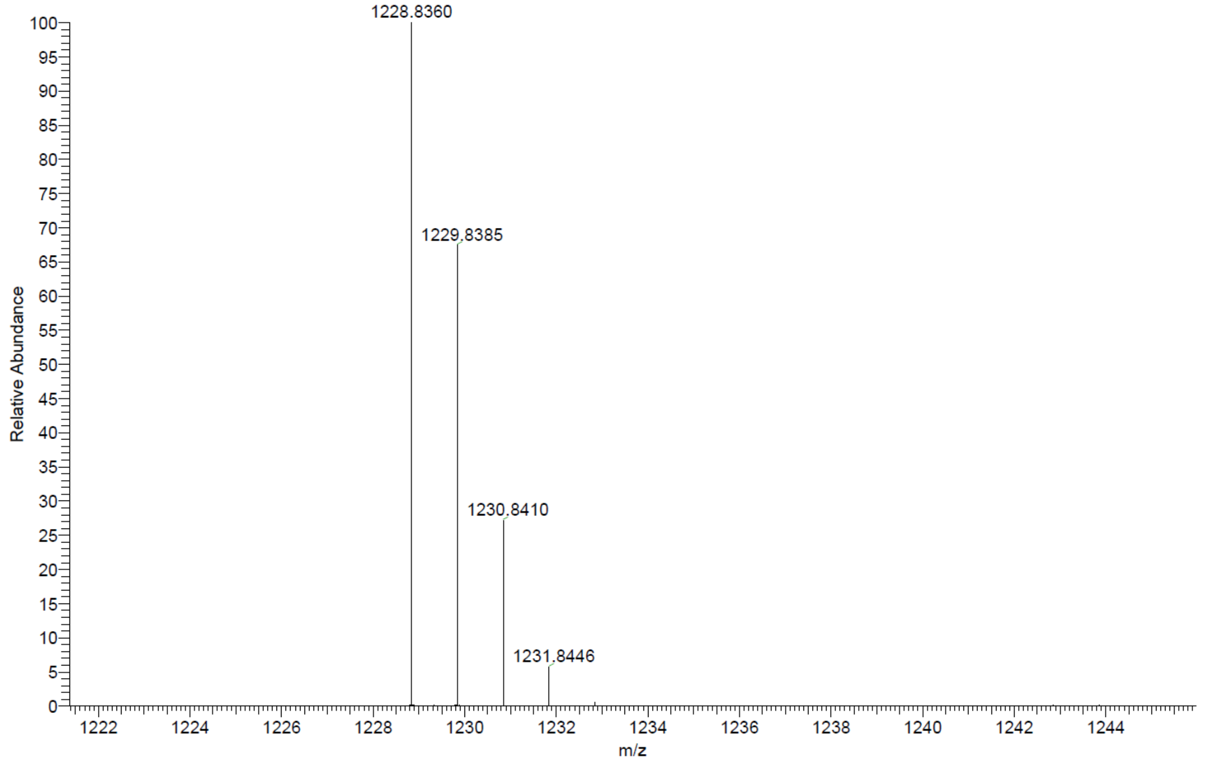
1228.8360
1229.8385
1230.8410
1231.8446
Relative Abundance
m/z

EB25 was obtained as a foamy white solid after preparative RP-HPLC (11 mg, 5 %). **Analytical RP-HPLC**: t_R = 2.69 min (A/D 100:0 to 0:100 in 7.0 min, λ = 214 nm). **MS** (ESI$^+$): $C_{55}H_{94}N_{16}O_{11}$ calc./obs. 1154.7288/1154.7265 Da [M].

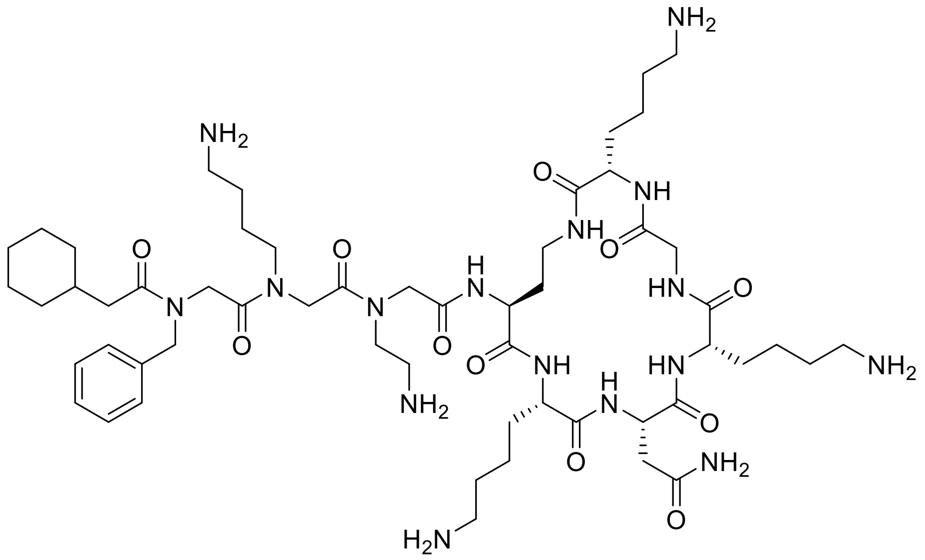

Chemical Formula: $C_{55}H_{94}N_{16}O_{11}$
Exact Mass: 1154.7288
Molecular Weight: 1155.4580

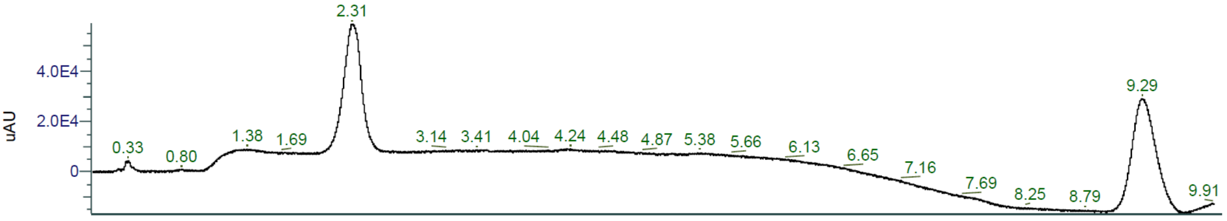

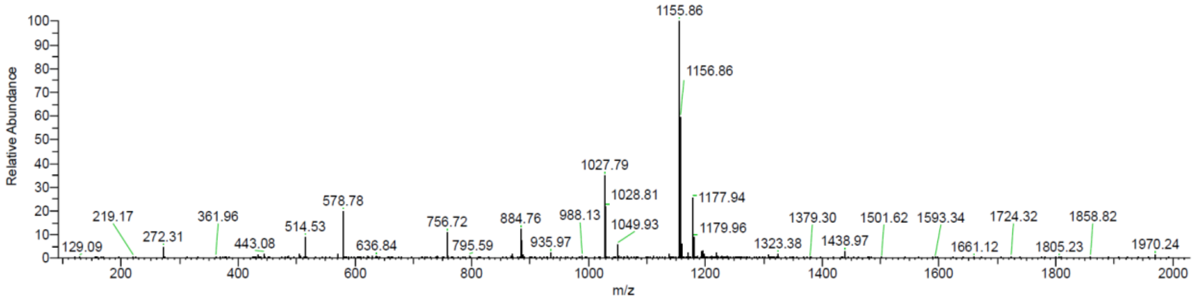

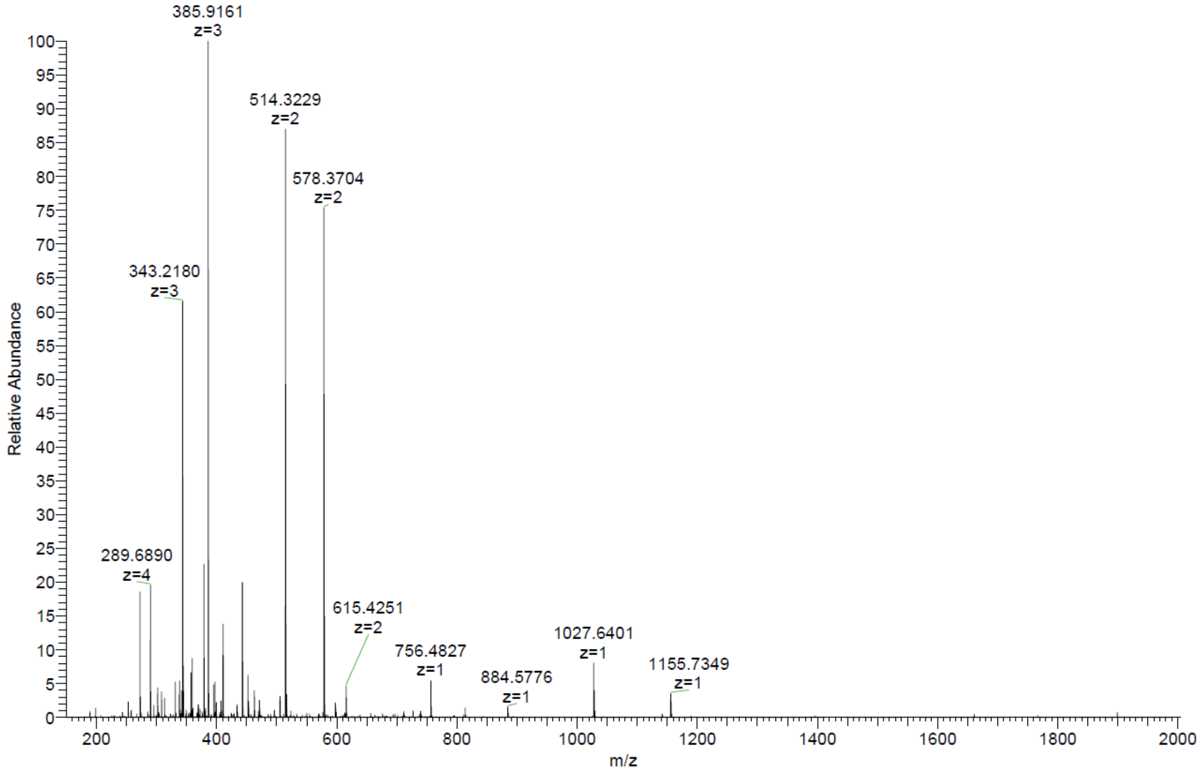
385.9161
z=3
514.3229
z=2
578.3704
z=2
343.2180
z=3
289.6890
z=4
615.4251
z=2
756.4827
z=1
884.5776
z=1
1027.6401
z=1
1155.7349
z=1
Relative Abundance
m/z

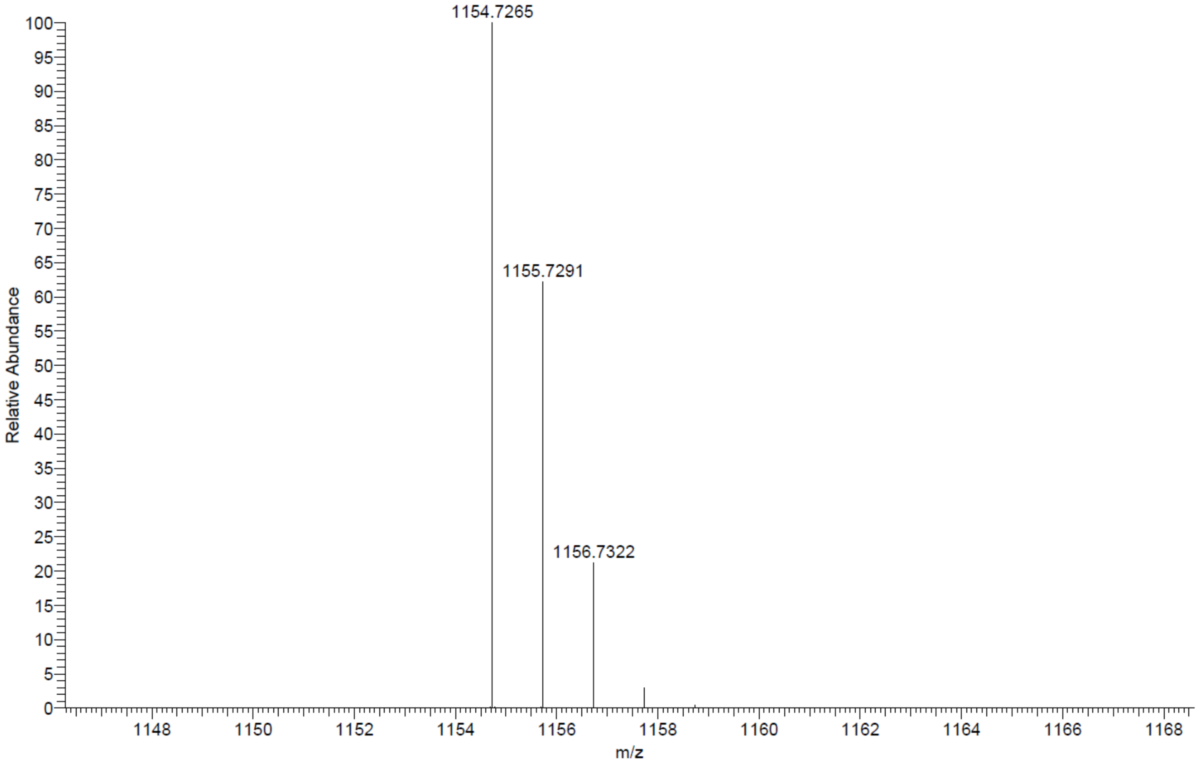
1154.7265
1155.7291
1156.7322
Relative Abundance
m/z

EB26 was obtained as a foamy white solid after preparative RP-HPLC (14 mg, 7 %). **Analytical RP-HPLC**: t_R = 3.06 min (A/D 100:0 to 0:100 in 7.0 min, λ = 214 nm). **MS** (ESI$^+$): $C_{54}H_{93}N_{17}O_{11}$ calc./obs. 1155.7240/1175.7219 Da [M].

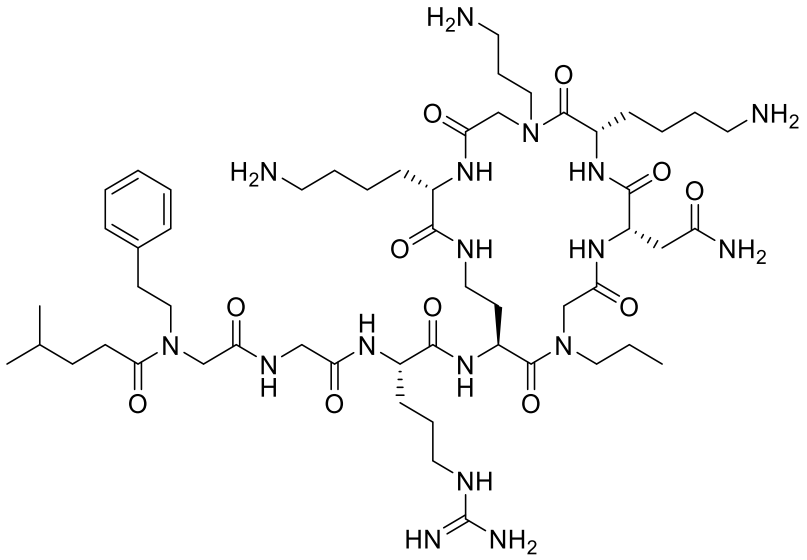

Chemical Formula: $C_{54}H_{93}N_{17}O_{11}$
Exact Mass: 1155.7240
Molecular Weight: 1156.4460

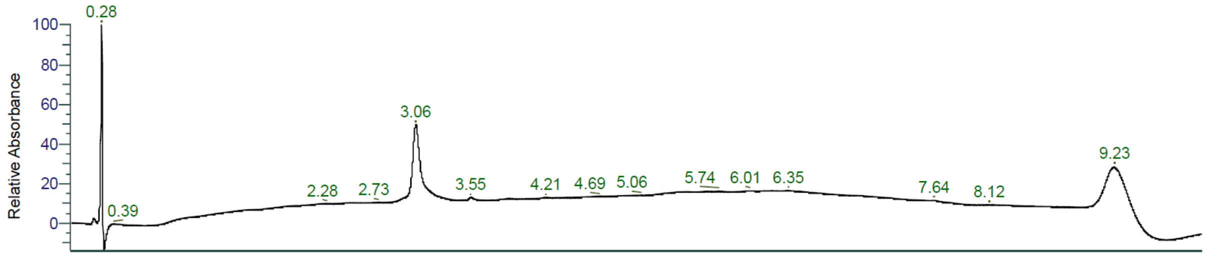

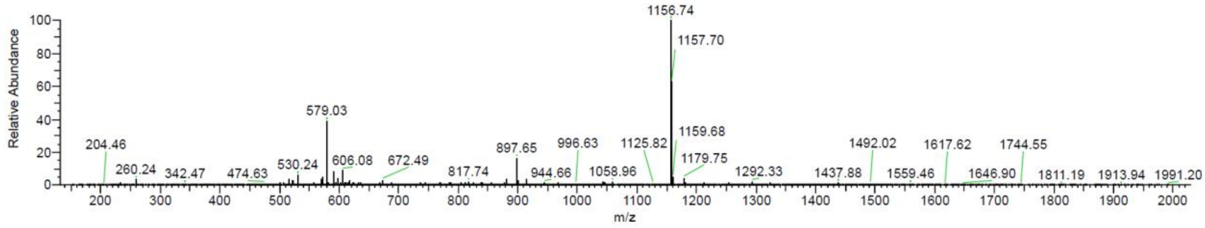

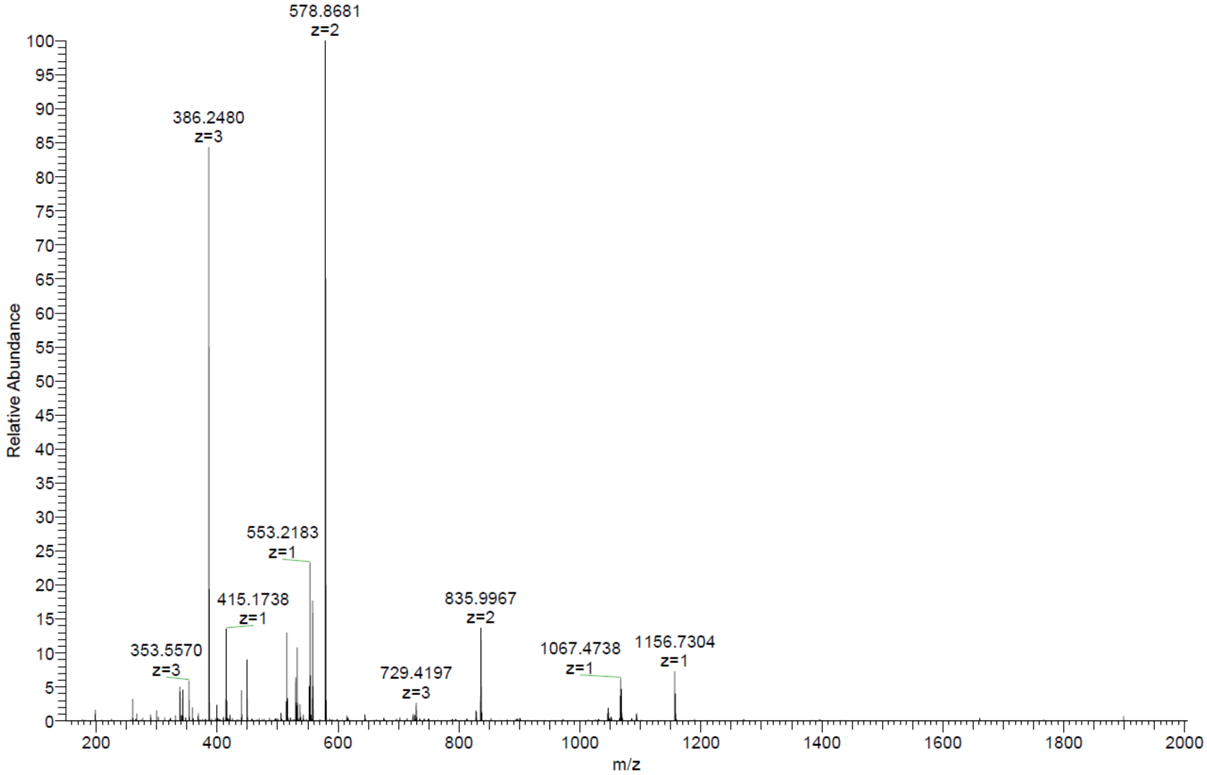
578.8681
z=2
386.2480
z=3
553.2183
z=1
415.1738
z=1
835.9967
z=2
353.5570
z=3
729.4197
z=3
1067.4738
z=1
1156.7304
z=1
Relative Abundance
m/z

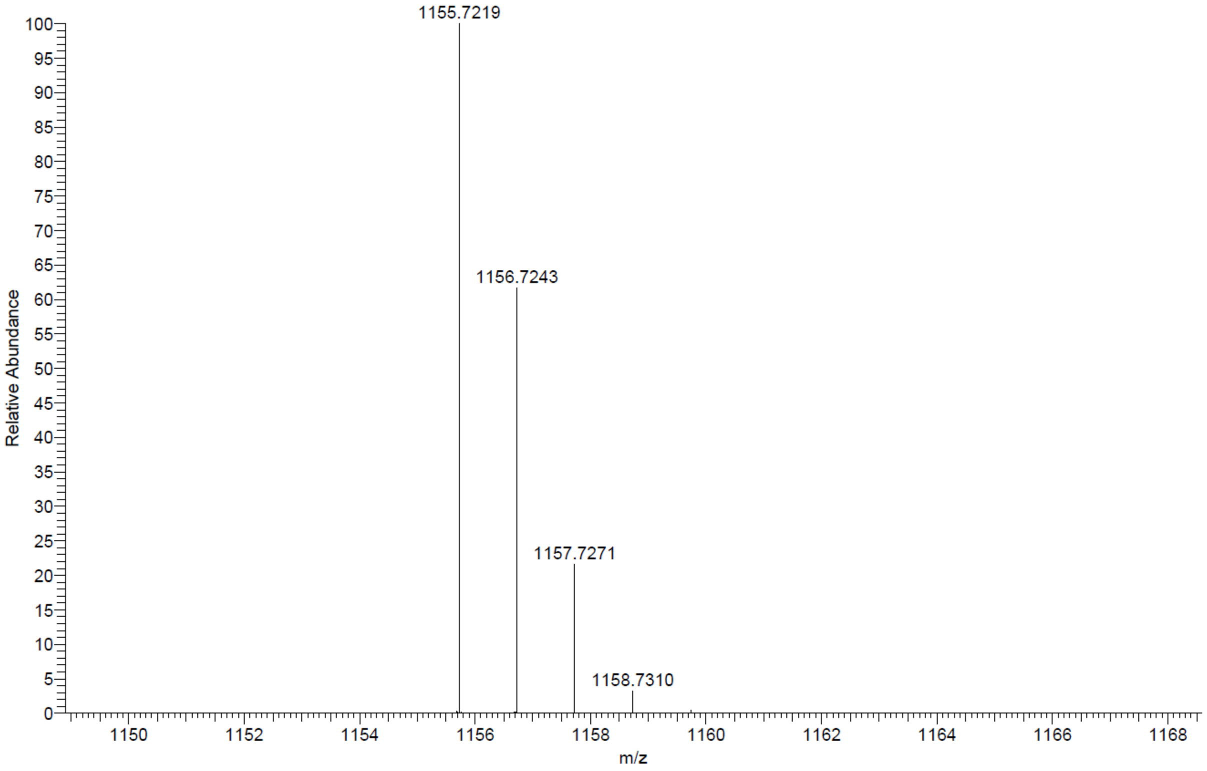
1155.7219
1156.7243
1157.7271
1158.7310
Relative Abundance
m/z

EB27 was obtained as a foamy white solid after preparative RP-HPLC (90 mg, 39 %). **Analytical RP-HPLC**: t_R = 2.50 min (A/D 100:0 to 0:100 in 7.0 min, λ = 214 nm). **MS** (ESI$^+$): $C_{53}H_{92}N_{20}O_{12}$ calc./obs. 1200.7204/1200.7193 Da [M].

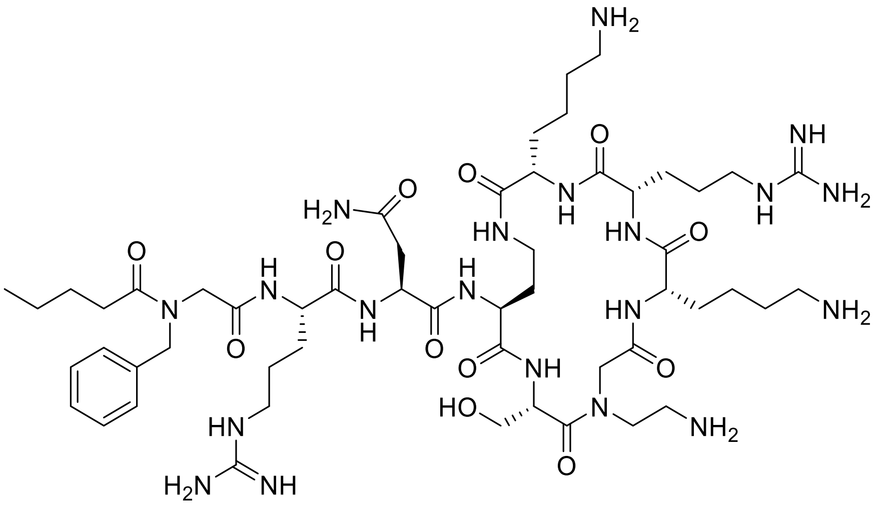

Chemical Formula: $C_{53}H_{92}N_{20}O_{12}$
Exact Mass: 1200.7204
Molecular Weight: 1201.4470

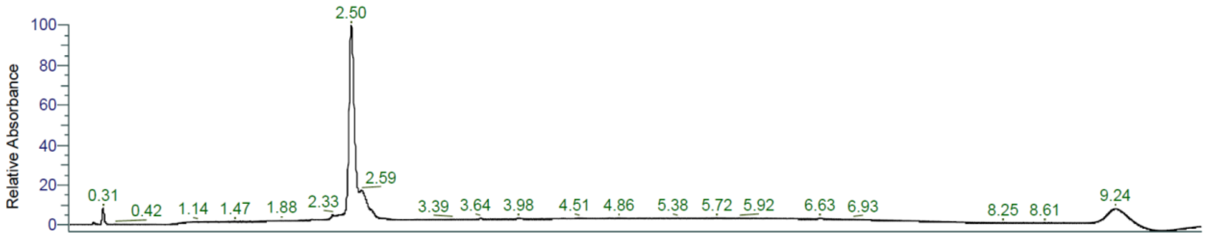

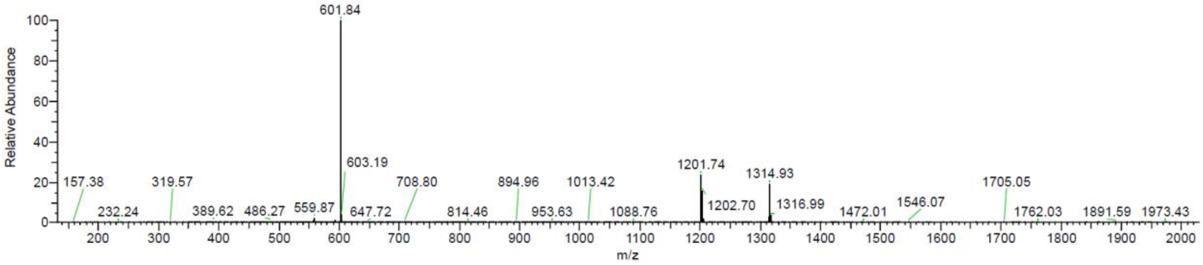

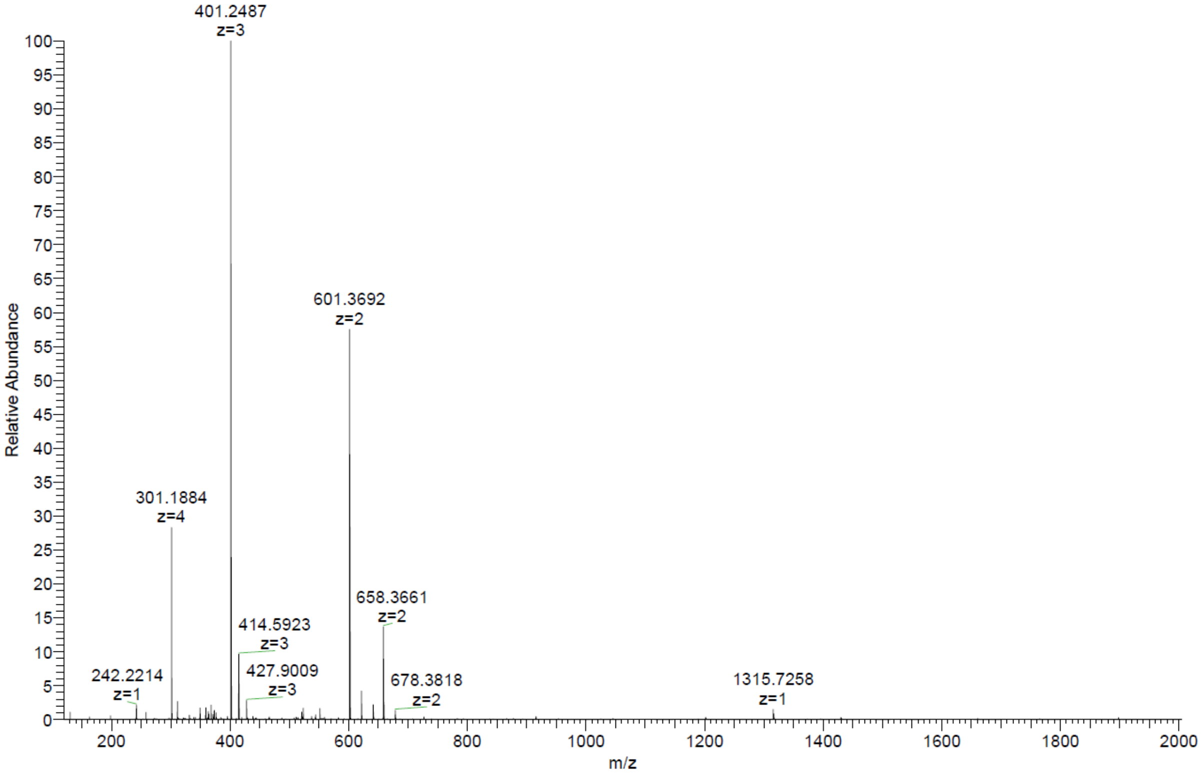
401.2487
z=3
601.3692
z=2
301.1884
z=4
658.3661
z=2
414.5923
z=3
427.9009
z=3
678.3818
z=2
242.2214
z=1
1315.7258
z=1
Relative Abundance
m/z

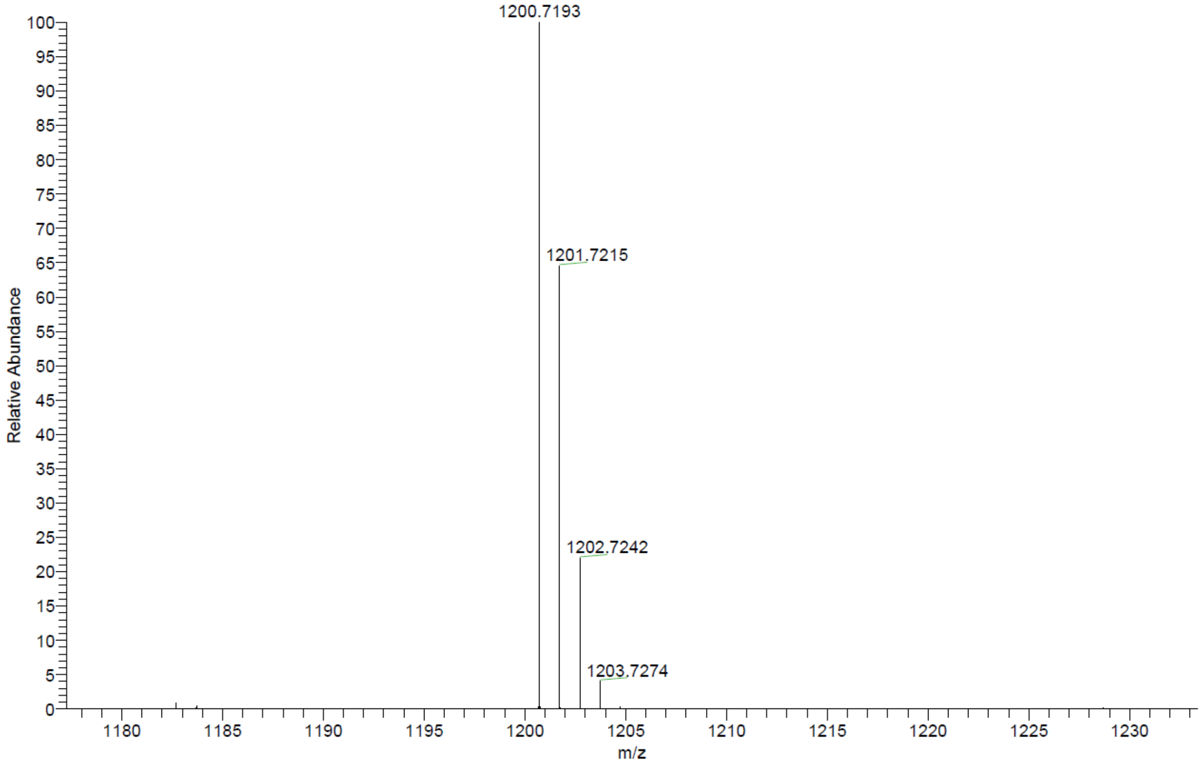
1200.7193
1201.7215
1202.7242
1203.7274
Relative Abundance
m/z

EB28 was obtained as a foamy white solid after preparative RP-HPLC (23 mg, 10 %). **Analytical RP-HPLC**: t_R = 2.23 min (A/D 100:0 to 0:100 in 7.0 min, λ = 214 nm). **MS** (ESI$^+$): $C_{57}H_{99}N_{17}O_{11}$ calc./obs. 1197.7710/1197.7696 Da [M].

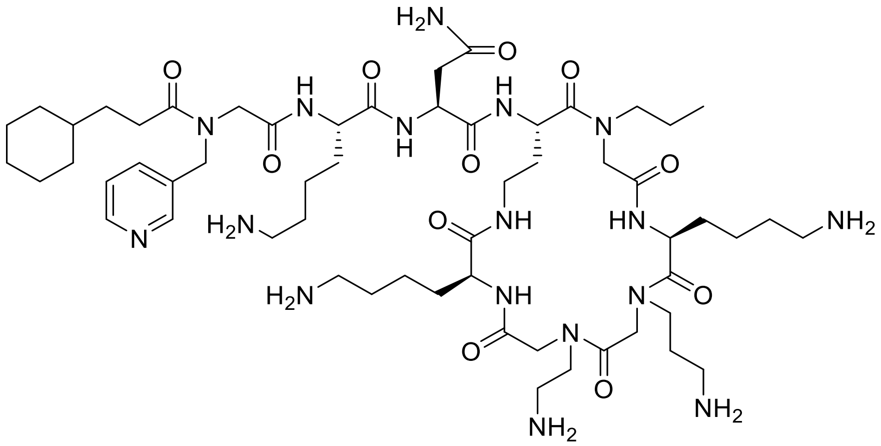

Chemical Formula: $C_{57}H_{99}N_{17}O_{11}$
Exact Mass: 1197.7710
Molecular Weight: 1198.5270

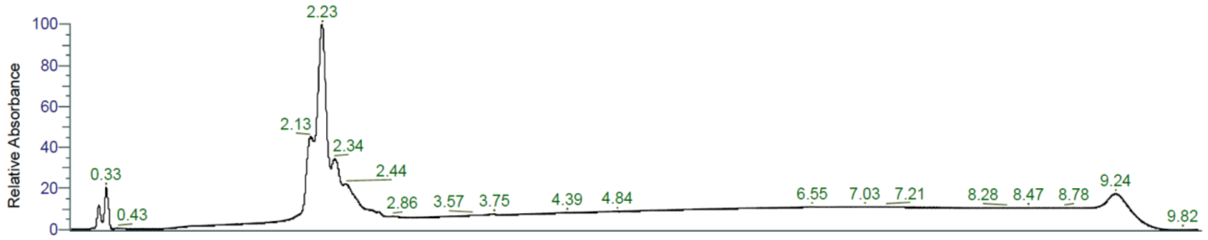

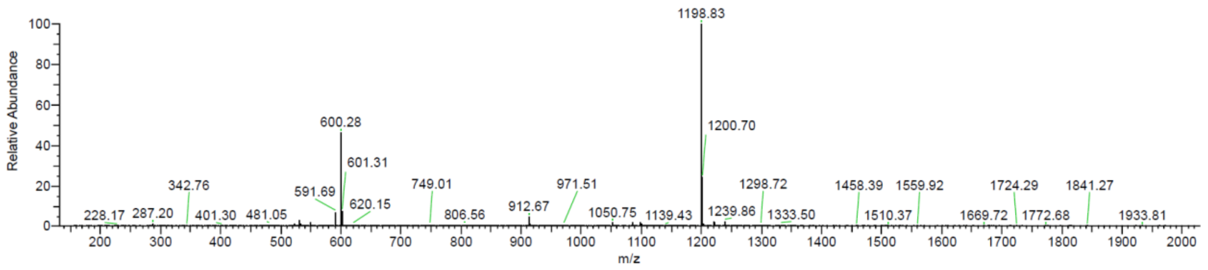

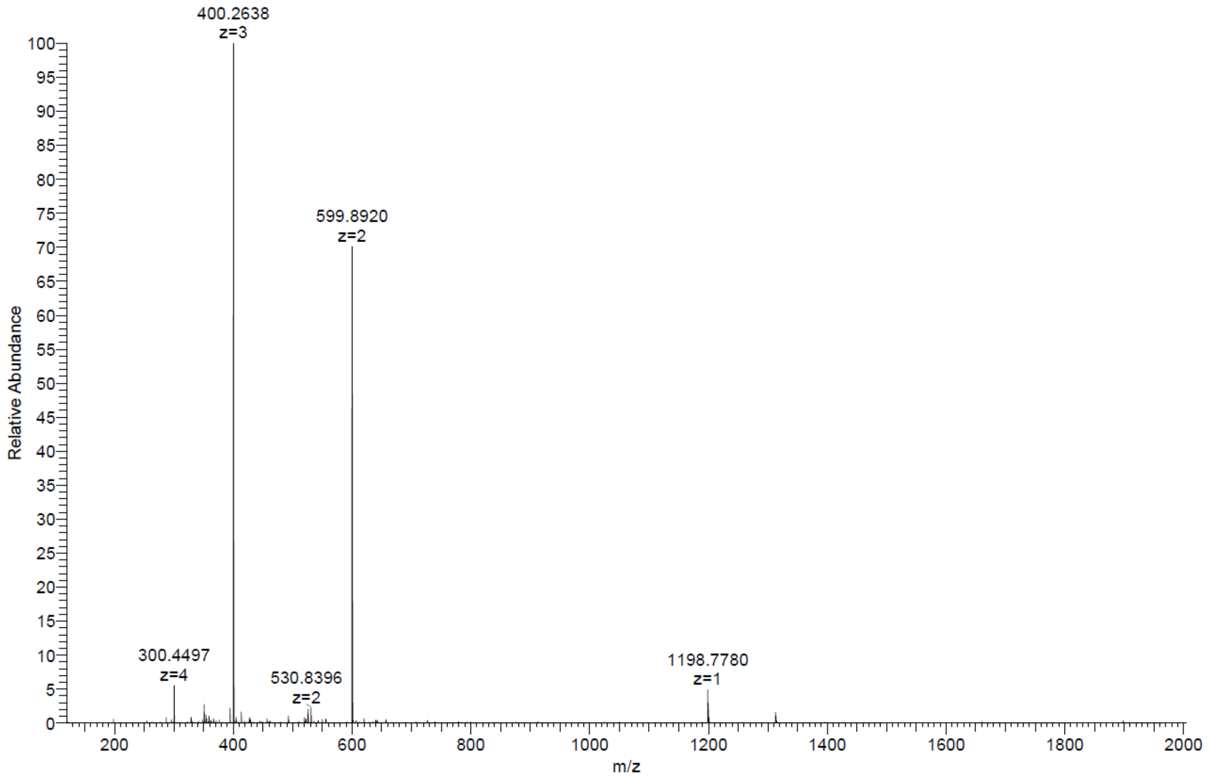

400.2638
z=3
599.8920
z=2
300.4497
z=4
530.8396
z=2
1198.7780
z=1
Relative Abundance
m/z

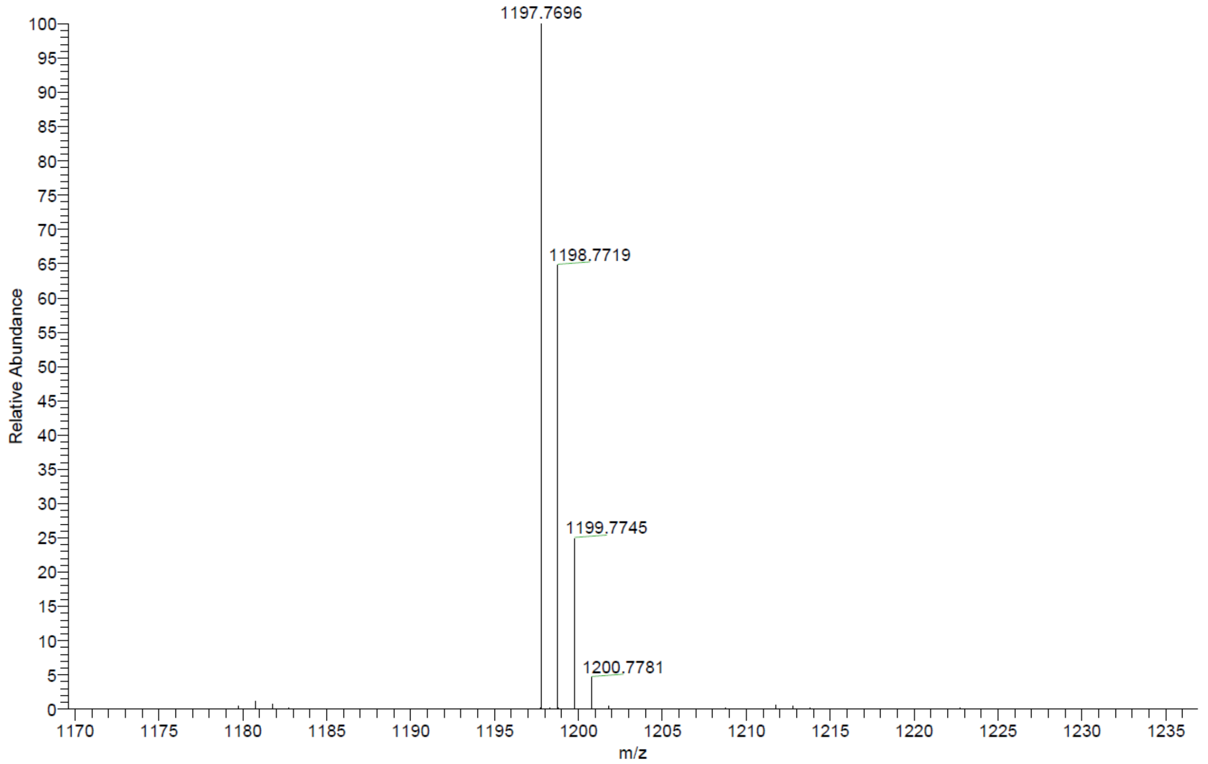

1197.7696
1198.7719
1199.7745
1200.7781
Relative Abundance
m/z

EB29 was obtained as a foamy white solid after preparative RP-HPLC (14 mg, 4 %). **Analytical RP-HPLC**: t_R = 2.65 min (A/D 100:0 to 0:100 in 7.0 min, λ = 214 nm). **MS** (ESI$^+$): $C_{59}H_{102}N_{16}O_{12}$ calc./obs. 1226.7863/1226.7845 Da [M].

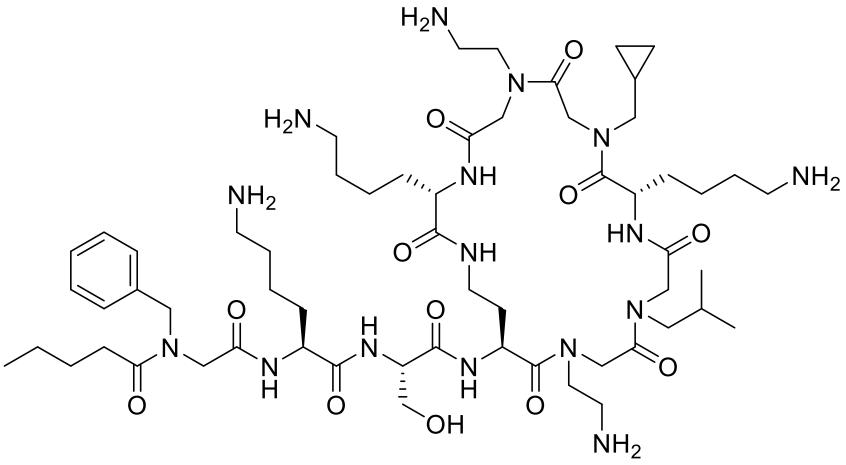

Chemical Formula: $C_{59}H_{102}N_{16}O_{12}$
Exact Mass: 1226.7863
Molecular Weight: 1227.5650

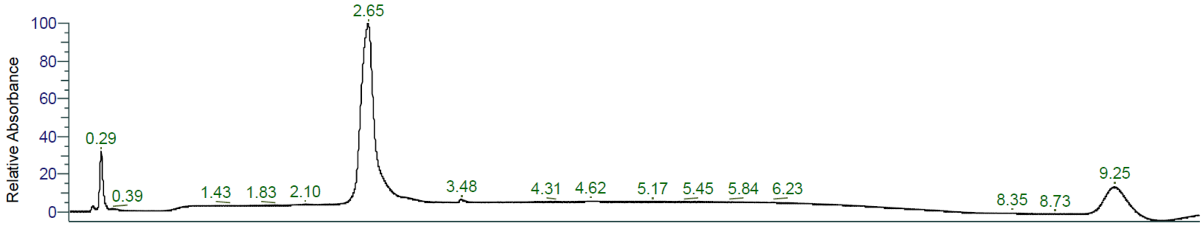

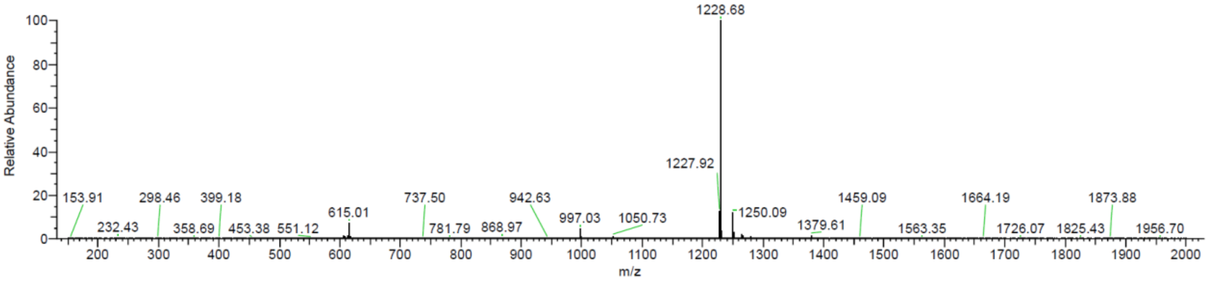

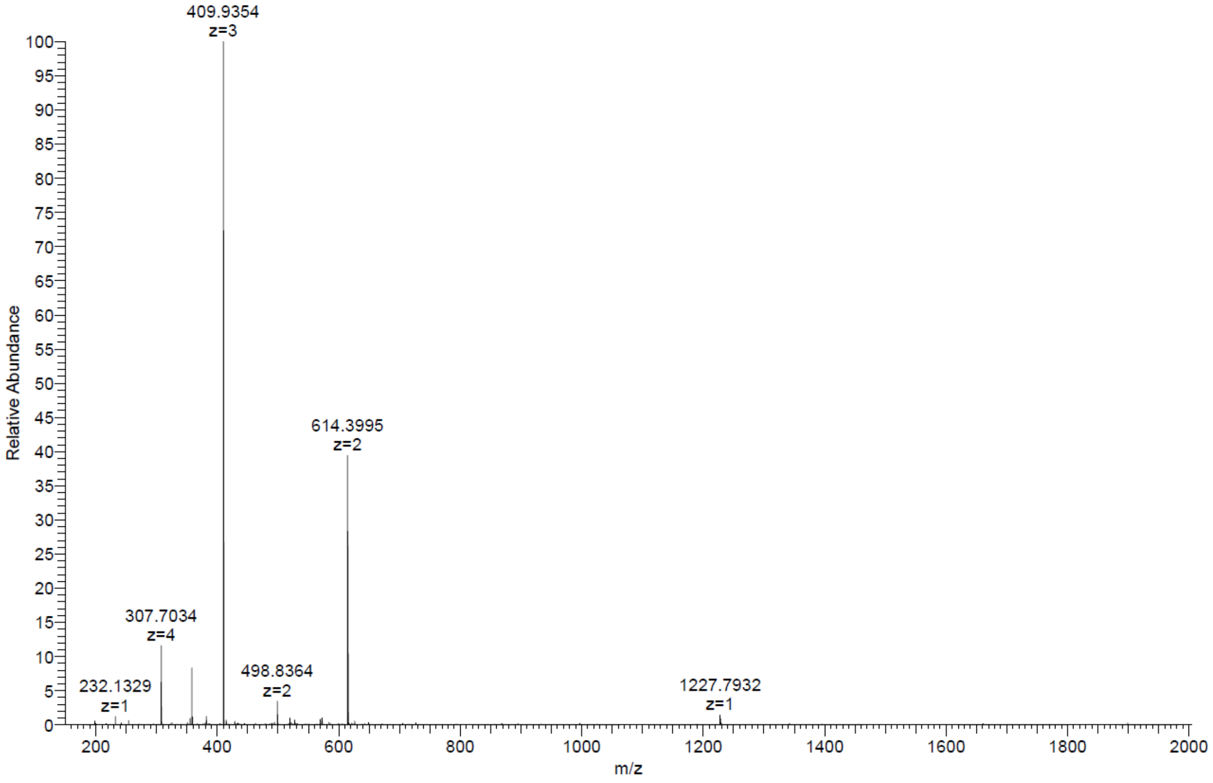
409.9354
z=3
614.3995
z=2
307.7034
z=4
232.1329
z=1
498.8364
z=2
1227.7932
z=1
Relative Abundance
m/z

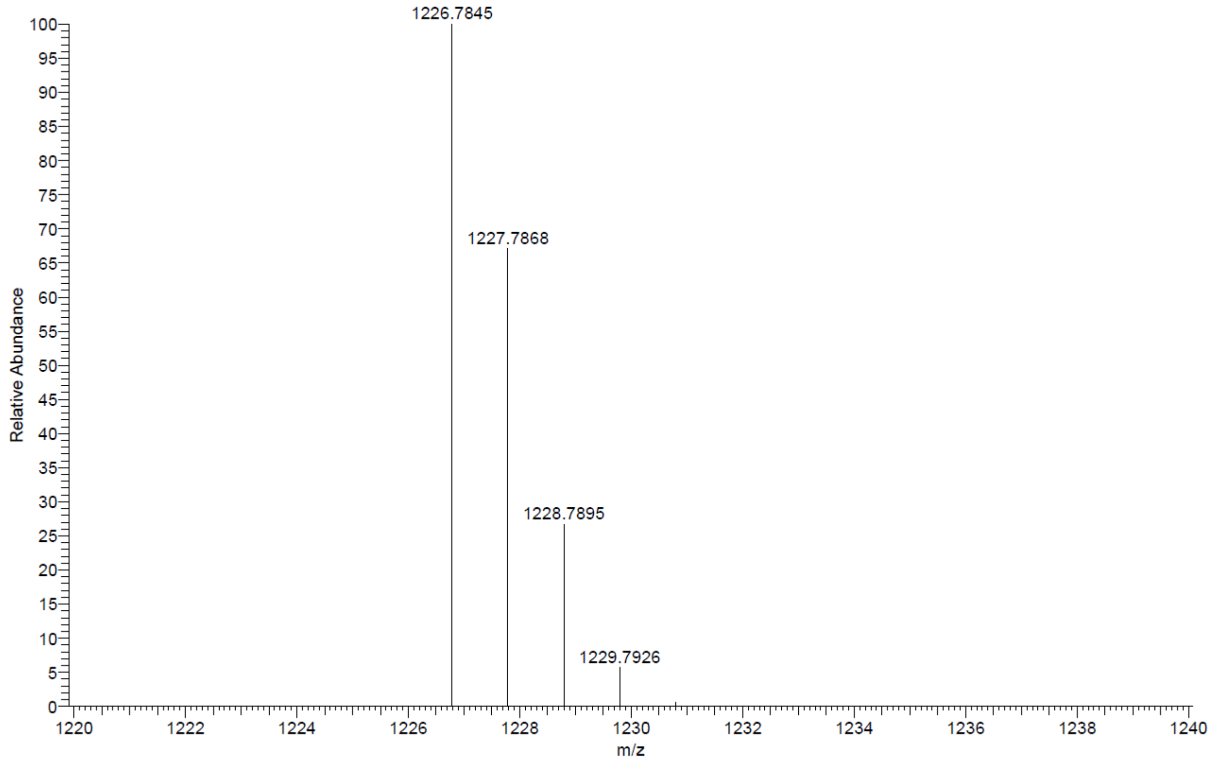
1226.7845
1227.7868
1228.7895
1229.7926
Relative Abundance
m/z

EB30 was obtained as a foamy white solid after preparative RP-HPLC (50 mg, 22 %). **Analytical RP-HPLC**: t_R = 2.38 min (A/D 100:0 to 0:100 in 7.0 min, λ = 214 nm). **MS** (ESI$^+$): $C_{55}H_{95}N_{17}O_{12}$ calc./obs. 1185.7346/1185.7326 Da [M].

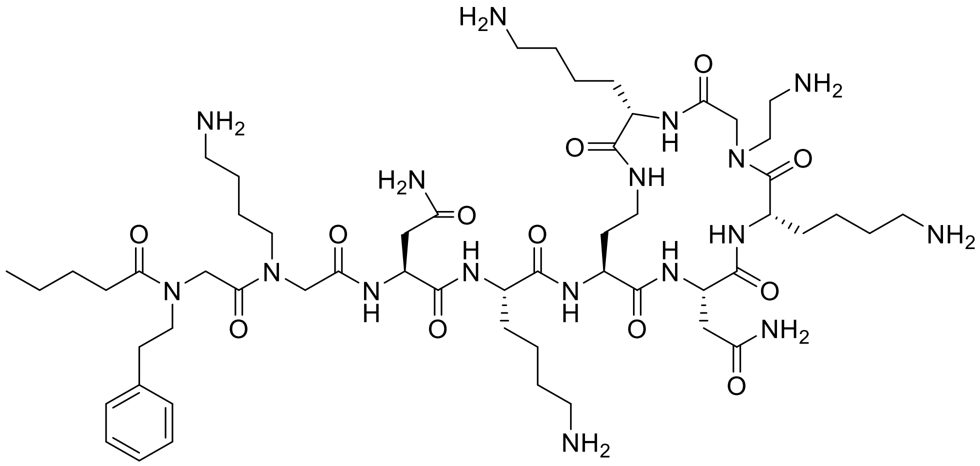

Chemical Formula: $C_{55}H_{95}N_{17}O_{12}$
Exact Mass: 1185.7346
Molecular Weight: 1186.4720

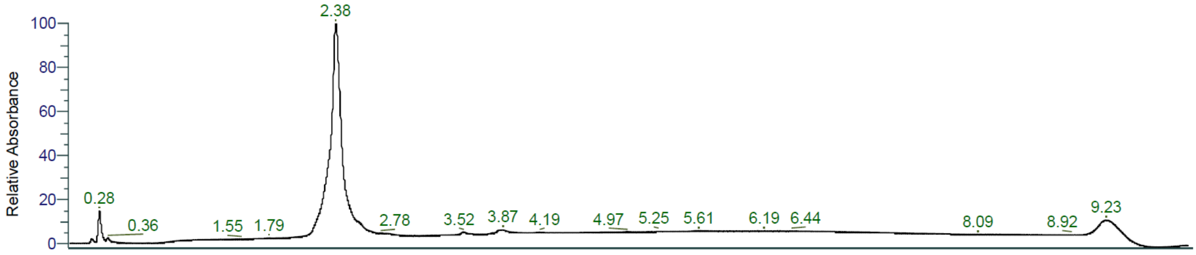

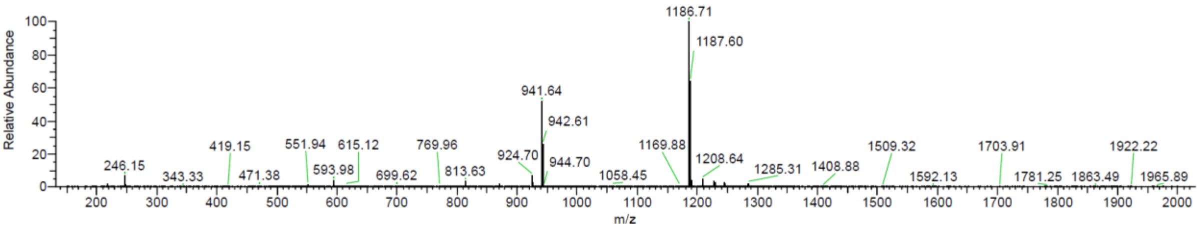

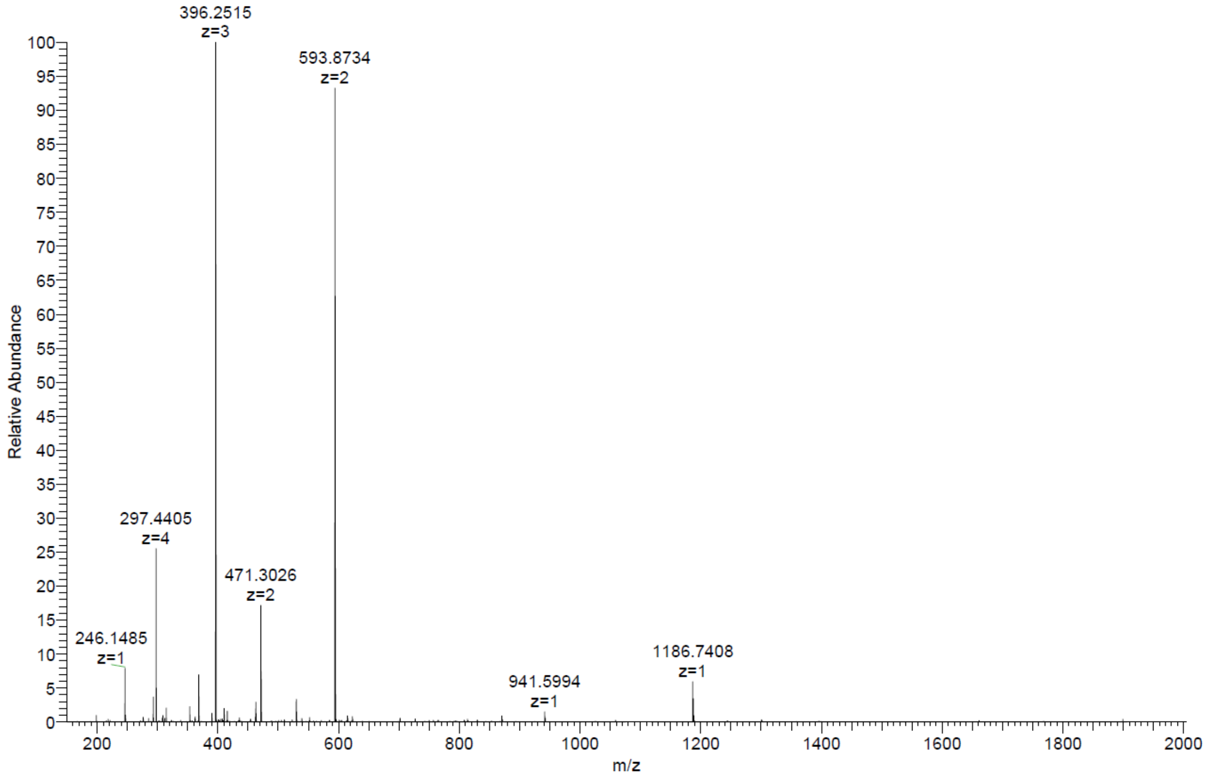
396.2515
z=3
593.8734
z=2
297.4405
z=4
471.3026
z=2
246.1485
z=1
941.5994
z=1
1186.7408
z=1
Relative Abundance
m/z

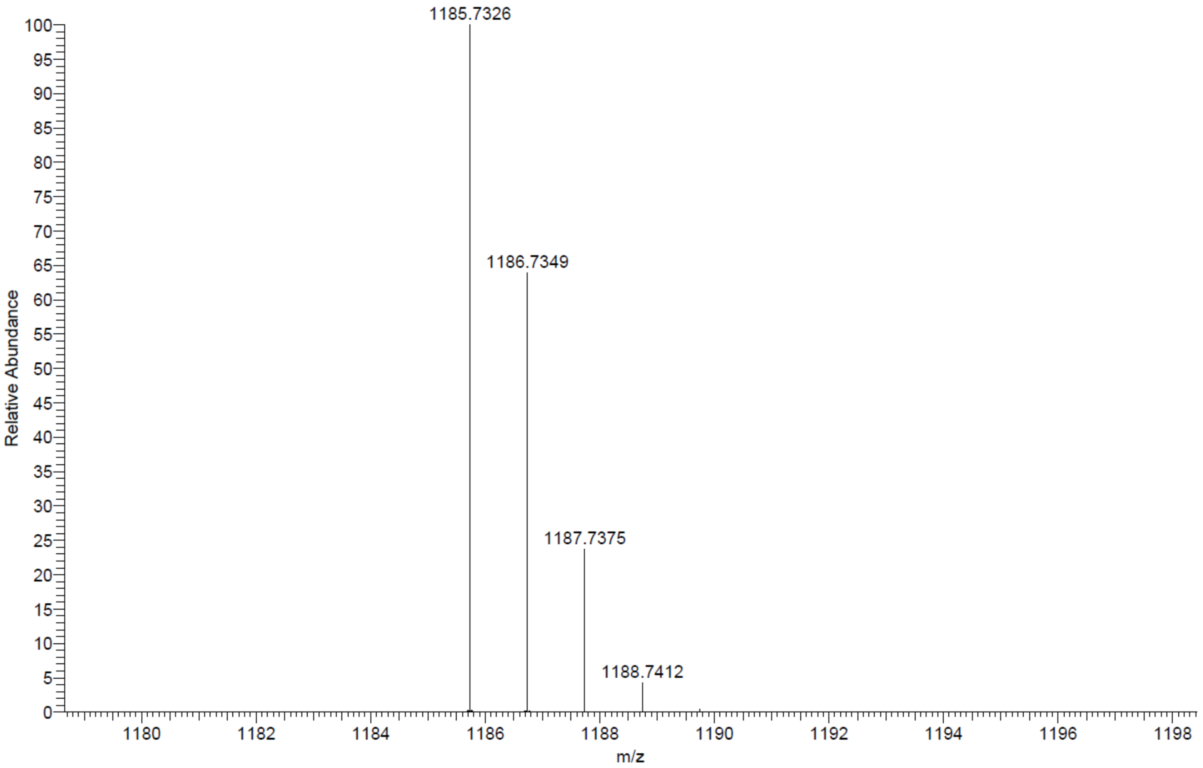
1185.7326
1186.7349
1187.7375
1188.7412
Relative Abundance
m/z

EB31 was obtained as a foamy white solid after preparative RP-HPLC (14 mg, 6 %).

Analytical RP-HPLC: t_R = 2.35 min (A/D 100:0 to 0:100 in 7.0 min, λ = 214 nm). **MS** (ESI$^+$): $C_{58}H_{100}N_{16}O_{13}$ calc./obs. 1228.7656/1228.7634 Da [M].

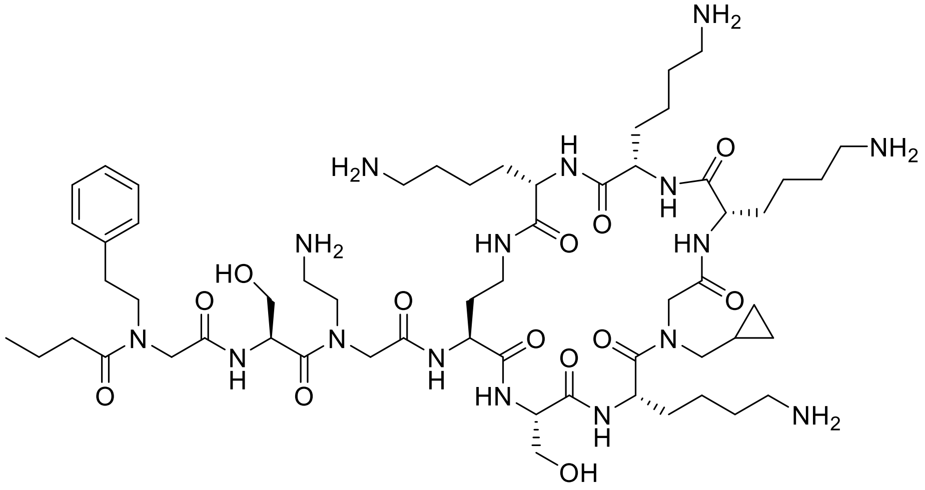

Chemical Formula: $C_{58}H_{100}N_{16}O_{13}$
Exact Mass: 1228.7656
Molecular Weight: 1229.5370

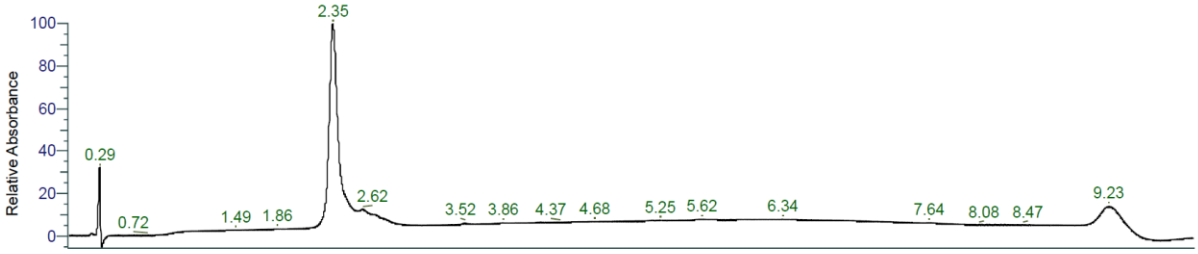

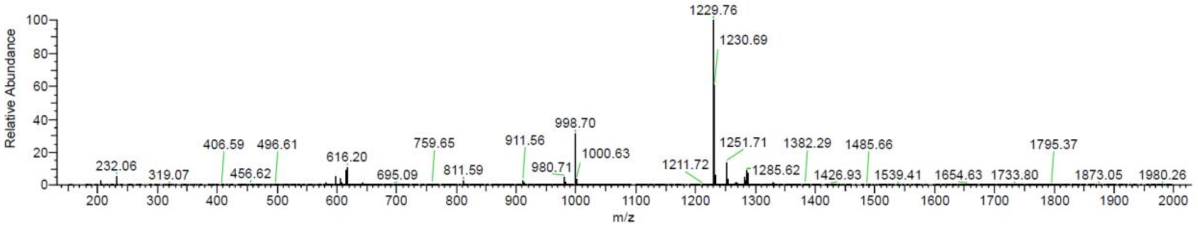

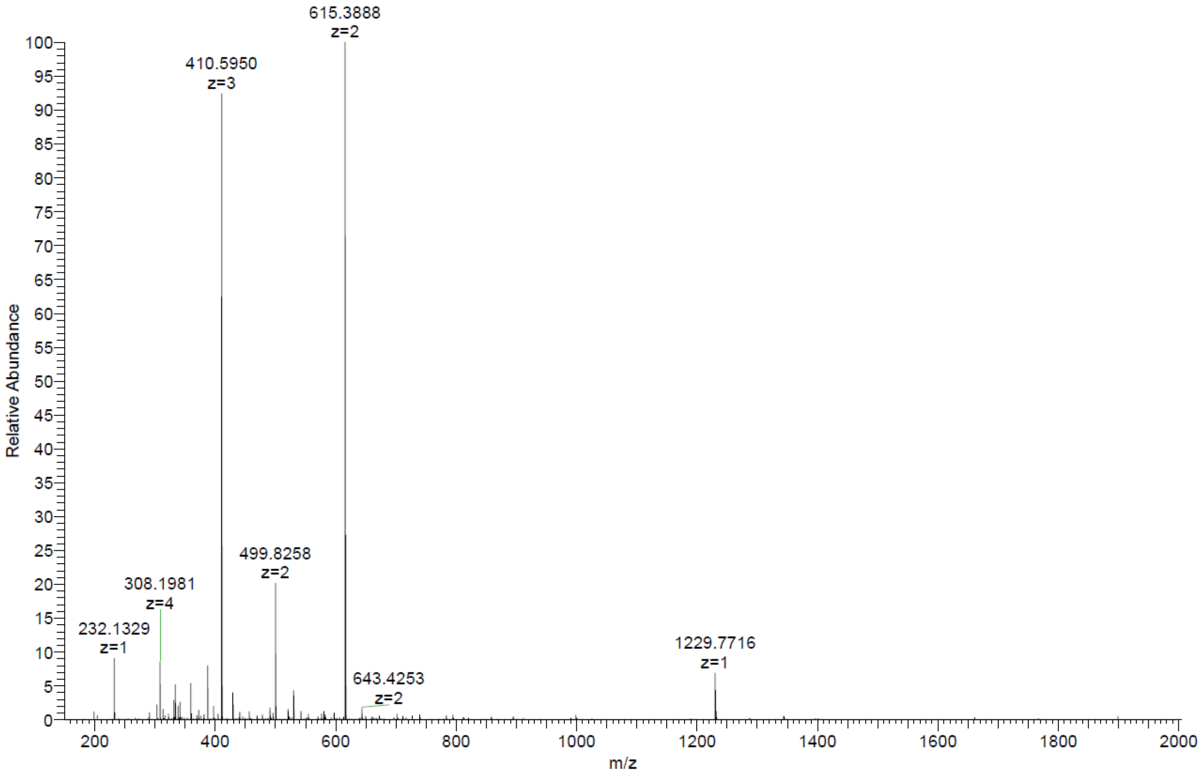
615.3888
z=2
410.5950
z=3
499.8258
z=2
308.1981
z=4
232.1329
z=1
643.4253
z=2
1229.7716
z=1
Relative Abundance
m/z

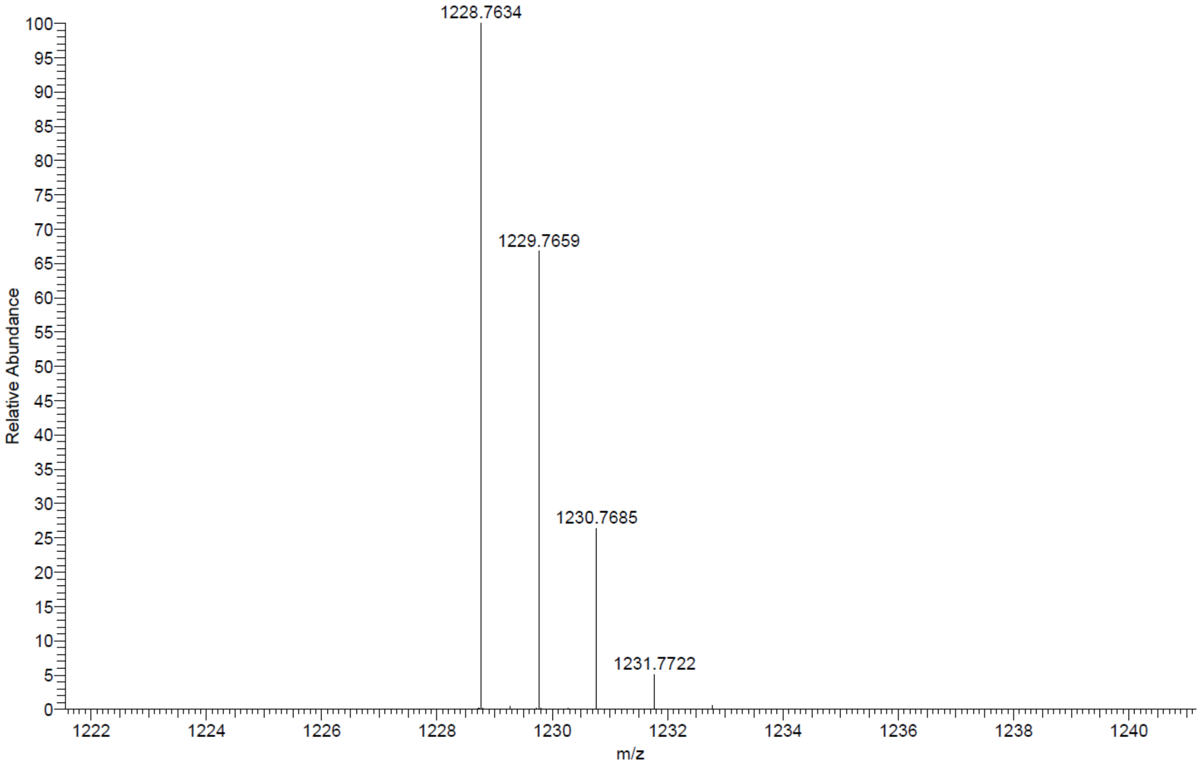
1228.7634
1229.7659
1230.7685
1231.7722
Relative Abundance
m/z

EB32 was obtained as a foamy white solid after preparative RP-HPLC (61 mg, 23 %). **Analytical RP-HPLC**: t_R = 2.93 min (A/D 100:0 to 0:100 in 7.0 min, λ = 214 nm). **MS** (ESI[+]): $C_{57}H_{95}N_{15}O_{12}$ calc./obs. 1181.7285/1181.7324 Da [M].

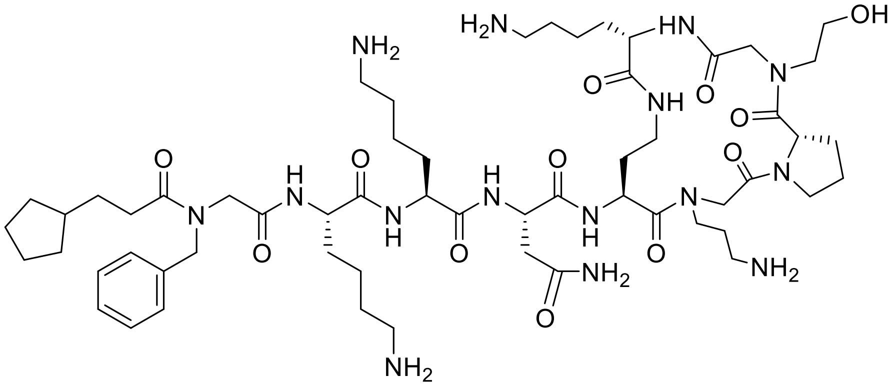

Chemical Formula: $C_{57}H_{95}N_{15}O_{12}$
Exact Mass: 1181.7285
Molecular Weight: 1182.4800

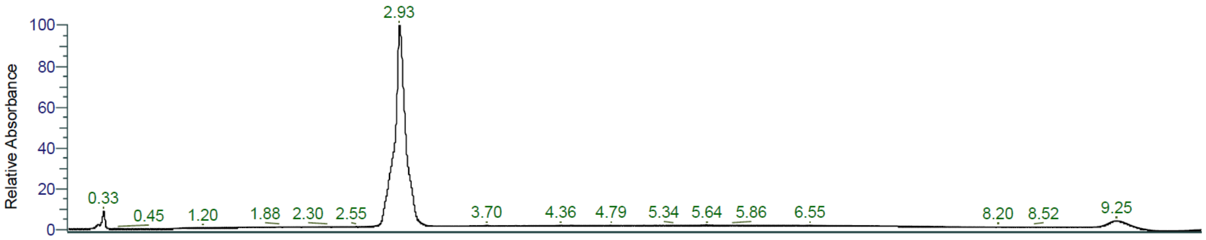

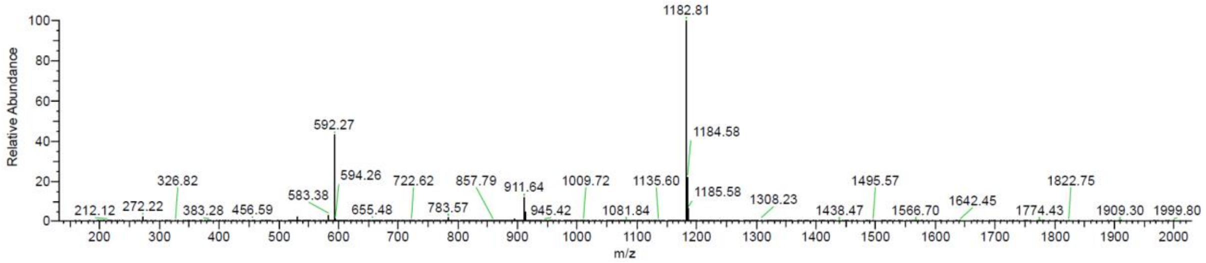

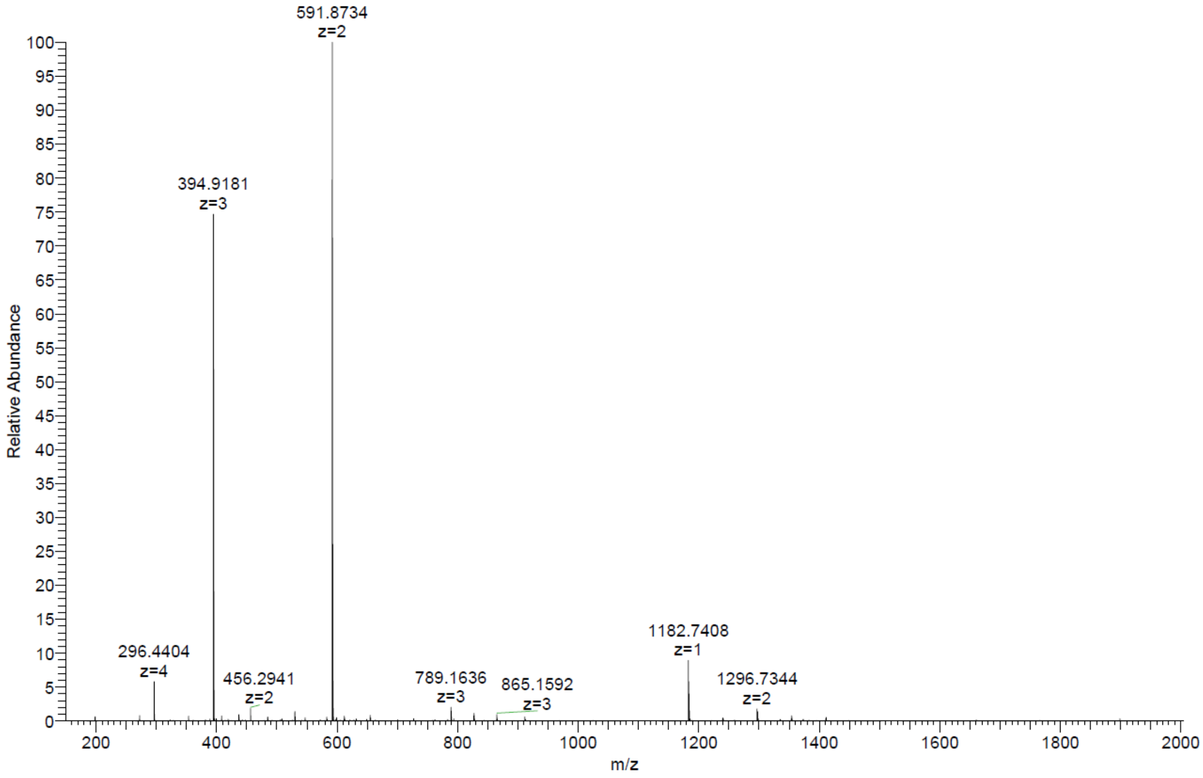
591.8734
z=2
394.9181
z=3
296.4404
z=4
456.2941
z=2
789.1636
z=3
865.1592
z=3
1182.7408
z=1
1296.7344
z=2
Relative Abundance
m/z

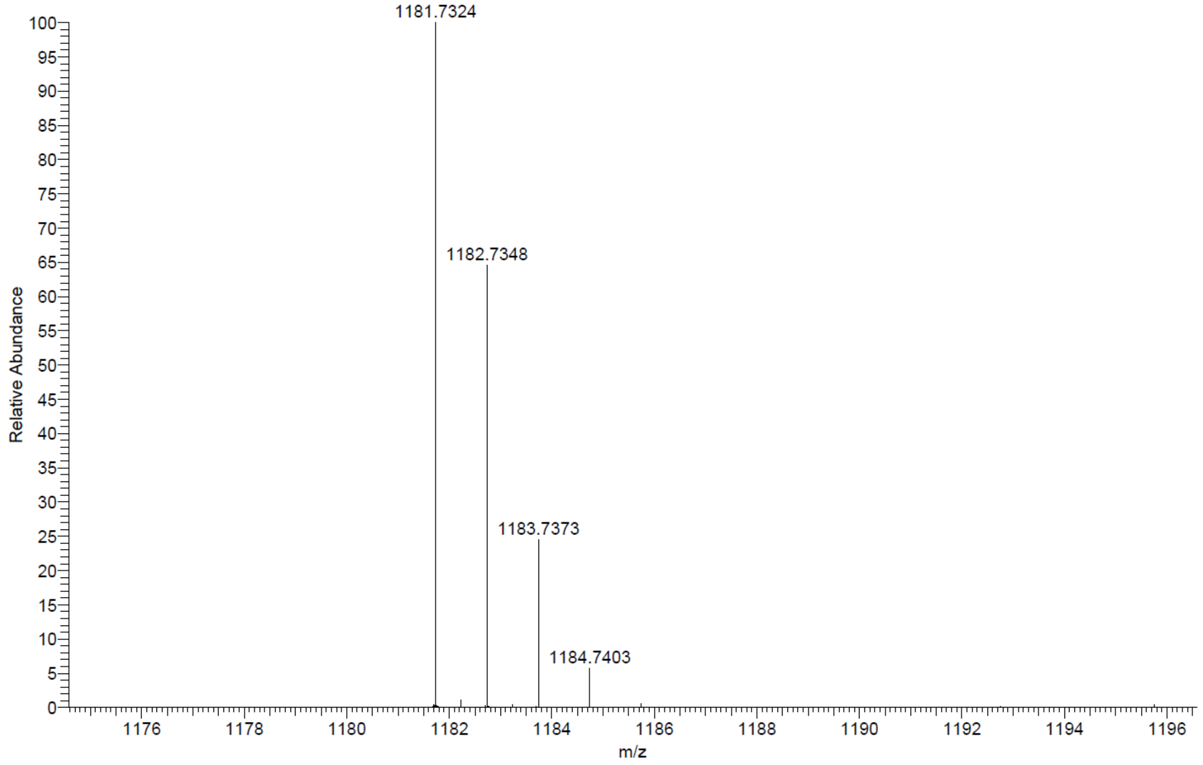
1181.7324
1182.7348
1183.7373
1184.7403
Relative Abundance
m/z

EB33 was obtained as a foamy white solid after preparative RP-HPLC (19 mg, 6 %). **Analytical RP-HPLC**: t_R = 2.49 min (A/D 100:0 to 0:100 in 7.0 min, λ = 214 nm). **MS** (ESI$^+$): $C_{61}H_{110}N_{18}O_{12}$ calc./obs. 1286.8551/1286.8593 Da [M].

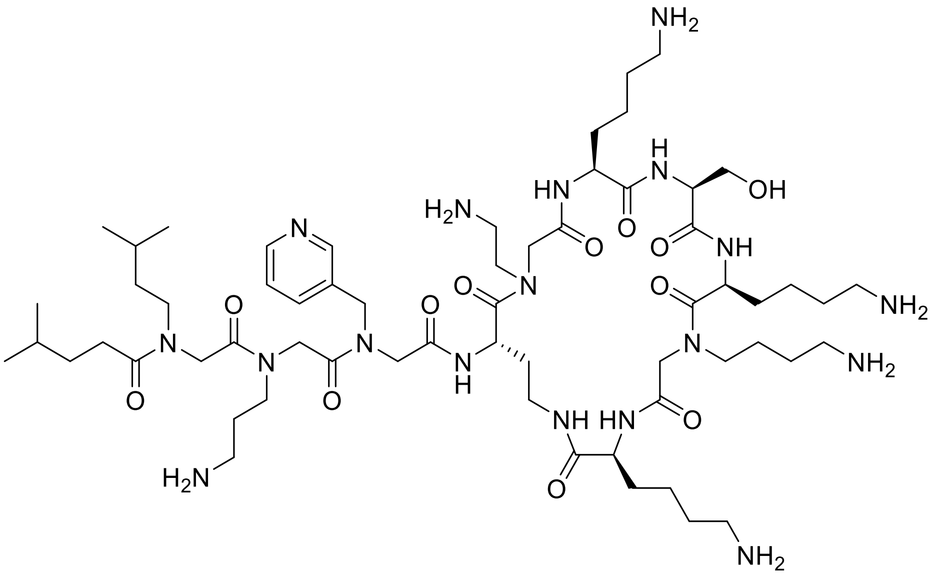

Chemical Formula: $C_{61}H_{110}N_{18}O_{12}$
Exact Mass: 1286.8551
Molecular Weight: 1287.6650

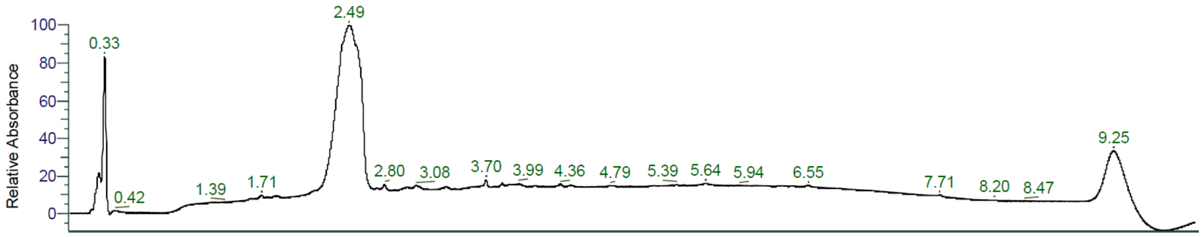

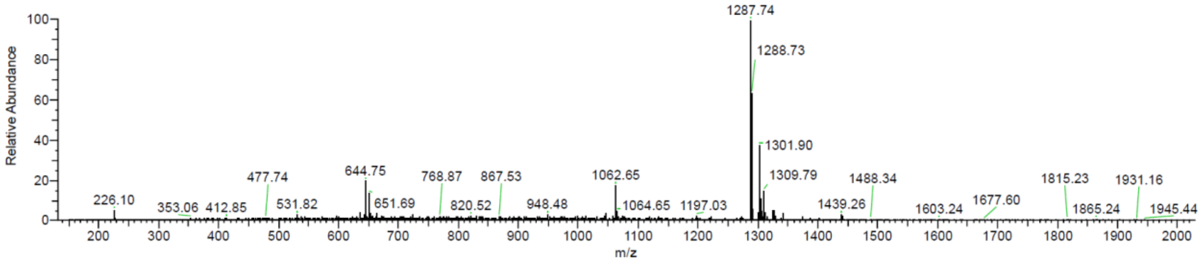

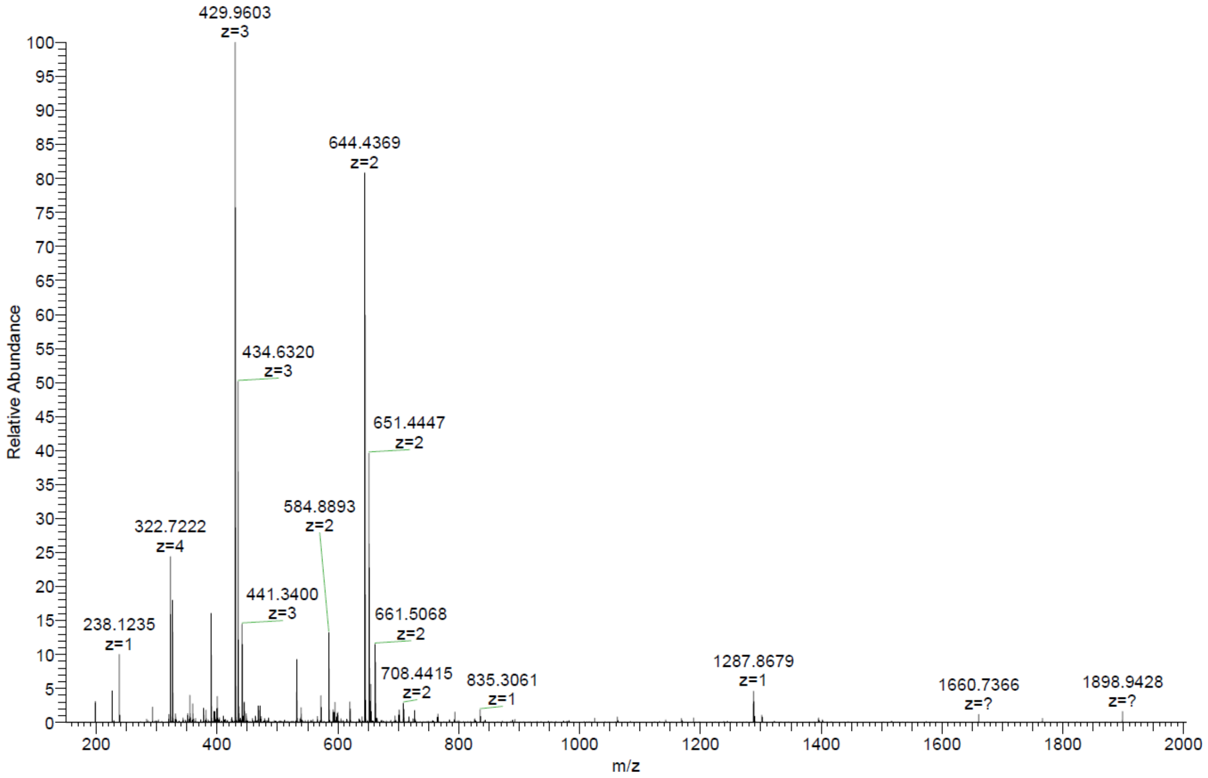
429.9603
z=3
644.4369
z=2
434.6320
z=3
651.4447
z=2
584.8893
z=2
322.7222
z=4
441.3400
z=3
661.5068
z=2
238.1235
z=1
708.4415
z=2
835.3061
z=1
1287.8679
z=1
1660.7366
z=?
1898.9428
z=?
Relative Abundance
m/z

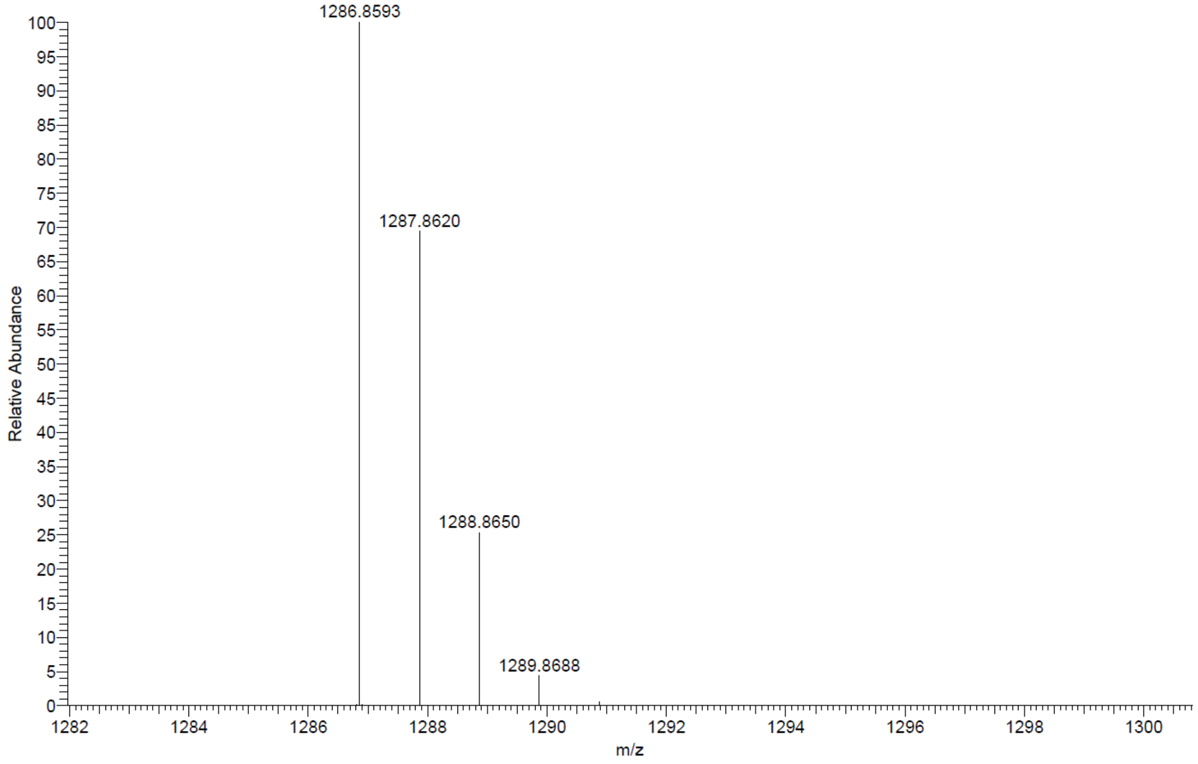
1286.8593
1287.8620
1288.8650
1289.8688
Relative Abundance
m/z

EB34 was obtained as a foamy white solid after preparative RP-HPLC (46 mg, 15 %). **Analytical RP-HPLC**: t_R = 2.15 min (A/D 100:0 to 0:100 in 7.0 min, λ = 214 nm). **MS** (ESI$^+$): $C_{53}H_{92}N_{16}O_{11}$ calc./obs. 1128.7131/1128.7133 Da [M].

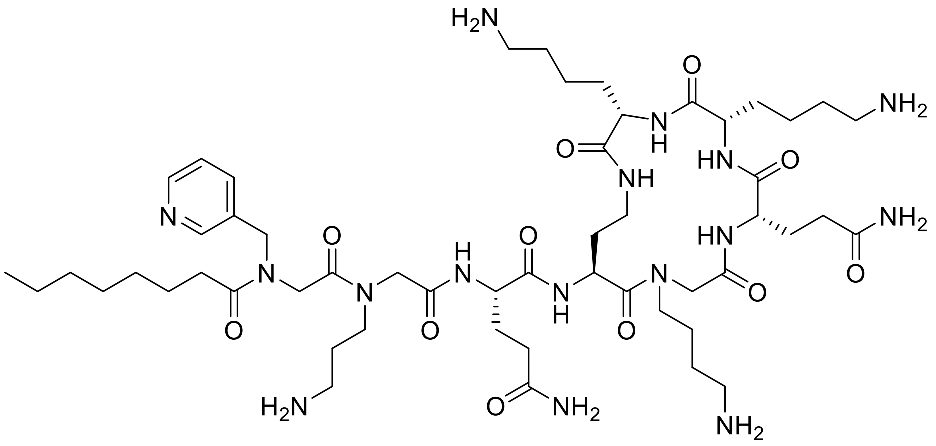

Chemical Formula: $C_{53}H_{92}N_{16}O_{11}$
Exact Mass: 1128.7131
Molecular Weight: 1129.4200

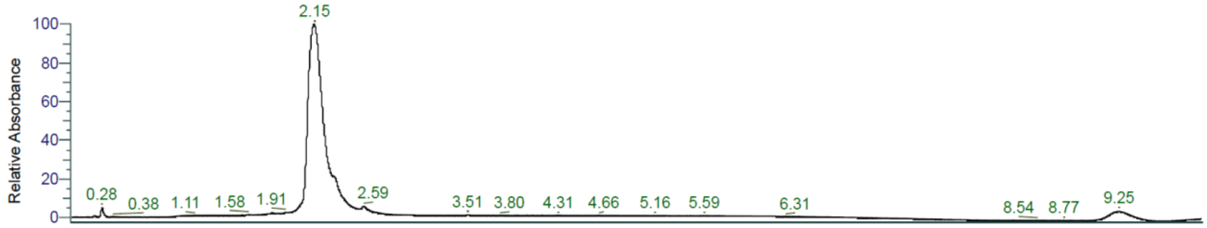

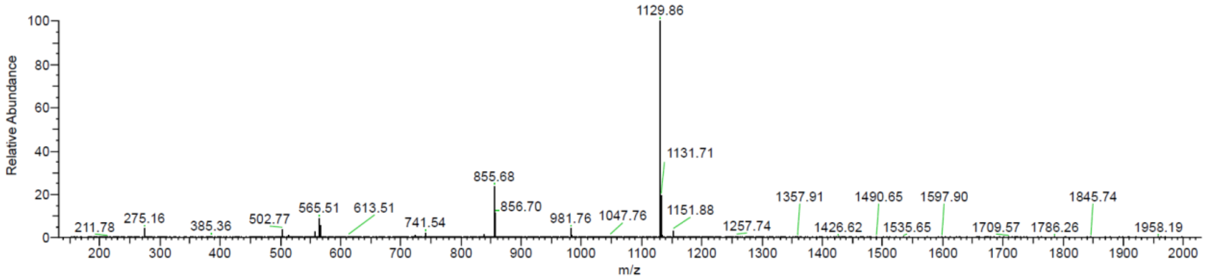

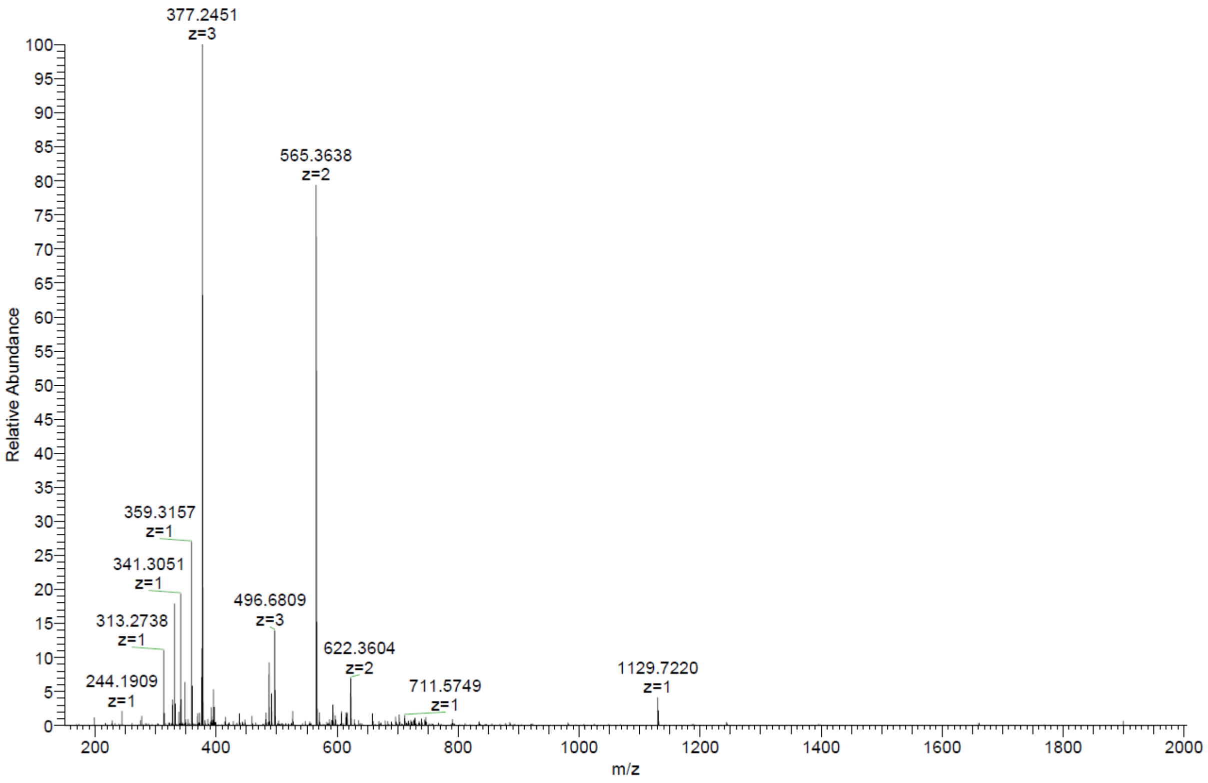
377.2451
z=3
565.3638
z=2
359.3157
z=1
341.3051
z=1
496.6809
z=3
313.2738
z=1
622.3604
z=2
711.5749
z=1
1129.7220
z=1
244.1909
z=1
Relative Abundance
m/z

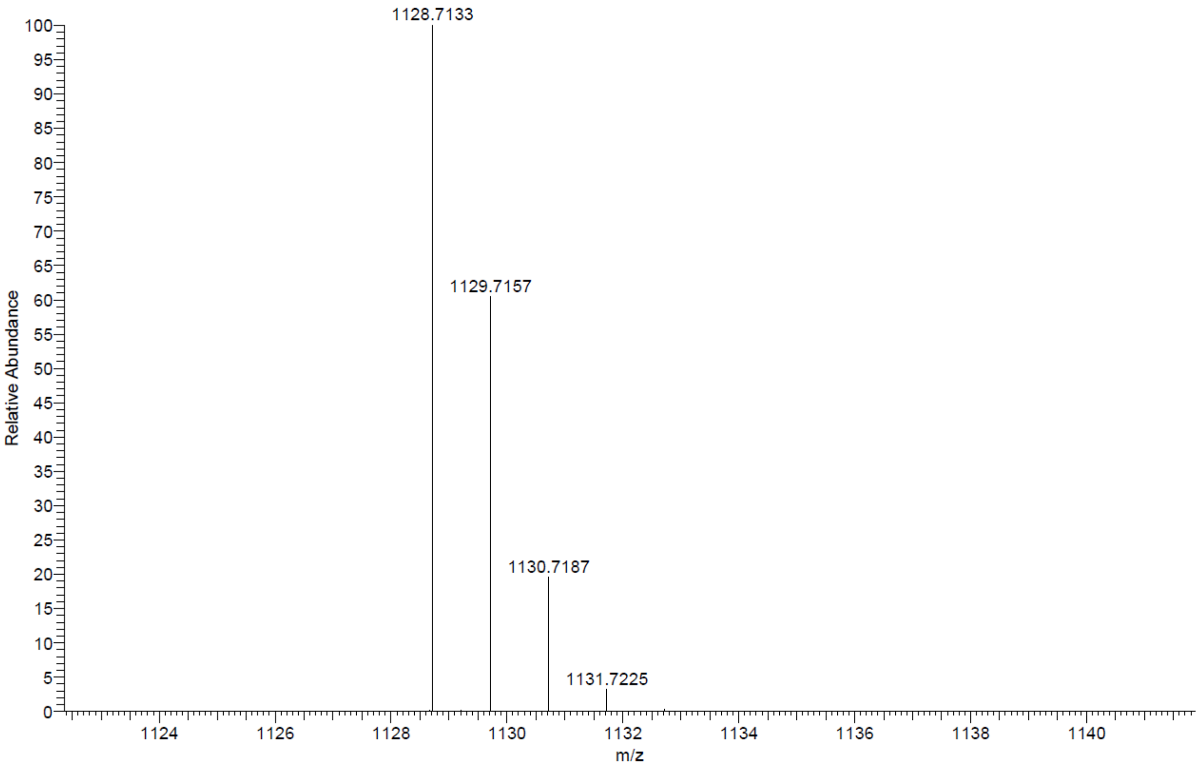
1128.7133
1129.7157
1130.7187
1131.7225
Relative Abundance
m/z

EB35 was obtained as a foamy white solid after preparative RP-HPLC (100 mg, 31 %). **Analytical RP-HPLC**: t_R = 2.25 min (A/D 100:0 to 0:100 in 7.0 min, λ = 214 nm). **MS** (ESI$^+$): $C_{55}H_{96}N_{16}O_{12}$ calc./obs. 1172.7394/1172.7389 Da [M].

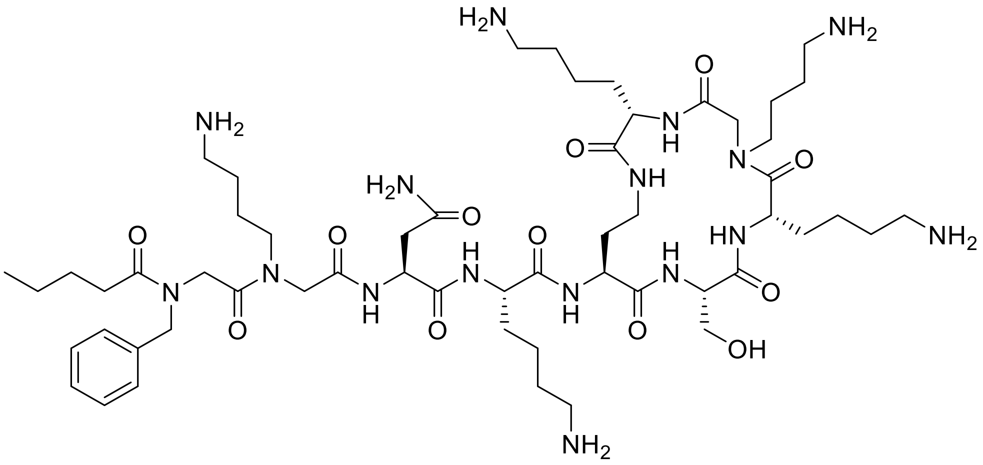

Chemical Formula: $C_{55}H_{96}N_{16}O_{12}$
Exact Mass: 1172.7394
Molecular Weight: 1173.4730

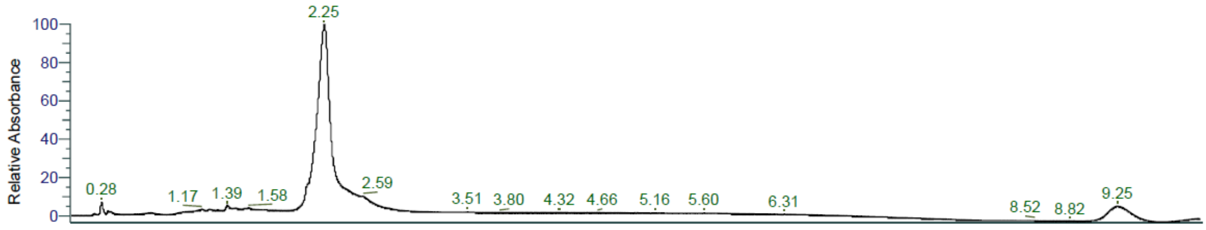

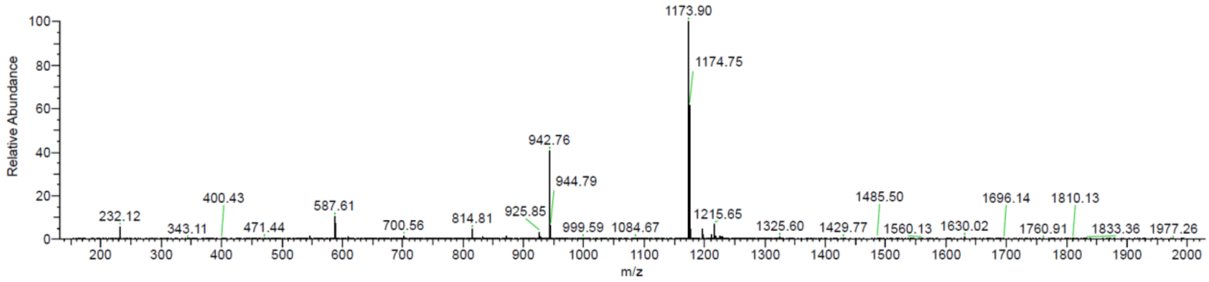

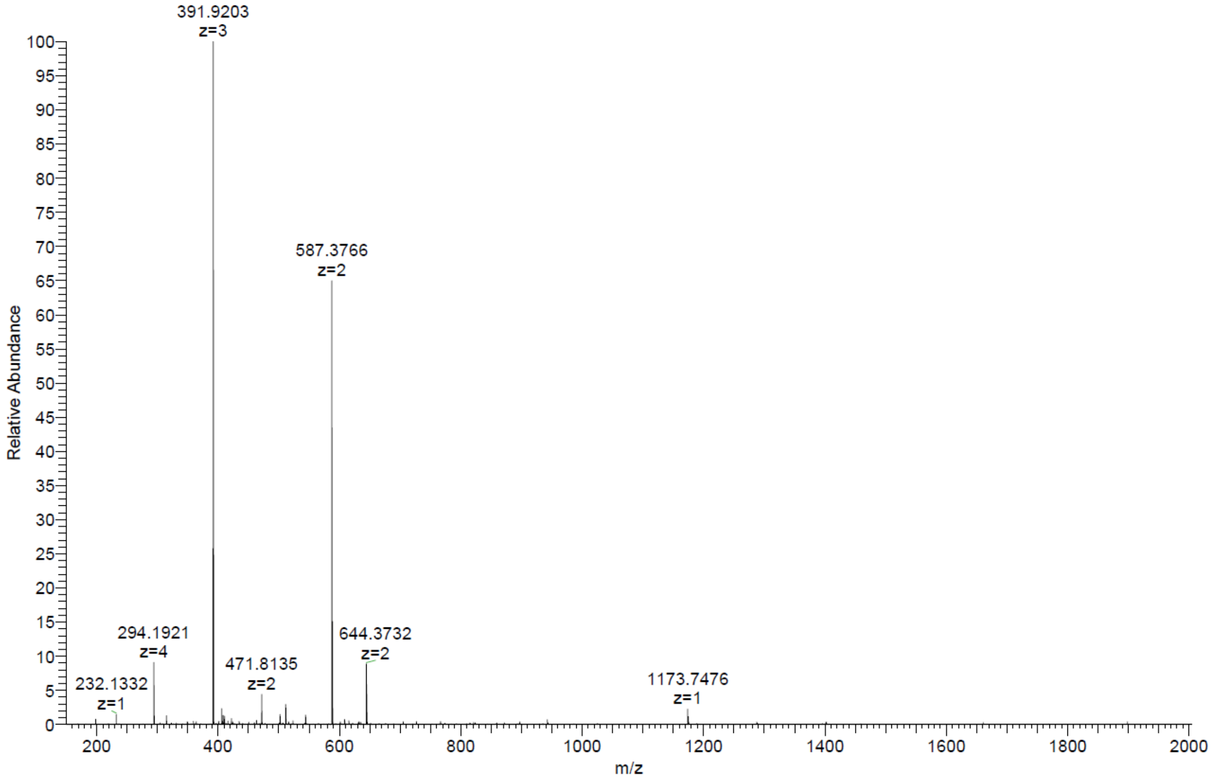
391.9203
z=3
587.3766
z=2
294.1921
z=4
644.3732
z=2
471.8135
z=2
232.1332
z=1
1173.7476
z=1
Relative Abundance
m/z

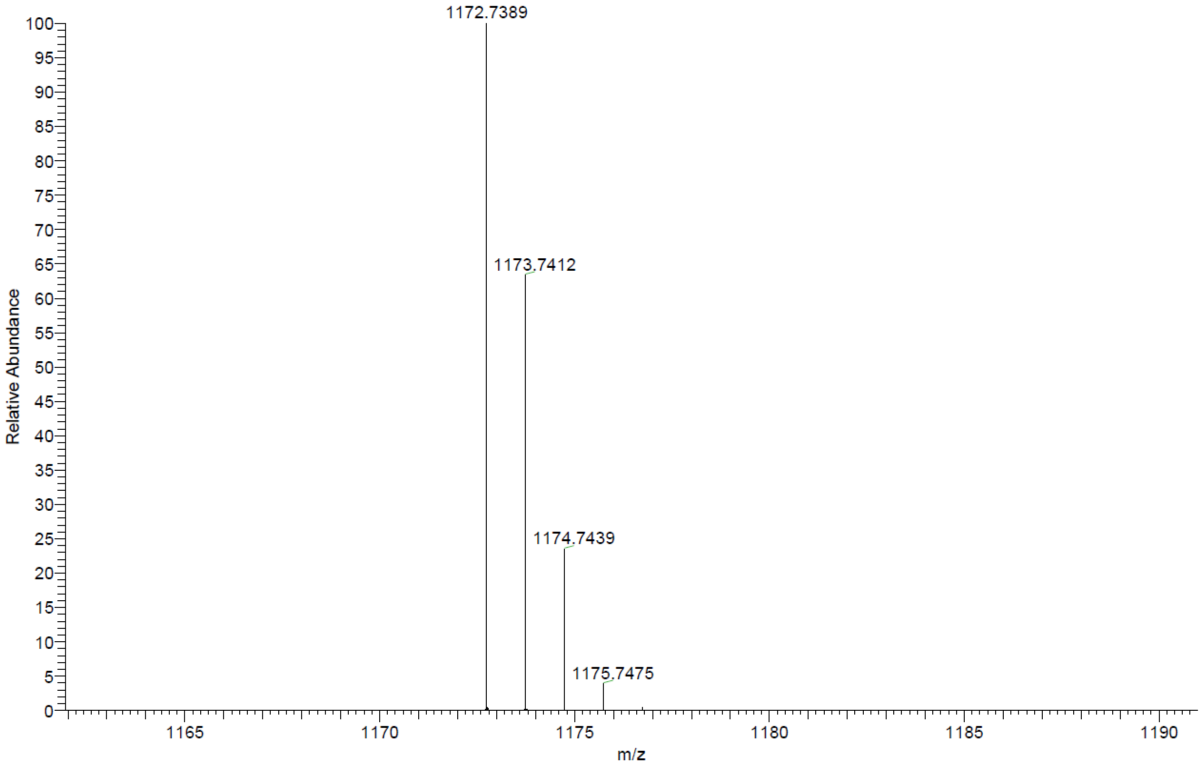
1172.7389
1173.7412
1174.7439
1175.7475
Relative Abundance
m/z

EB36 was obtained as a foamy white solid after preparative RP-HPLC (72 mg, 24 %). **Analytical RP-HPLC**: t_R = 3.41 min (A/D 100:0 to 0:100 in 7.0 min, λ = 214 nm). **MS** (ESI$^+$): $C_{56}H_{93}N_{15}O_{13}$ calc./obs. 1183.7077/1183.7083 Da [M].

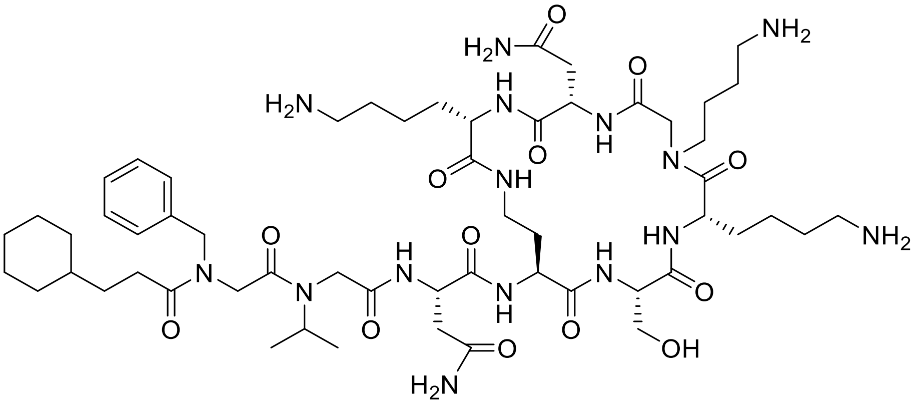

Chemical Formula: $C_{56}H_{93}N_{15}O_{13}$
Exact Mass: 1183.7077
Molecular Weight: 1184.4520

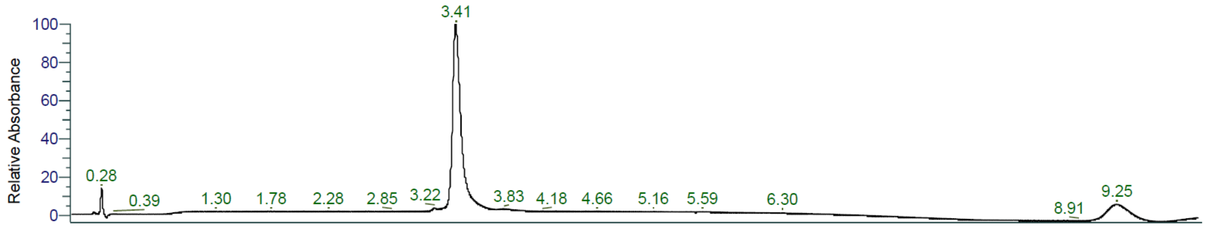

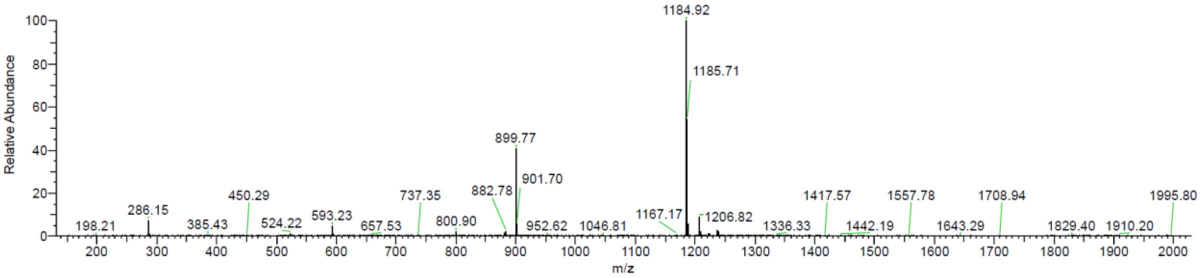

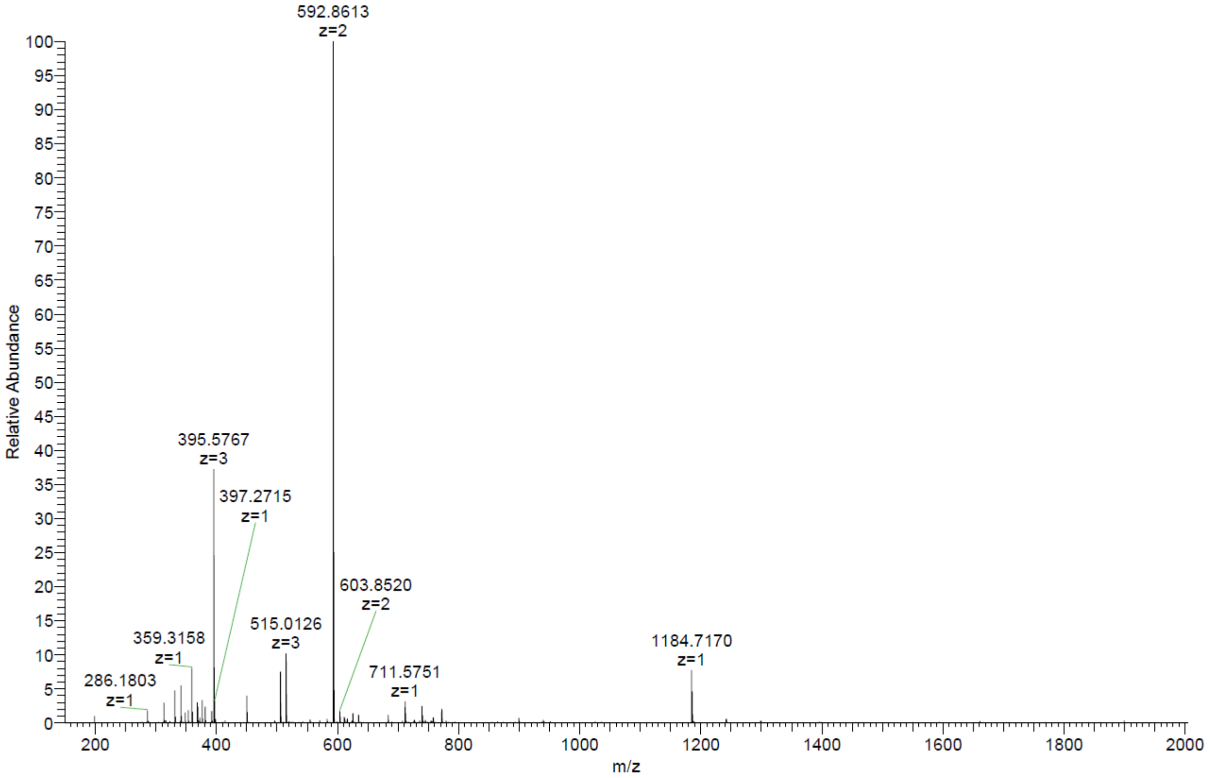
592.8613
z=2
395.5767
z=3
397.2715
z=1
603.8520
z=2
515.0126
z=3
359.3158
z=1
1184.7170
z=1
711.5751
z=1
286.1803
z=1
Relative Abundance
m/z

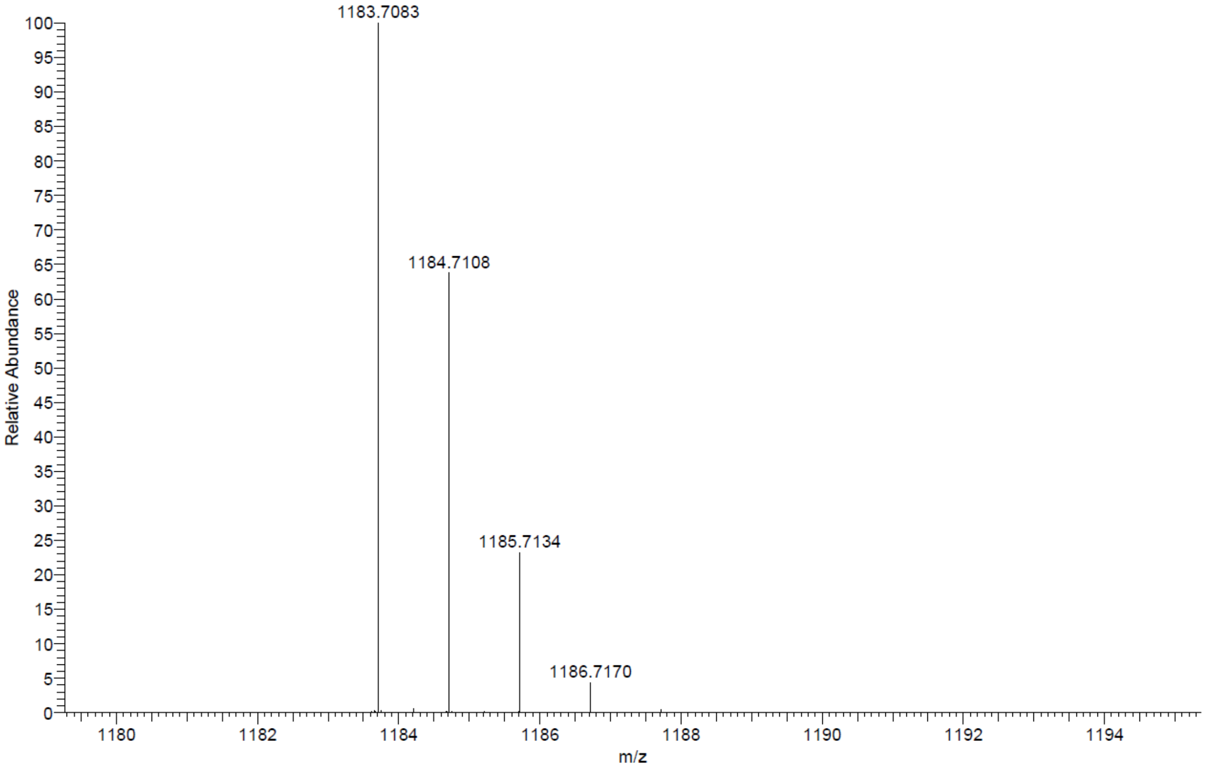
1183.7083
1184.7108
1185.7134
1186.7170
Relative Abundance
m/z

EB37 was obtained as a foamy white solid after preparative RP-HPLC (62 mg, 20 %). **Analytical RP-HPLC**: t_R = 3.47 min (A/D 100:0 to 0:100 in 7.0 min, λ = 214 nm). **MS** (ESI$^+$): $C_{60}H_{99}N_{15}O_{13}$ calc./obs. 1237.7547/1237.7549 Da [M].

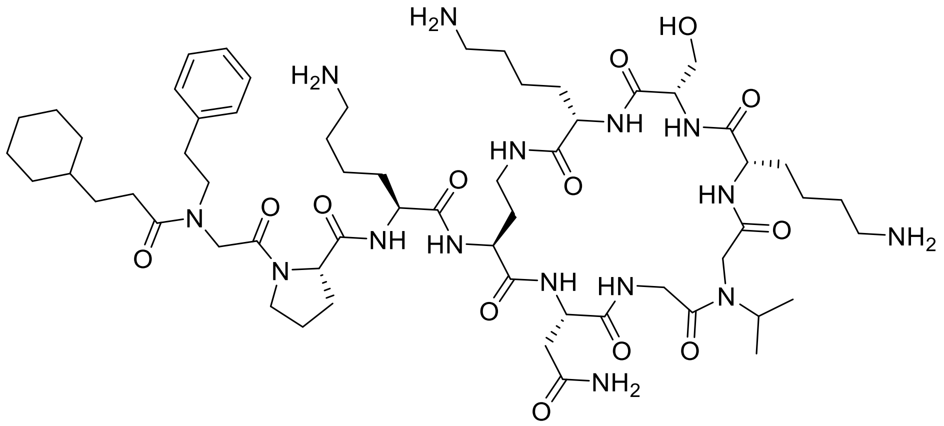

Chemical Formula: $C_{60}H_{99}N_{15}O_{13}$
Exact Mass: 1237.7547
Molecular Weight: 1238.5440

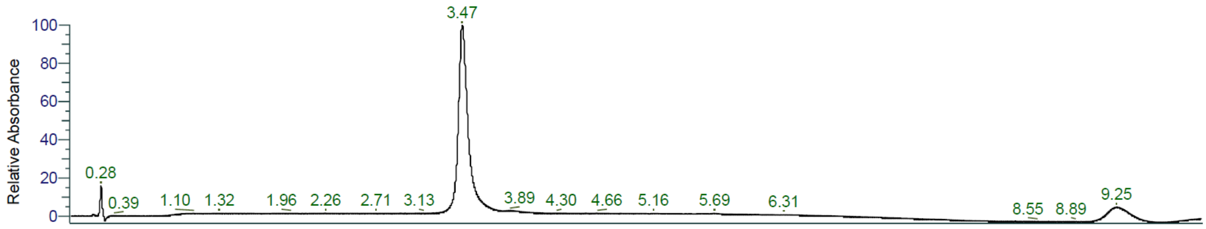

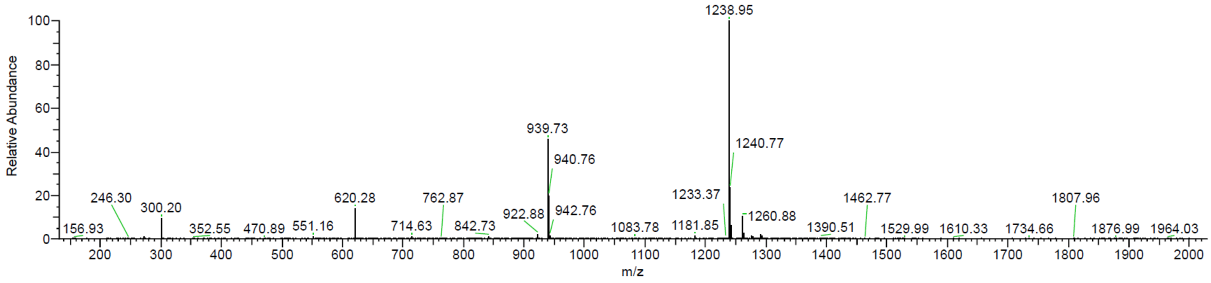

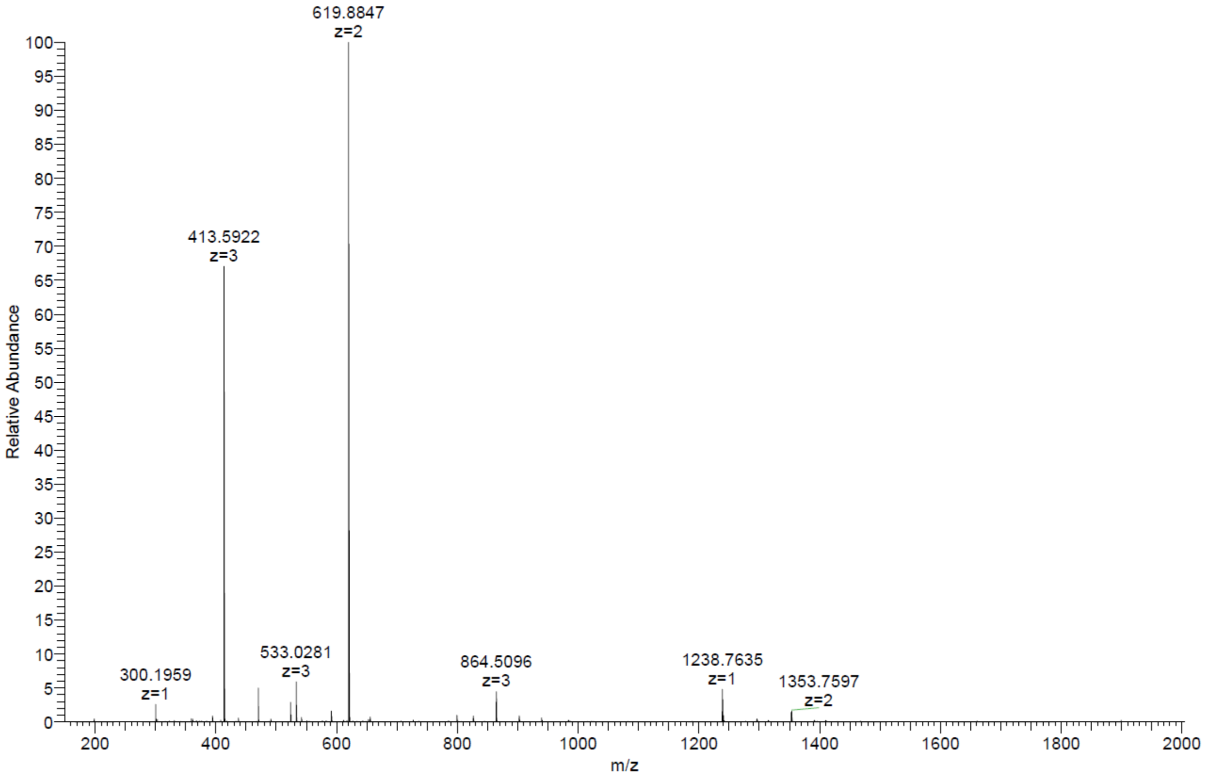
619.8847
z=2
413.5922
z=3
300.1959
z=1
533.0281
z=3
864.5096
z=3
1238.7635
z=1
1353.7597
z=2
Relative Abundance
m/z

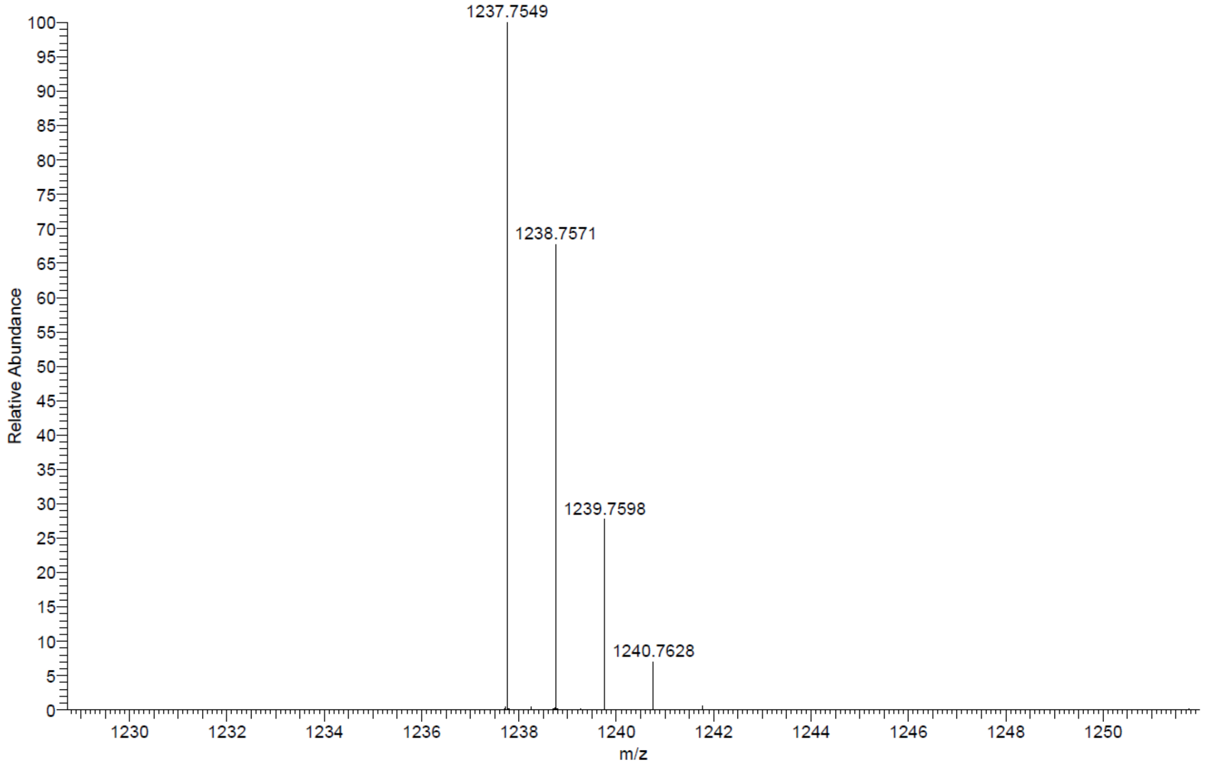
1237.7549
1238.7571
1239.7598
1240.7628
Relative Abundance
m/z

EB38 was obtained as a foamy white solid after preparative RP-HPLC (78 mg, 24 %). **Analytical RP-HPLC**: t_R = 2.01 min (A/D 100:0 to 0:100 in 7.0 min, λ = 214 nm). **MS** (ESI$^+$): $C_{55}H_{93}N_{17}O_{12}$ calc./obs. 1183.7190/1183.7191 Da [M].

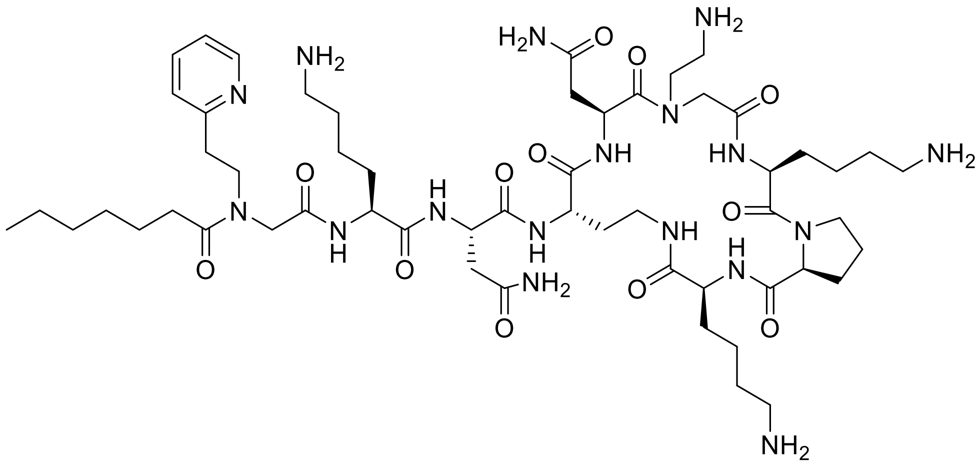

Chemical Formula: $C_{55}H_{93}N_{17}O_{12}$
Exact Mass: 1183.7190
Molecular Weight: 1184.4560

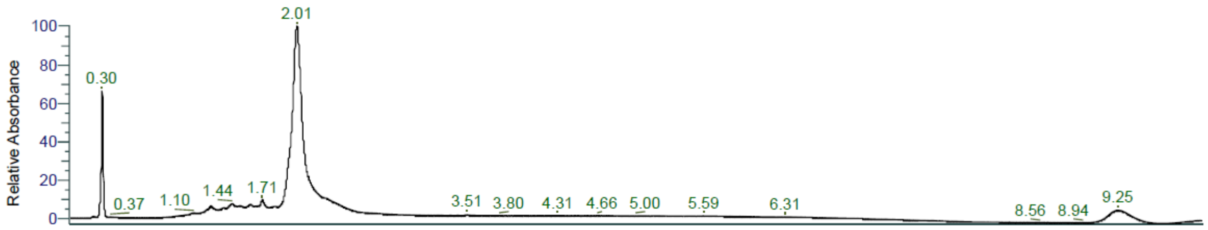

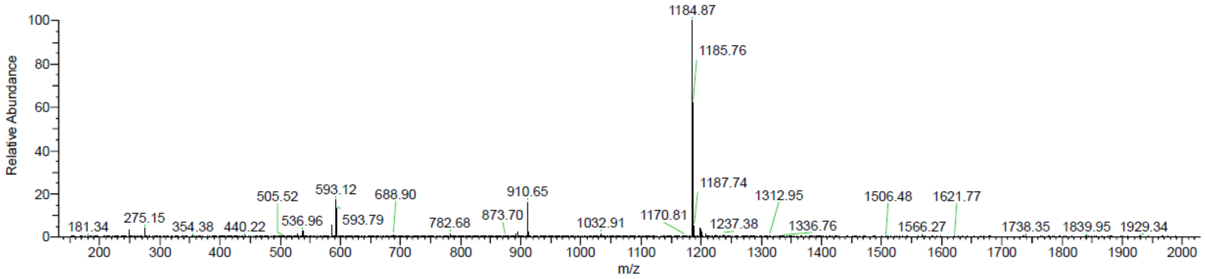

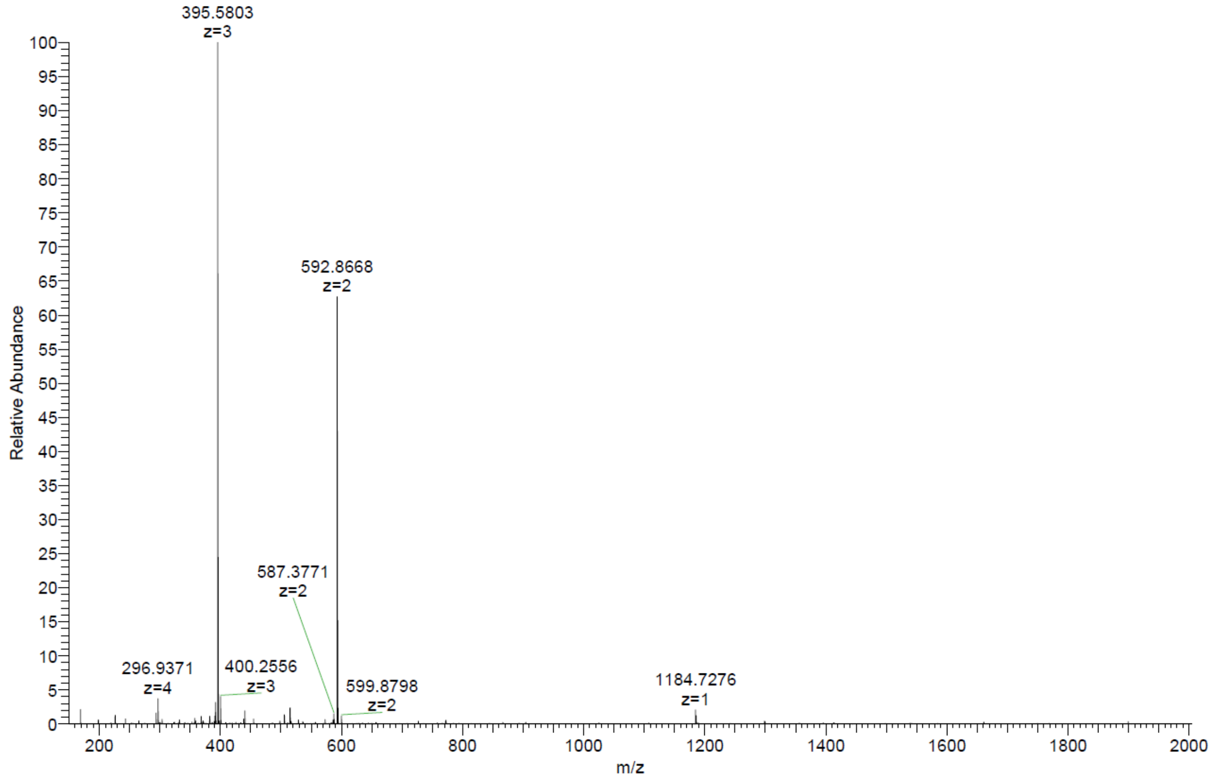
395.5803
z=3
592.8668
z=2
587.3771
z=2
296.9371
z=4
400.2556
z=3
599.8798
z=2
1184.7276
z=1
Relative Abundance
m/z

3

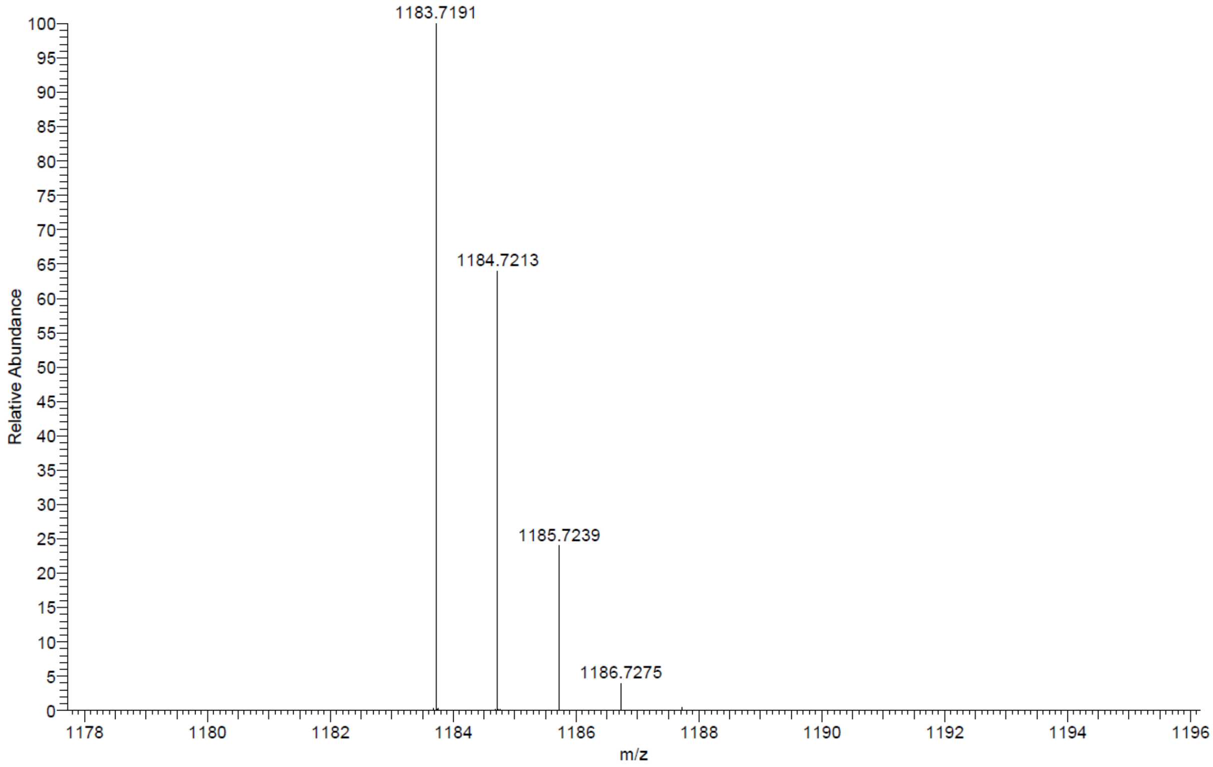
1183.7191
1184.7213
1185.7239
1186.7275
Relative Abundance
m/z

EB39 was obtained as a foamy white solid after preparative RP-HPLC (78 mg, 29 %). **Analytical RP-HPLC**: t_R = 2.16 min (A/D 100:0 to 0:100 in 7.0 min, λ = 214 nm). **MS** (ESI$^+$): $C_{56}H_{95}N_{17}O_{12}$ calc./obs. 1197.7346/1197.7384 Da [M].

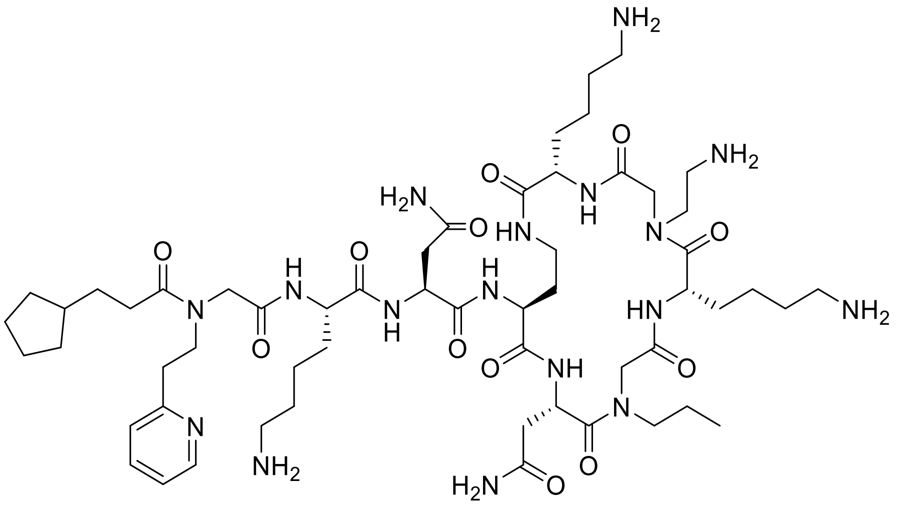

Chemical Formula: $C_{56}H_{95}N_{17}O_{12}$
Exact Mass: 1197.7346
Molecular Weight: 1198.4830

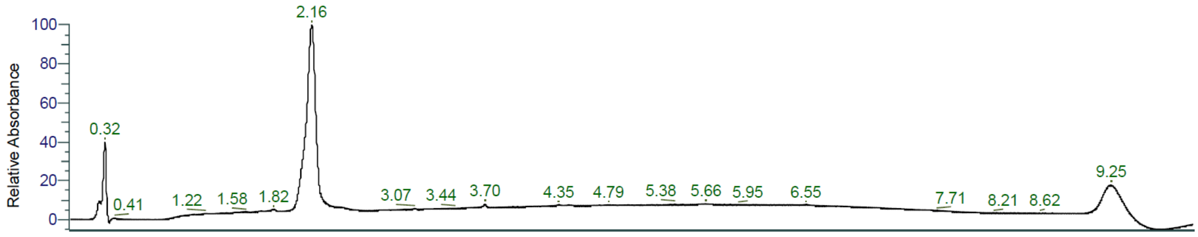

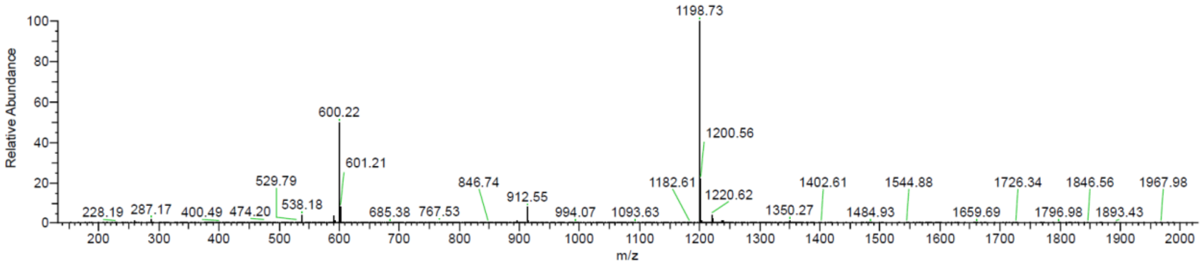

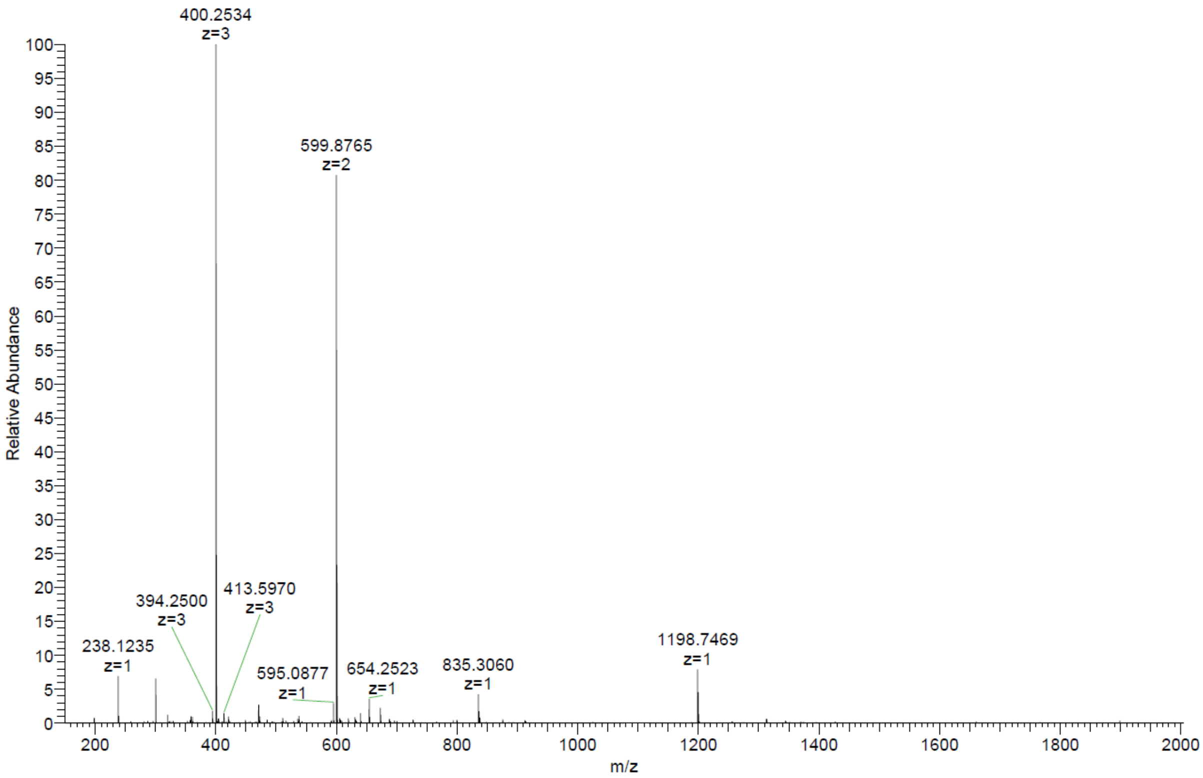

400.2534
z=3
599.8765
z=2
394.2500
z=3
413.5970
z=3
238.1235
z=1
595.0877
z=1
654.2523
z=1
835.3060
z=1
1198.7469
z=1
Relative Abundance
m/z

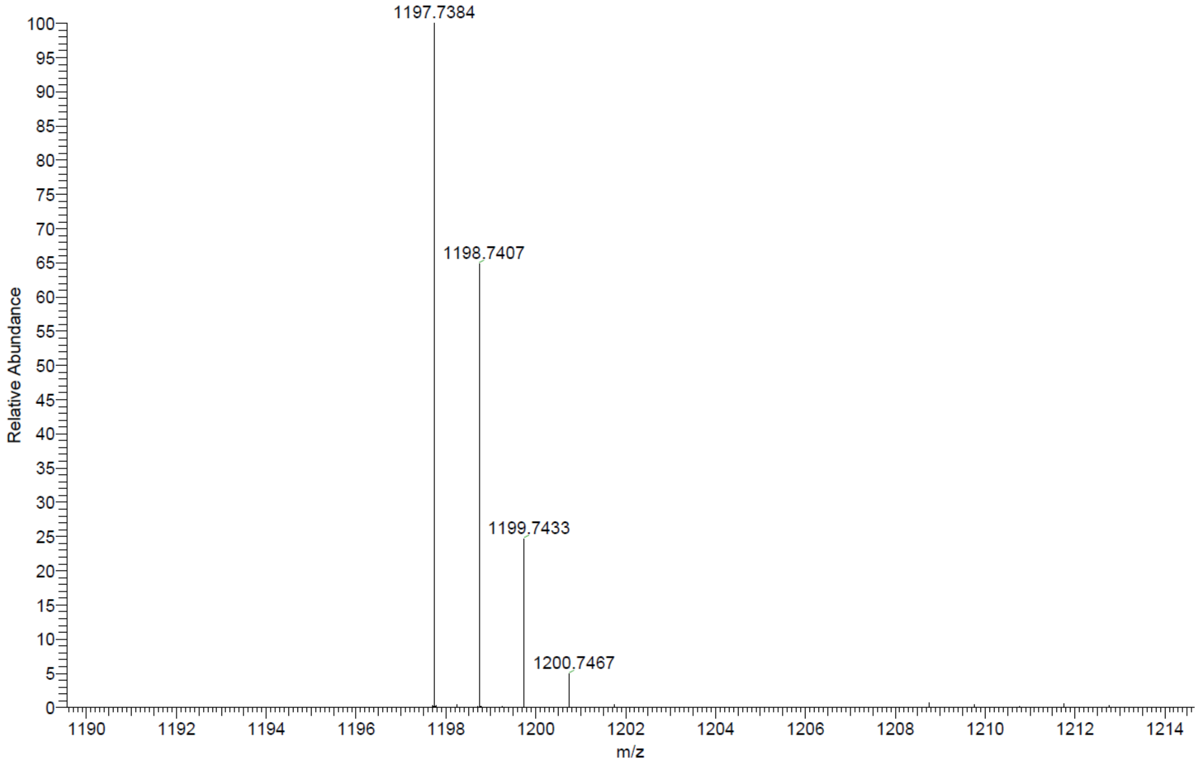

1197.7384
1198.7407
1199.7433
1200.7467
Relative Abundance
m/z

EB40 was obtained as a foamy white solid after preparative RP-HPLC (28 mg, 9 %). **Analytical RP-HPLC**: t_R = 2.50 min (A/D 100:0 to 0:100 in 7.0 min, λ = 214 nm). **MS** (ESI$^+$): $C_{57}H_{96}N_{16}O_{14}$ calc./obs. 1228.7292/1228.7279 Da [M].

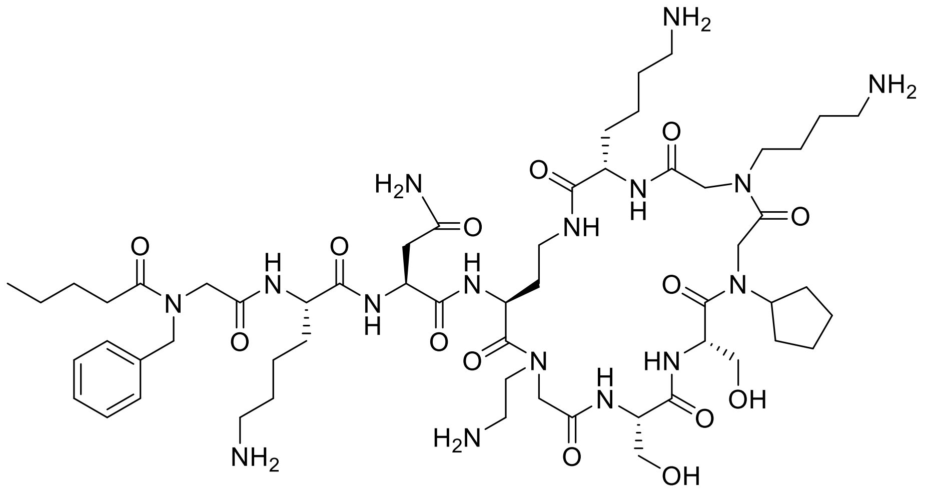

Chemical Formula: $C_{57}H_{96}N_{16}O_{14}$
Exact Mass: 1228.7292
Molecular Weight: 1229.4930

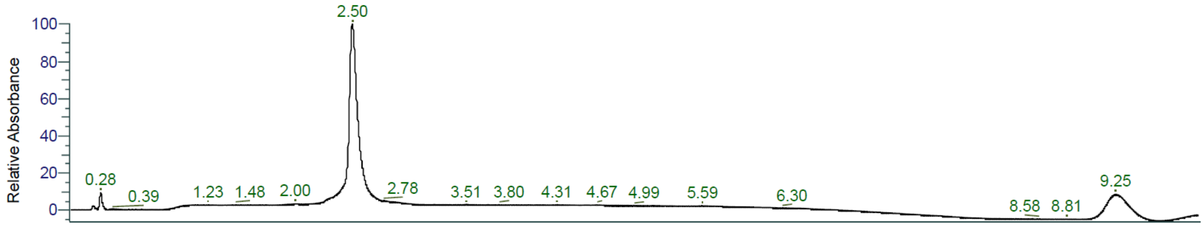

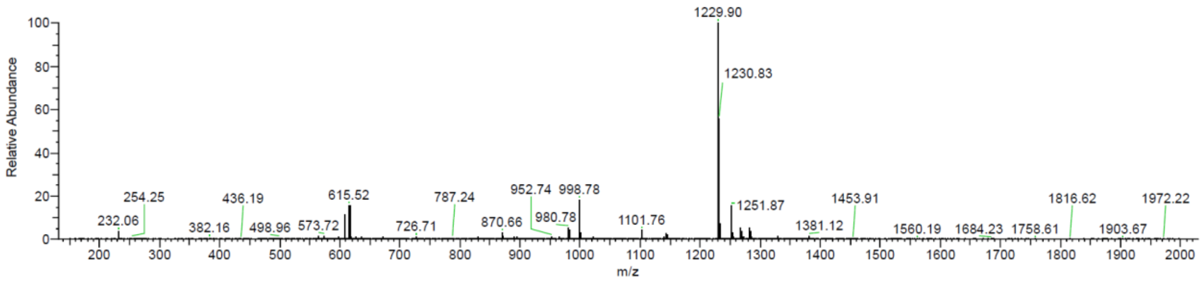

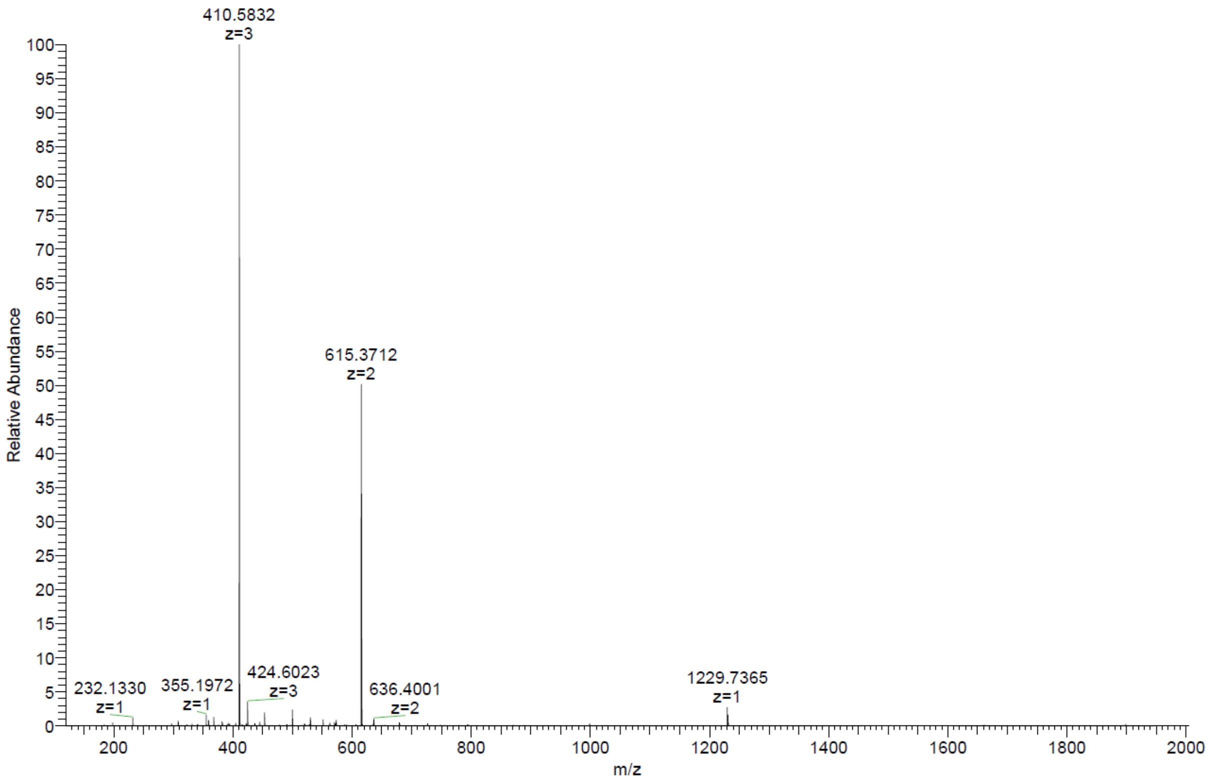
410.5832
z=3
615.3712
z=2
232.1330
z=1
355.1972
z=1
424.6023
z=3
636.4001
z=2
1229.7365
z=1
Relative Abundance
m/z

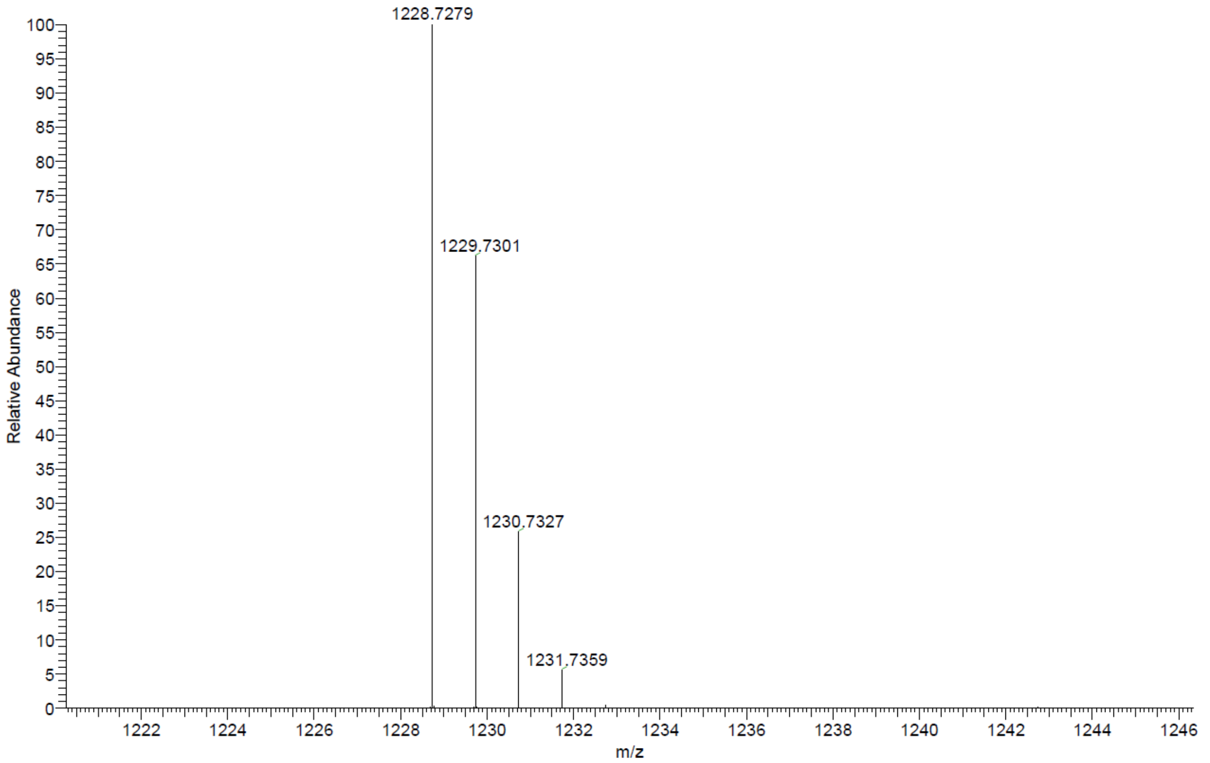
1228.7279
1229.7301
1230.7327
1231.7359
Relative Abundance
m/z

EB41 was obtained as a foamy white solid after preparative RP-HPLC (187 mg, 52 %). **Analytical RP-HPLC**: t_R = 1.92 min (A/D 100:0 to 0:100 in 7.0 min, λ = 214 nm). **MS** (ESI^+): $C_{54}H_{96}N_{18}O_{11}$ calc./obs. 1172.7506/1172.7511 Da [M].

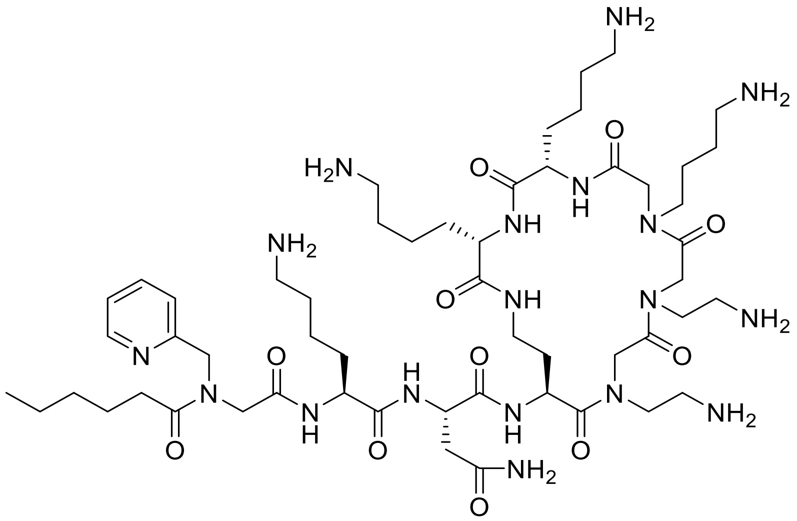

Chemical Formula: $C_{54}H_{96}N_{18}O_{11}$
Exact Mass: 1172.7506
Molecular Weight: 1173.4770

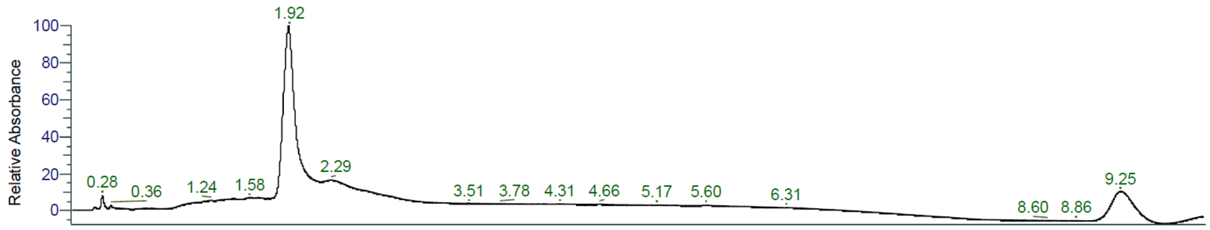

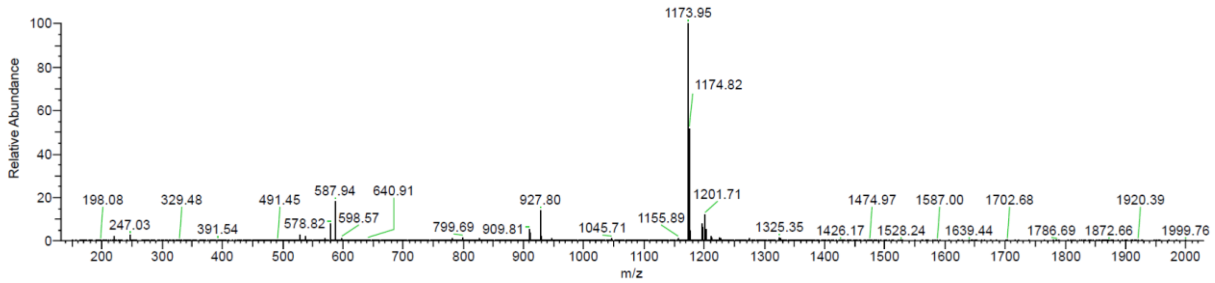

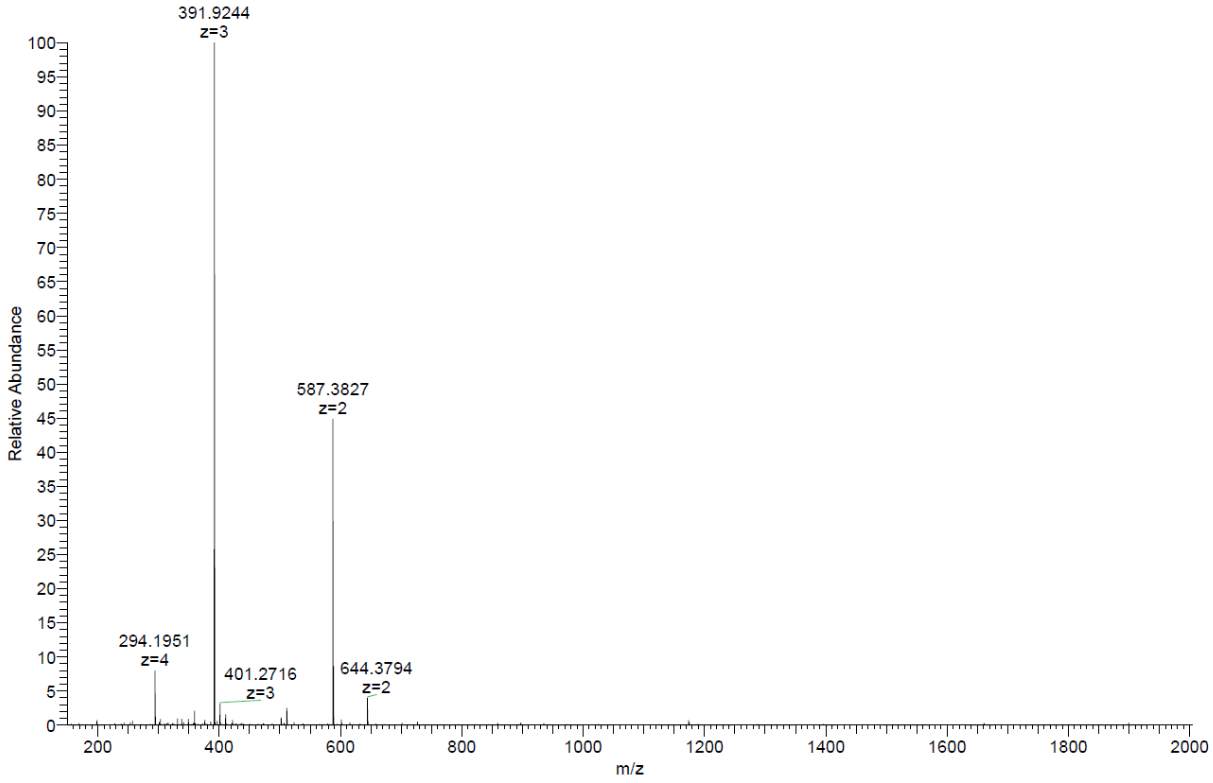
391.9244
z=3
587.3827
z=2
294.1951
z=4
401.2716
z=3
644.3794
z=2
Relative Abundance
m/z

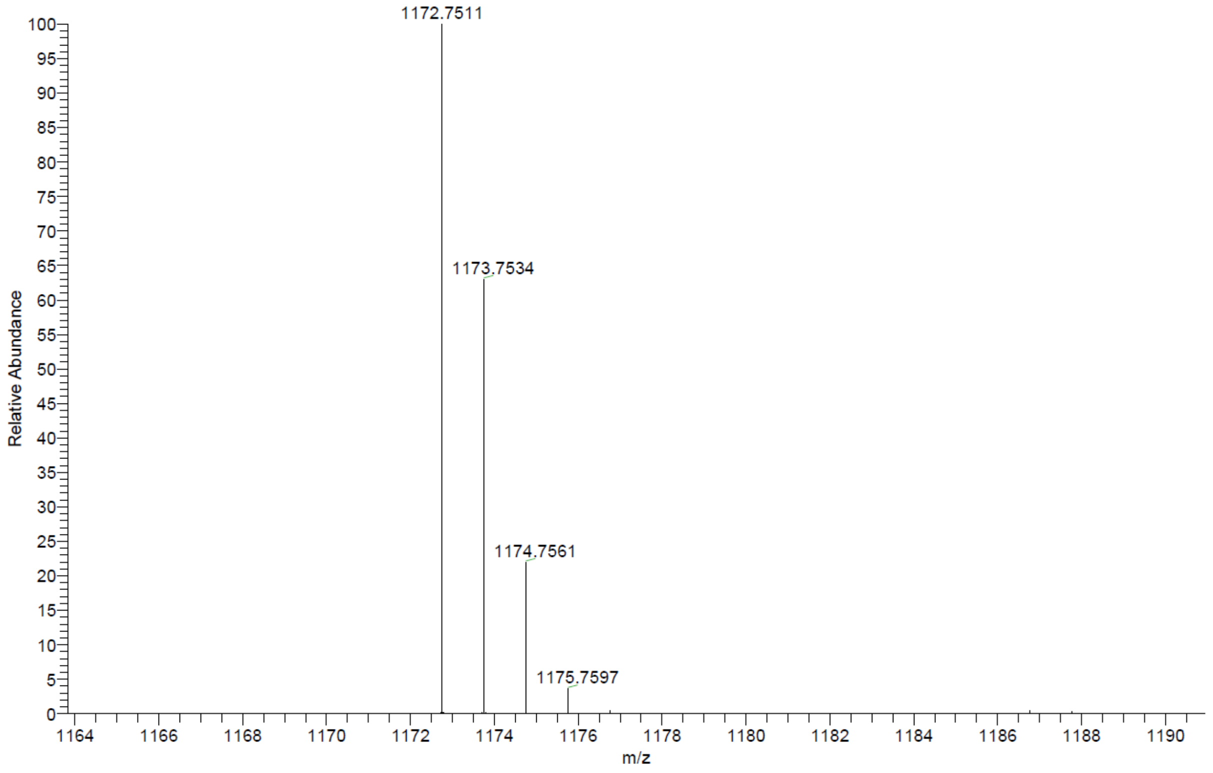
1172.7511
1173.7534
1174.7561
1175.7597
Relative Abundance
m/z

EB42 was obtained as a foamy white solid after preparative RP-HPLC (55 mg, 20 %). **Analytical RP-HPLC**: t_R = 1.77 min (A/D 100:0 to 0:100 in 7.0 min, λ = 214 nm). **MS** (ESI$^+$): $C_{48}H_{83}N_{17}O_{12}$ calc./obs. 1089.6407/1089.6415 Da [M].

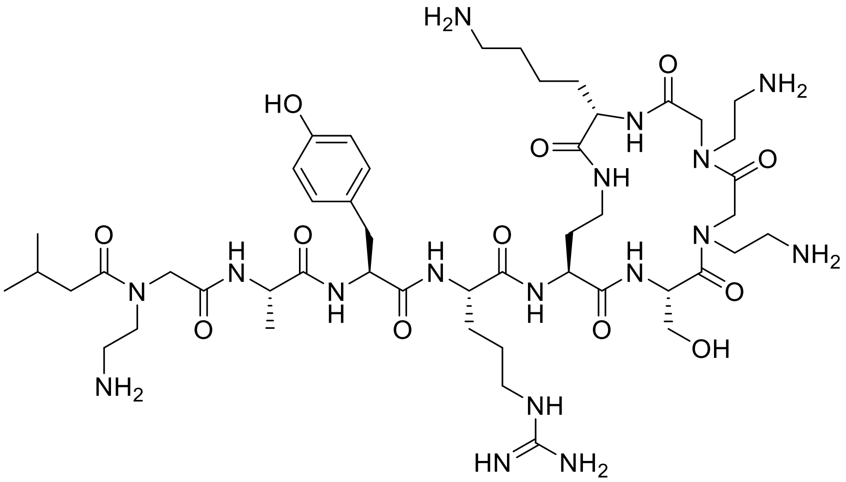

Chemical Formula: $C_{48}H_{83}N_{17}O_{12}$
Exact Mass: 1089.6407
Molecular Weight: 1090.2990

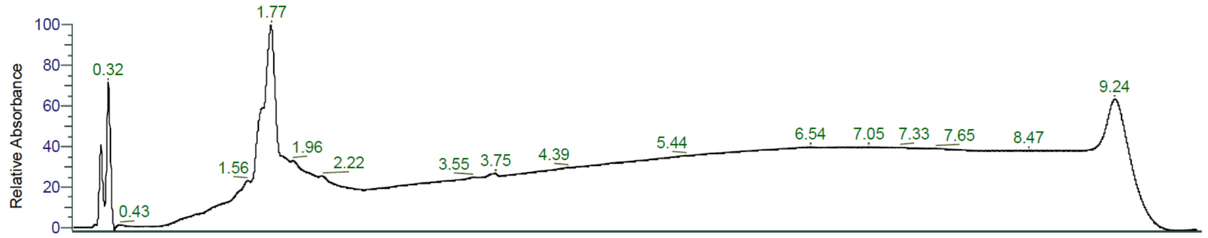

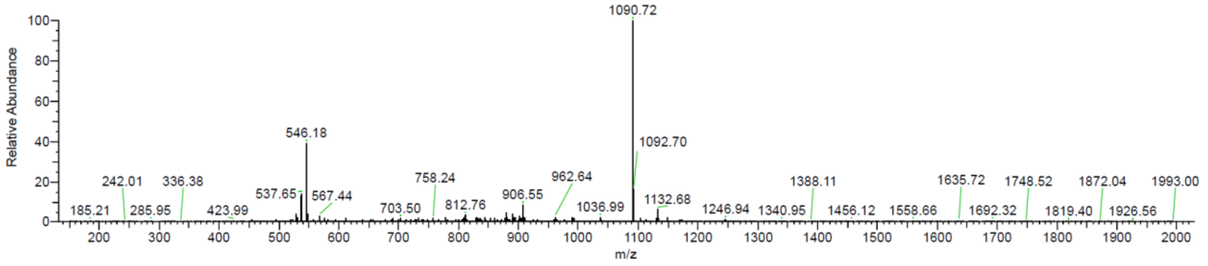

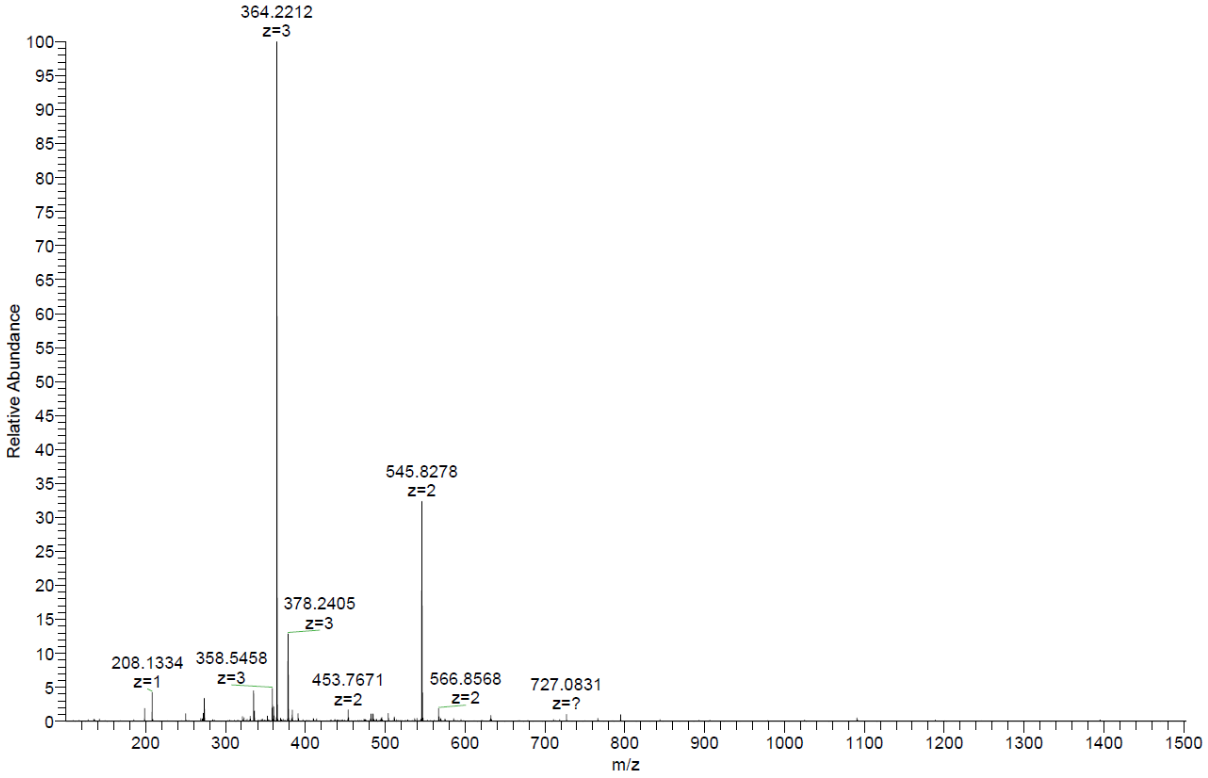
364.2212
z=3
545.8278
z=2
378.2405
z=3
208.1334
z=1
358.5458
z=3
453.7671
z=2
566.8568
z=2
727.0831
z=?
Relative Abundance
m/z

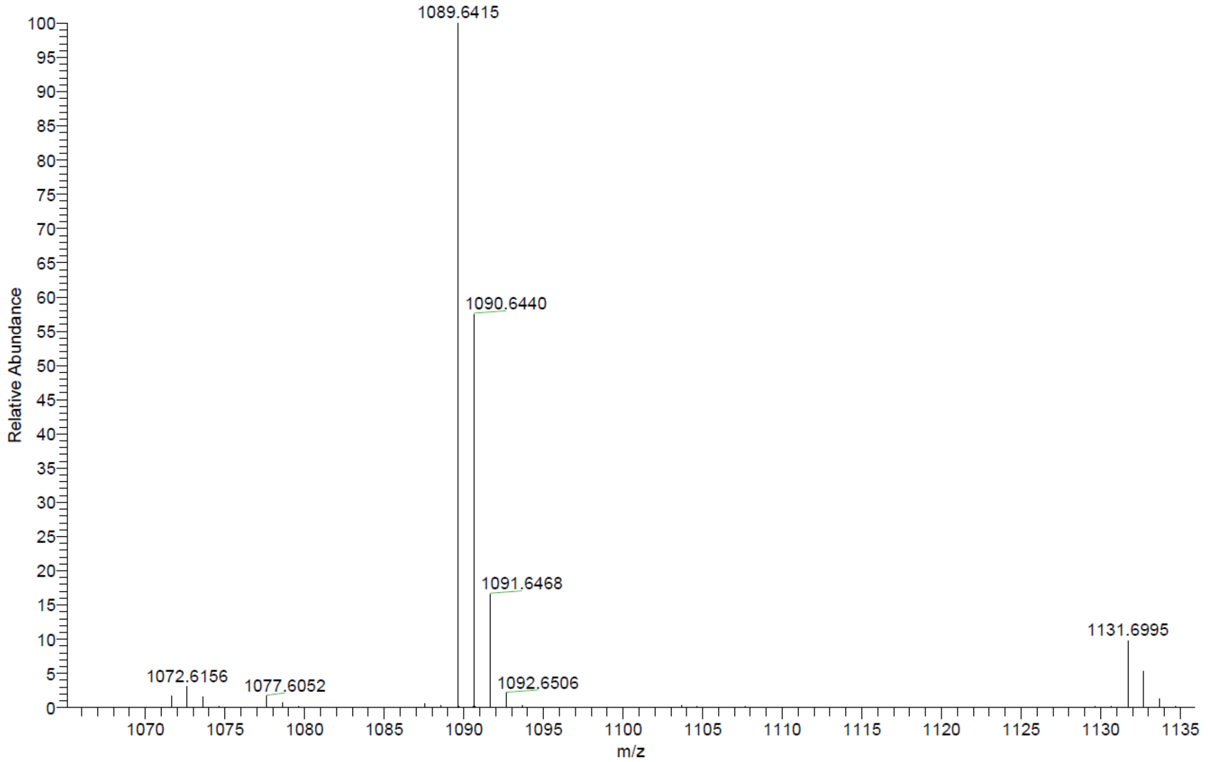
1089.6415
1090.6440
1091.6468
1131.6995
1072.6156
1077.6052
1092.6506
Relative Abundance
m/z

EB43 was obtained as a foamy white solid after preparative RP-HPLC (46 mg, 16 %). **Analytical RP-HPLC**: t_R = 1.70 min (A/D 100:0 to 0:100 in 7.0 min, λ = 214 nm). **MS** (ESI$^+$): $C_{54}H_{95}N_{19}O_{11}$ calc./obs. 1185.7458/1185.7498 Da [M].

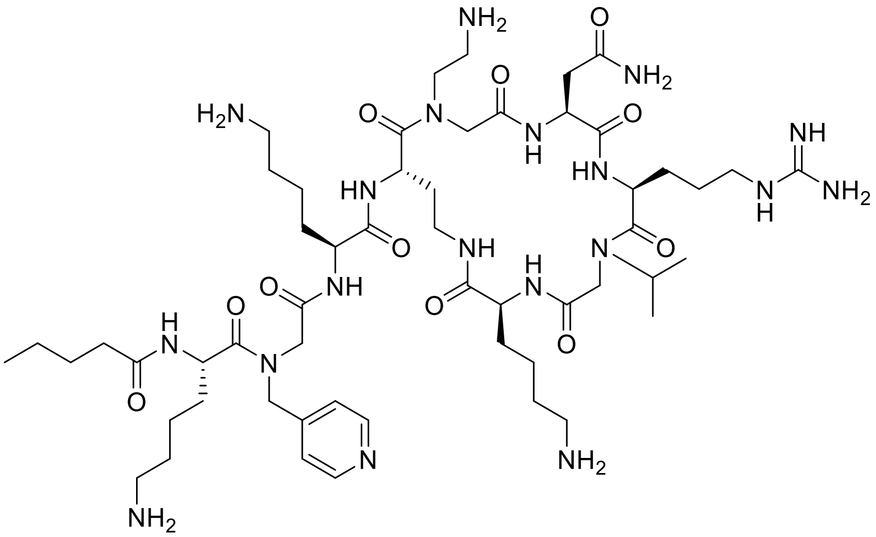

Chemical Formula: $C_{54}H_{95}N_{19}O_{11}$
Exact Mass: 1185.7458
Molecular Weight: 1186.4760

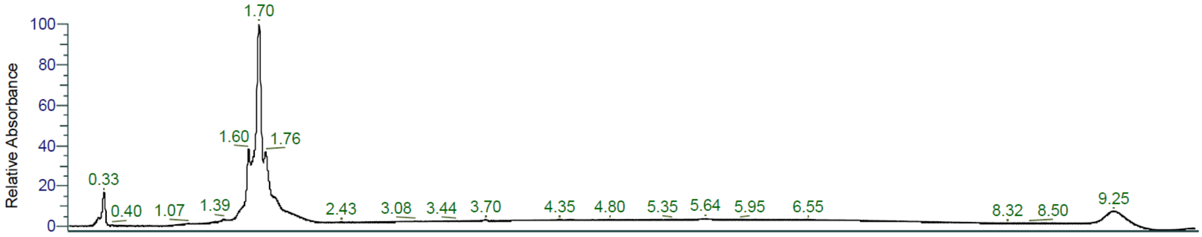

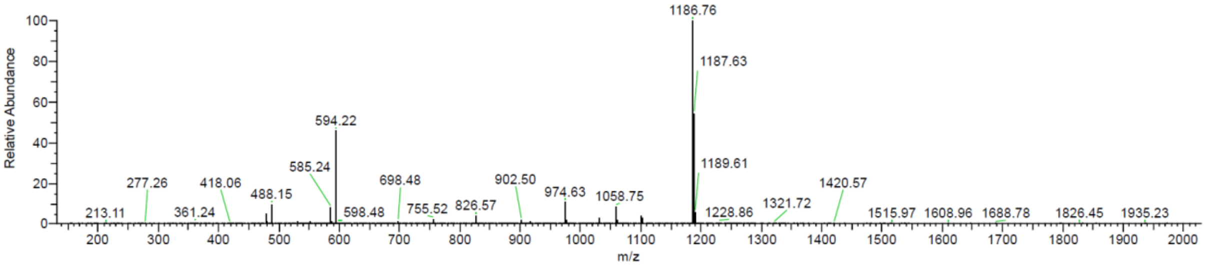

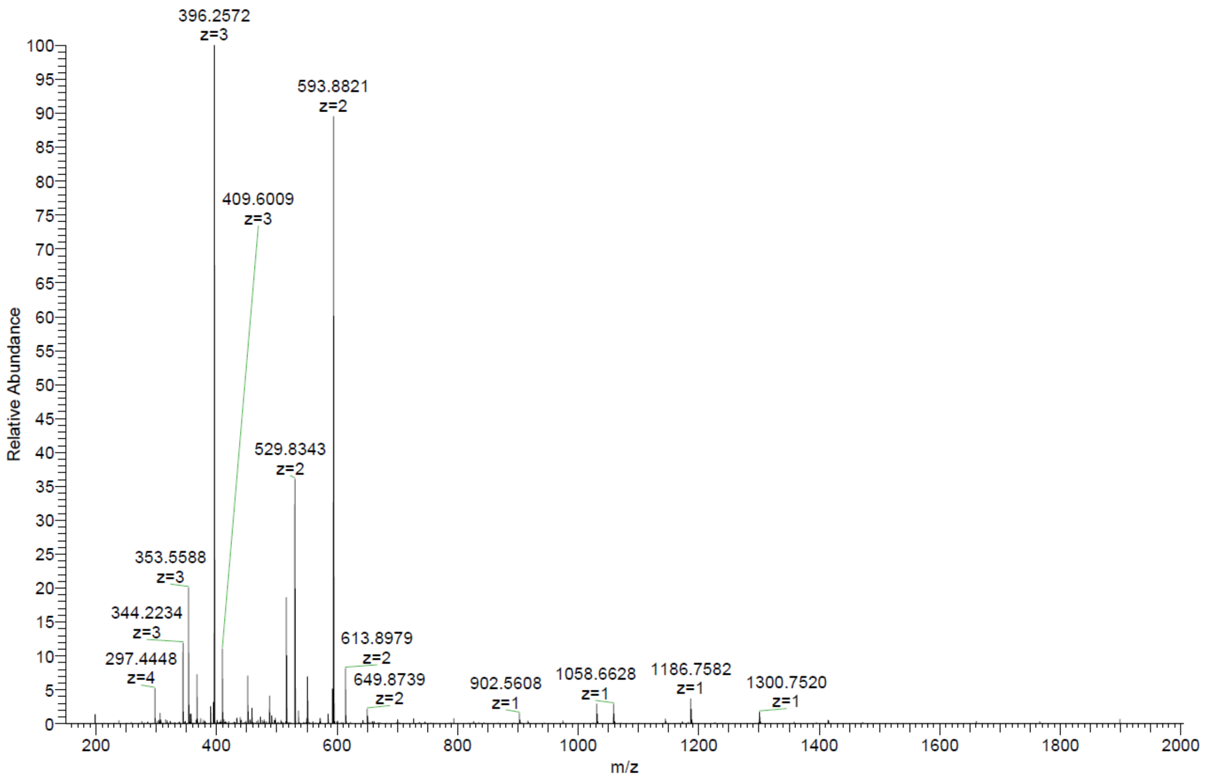

396.2572 z=3
593.8821 z=2
409.6009 z=3
529.8343 z=2
353.5588 z=3
344.2234 z=3
297.4448 z=4
613.8979 z=2
649.8739 z=2
902.5608 z=1
1058.6628 z=1
1186.7582 z=1
1300.7520 z=1
Relative Abundance
m/z

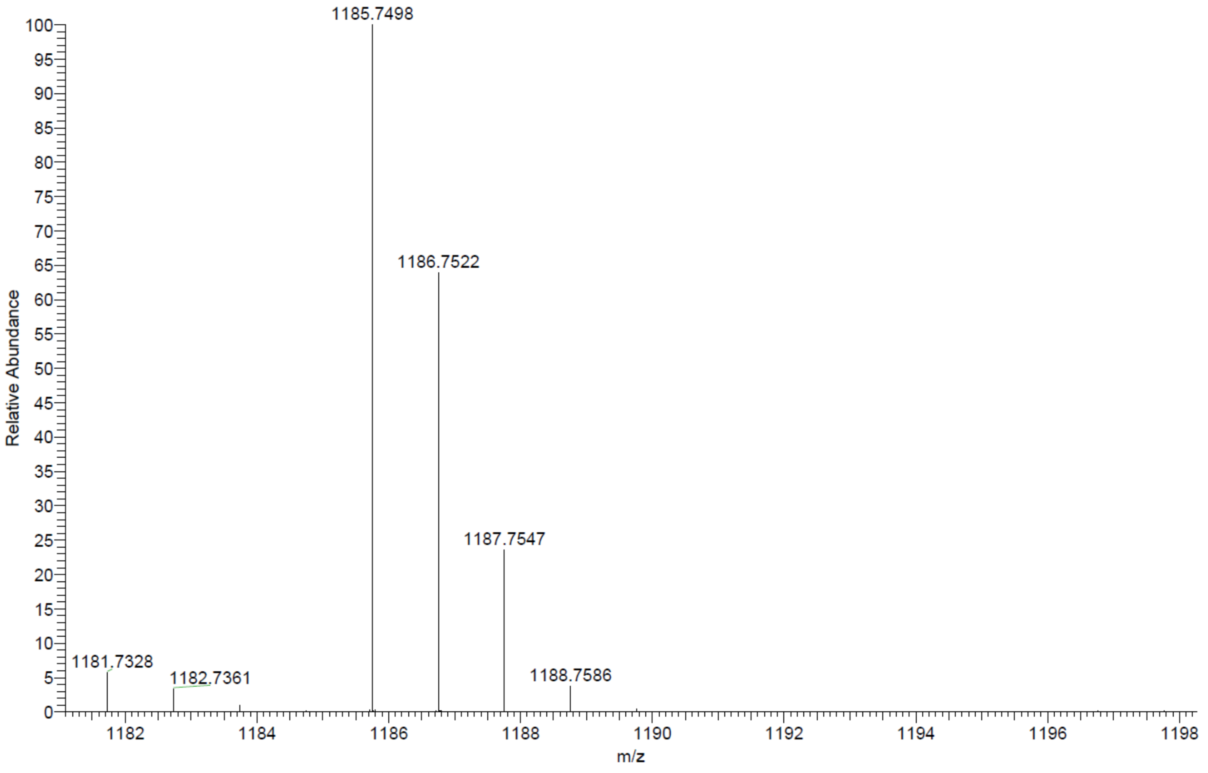

1185.7498
1186.7522
1187.7547
1181.7328
1182.7361
1188.7586
Relative Abundance
m/z

EB44 was obtained as a foamy white solid after preparative RP-HPLC (32 mg, 15 %). **Analytical RP-HPLC**: t_R = 2.40 min (A/D 100:0 to 0:100 in 7.0 min, λ = 214 nm). **MS** (ESI$^+$): $C_{52}H_{89}N_{15}O_{12}$ calc./obs. 1116.6815/1116.6929 Da [M].

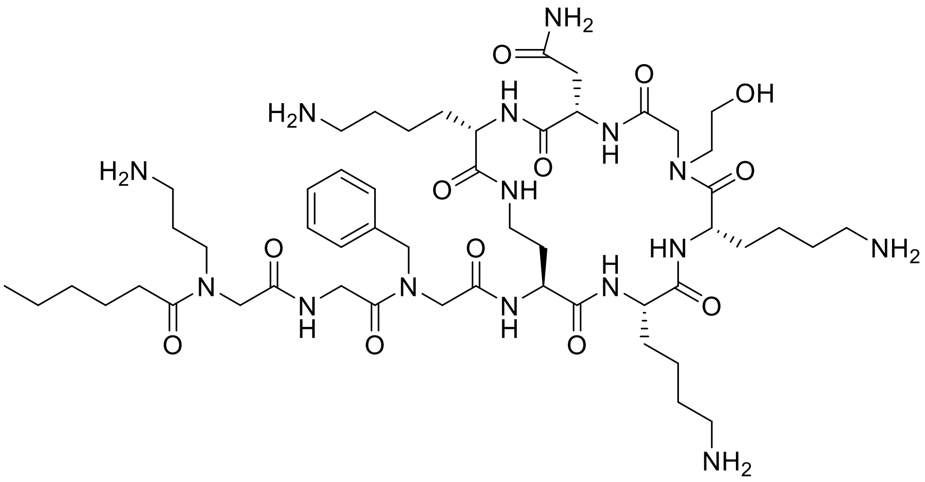

Chemical Formula: $C_{52}H_{89}N_{15}O_{12}$
Exact Mass: 1115.6815
Molecular Weight: 1116.3770

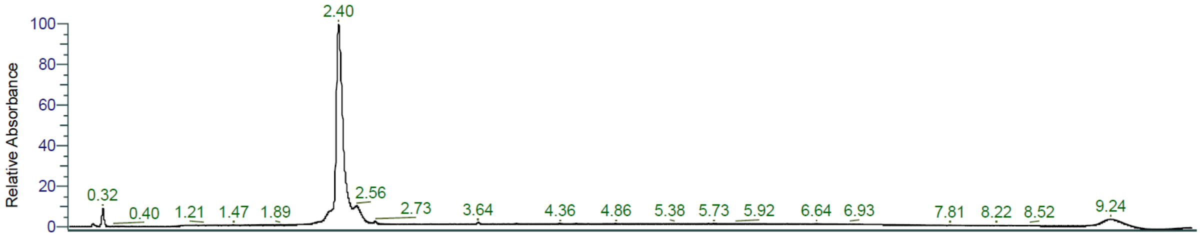

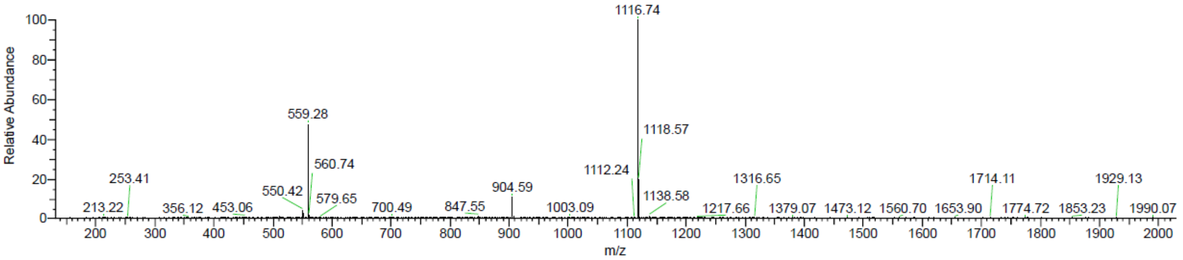

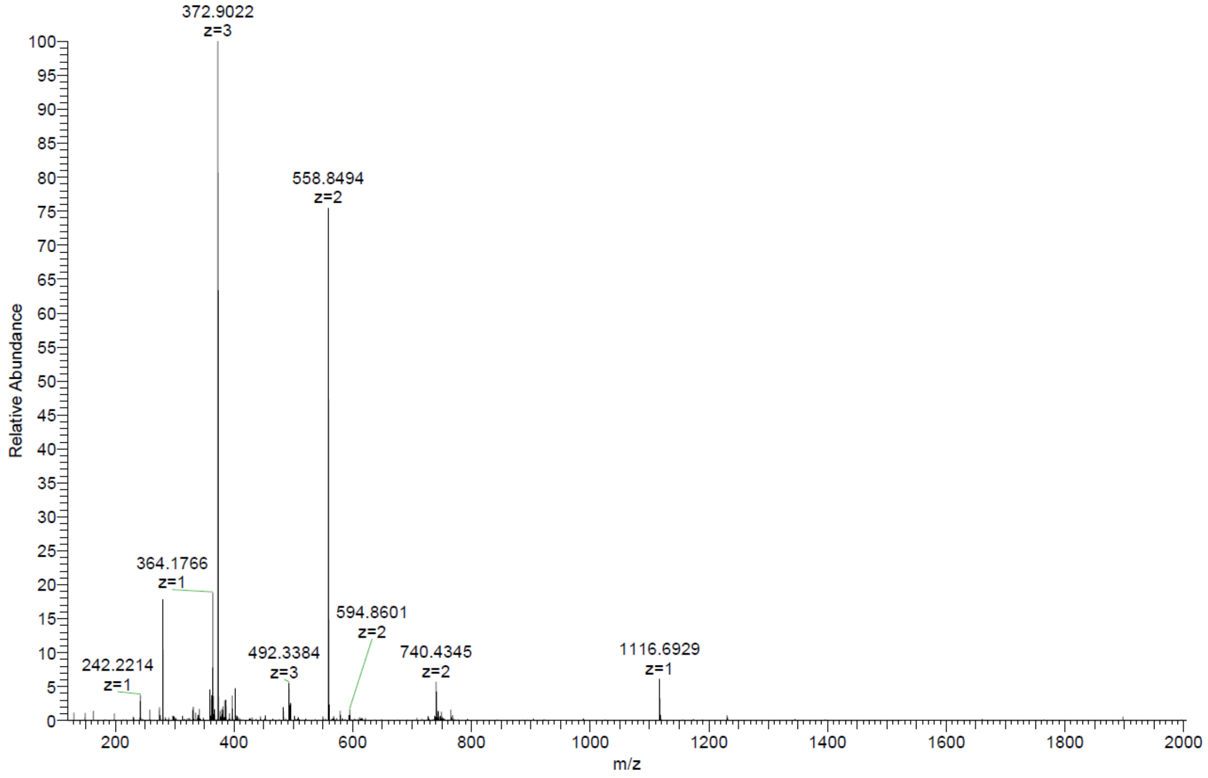
372.9022
z=3
558.8494
z=2
364.1766
z=1
594.8601
z=2
492.3384
z=3
740.4345
z=2
1116.6929
z=1
242.2214
z=1
Relative Abundance
m/z

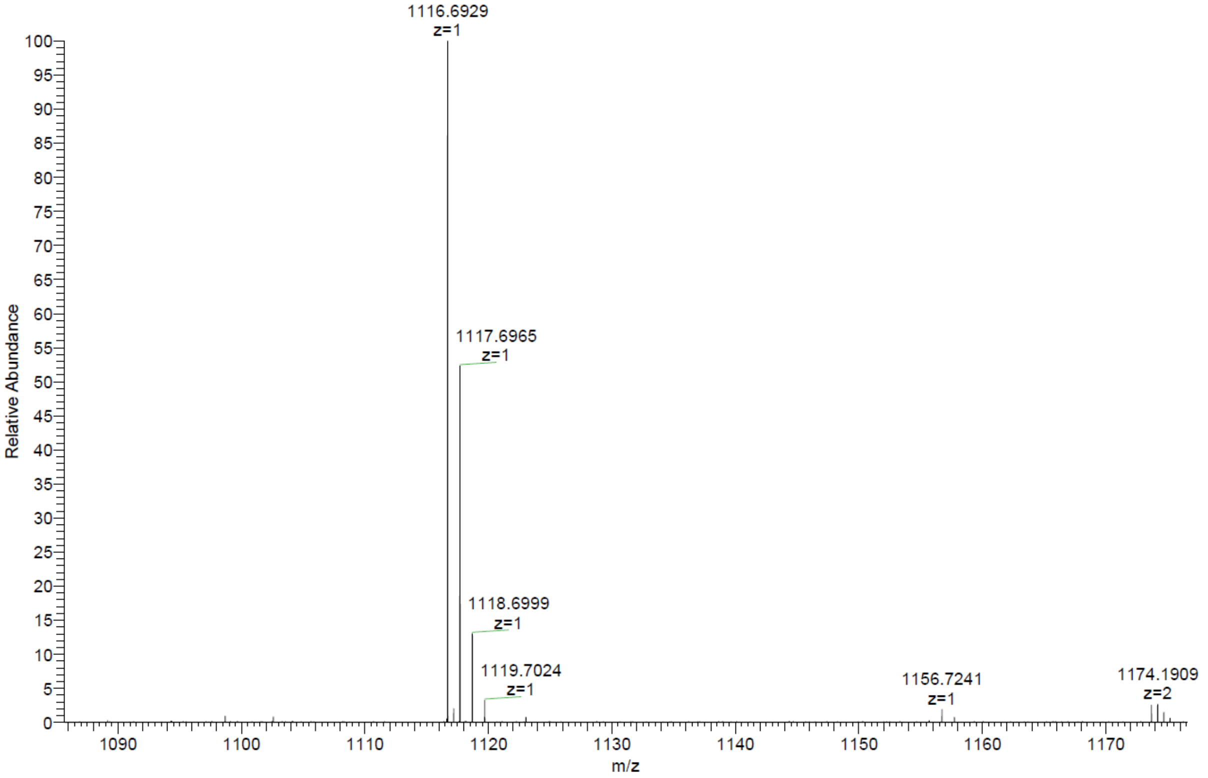
1116.6929
z=1
1117.6965
z=1
1118.6999
z=1
1119.7024
z=1
1156.7241
z=1
1174.1909
z=2
Relative Abundance
m/z

EB45 was obtained as a foamy white solid after preparative RP-HPLC (42 mg, 17 %). **Analytical RP-HPLC**: t_R = 3.30 min (A/D 100:0 to 0:100 in 7.0 min, λ = 214 nm). **MS** (ESI$^+$): $C_{54}H_{91}N_{15}O_{12}$ calc./obs. 1141.6972/1141.7009 Da [M].

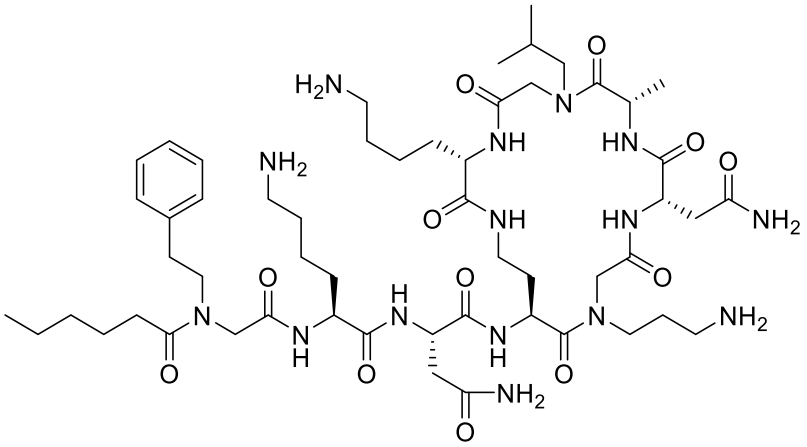

Chemical Formula: $C_{54}H_{91}N_{15}O_{12}$
Exact Mass: 1141.6972
Molecular Weight: 1142.4150

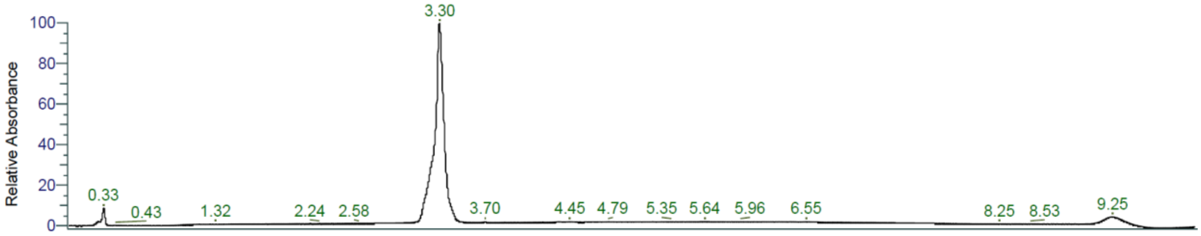

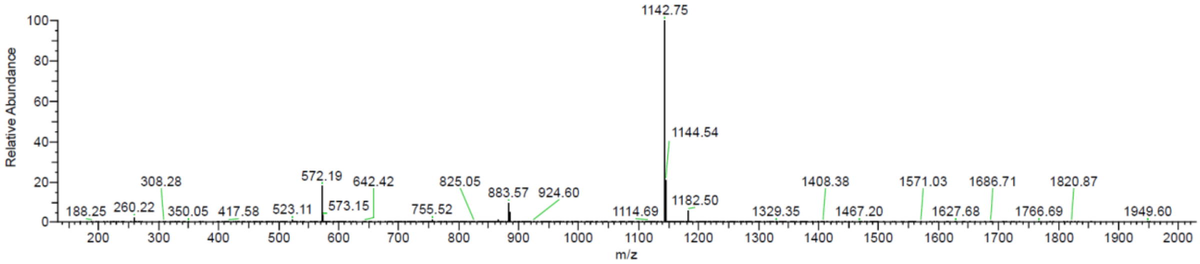

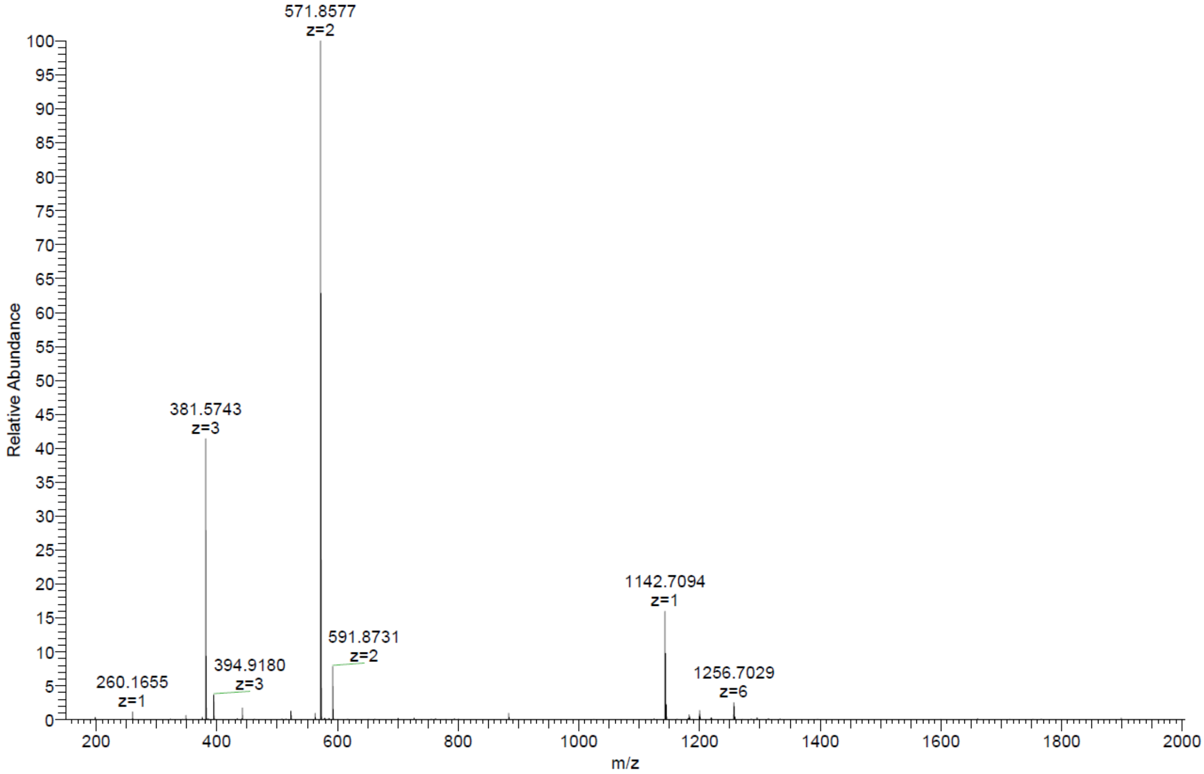
571.8577
z=2
381.5743
z=3
394.9180
z=3
591.8731
z=2
260.1655
z=1
1142.7094
z=1
1256.7029
z=6
Relative Abundance
m/z

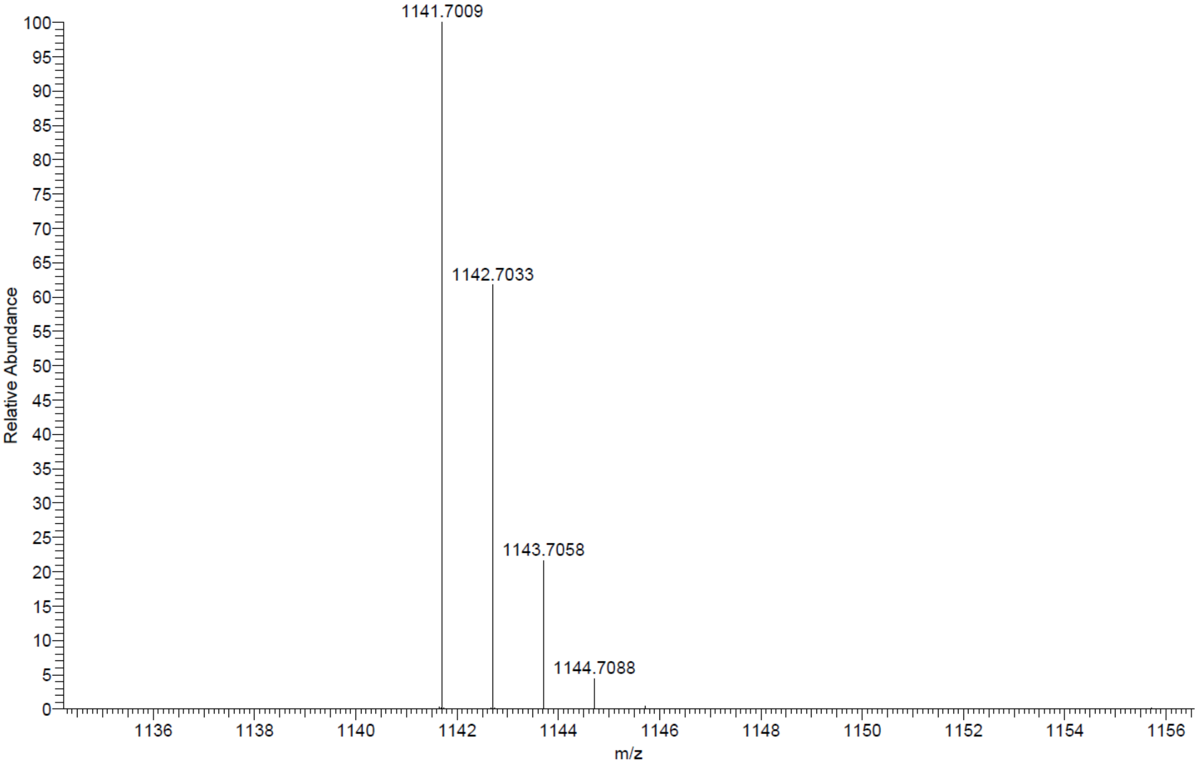
1141.7009
1142.7033
1143.7058
1144.7088
Relative Abundance
m/z

EB46 was obtained as a foamy white solid after preparative RP-HPLC (30 mg, 10 %).

Analytical RP-HPLC: t_R = 1.53 min (A/D 90:10 to 10:90 in 7.0 min, λ = 214 nm). **MS** (ESI$^+$): $C_{54}H_{91}N_{15}O_{12}$ calc./obs. 1141.6972/1141.6969 Da [M].

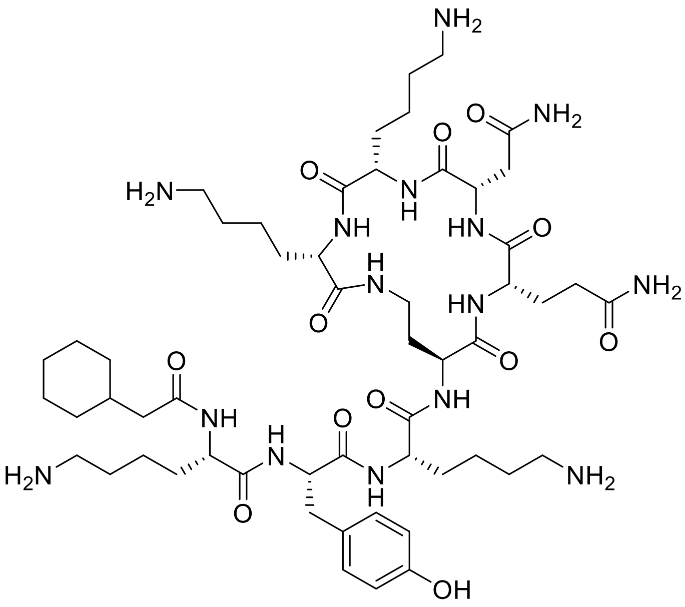

Chemical Formula: $C_{54}H_{91}N_{15}O_{12}$
Exact Mass: 1141.6972
Molecular Weight: 1142.4150

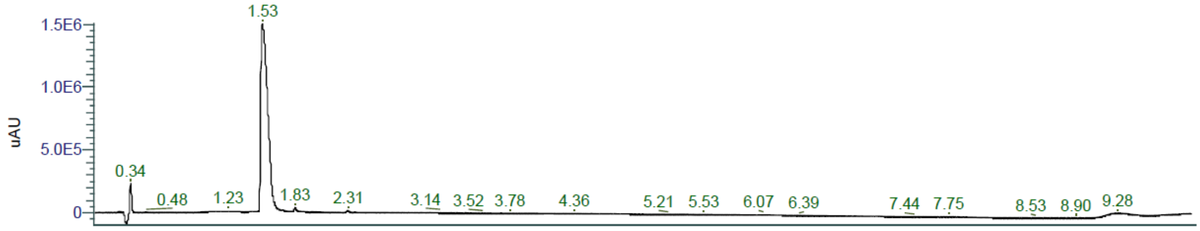

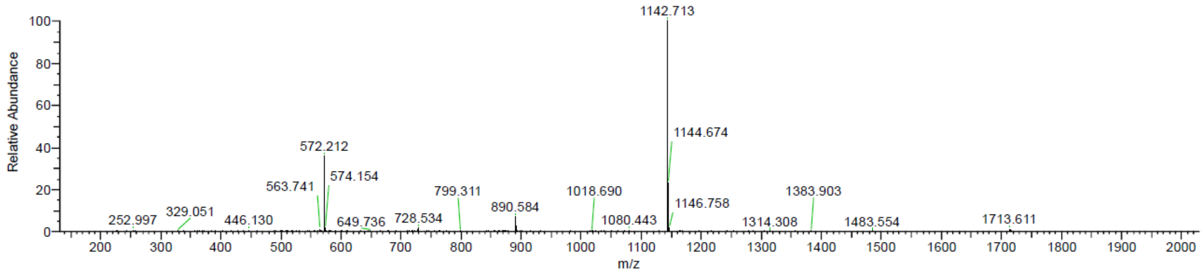

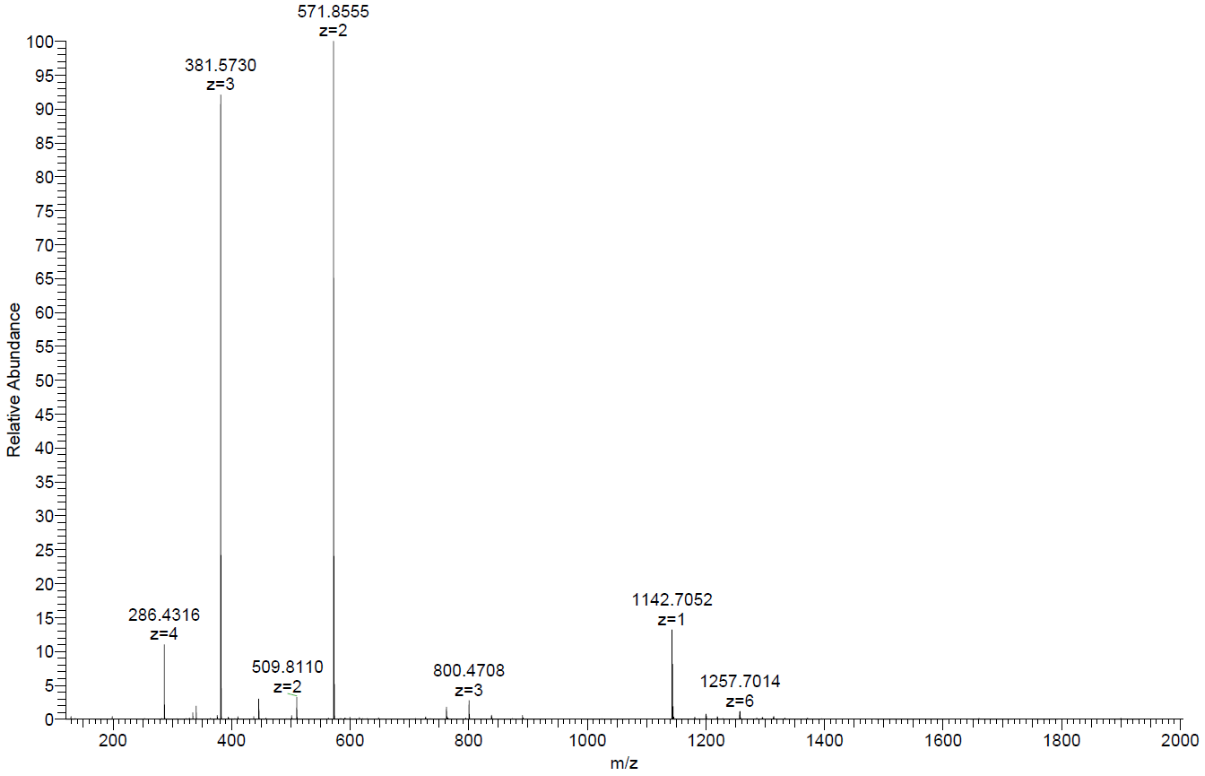

571.8555
z=2
381.5730
z=3
286.4316
z=4
509.8110
z=2
800.4708
z=3
1142.7052
z=1
1257.7014
z=6
Relative Abundance
m/z

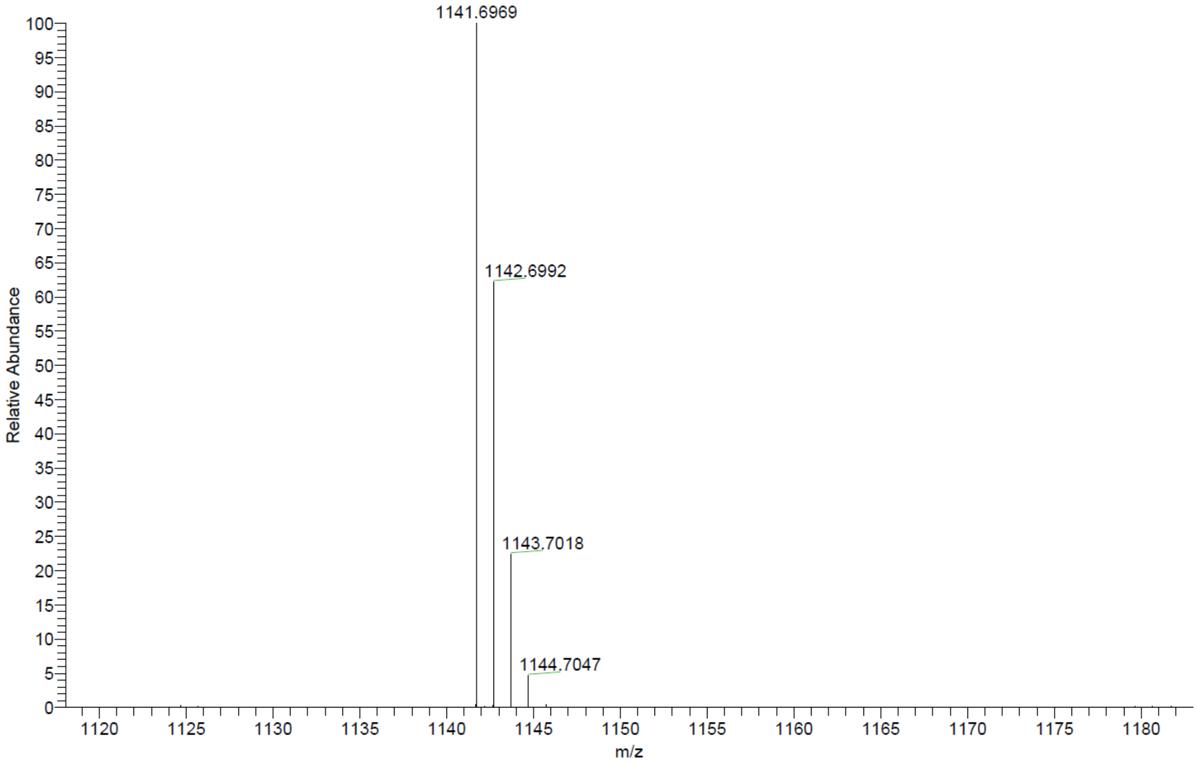

1141.6969
1142.6992
1143.7018
1144.7047
Relative Abundance
m/z

EB47 was obtained as a foamy white solid after preparative RP-HPLC (24 mg, 8 %).

Analytical RP-HPLC: t_R = 2.30 min (A/D 90:10 to 10:90 in 7.0 min, λ = 214 nm). **MS** (ESI$^+$): $C_{54}H_{91}N_{15}O_{13}$ calc./obs. 1186.7438/1186.7438 Da [M].

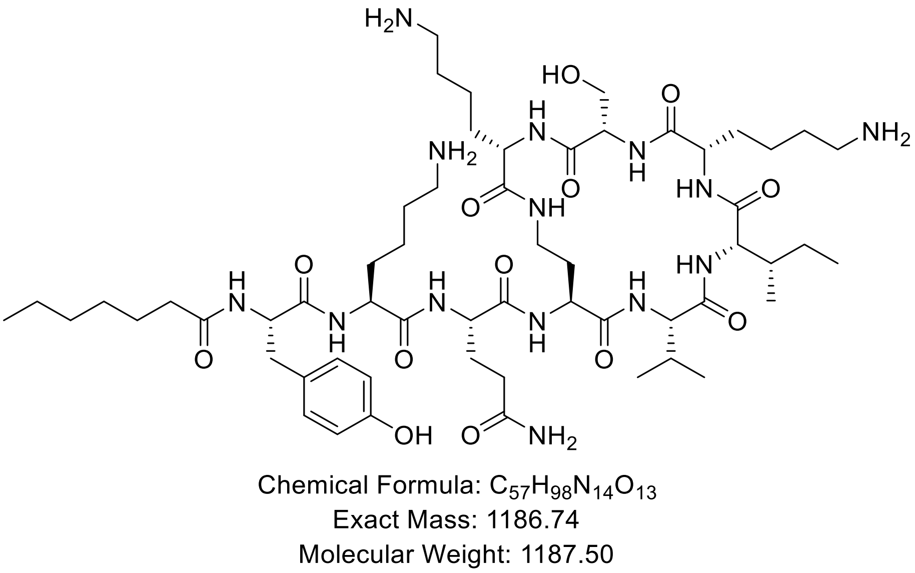

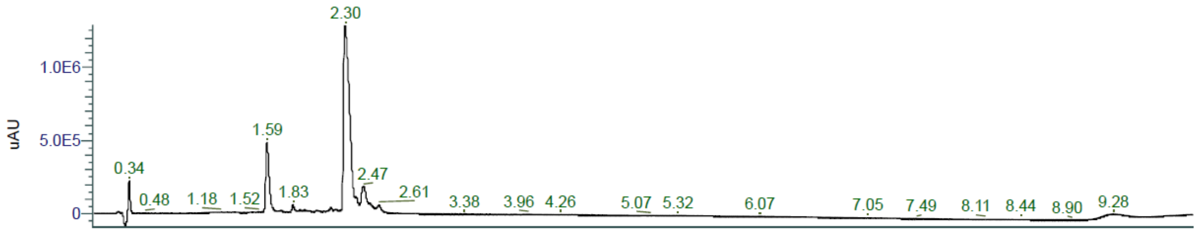

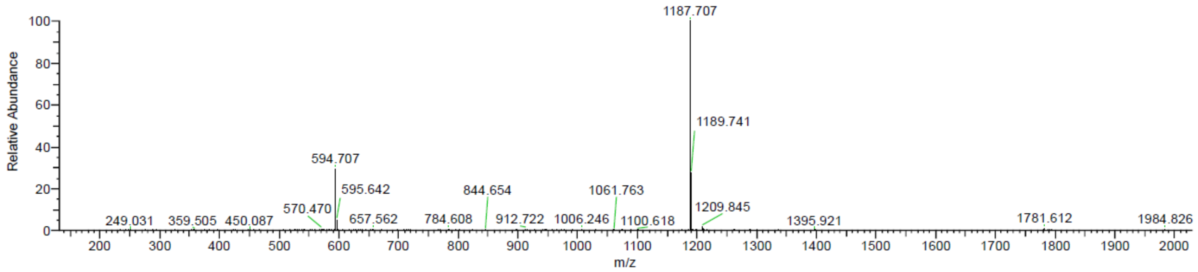

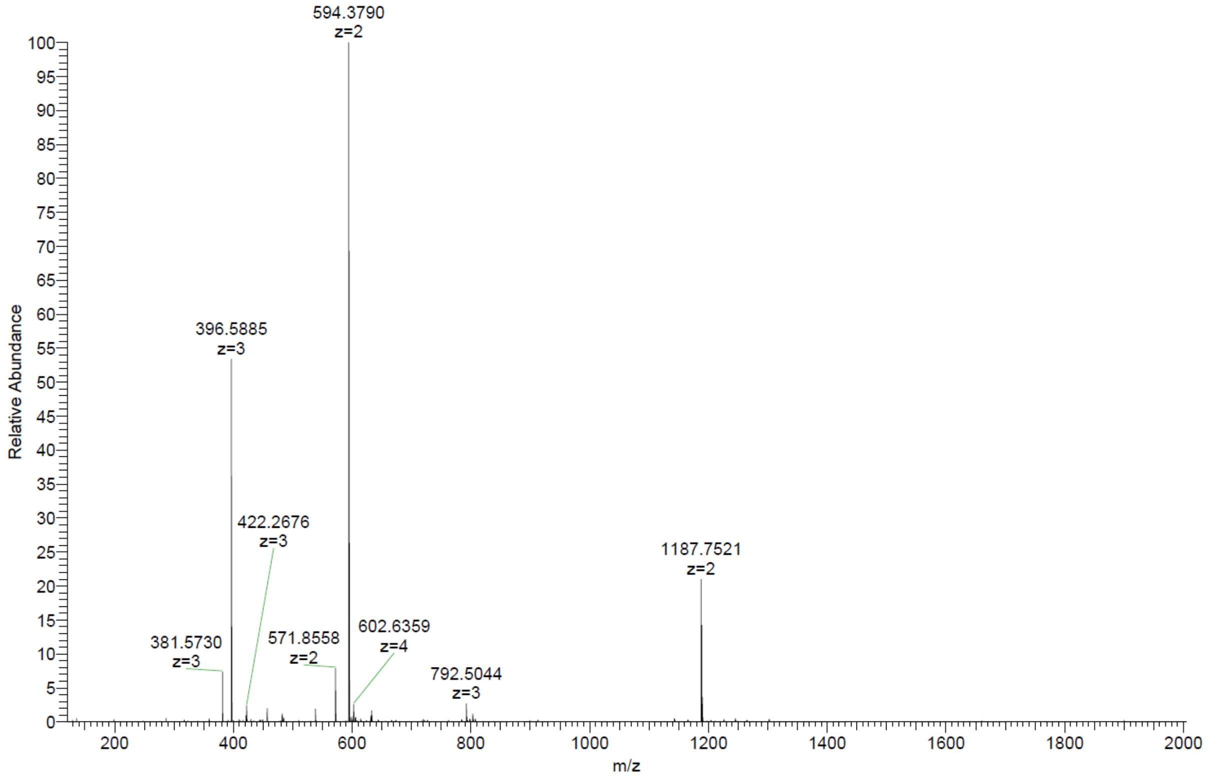
594.3790
z=2
396.5885
z=3
422.2676
z=3
381.5730
z=3
571.8558
z=2
602.6359
z=4
792.5044
z=3
1187.7521
z=2
Relative Abundance
m/z

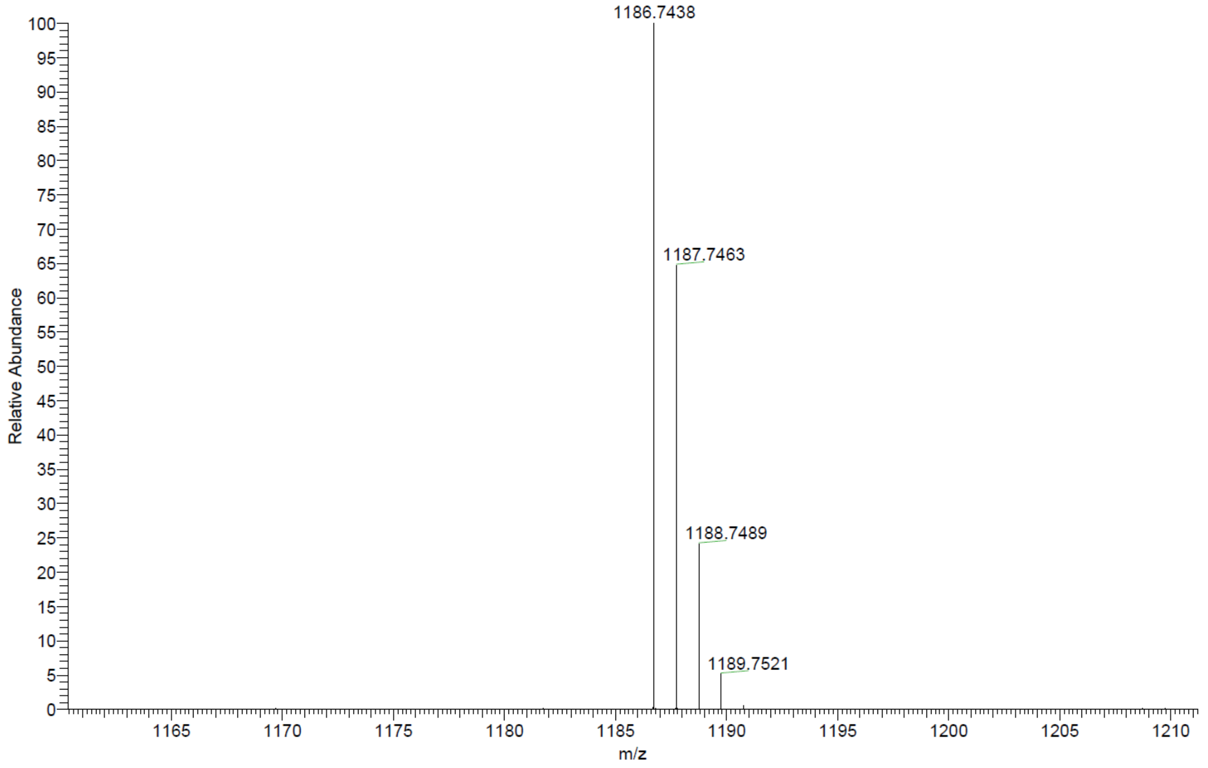
1186.7438
1187.7463
1188.7489
1189.7521
Relative Abundance
m/z

EB48 was obtained as a foamy white solid after preparative RP-HPLC (24 mg, 7 %).

Analytical RP-HPLC: t_R = 1.49 min (A/D 90:10 to 10:90 in 7.0 min, λ = 214 nm). **MS** (ESI$^+$): $C_{55}H_{97}N_{15}O_{11}$ calc./obs. 1143.7492/1143.7489 Da [M].

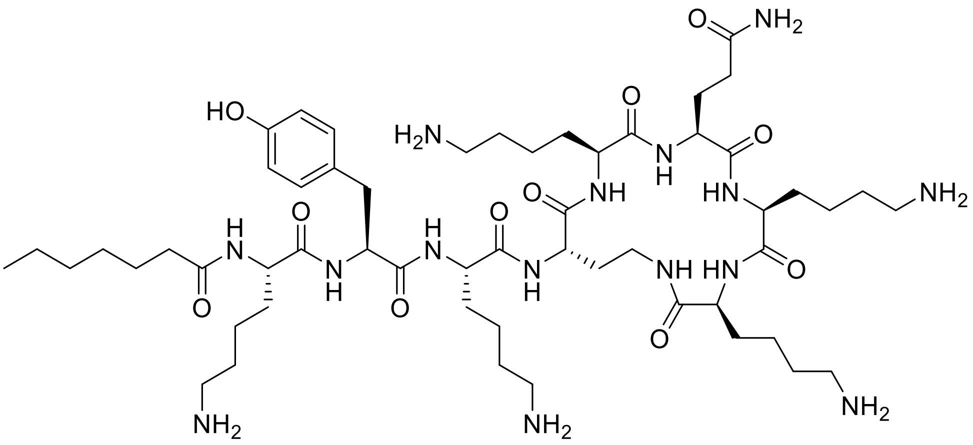

Chemical Formula: $C_{55}H_{97}N_{15}O_{11}$
Exact Mass: 1143.7492
Molecular Weight: 1144.4750

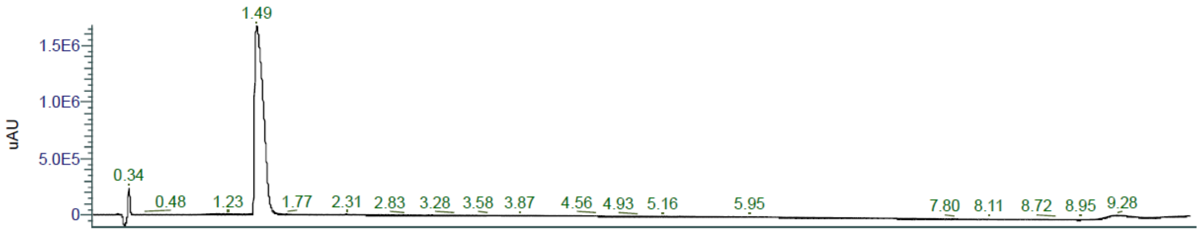

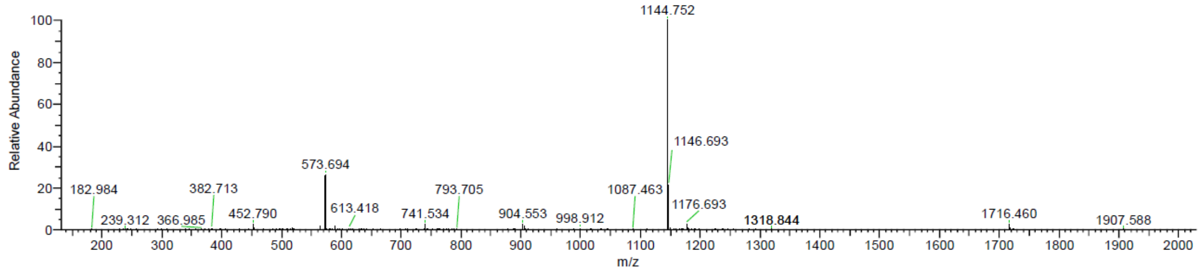

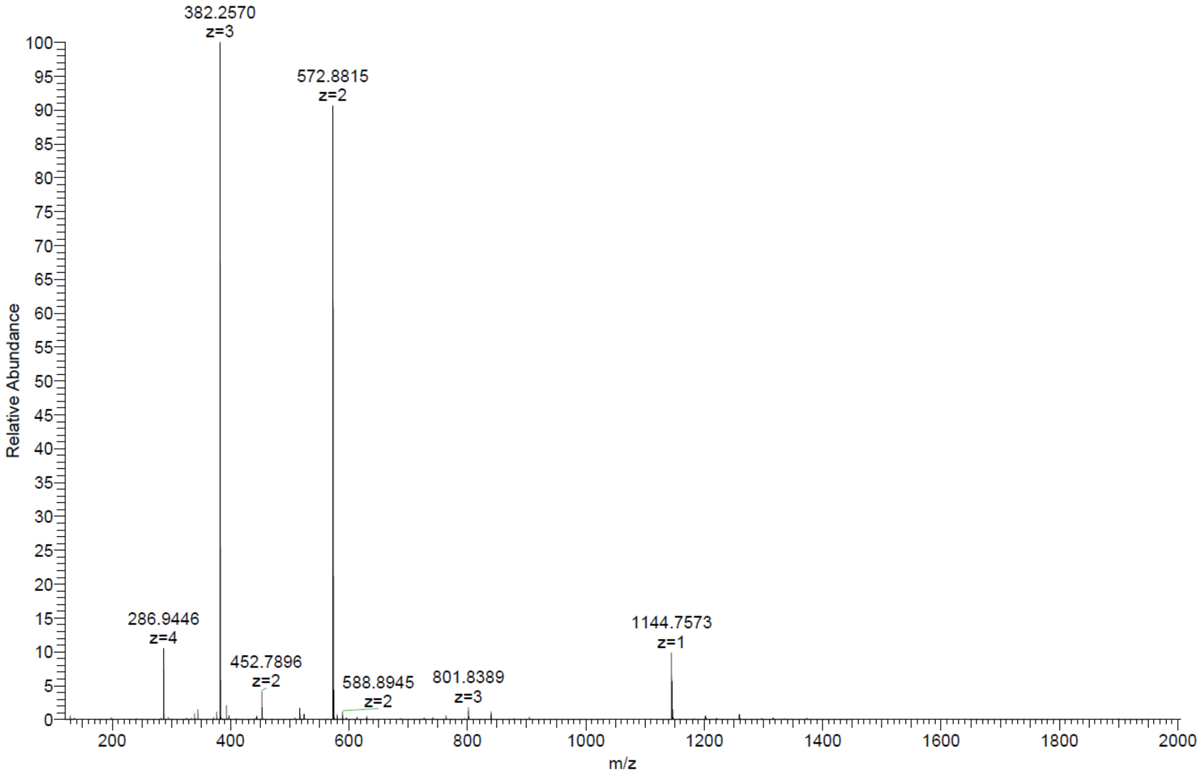
382.2570
z=3
572.8815
z=2
286.9446
z=4
452.7896
z=2
588.8945
z=2
801.8389
z=3
1144.7573
z=1
Relative Abundance
m/z

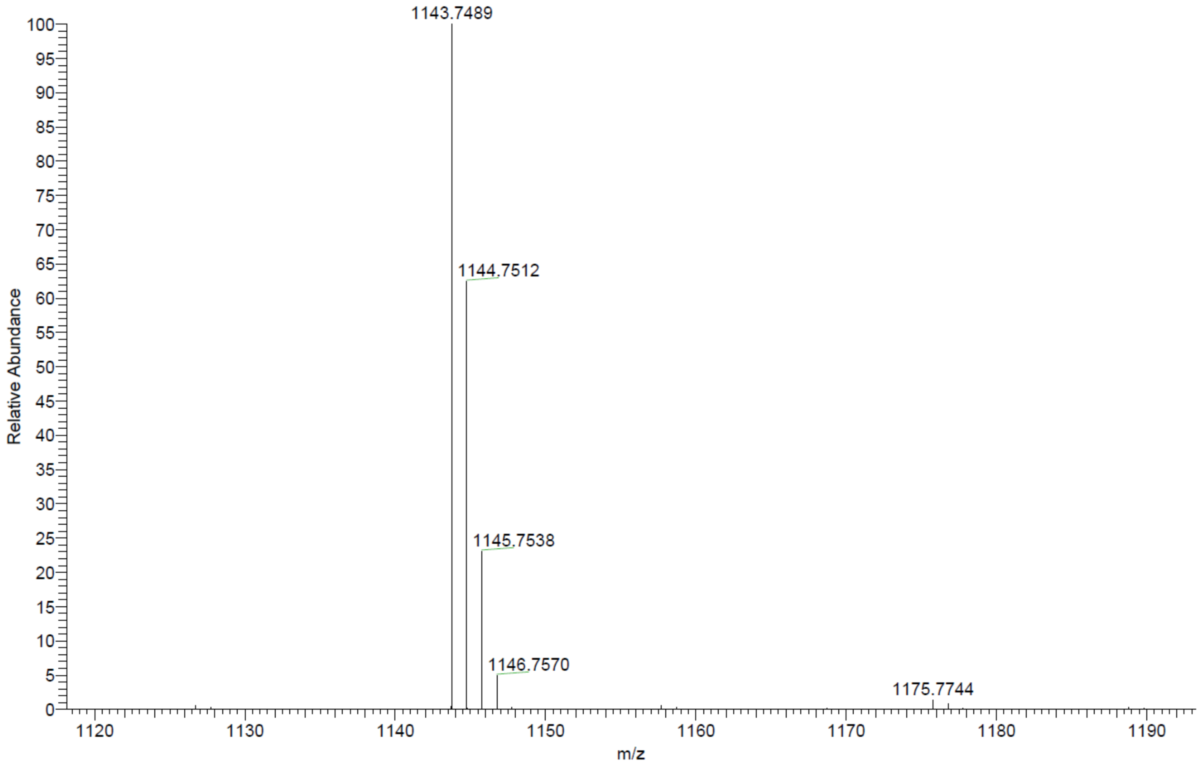
1143.7489
1144.7512
1145.7538
1146.7570
1175.7744
Relative Abundance
m/z

EB49 was obtained as a foamy white solid after preparative RP-HPLC (35 mg, 13 %).

Analytical RP-HPLC: t_R = 2.18 min (A/D 90:10 to 10:90 in 7.0 min, λ = 214 nm). **MS** (ESI$^+$): $C_{54}H_{91}N_{15}O_{13}$ calc./obs. 1157.6921/1157.6920 Da [M].

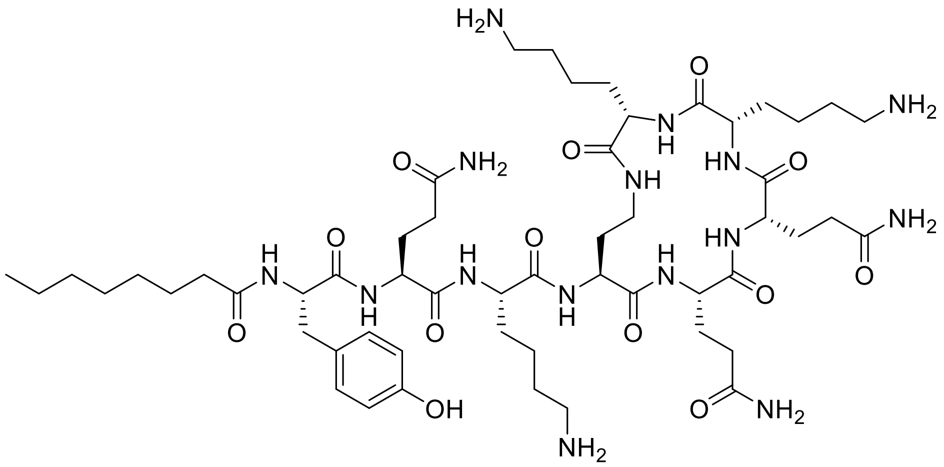

Chemical Formula: $C_{54}H_{91}N_{15}O_{13}$
Exact Mass: 1157.6921
Molecular Weight: 1158.4140

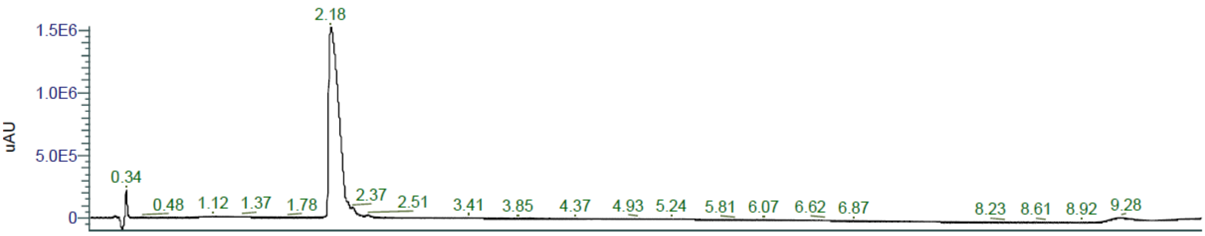

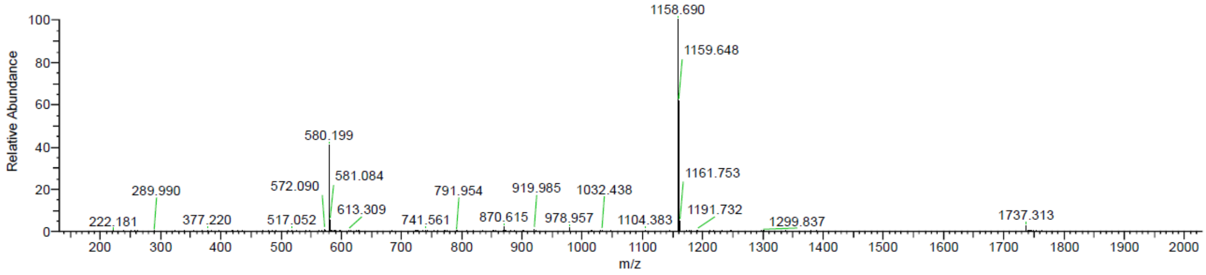

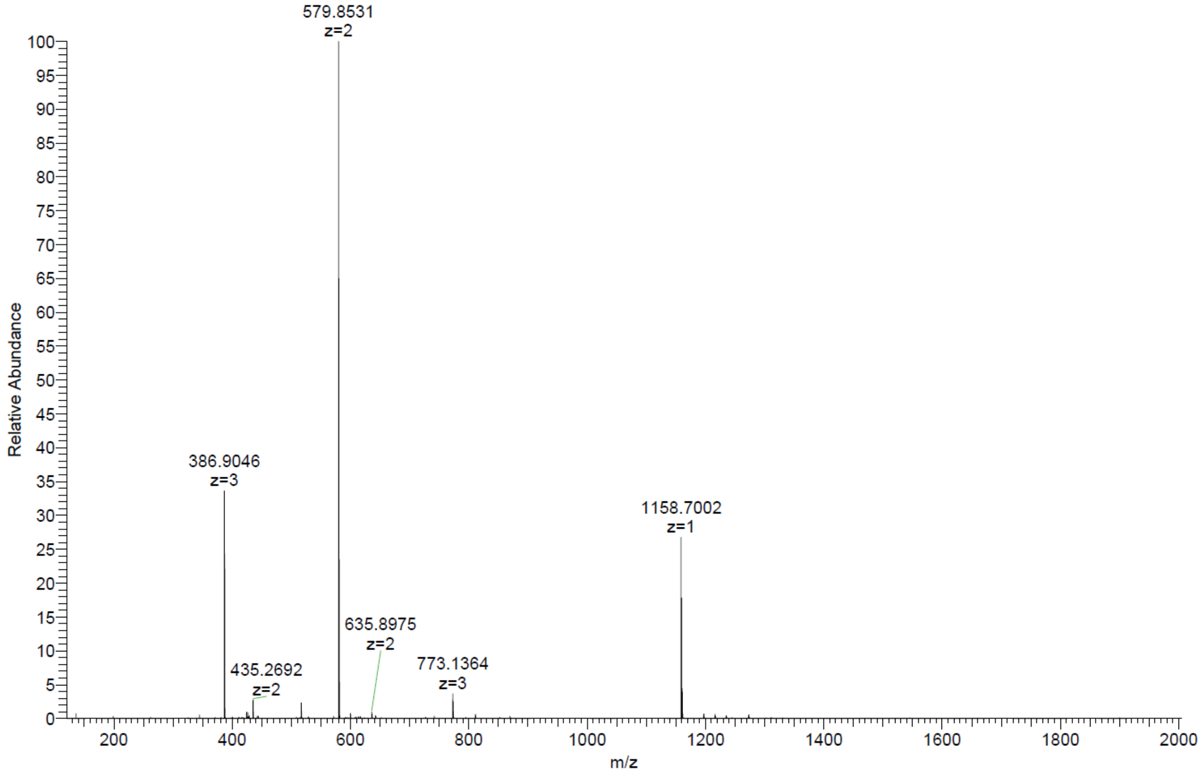
579.8531
z=2
386.9046
z=3
1158.7002
z=1
635.8975
z=2
435.2692
z=2
773.1364
z=3
Relative Abundance
m/z

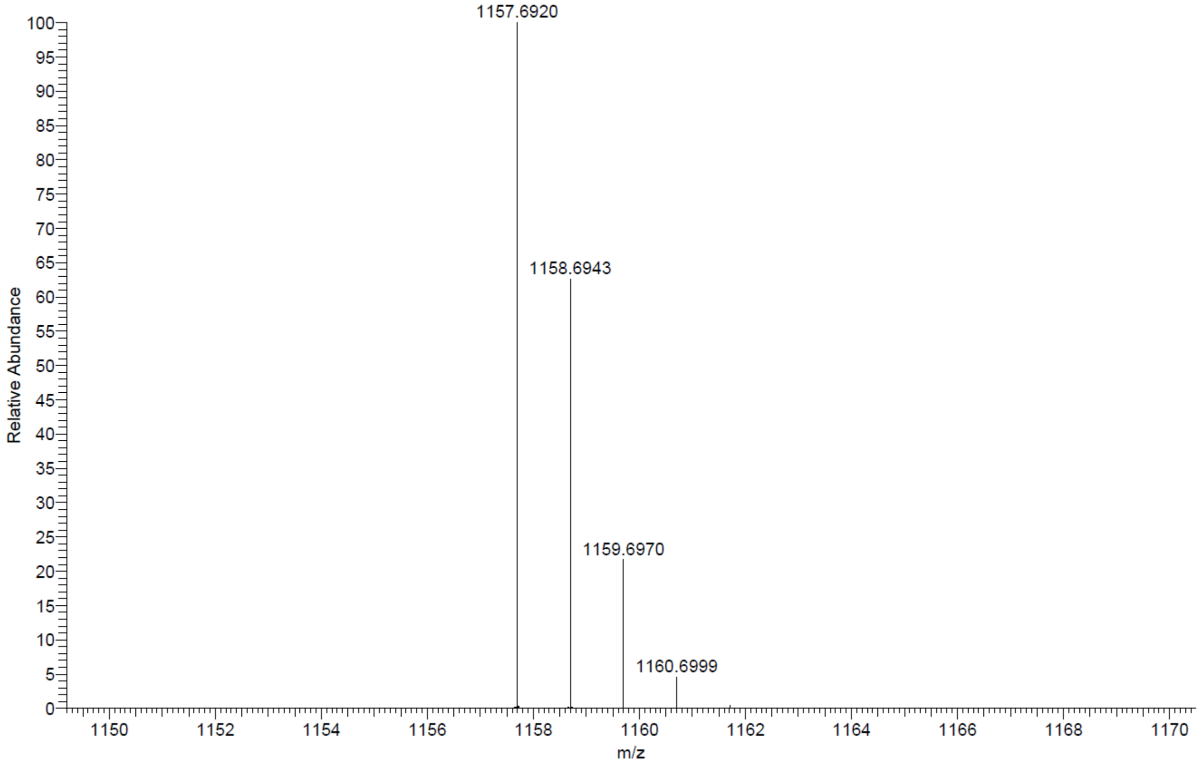
1157.6920
1158.6943
1159.6970
1160.6999
Relative Abundance
m/z

EB50 was obtained as a foamy white solid after preparative RP-HPLC (22 mg, 7 %).

Analytical RP-HPLC: t_R = 1.27 min (A/D 90:10 to 10:90 in 7.0 min, λ = 214 nm). **MS** (ESI$^+$): $C_{52}H_{89}N_{15}O_{12}$ calc./obs. 1115.6815/1115.6818 Da [M].

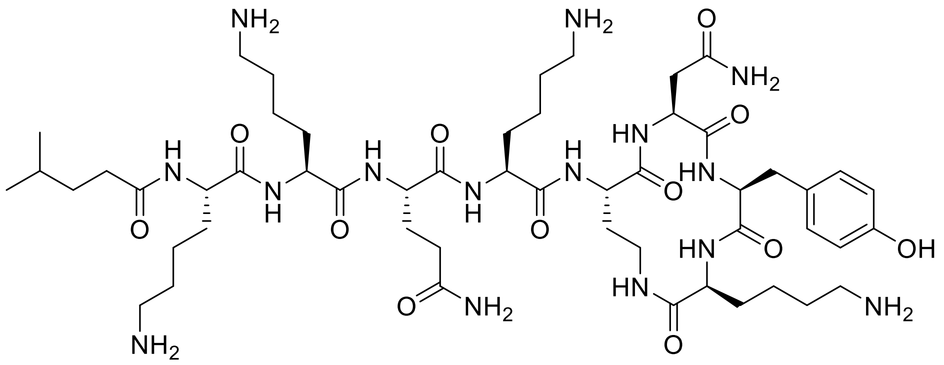

Chemical Formula: $C_{52}H_{89}N_{15}O_{12}$
Exact Mass: 1115.6815
Molecular Weight: 1116.3770

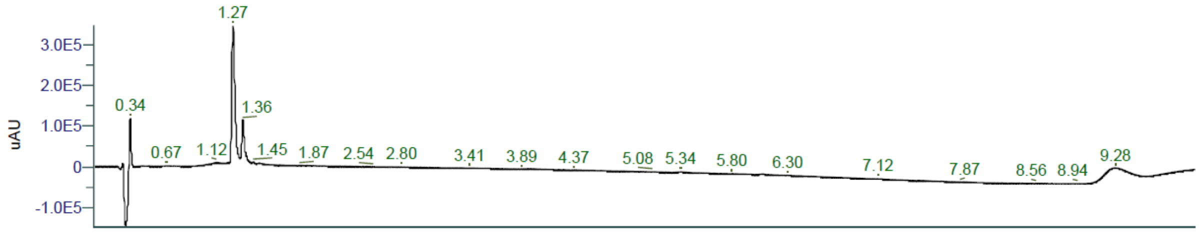

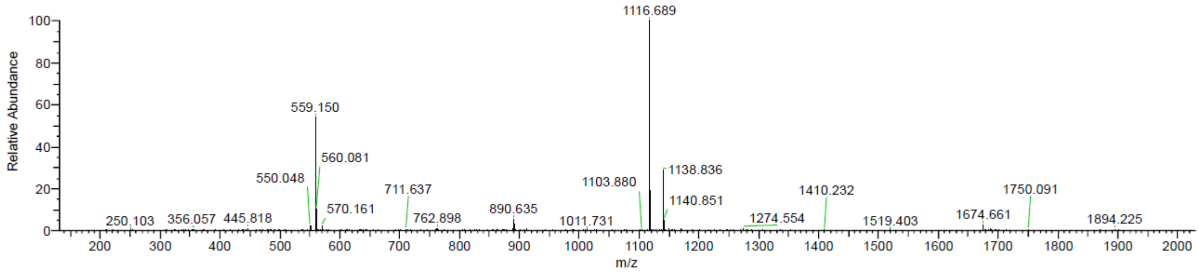

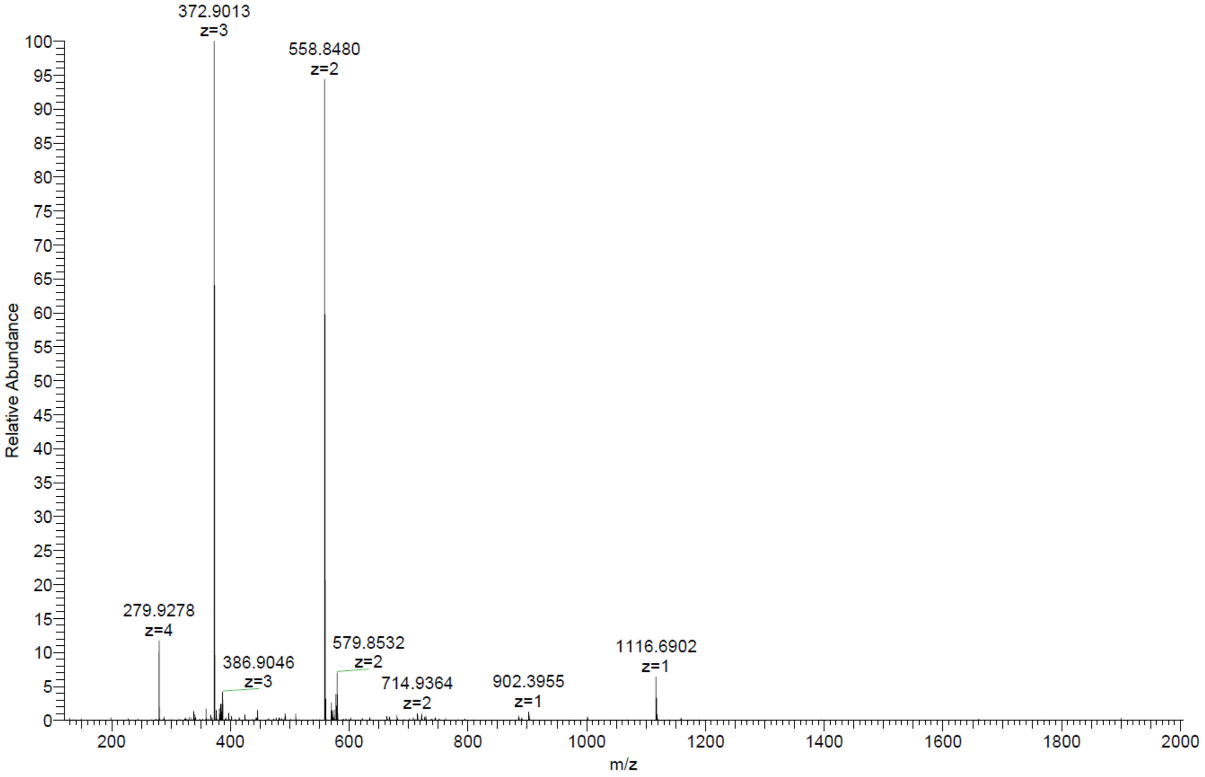
372.9013
z=3
558.8480
z=2
279.9278
z=4
386.9046
z=3
579.8532
z=2
714.9364
z=2
902.3955
z=1
1116.6902
z=1
Relative Abundance
m/z

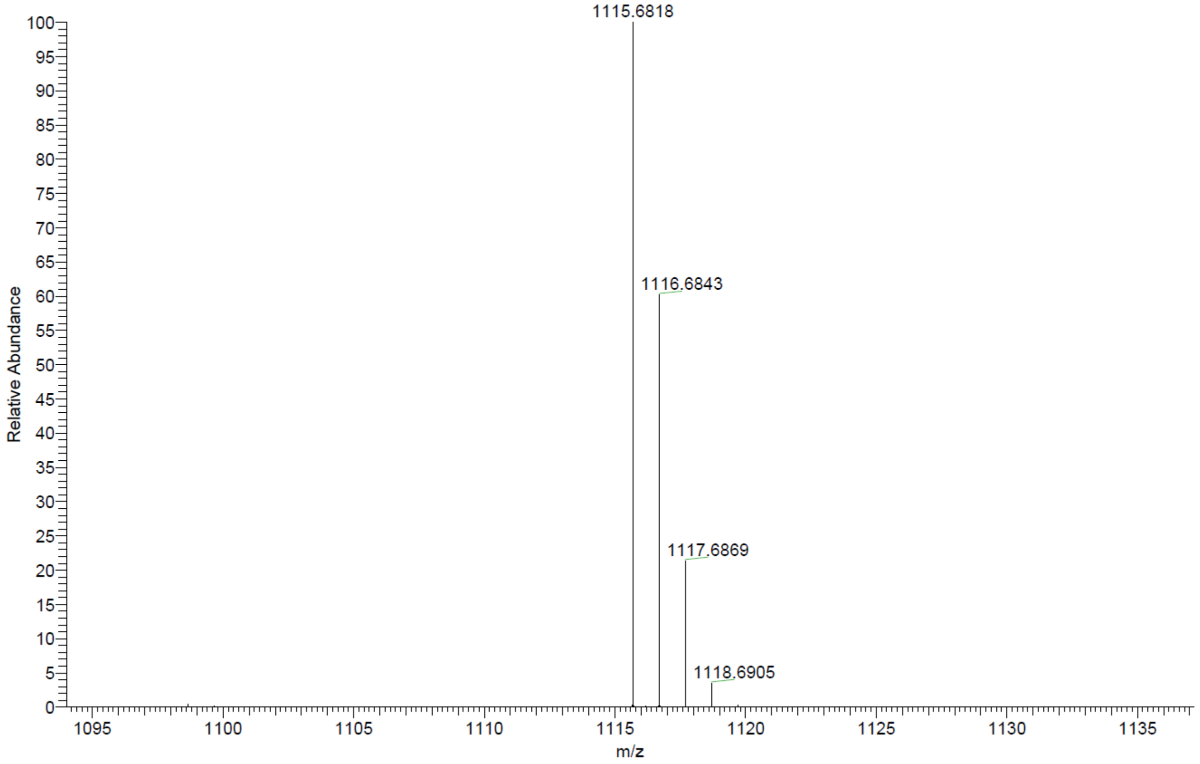
1115.6818
1116.6843
1117.6869
1118.6905
Relative Abundance
m/z

EB51 was obtained as a foamy white solid after preparative RP-HPLC (17 mg, 8 %). **Analytical RP-HPLC**: t_R = 2.16 min (A/D 100:0 to 0:100 in 7.0 min, λ = 214 nm). **MS** (ESI$^+$): $C_{54}H_{94}N_{16}O_{12}$ calc./obs. 1158.7237/1158.7227 Da [M].

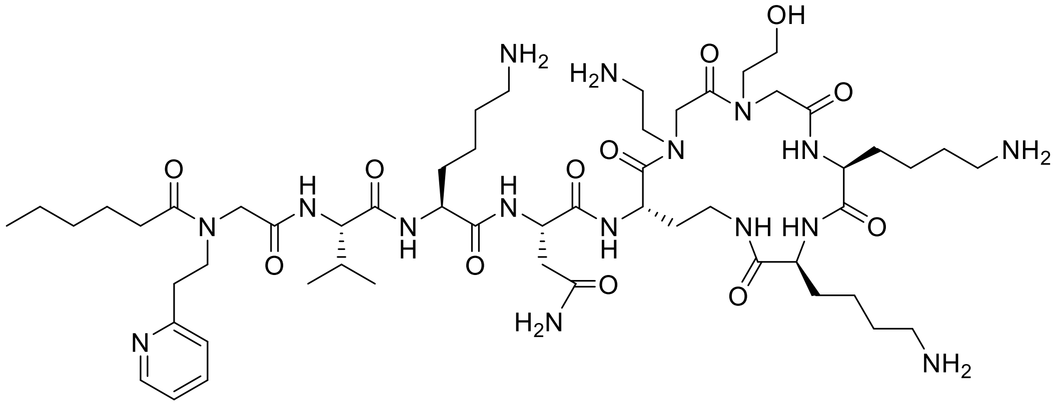

Chemical Formula: $C_{54}H_{94}N_{16}O_{12}$
Exact Mass: 1158.7237
Molecular Weight: 1159.4460

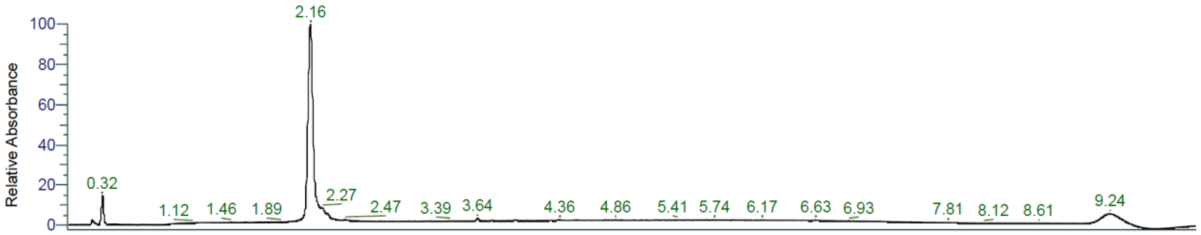

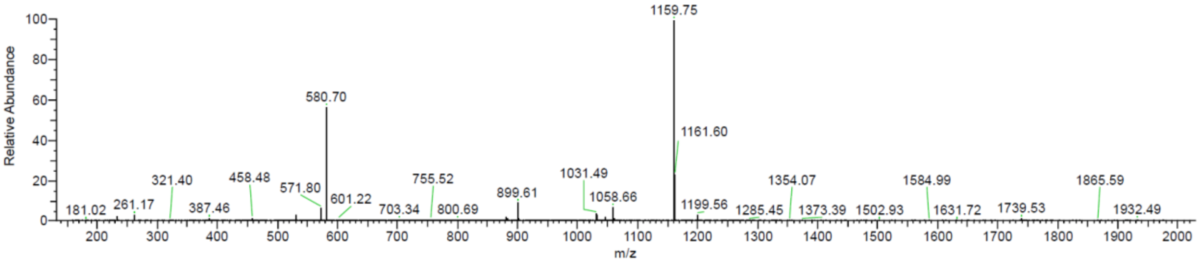

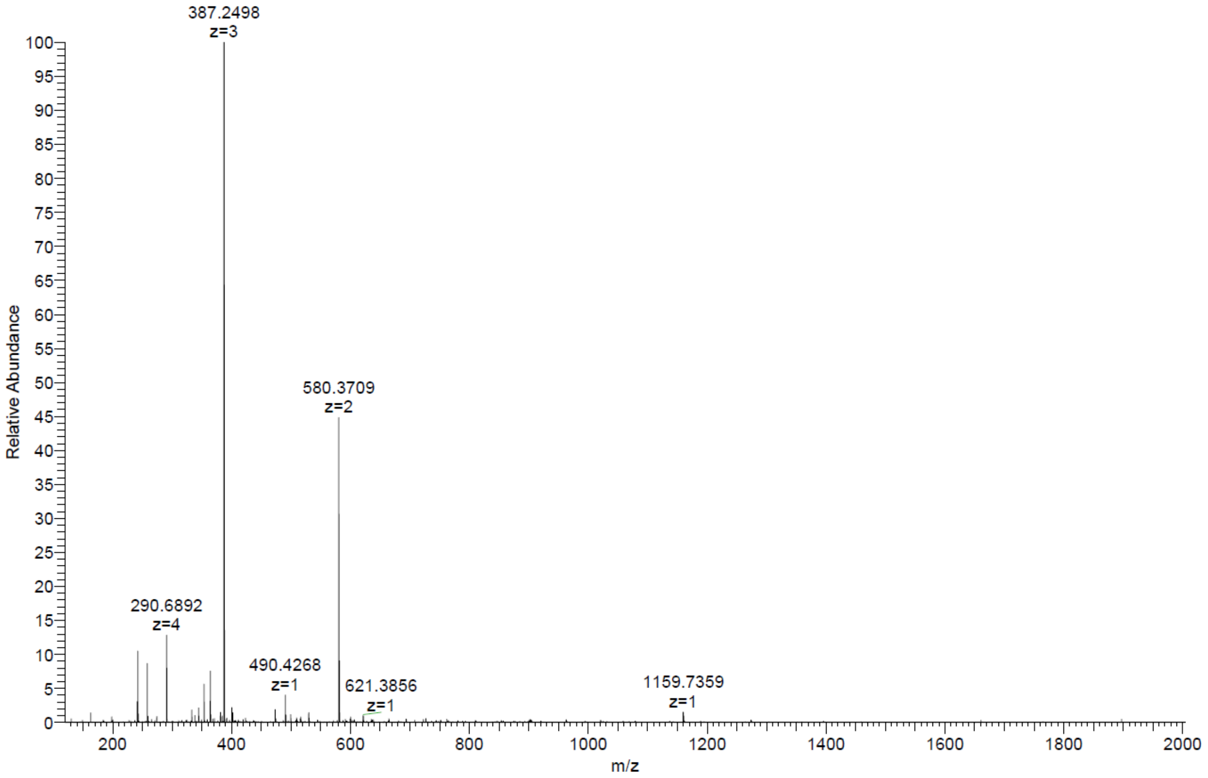
387.2498
z=3
580.3709
z=2
290.6892
z=4
490.4268
z=1
621.3856
z=1
1159.7359
z=1
Relative Abundance
m/z

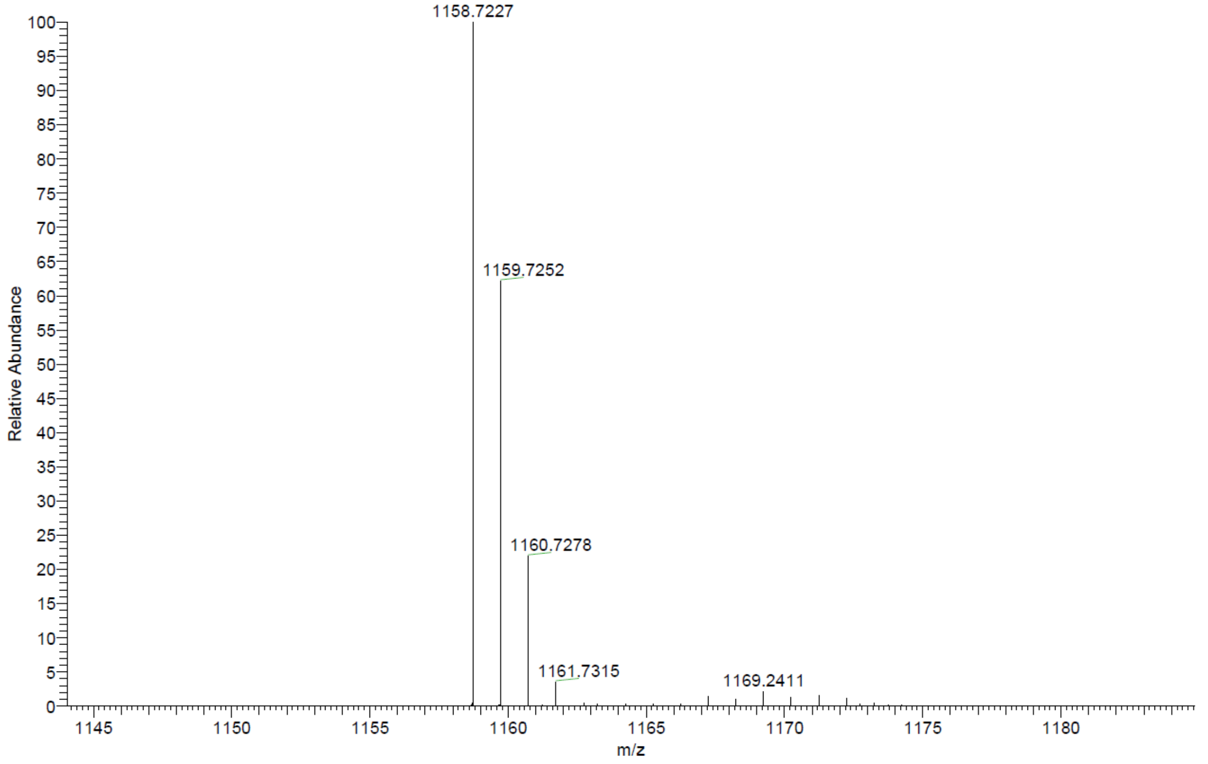
1158.7227
1159.7252
1160.7278
1161.7315
1169.2411
Relative Abundance
m/z

EB52 was obtained as a foamy white solid after preparative RP-HPLC (12 mg, 6 %).

Analytical RP-HPLC: t_R = 1.86 min (A/D 90:10 to 10:90 in 7.0 min, λ = 214 nm). **MS** (ESI$^+$): $C_{55}H_{93}N_{17}O_{12}$ calc./obs. 1183.7190/1183.7167 Da [M].

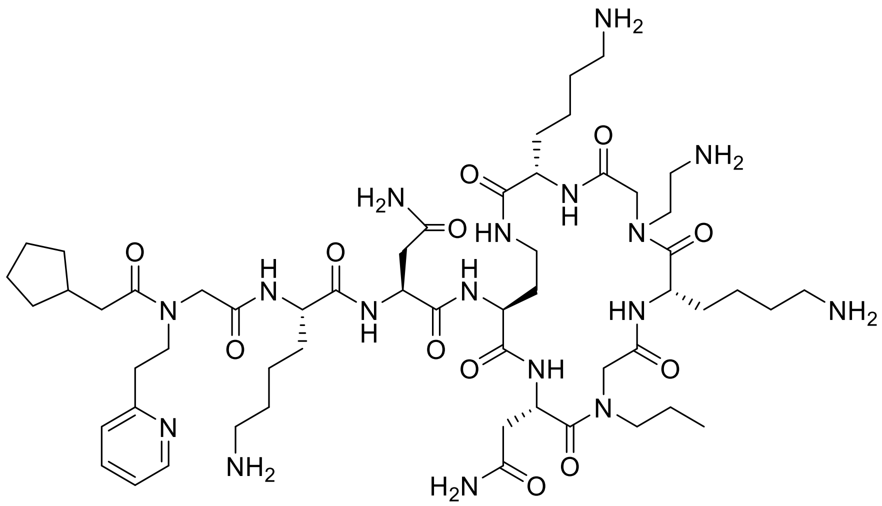

Chemical Formula: $C_{55}H_{93}N_{17}O_{12}$
Exact Mass: 1183.7190
Molecular Weight: 1184.4560

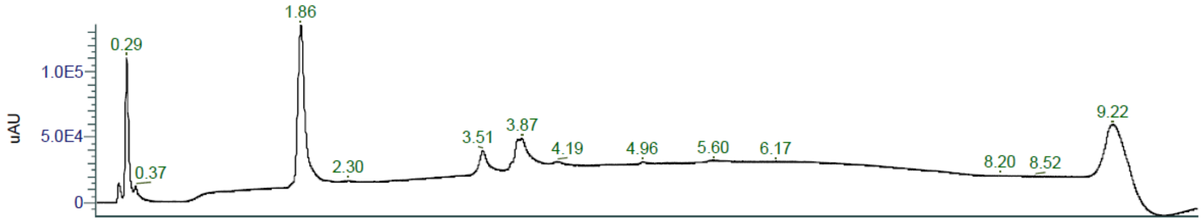

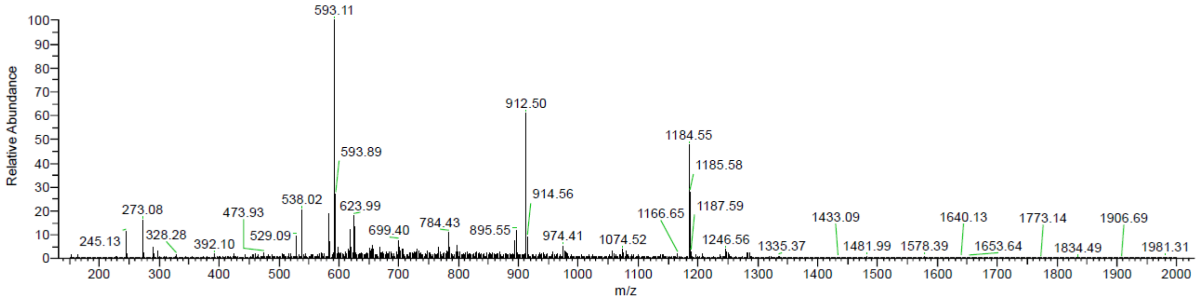

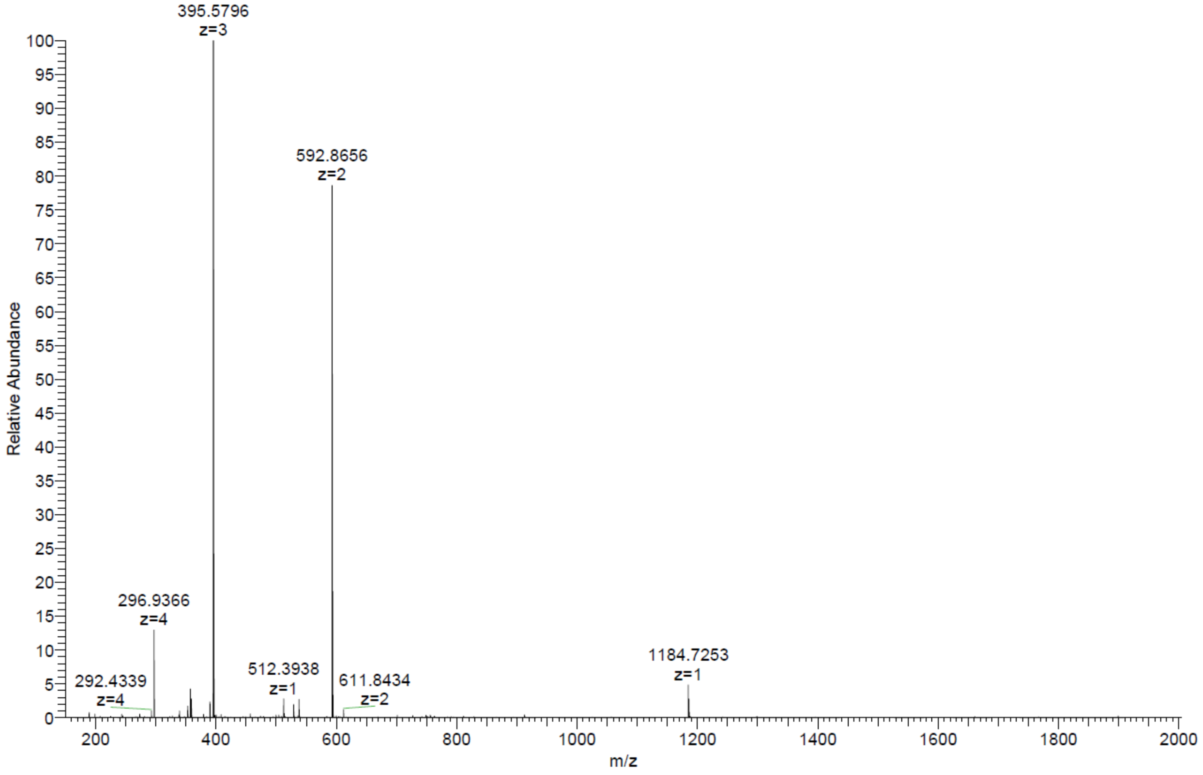
395.5796
z=3
592.8656
z=2
296.9366
z=4
292.4339
z=4
512.3938
z=1
611.8434
z=2
1184.7253
z=1
Relative Abundance
m/z

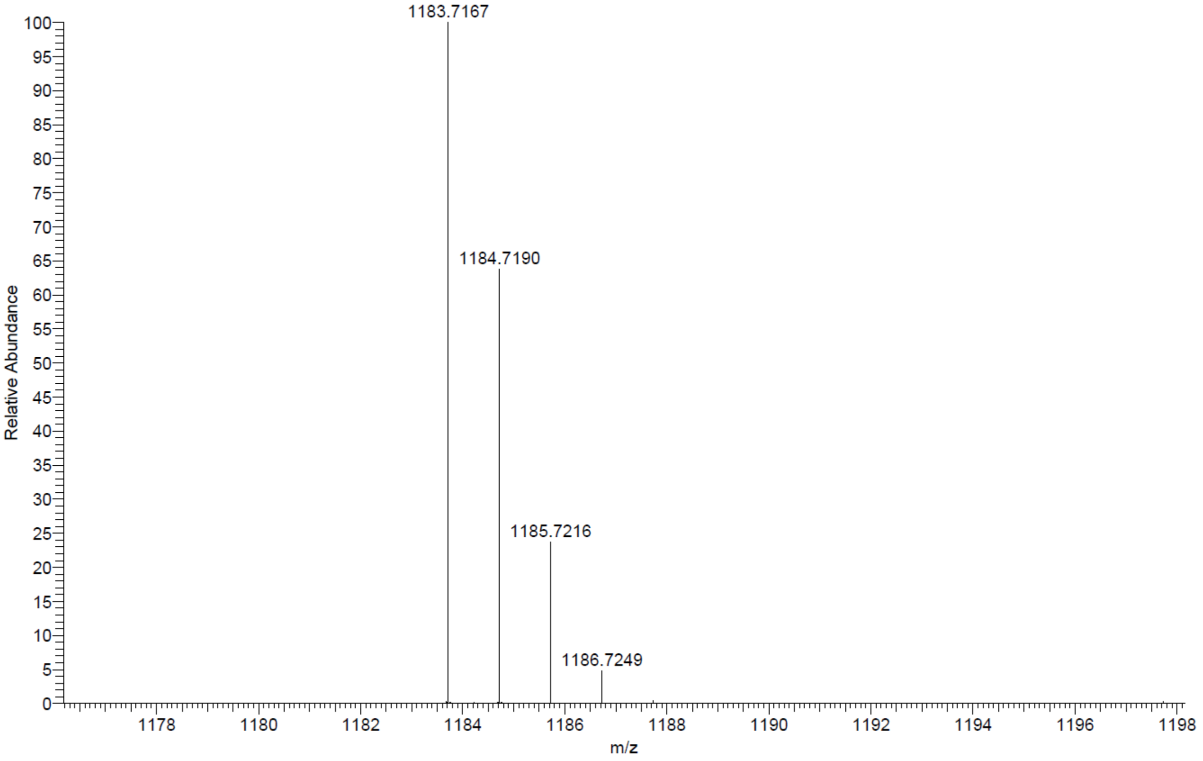
1183.7167
1184.7190
1185.7216
1186.7249
Relative Abundance
m/z

PMB2lin was obtained as a foamy white solid after preparative RP-HPLC (30 mg, 31 %).

Analytical RP-HPLC: t_R = 3.17 min (A/D 90:10 to 10:90 in 7.0 min, λ = 214 nm).

MS (ESI+): $C_{54}H_{96}N_{16}O_{13}$ calc./obs. 1176.7343/1176.7343 Da [M].

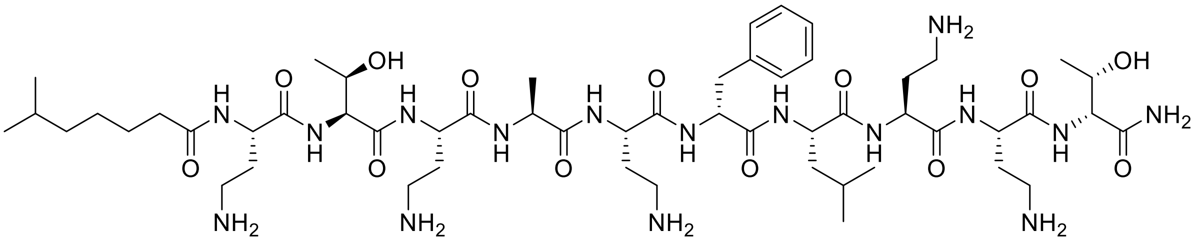

Chemical Formula: $C_{54}H_{96}N_{16}O_{13}$
Exact Mass: 1176.7343
Molecular Weight: 1177.4610

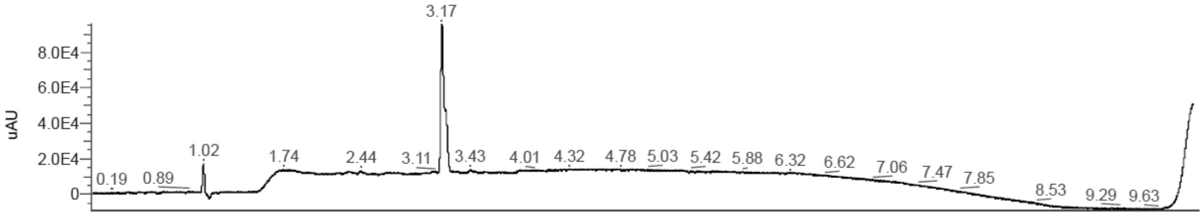

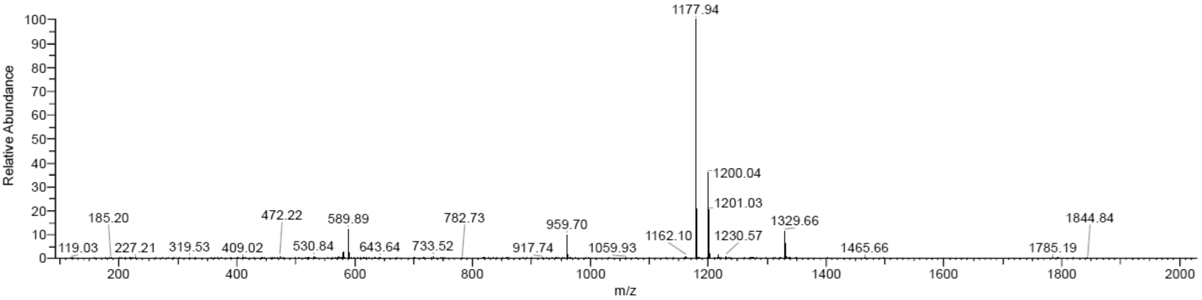

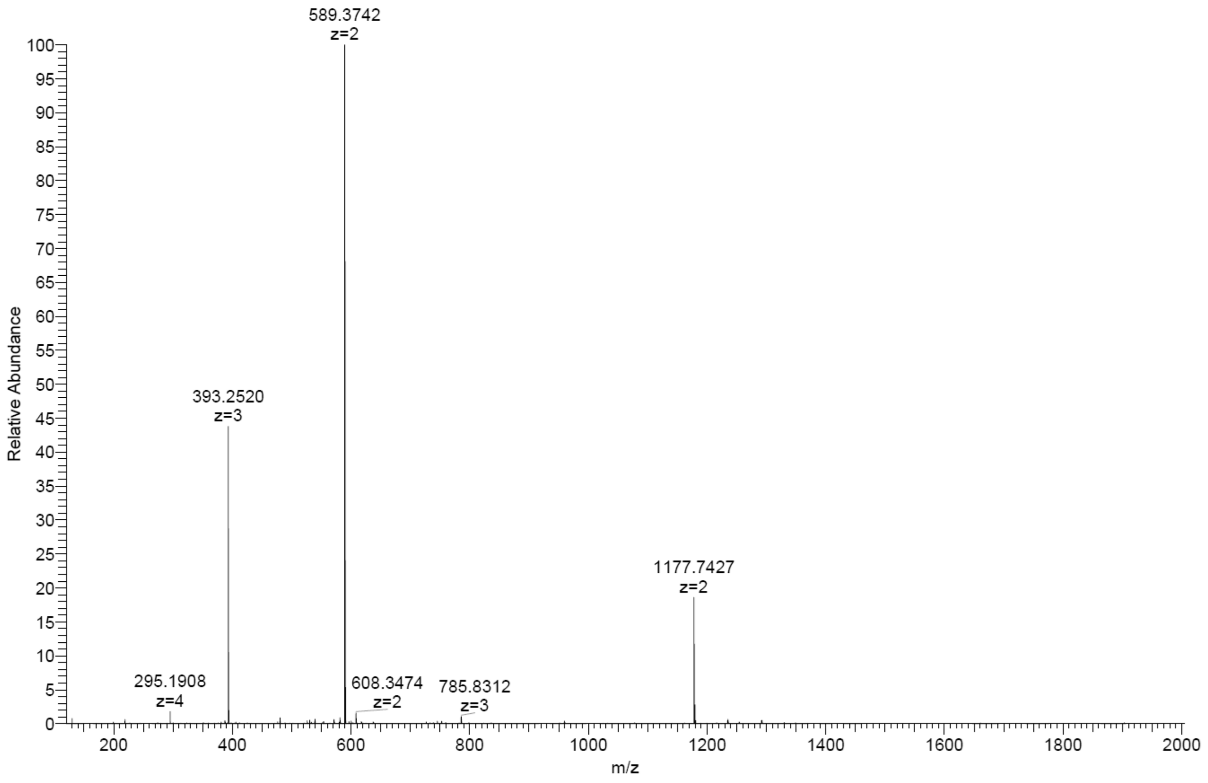
589.3742
z=2
393.2520
z=3
1177.7427
z=2
295.1908
z=4
608.3474
z=2
785.8312
z=3
Relative Abundance
m/z

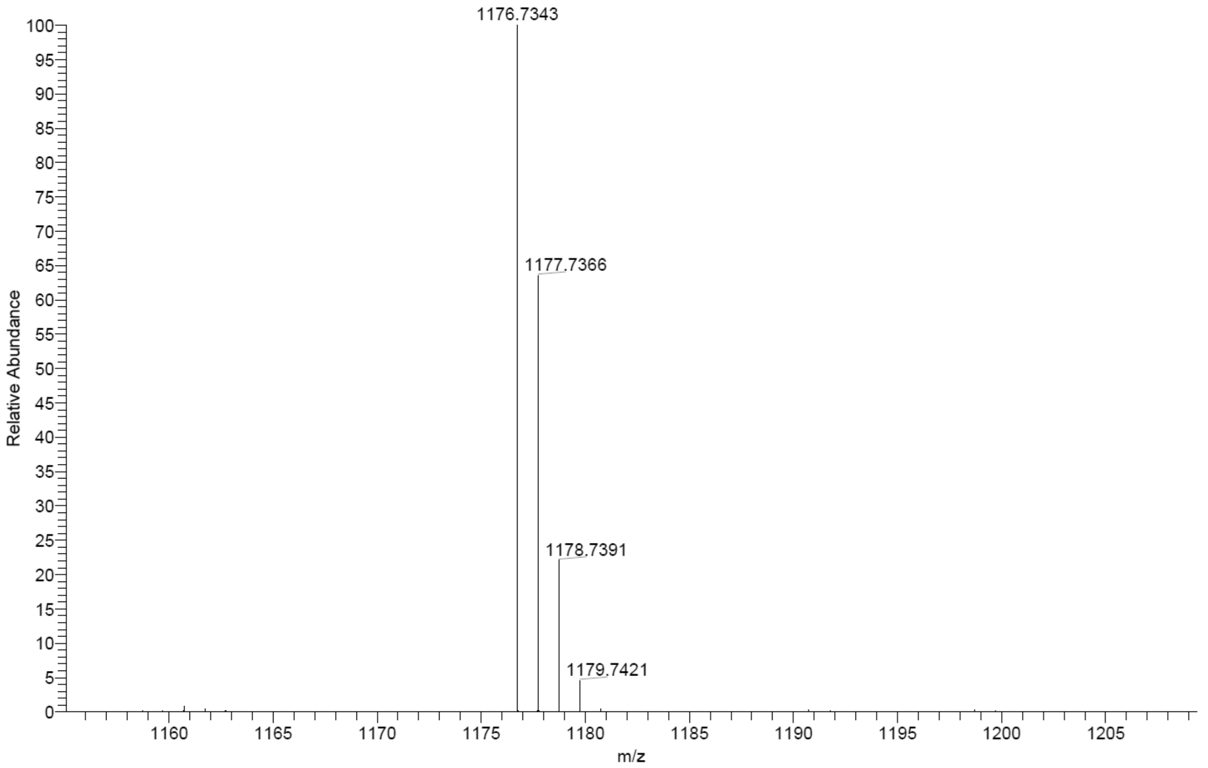
1176.7343
1177.7366
1178.7391
1179.7421
Relative Abundance
m/z

EB12lin was obtained as a foamy white solid after preparative RP-HPLC (22 mg, 23 %).

Analytical RP-HPLC: t_R = 3.70 min (A/D 90:10 to 10:90 in 7.0 min, λ = 214 nm).

MS (ESI$^+$): $C_{59}H_{106}N_{16}O_{11}$ calc./obs. 1214.8227/1214.8222 Da [M].

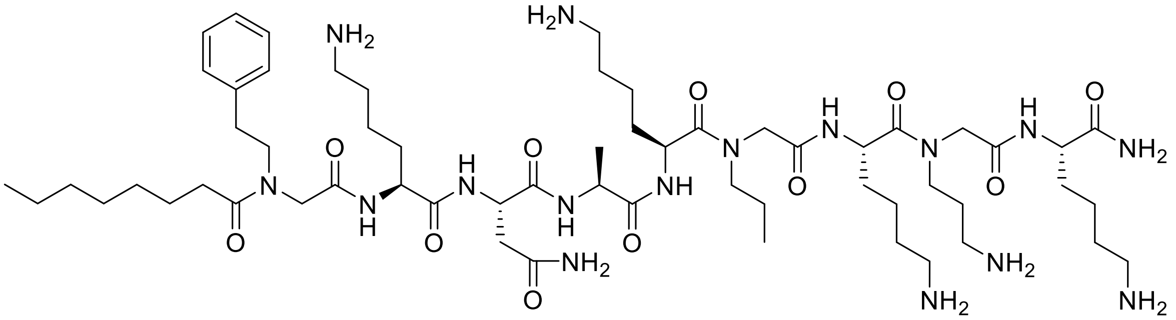

Chemical Formula: $C_{59}H_{106}N_{16}O_{11}$
Exact Mass: 1214.8227
Molecular Weight: 1215.5980

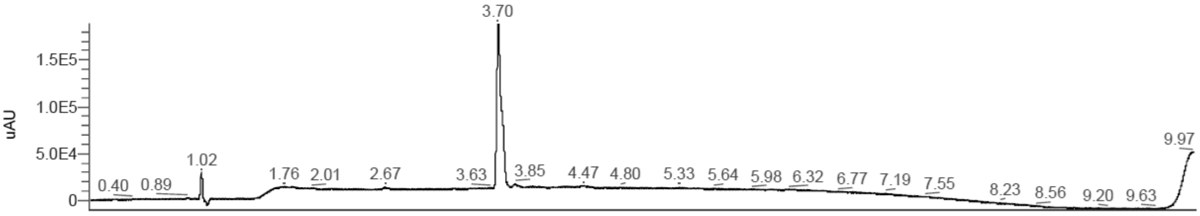

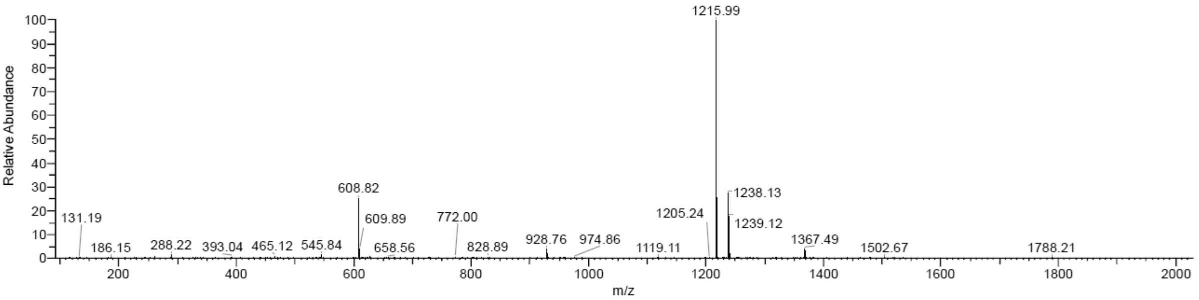

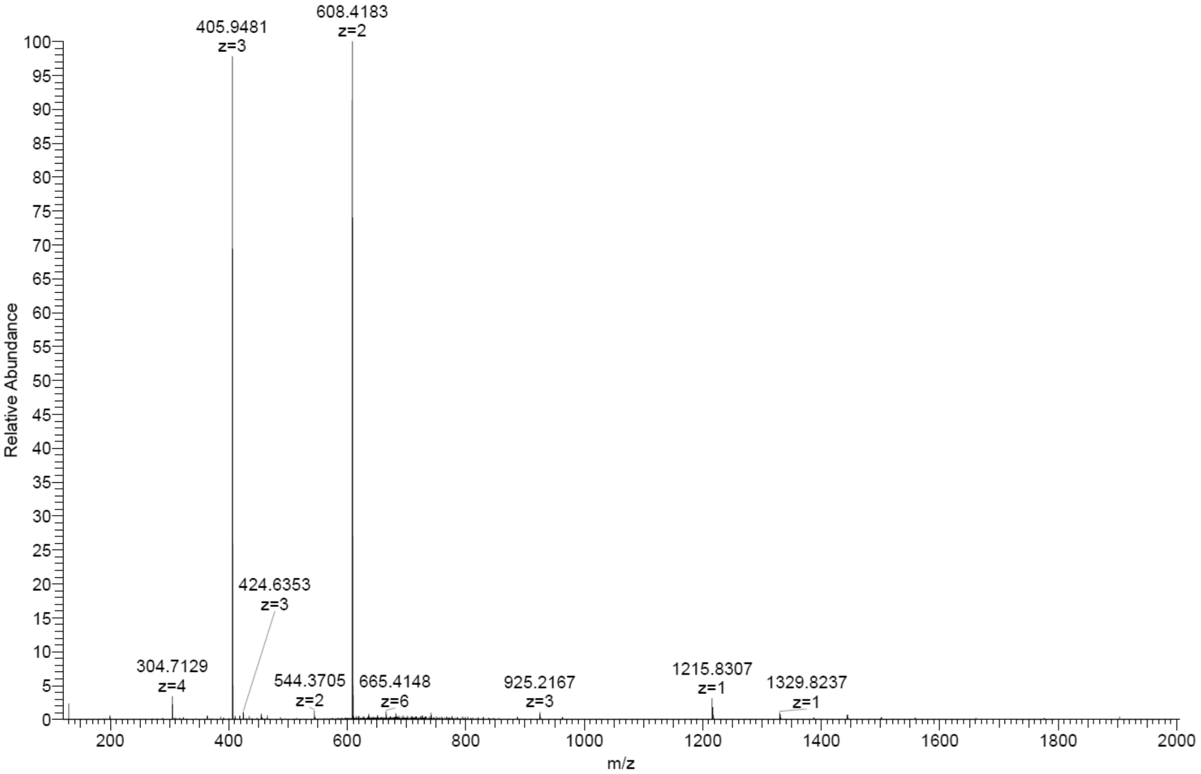
405.9481
z=3
608.4183
z=2
424.6353
z=3
304.7129
z=4
544.3705
z=2
665.4148
z=6
925.2167
z=3
1215.8307
z=1
1329.8237
z=1
Relative Abundance
m/z

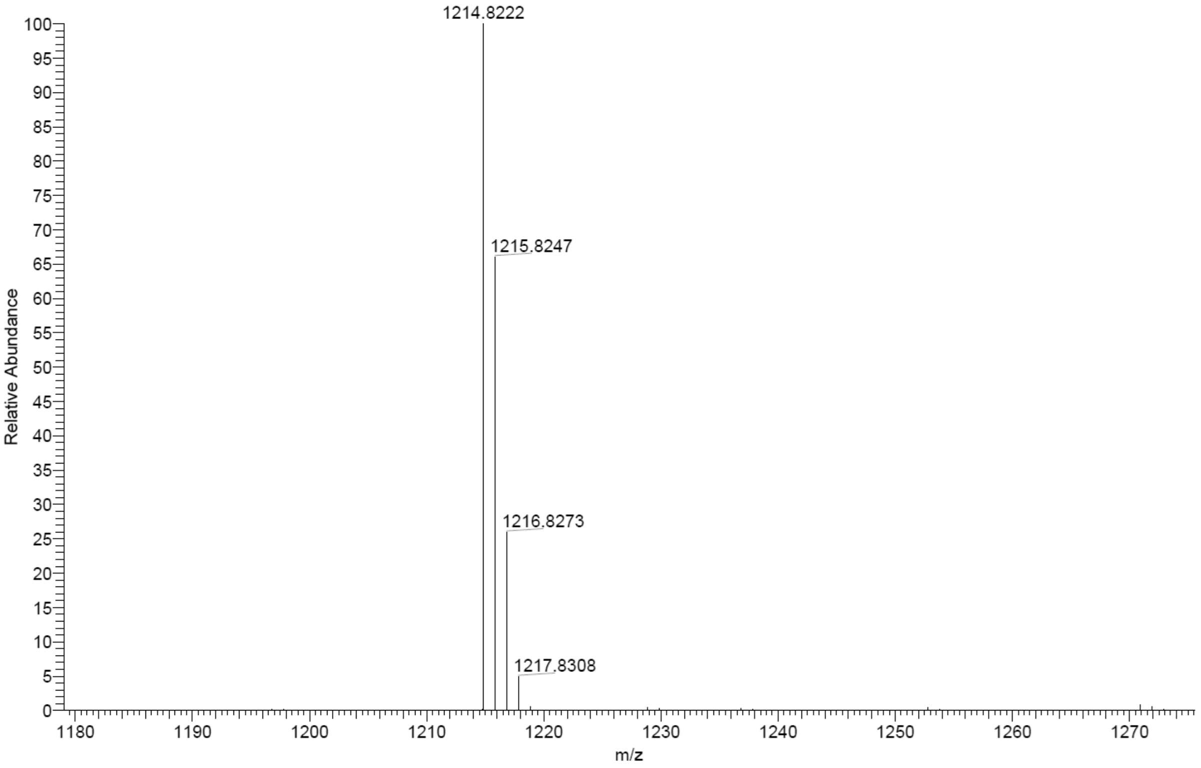
1214.8222
1215.8247
1216.8273
1217.8308
Relative Abundance
m/z

EB13lin was obtained as a foamy white solid after preparative RP-HPLC (10 mg, 10 %).

Analytical RP-HPLC: t_R = 3.68 min (A/D 90:10 to 10:90 in 7.0 min, λ = 214 nm).

MS (ESI$^+$): $C_{55}H_{99}N_{15}O_{10}$ calc./obs. 1129.7699/1129.7784 Da [M].

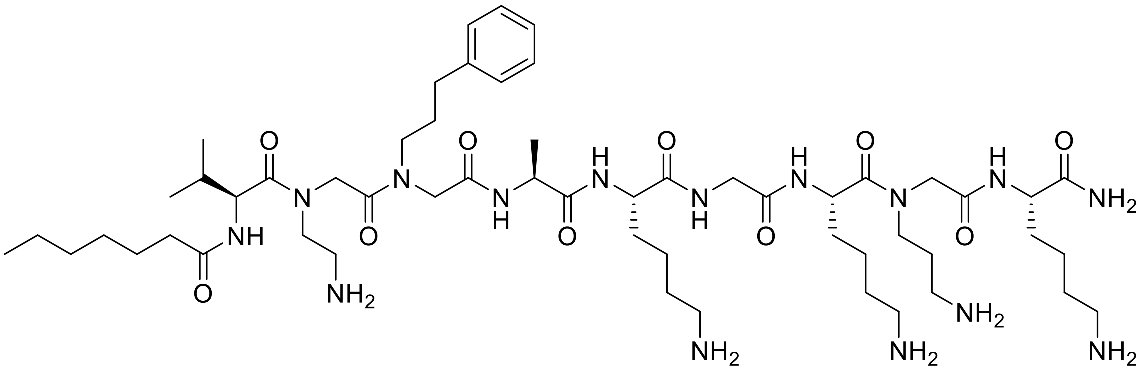

Chemical Formula: $C_{55}H_{99}N_{15}O_{10}$
Exact Mass: 1129.7699
Molecular Weight: 1130.4920

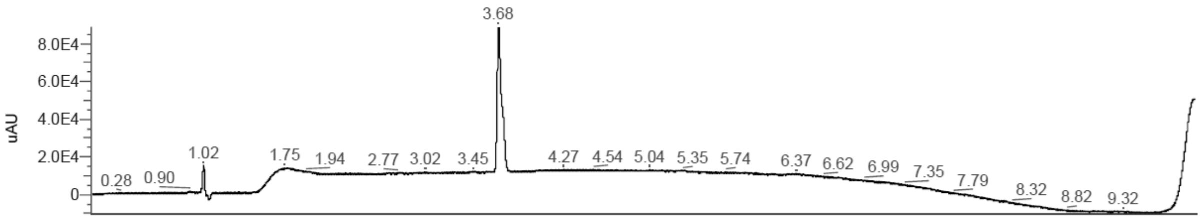

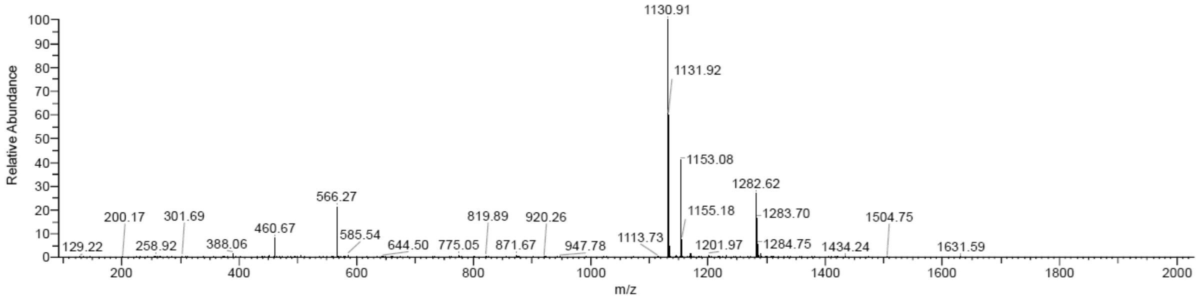

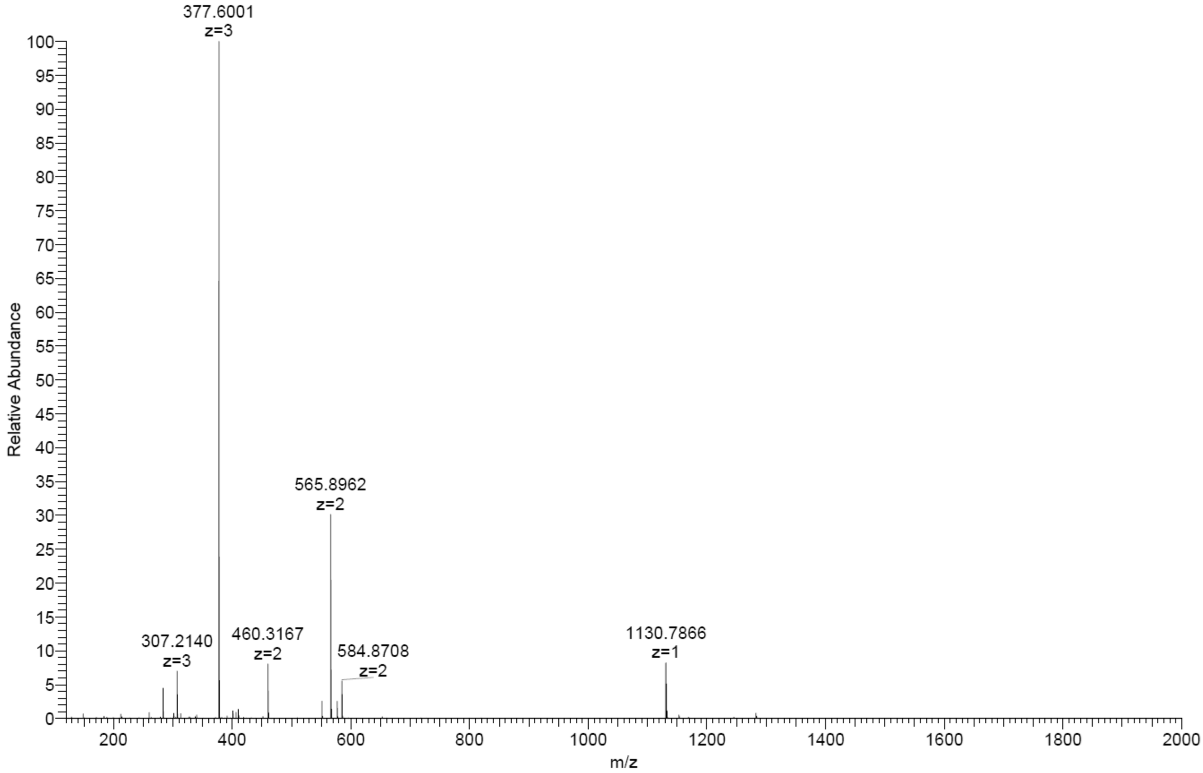
377.6001
z=3
565.8962
z=2
307.2140
z=3
460.3167
z=2
584.8708
z=2
1130.7866
z=1
Relative Abundance
m/z

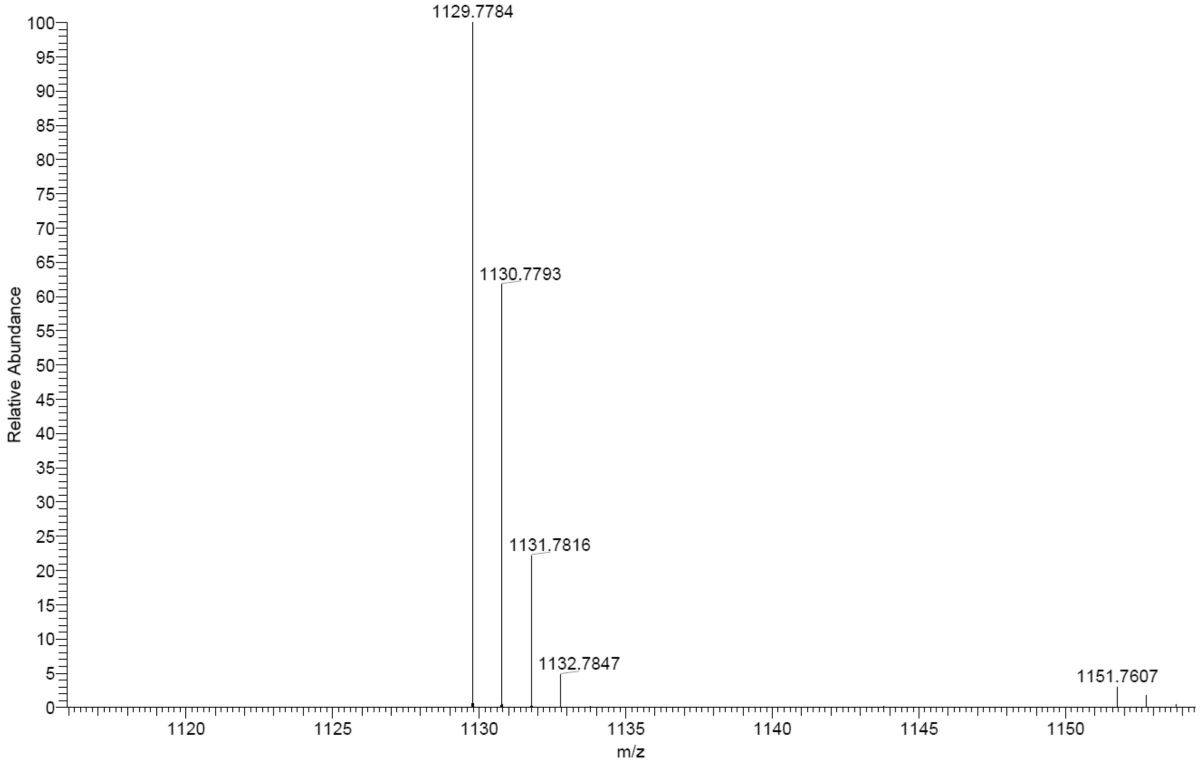
1129.7784
1130.7793
1131.7816
1132.7847
1151.7607
Relative Abundance
m/z

17. References

[1] A. Capecchi, A. Zhang, J.-L. Reymond, *J. Chem. Inf. Model.* **2020**, *60*, 121–132.
[2] M. Orsi, J.-L. Reymond, *Molecular Informatics* **2024**, e202400186.
[3] A. Capecchi, M. Awale, D. Probst, J. Reymond, *Mol. Inform.* **2019**, *38*, 1900016.
[4] M. Orsi, D. Probst, P. Schwaller, J.-L. Reymond, *Digit. Discov.* **2023**, *2*, 1289–1296.
[5] M. Gude, J. Ryf, P. D. White, *Lett. Pept. Sci.* **2002**, *9*, 203–206.
[6] I. Wiegand, K. Hilpert, R. E. W. Hancock, *Nat. Protoc.* **2008**, *3*, 163–175.
[7] M. V. Berridge, P. M. Herst, A. S. Tan, in *Biotechnology Annual Review*, Elsevier, **2005**, pp. 127–152.
[8] E. Bonvin, H. Personne, T. Paschoud, J. Reusser, B.-H. Gan, A. Luscher, T. Köhler, C. van Delden, J.-L. Reymond, *ACS Infect. Dis.* **2023**, *9*, 2593–2606.
[9] S. Baeriswyl, H. Personne, I. D. Bonaventura, T. Köhler, C. van Delden, A. Stocker, S. Javor, J.-L. Reymond, *RSC Chem. Biol.* **2021**, *2*, 1608–1617.
[10] B.-H. Gan, T. N. Siriwardena, S. Javor, T. Darbre, J.-L. Reymond, *ACS Infect. Dis.* **2019**, *5*, 2164–2173.
[11] X. Cai, M. Orsi, A. Capecchi, T. Köhler, C. van Delden, S. Javor, J.-L. Reymond, *Cell Rep. Phys. Sci.* **2022**, *3*, 101161.
[12] R. A. Moore, N. C. Bates, R. E. Hancock, *Antimicrob. Agents Chemother.* **1986**, *29*, 496–500.
[13] S. He, C. M. Deber, *Sci. Rep.* **2024**, *14*, 1894.
